# Supplementary material for: Bacterial effectors mediate kinase reprogramming through mimicry of conserved eukaryotic motifs
Source: EMBO Rep. 2025 May 12;26(14):3529–53. doi: 10.1038/s44319-025-00472-y (PMC12287357; doi:10.1038/s44319-025-00472-y)
Supplement: Supplementary file 1 — Appendix [file 44319_2025_472_MOESM1_ESM.pdf]

Appendix

Bacterial effectors mediate kinase reprogramming through mimicry of conserved eukaryotic motifs

Ioanna Panagi, Janina H. Muench, Alexi Ronneau, Ines Diaz-del-Olmo, Agnel Aliyath, Xiu-Jun Yu, Hazel Mak, Enkai Jin, Jingkun Zeng, Diego Esposito, Elliott Jennings, Timesh D. Pillay, Regina A. Günster, Sarah L. Maslen, Katrin Rittinger, Teresa L.M. Thurston.

Table of content

Appendix Table S1 page 2
Appendix Table S2 page 6
Appendix Table S3 page 8

Appendix Table S1: List of oligonucleotides used in this study.

ID	Sequence (5' to 3')	Forward/ Reverse	Restriction Site/Cloning Method	Purpose
**External primers for cloning in ptCMV-GFP** (N-terminal GFP tag, mammalian expression plasmid)				
EJ394	CGCGGGCCATGGCAATGTT<br>TACAATTAATAGTACTAA	F	NcoI	SteE
Trx601	CGCGGGGCGGCCGCTTATT<br>CATCCGGGAAAACCTCTG	R	NotI	SteE
Trx651	CGCGGG ACATGT CA<br>GATGTTAATTTAGAGGAC	F	PciI	SteEΔN20
Trx738	CGCGGGGCGGCCGCTTATT<br>CATCCGGGAAAACCTCTGC<br>AGAATGCCTGTATTGAGCG<br>ATAAAACCAGCCGGTGGGT<br>TATGACTGGC	R	NotI	SteE$^{S141A / Y143F}$
EJ670	CATGACATGTCAATGCTGC<br>GTAGCCAGCG	F	PciI	Epc06920
EJ671	CATGGCGGCCGCTTACAGC<br>GGCAGGCTGGTGC	R	NotI	Epc06920
EJ672	CATGACATGTCAATGGTGG<br>AATTCGTGTATAACG	F	PciI	KML20850
EJ673	CATGGCGGCCGCTTACACA<br>TAACCATTGCTTTTCACG	R	NotI	KML20850
EJ666	CATGACATGTCAATGATCAG<br>CAACAACATGATC	F	PciI	ALT06054
EJ667	CATGGCGGCCGCTTACACA<br>TAGCCATTTGCG	R	NotI	ALT06054
EJ674	CATGACATGTCAATGCTGA<br>GCAGCCGTT	F	PciI	SAMN04490206
EJ675	CATGGCGGCCGCTTAGGTG<br>GTCATAACGCTACCA	R	NotI	SAMN04490206
EJ668	CATGACATGTCAATGAGCAA<br>CAAGATCAACATC	F	PciI	ArsFin19530
EJ669	CATGGCGGCCGCTTAATCC<br>AGTTCTTTCATTTTGCA	R	NotI	ArsFin19530

**Internal primers for mutagenesis by overlap PCR**<br>(combined with appropriate external primers)				
IOP093	ATTGTTTTTCTAgcCGGCACC CCTGCAGTTTTAGAGACT	F	None	SteE^{L89A}
IOP094	AGTCTCTAAAACTGCAGGGG TGCCGgcTAGAAAAACAAT	R		
IOP095	ATTGTTTTTCTACTCGcCACC CCTGCAGTTTTAGAGACT	F	None	SteE^{G90A}
IOP096	AGTCTCTAAAACTGCAGGGG TGgCGAGTAGAAAAACAAT	R		
JZ019	ATTGTTTTTCTACTCGGCGC GCCTGCAGTTTTAGAGACT	F	None	SteE^{T91A}
JZ020	AGTCTCTAAAACTGCAGGC GCGCCGAGTAGAAAAACAA T	R		
IOP097	ATTGTTTTTCTACTCGGCACC gCTGCAGTTTTAGAGACT	F	None	SteE^{P92A}
IOP098	AGTCTCTAAAACTGCAGcGG TGCCGAGTAGAAAAACAAT	R		
Trx660	GTTTCCCCGGATGCTTTAAC CGCAGCAGCATTAAGCATT AGTAGCAATGAT	F	None	SteE$^{QKL\text{-}AAA}$
Trx661	ATCATTGCTACTAATGCTTA ATGCTGCTGCGGTTAAAGC ATCCGGGGAAAC	R		
Trx653	CCTGCAGTTTTAGAGACTG CAGCGGCTGCATCATTACC AGTTTCCCCG	F	None	SteE$^{KESL\text{-}AAAA}$
Trx654	CGGGGAAACTGGTAATGAT GCAGCCGCTGCAGTCTCTA AAACTGCAGG	R		
Trx670	CAGTTTTAGAGACTAAAGAG GCTGCAGCAGCACCAGTTT CCCCGGATGC	F	None	SteE$^{SLSL\text{-}AAAA}$
Trx671	GCATCCGGGGAAACTGGTG CTGCTGCAGCCTCTTTAGTC TCTAAAACTG	R		
Trx655	GAGACTAAAGAGTCTTTA GCAGCAGCAGCT TCCCCGGATGCTTTAACC	F	None	SteE$^{SLPV\text{-}AAAA}$
Trx656	GGTTAAAGCATCCGGGGAA GCTGCTGCTGCTAAAGACT CTTTAGTCTC	R		
Trx699	TCTTTATCATTACCAGTTTTC TCAGATGCTTTAACCCAAAA A	F	None	SteE$^{SP\text{-}FS}$
Trx700	TTTTTGGGTTAAAGCATCTG AGAAAACTGGTAATGATAAA GA	R		

**External primers for cloning in pWSK29**				
EJ399B	CATGGAATTCCTTGTGGTTTTCCTTAGGAGGTA	F	EcoRI	SteE + 25 bp SD sequence
EJ400	CATGGGATCCTTCATCCGGGAAAACCTCTGC	R	BamHI	SteE, no stop codon
IOP034	CATGGGATCCTTCATCCGGGAAAACCTCTGCAGAATGCCTGTATTGAGCGATATAACCAGCCGGTGGGTTATGACTGGC	R	BamHI	$SteE^{S141A}$, no stop codon
IOP035	CATGGGATCCTTCATCCGGGAAAACCTCTGCAGAATGCCTGTATTGAGCGATAAAACCGGACGGTGGGTTATGACTGGC	R	BamHI	$SteE^{Y143F}$, no stop codon
**Primers for protein expression vectors** (Gibson Assembly)				
N/A	TAACGCTCTGGTGCCACGCGGTAGTAAAGAAACCGC	F	Gibson Assembly	pET49-His_6-3C
N/A	ACCGGGTCCCTGAAAGAGGACTTCAAGAGCCGCGGA	R	Gibson Assembly	pET49-His_6-3C
N/A	CCTCTTTCAGGGACCCGGTAGCGATGTTAATTTAGAGGAC	F	Gibson Assembly	$SteE^{\Delta N20}$, assembly into pET49-His_6-3C vector
N/A	CCGCGTGGCACCAGAGCGTTATTATTCATCCGGGAAAACCTCTG	R	Gibson Assembly	$SteE^{\Delta N20}$, assembly into pET49-His_6-3C vector
N/A	CCTCTTTCAGGGACCCGGTCAGGCCAACCACCCCACAGCAGCCGTG	F	Gibson Assembly	$STAT3^{127\text{-}715}$, assembly into pET49-His_6-3C
N/A	CCGCGTGGCACCAGAGCGTTATTATGGTGTCACACAGATAAACTTGGTCTT	R	Gibson Assembly	$STAT3^{127\text{-}715}$, assembly into pET49-His_6-3C
N/A	CCGAAGCGCGCGGAATTCAAAGGC	F	Gibson Assembly	pACEBAC1 forward primer (used for GSK3β/ $GSK3β^{S9A}$)
N/A	ACCGGGATCCGCGCCCGATGG	R	Gibson Assembly	pACEBAC1 reverse primer (used for GSK3β)
N/A	GGGCGCGGATCCCGGTATGTCTAGTGGTTCTGGTCATCACCATCAC	F	Gibson Assembly	His_6-3C-GSK3β, assembly into pACEBAC1
N/A	TTTGAATTCCGCGCGCTTCGGTCAGGTGGAGTTGGAAGCTGATG	R	Gibson Assembly	GSK3β, assembly into pACEBAC1
N/A	CCTCTTTCAGGGACCCGGTATGTCAGGGCGGCCCAGAAC	F	Gibson Assembly	$GSK3β^{S9A}$, assembly into pACEBAC1-His_6-3C

N/A	GCTCAGAAGATCGAGTGGCACGAGTCCGCGGCTCTTGAAGTCCTCTTT	F	Gibson Assembly	Insertion of Avi-tag for pACEBAC1-His_6-Avi-3C-GSK3β^{S9A}
N/A	CTCGAAGATGTCGTTCAGACCGGAACCGTGATGGTGATGGTGATGACCAGAACC	R	Gibson Assembly	Insertion of Avi-tag for pACEBAC1-His_6-Avi-3C-GSK3β^{S9A}

F = forward primer; R = reverse primer; bp = base pairs; SD = Shine-Dalgarno; N/A = not applicable

Appendix Table S2: List of synthesized genes.

Target	Sequence (5’ to 3’)
ALT06054	ATGATCAGCAACAACATGATCAAGTGCAACGTGTTTCATGATCTGCATATT<br>CCGAATGATGCCGCAGAAAGTGATGTTGAAACCTATAAAAACGATCTGAG<br>CGAACGTATCAGCTGCGAATATCATACCGATAGCAACGAAGTTTTTATTCT<br>GGCAAGTCCGGAAGAACTGGAAGATACCGAAAGCCTGAGCCTGGCAGTTA<br>GCCCGAAAGCACTGCATCAGGCAATTAGCTGTGAACTGGCAAAAATGACC<br>GATCACGATCTGCGTGAACGTGATATGGTTGAAACCGGCAAAGAAATCAA<br>ACCGGAAGAAGATGTTACCAAACTGCACGAATACATTATTCGCGCAAATG<br>GCTATGTGTAA
ArsFin19530	ATGAGCAACAAGATCAACATCACCAAAGATCGTCCGCTGATTAAAAACAGC<br>CAGCAGCAGAATCAGCTGAAAACCAATGCACTGATCAGCAAAATCAAAAA<br>CTGCTTCGTGCTGAACAACATCCATATTAGCACCACCATTGGTAGCAGCAA<br>AGCCAAATTCTATCACCAGCTGATGCTGCGTCTGAATCTGGATAGCAGCAT<br>TCAGATCAAAAATTACCATCACTATCATCTGACCAGTCGCCTGAACCTGGA<br>ATATAACAGCACCACCGAAAATGTTGCACTGCAGGGCACCCCGAAAAACA<br>TTCATAGCGCAGAAAGCCTGAGCCTGCCGGTTTGTCCGTTTCTGCTGAGCC<br>AGAAACTGATTCAGGTGAAAAACAAGTTCACCAAAAAAAAGAAATGCAAA<br>ATGAAAGAACTGGAT
Epc06920	ATGCTGCGTAGCCAGCGTTTTCTGAGCATTCCGAAACTGGATTATATCTGC<br>GATCATGTGATGGAAGAAAACATGCTGGCAATTCATAATCTGAACCGTATT<br>GAAACCGCAGTTGTGAAACACGTTAATTGCGCAAATGTTGAAGGTAAACC<br>GACCTTTGTTGCAAGCGTTAGCAGCGTTGAAAATGTTAGCACCGATATGCT<br>GAAACCGAAAATCTTTGCACAGTGCGTGATTCTGAATAATCTGCATGTTCC<br>GCAGAATATTCCGGATACCGATATTGAAGGTTACAACAAAGGCATGCAGG<br>TTCGCATTAATCAAGAATATCAGCCGCAGGGTAAAACCGTTTTTCTGCTGG<br>GTAGTCCGGAAGTTCTGGAACCGGATGAAAGCCTGAGCCTGCCTGCAAGT<br>CCGCATATTCTGGCACAGAAACTGAGCAGCATTGCAAACATTAAAGCATGC<br>GCATTTAGCTTTGAAAGCAATGGTTATGTTCAGCGCAGCGAAGATAATTTC<br>ATTTATCGTAATGGCACCAGCCTGCCGCTG
KML20850	ATGGTGGAATTCGTGTATAACGAACTGAATACCCTGCGTAAAGGCACCGC<br>ACTGGGTAGCGTTTGGGAAAGCAATATGTTTACCATTATTACCAATGCCGA<br>TCGTATGGCAGCAGCAGCCGCAGATACCCATCTGAATCATGTTAATGTTGA<br>AGGTAAAAGCGGTGTGGTTAATCTGGTTGTTAGCGTTAAAAACGATCCGA<br>TCAATATGCTGCGTCCGGATAACTTTAGCCAGTGTGTTATTCTGAATAATCT<br>GCATGTTCCGCAGGGTGCACTGGTTACCGATATTGATAACTATAACAAAGG<br>TCTGCAGCTGCGCATTAACCTGGAATATAATCCGAAAGGTAGCATCGTTTT<br>TCTGCTGGGTAGTCCGGAAGCACTGGATGCAAATGAAAGCCTGAGCCTGC<br>CGATTTTTAGCCATGTTCTGACCCAGAAACTGCTGAACATTAGCAATAGCA<br>AACTGTGTGAACTGAGCGTGAAAAGCAATGGTTATGTG

SAMN04490206	ATGCTGAGCAGCCGTTTTGATAGCGTTATTAGCAGCAATCAGGTTCTGAGC<br>GAACATACCGTTGAAGGTGCAGGTAGCCAGCGTGCAAGCGTGCTGAGTGA<br>AAATACCCTGAGCGTTCAGCAGACCAGCCGTAGCATGTGTAAAGCAAATG<br>ATGCCGGTGTTGATCGTCAGTGTATTACCAATGCATGTGAAGTGAATGCCG<br>AAAATAATGGTGTTCGTGTTCTGATGAGCGCACTGGTTAGCGTTGGTAATG<br>TTGTTAGCACCGGTTTTGTTCGCCAAGAAATCAATAAAGTGATCGTGCGTG<br>ATGATATTCATGTTCCGCAGGGTAGCATGCCGCATGATCGTAAAATGTGTG<br>ATGAAGGTCAGCGTATTCGTCTGAATGATGAGTATCATCCGCTGCATCATA<br>CCGTTTTTCTGCAGGGTACACCGGAACGTCTGGGTATTCATCATCAGCTGA<br>GCCTGCCGGTTAGCCCGAGCATGCTGACCGAAAAACTGATTGAAGTTATCC<br>GCGAGAAGAACGAAAAAGAACAGCGTGCCAGCCAGGCAGAAAAAAATGG<br>TTATGTTTGTCAGGTTGGTAGCGTTATGACCACC

Appendix Table S3: List of amino acid sequences for protein alignments.

Protein	Amino Acid Sequence
STM2585	MFTINSTNRVASTIAPYACVSDVNLEDKATFLDEHTSIHANDSSLQCFVLNDQ<br>HVPQNTLATDVEGYNRGLQERISLEYQPLESIVFLLGTPAVLETKESLSLPVSP<br>DALTQKLLSISSNDECKLSGSTSCTTPASHNPPSGYIAQYRHSAEVFPDE
ALT06054	MISNNMIKCNVFHDLHIPNDAAESDVETYKNDLSERISCEYHTDSNEVFILASP<br>EELEDTESLSLAVSPKALHQAISCELAKMTDHDLRERDMVETGKEIKPEEDVT<br>KLHEYIIRANGYV
ArsFin19530	MSNKINITKDRPLIKNSQQQNQLKTNALISKIKNCFVLNNIHISTTIGSSKAKFYH<br>QLMLRLNLDSSIQIKNYHHYHLTSRLNLEYNSTTENVALQGTPKNIHSAESLSL<br>PVCPFLLSQKLIQVKNKFTKKKKCKMKELD
Epc06920	MLRSQRFLSIPKLDYICDHVMEENMLAIHNLNRIETAVVKHVNCANVEGKPTF<br>VASVSSVENVSTDMLKPKIFAQCVILNNLHVPQNIPDTDIEGYNKGMQVRINQ<br>EYQPQGKTVFLLGSPEVLEPDESLSLPASPHILAQKLSSIANIKACAFSFESNG<br>YVQRSEDNFIYRNGTSLPL
KML20850	MVEFVYNELNTLRKGTALGSVWESNMFTIITNADRMAAAAADTHLNHVNVEG<br>KSGVVNLVVSVKNDPINMLRPDNFSQCVILNNLHVPQGALVTDIDNYNKGLQL<br>RINLEYNPKGSIVFLLGSPEALDANESLSLPIFSHVLTQKLLNISNSKLCELSVK<br>SNGYV
SAMN04490206	MLSSRFDSVISSNQVLSEHTVEGAGSQRASVLSENTLSVQQTSRSMCKAND<br>AGVDRQCITNACEVNAENNGVRVLMSALVSVGNVVSTGFVRQEINKVIVRDDI<br>HVPQGSMPHDRKMCDEGQRIRLNDEYHPLHHTVFLQGTPERLGIHHQLSLP<br>VSPSMLTEKLIEVIREKNEKEQRASQAEKNGYVCQVGSVMTT
