# Supplementary material for: Bacterial effectors mediate kinase reprogramming through mimicry of conserved eukaryotic motifs
Source: EMBO Rep. 2025 May 12;26(14):3529–53. doi: 10.1038/s44319-025-00472-y (PMC12287357; doi:10.1038/s44319-025-00472-y)
Supplement: Supplementary file 3 — Source data Fig. 1 [file 44319_2025_472_MOESM3_ESM.zip › Figure 1/1B/1B_readme.pptx]

## Slide 1
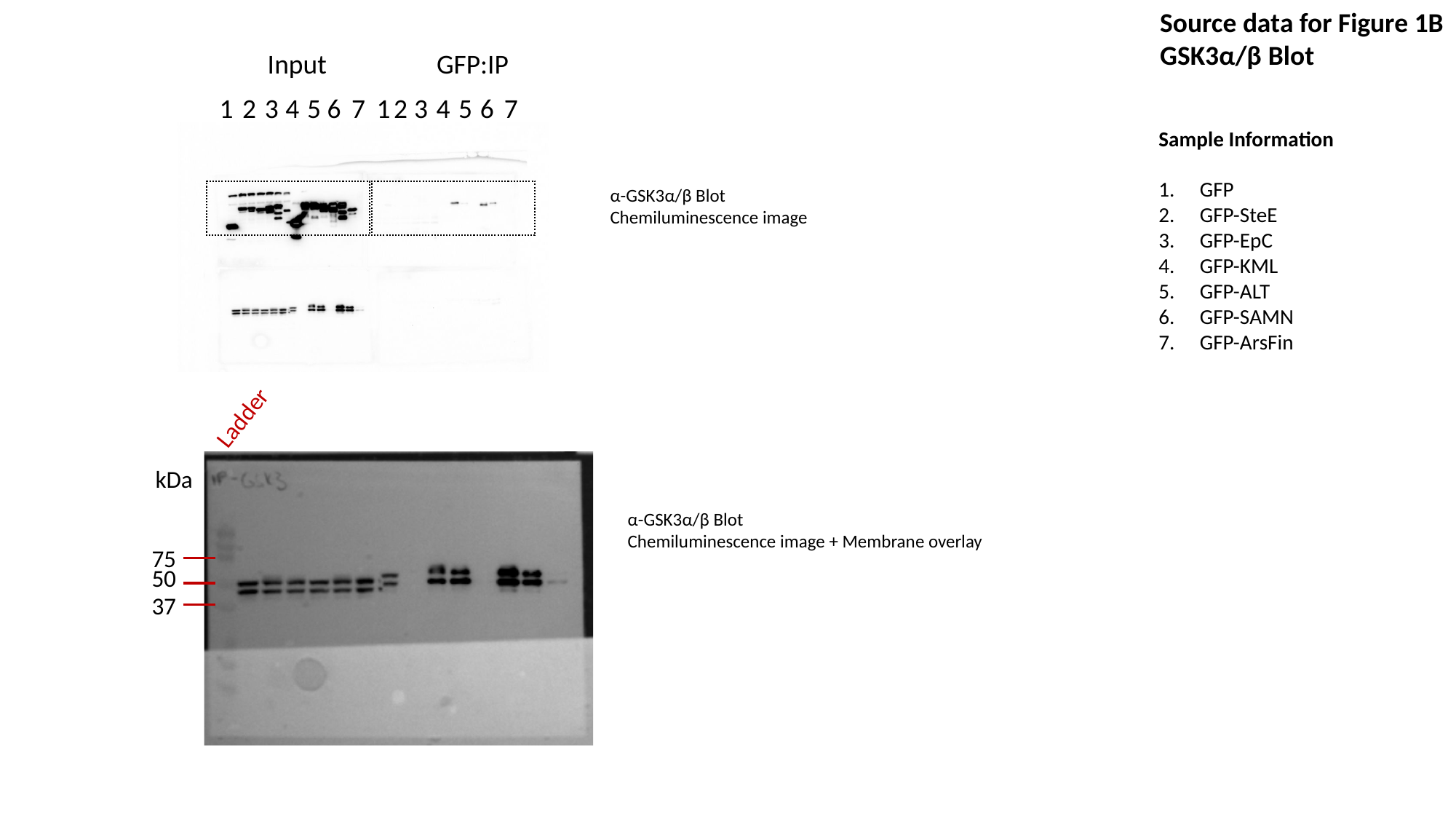

Source data for Figure 1B
GSK3α/β Blot
Input
GFP:IP
1
2
3
4
5
6
7
1
2
3
4
5
6
7
α-GSK3α/β Blot
Chemiluminescence image
Sample Information
GFP
GFP-SteE
GFP-EpC
GFP-KML
GFP-ALT
GFP-SAMN
GFP-ArsFin
Ladder
kDa
α-GSK3α/β Blot
Chemiluminescence image + Membrane overlay
75
50
37

## Slide 2
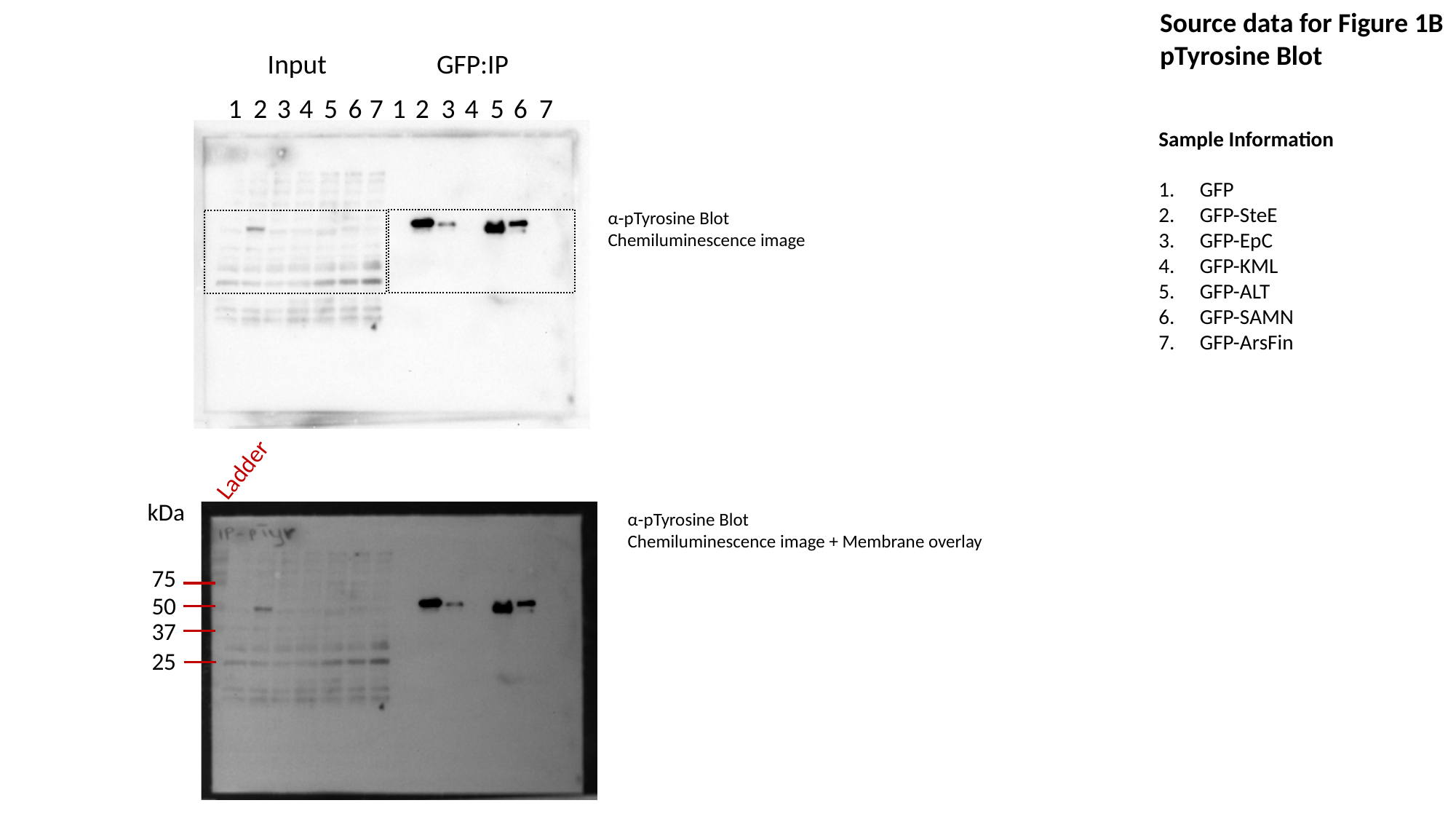

Source data for Figure 1B
pTyrosine Blot
Input
GFP:IP
1
2
3
4
5
6
7
1
2
3
4
5
6
7
α-pTyrosine Blot
Chemiluminescence image
Sample Information
GFP
GFP-SteE
GFP-EpC
GFP-KML
GFP-ALT
GFP-SAMN
GFP-ArsFin
Ladder
kDa
α-pTyrosine Blot
Chemiluminescence image + Membrane overlay
75
50
37
25

## Slide 3
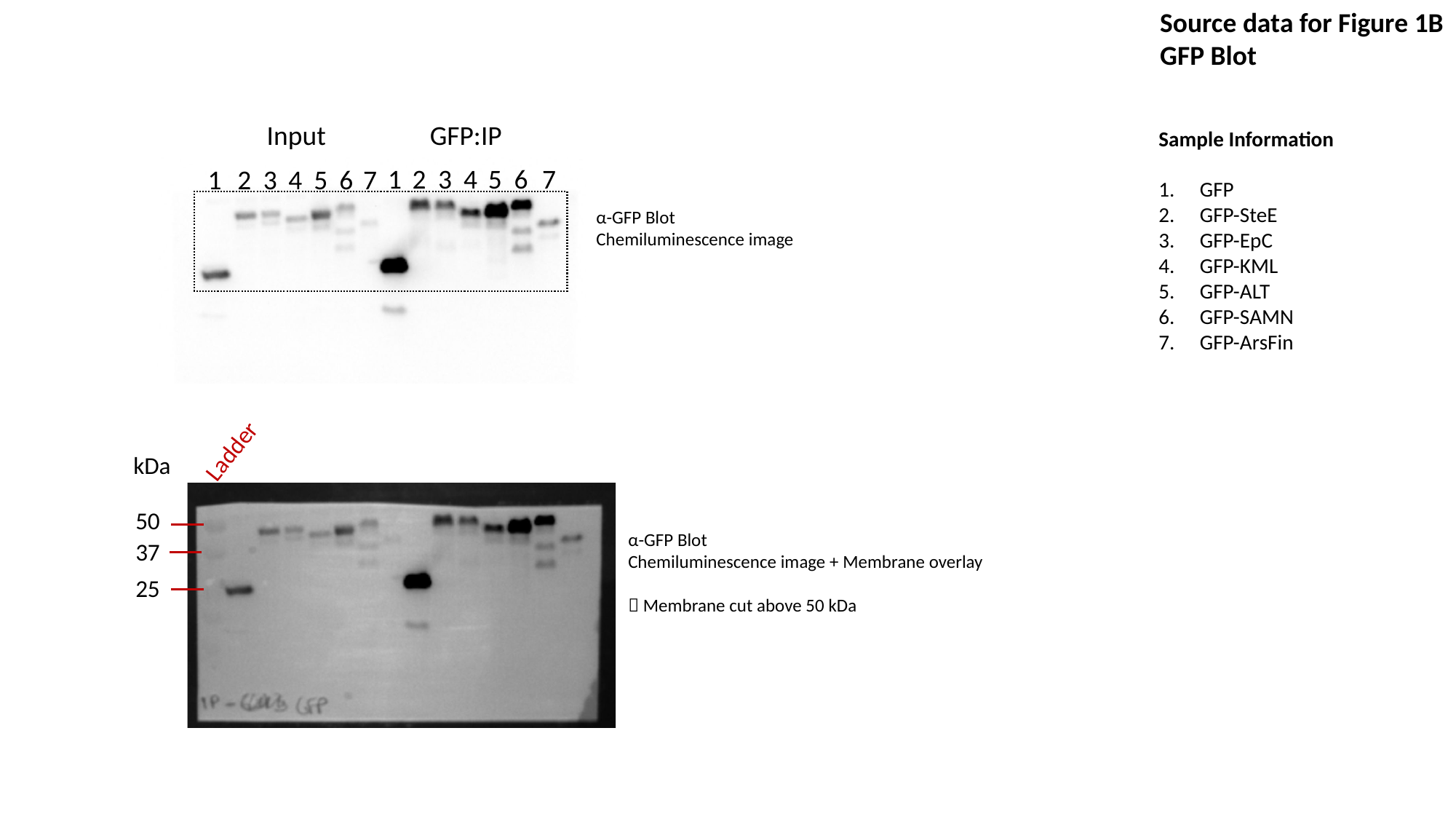

Source data for Figure 1B
GFP Blot
Input
GFP:IP
1
2
3
4
5
6
7
1
2
3
4
5
6
7
α-GFP Blot
Chemiluminescence image
Sample Information
GFP
GFP-SteE
GFP-EpC
GFP-KML
GFP-ALT
GFP-SAMN
GFP-ArsFin
Ladder
kDa
50
α-GFP Blot
Chemiluminescence image + Membrane overlay
 Membrane cut above 50 kDa
37
25
