# Supplementary material for: Bacterial effectors mediate kinase reprogramming through mimicry of conserved eukaryotic motifs
Source: EMBO Rep. 2025 May 12;26(14):3529–53. doi: 10.1038/s44319-025-00472-y (PMC12287357; doi:10.1038/s44319-025-00472-y)
Supplement: Supplementary file 3 — Source data Fig. 1 [file 44319_2025_472_MOESM3_ESM.zip › Figure 1/1C/1C_readme.pptx]

## Slide 1
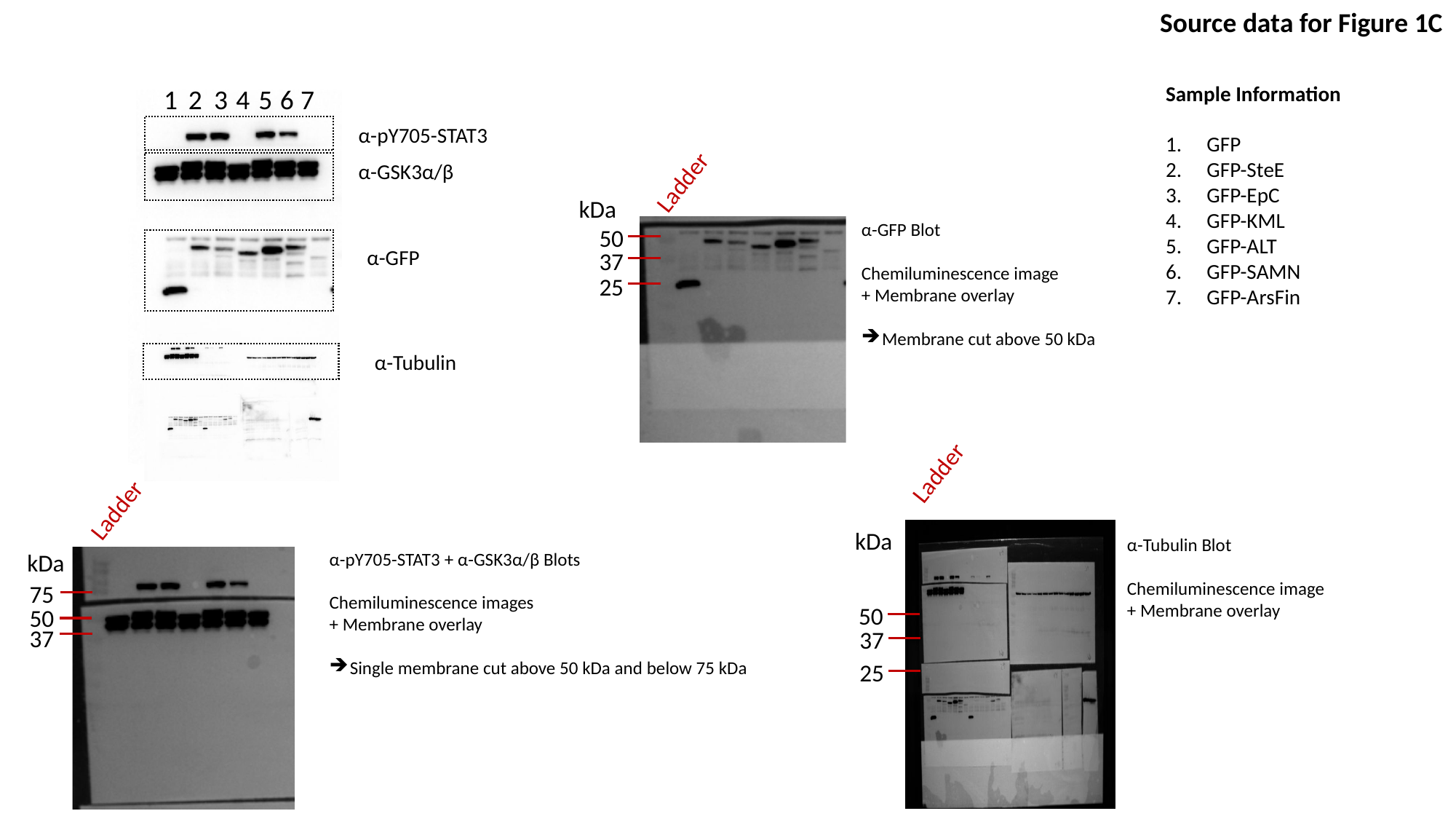

Source data for Figure 1C
Sample Information
GFP
GFP-SteE
GFP-EpC
GFP-KML
GFP-ALT
GFP-SAMN
GFP-ArsFin
1
2
3
4
5
6
7
α-pY705-STAT3
α-GSK3α/β
α-GFP
Ladder
α-GFP Blot
Chemiluminescence image
+ Membrane overlay
Membrane cut above 50 kDa
50
37
25
kDa
α-Tubulin
Ladder
α-Tubulin Blot
Chemiluminescence image
+ Membrane overlay
kDa
50
37
25
Ladder
α-pY705-STAT3 + α-GSK3α/β Blots
Chemiluminescence images
+ Membrane overlay
Single membrane cut above 50 kDa and below 75 kDa
kDa
75
50
37
