# Supplementary figures and images for: Bacterial effectors mediate kinase reprogramming through mimicry of conserved eukaryotic motifs
Source: EMBO Rep. 2025 May 12;26(14):3529–53. doi: 10.1038/s44319-025-00472-y (PMC12287357; doi:10.1038/s44319-025-00472-y)

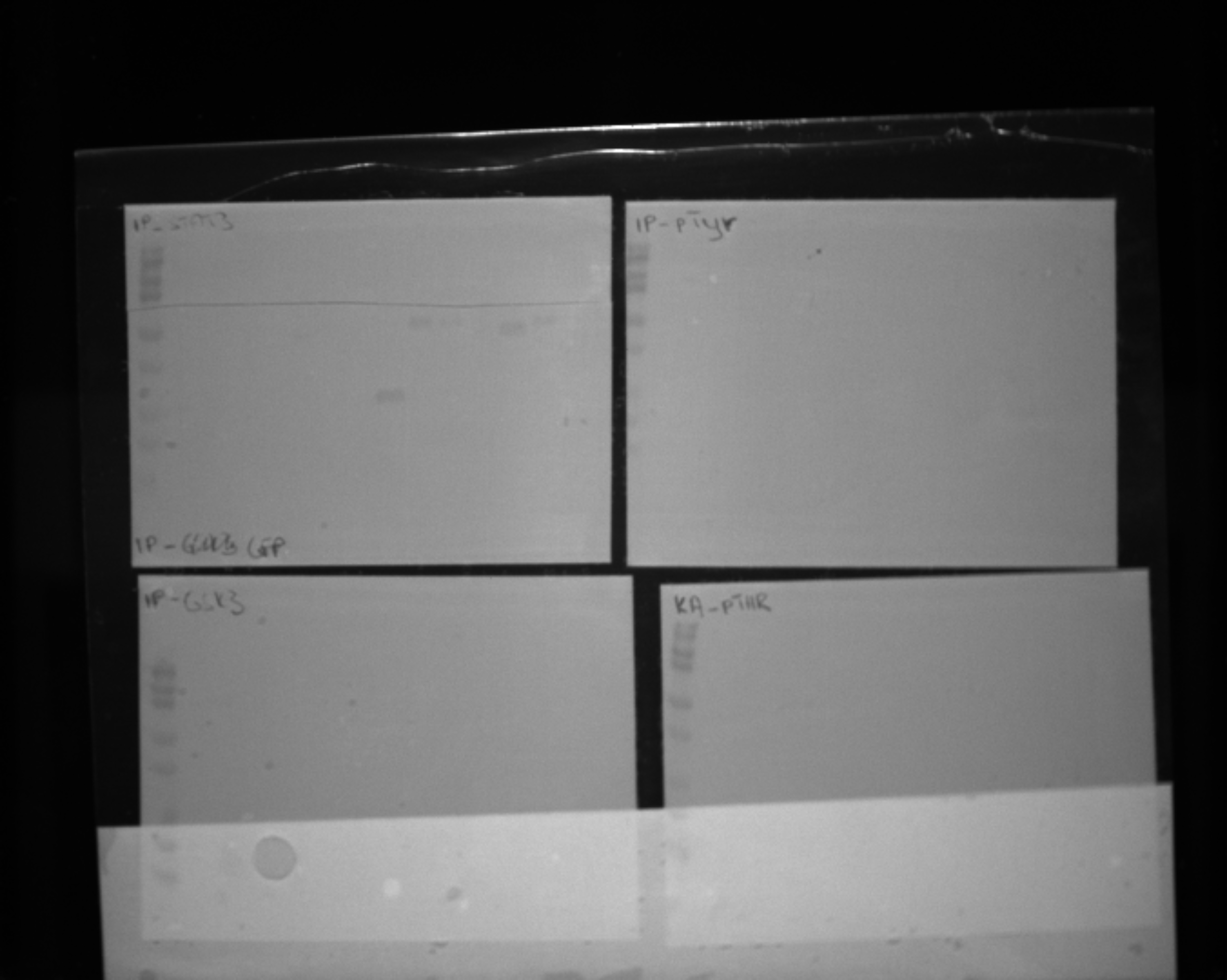

Supplement: Supplementary file 3 — Source data Fig. 1 [file 44319_2025_472_MOESM3_ESM.zip › Figure 1/1B/Ladder+pTyrosine/LadderpTyrosineMembrane.tif]

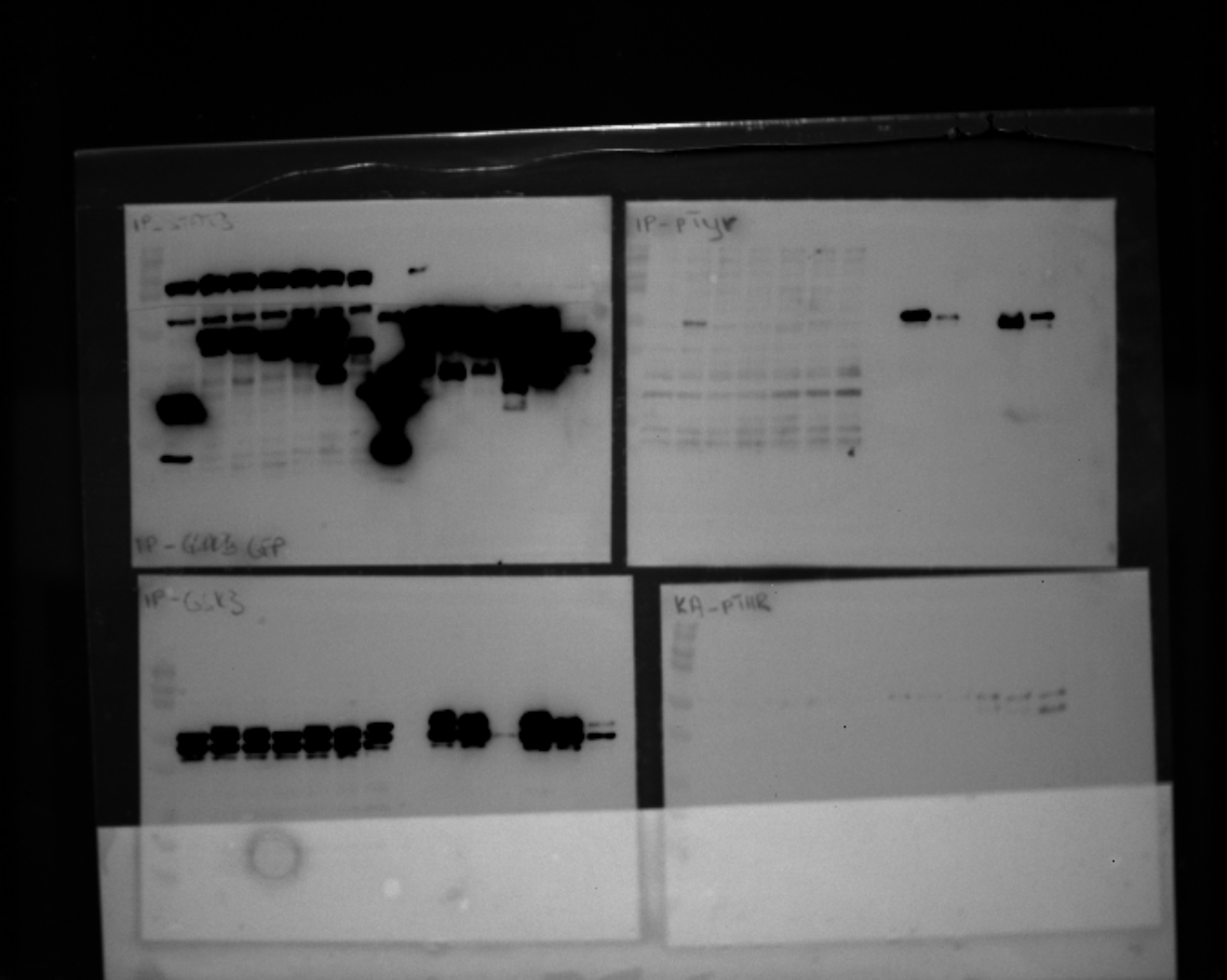

Supplement: Supplementary file 3 — Source data Fig. 1 [file 44319_2025_472_MOESM3_ESM.zip › Figure 1/1B/Ladder+pTyrosine/LadderpTyrosine_composite.tif]

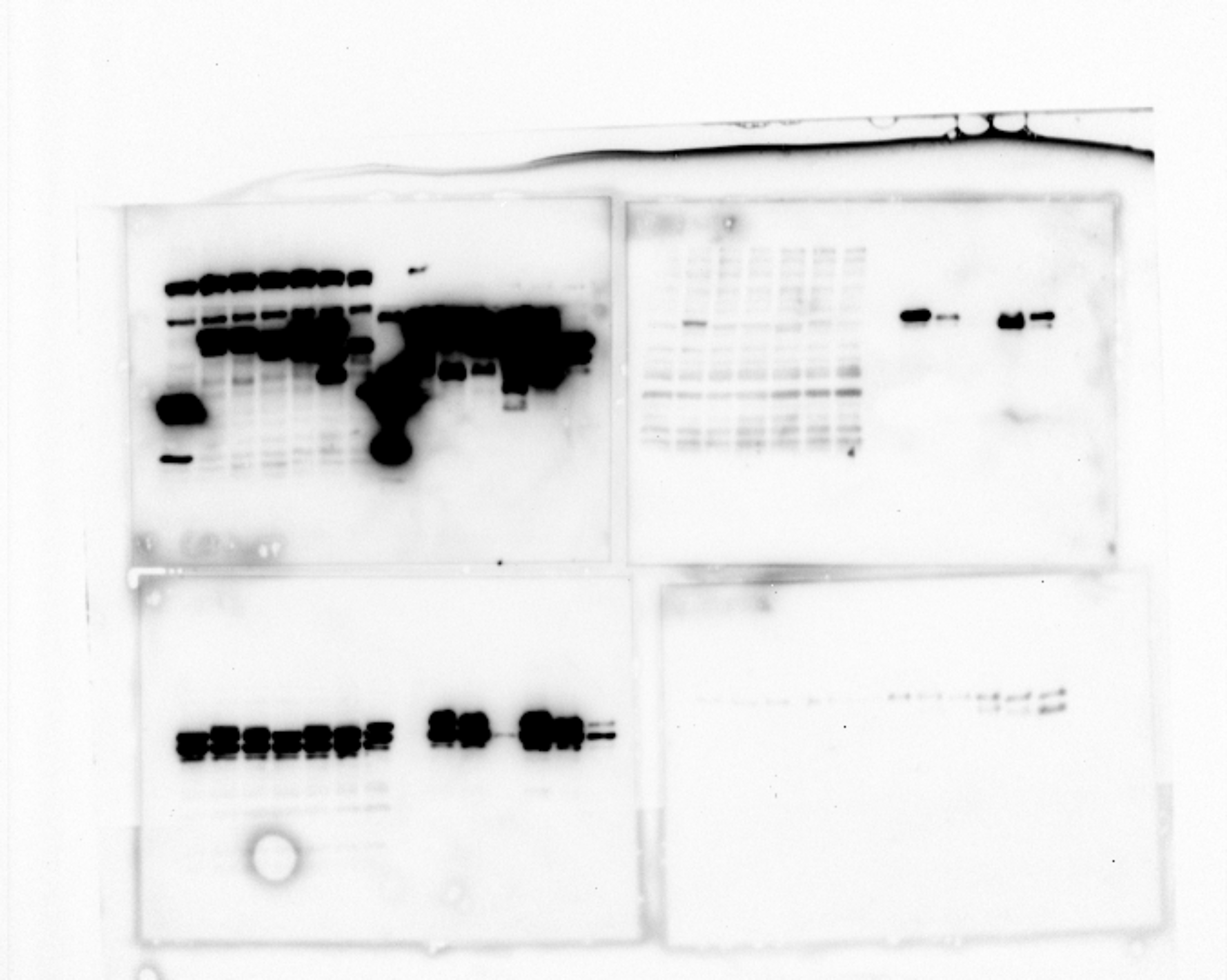

Supplement: Supplementary file 3 — Source data Fig. 1 [file 44319_2025_472_MOESM3_ESM.zip › Figure 1/1B/Ladder+pTyrosine/LadderpTyrosineChemi.tif]

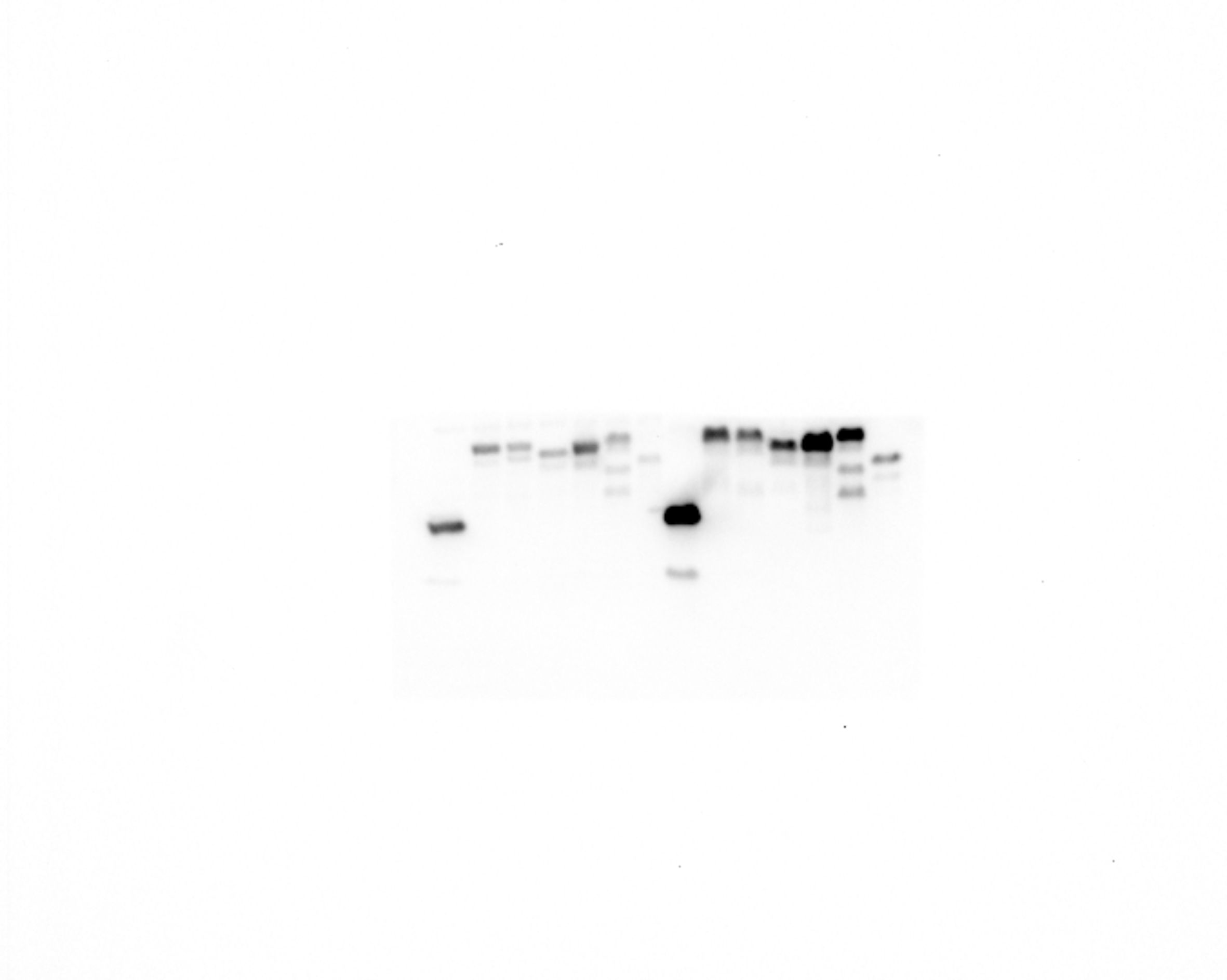

Supplement: Supplementary file 3 — Source data Fig. 1 [file 44319_2025_472_MOESM3_ESM.zip › Figure 1/1B/Ladder+GFP/LadderGFPChemi.tif]

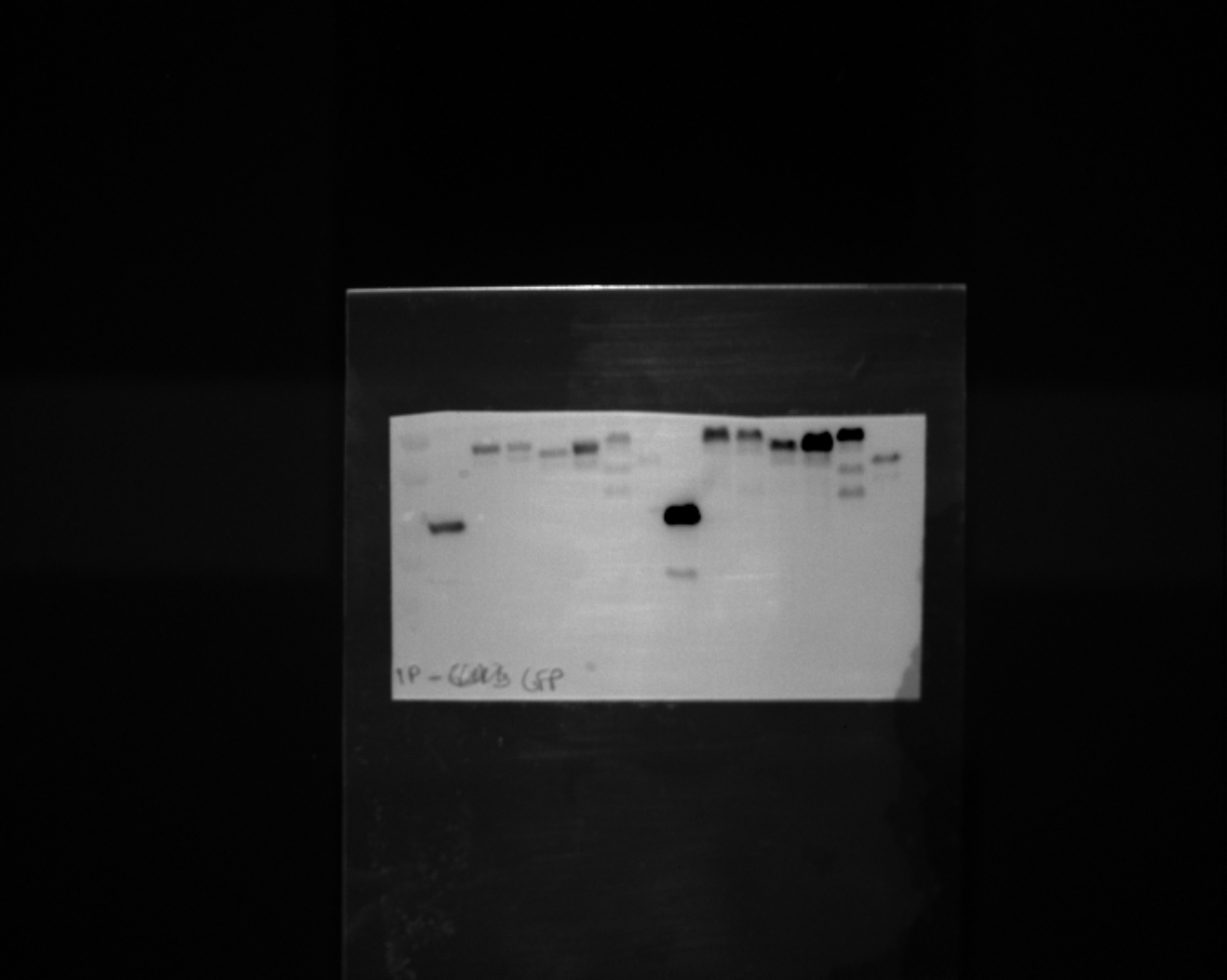

Supplement: Supplementary file 3 — Source data Fig. 1 [file 44319_2025_472_MOESM3_ESM.zip › Figure 1/1B/Ladder+GFP/LadderGFP_composite.tif]

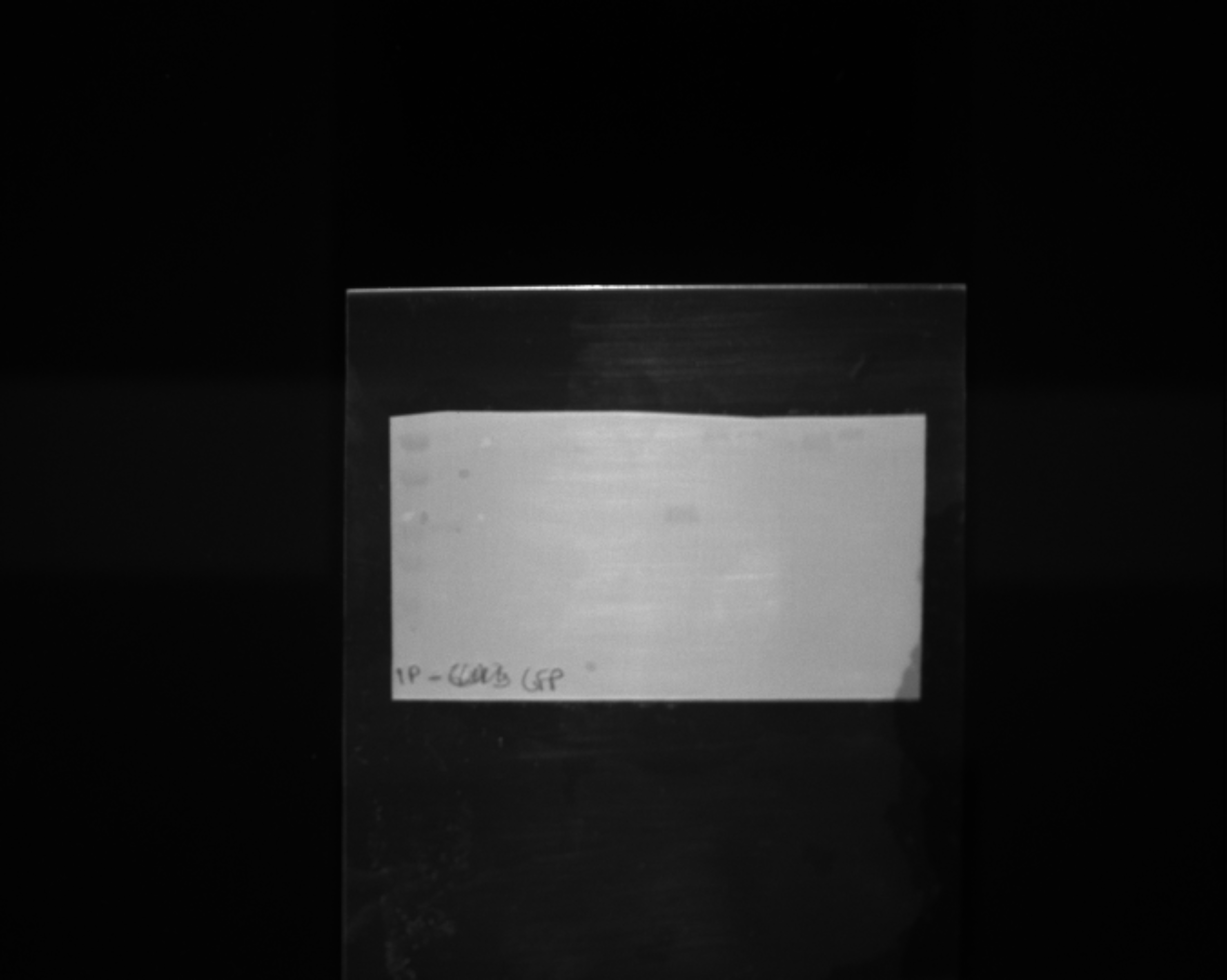

Supplement: Supplementary file 3 — Source data Fig. 1 [file 44319_2025_472_MOESM3_ESM.zip › Figure 1/1B/Ladder+GFP/LadderGFPMembrane.tif]

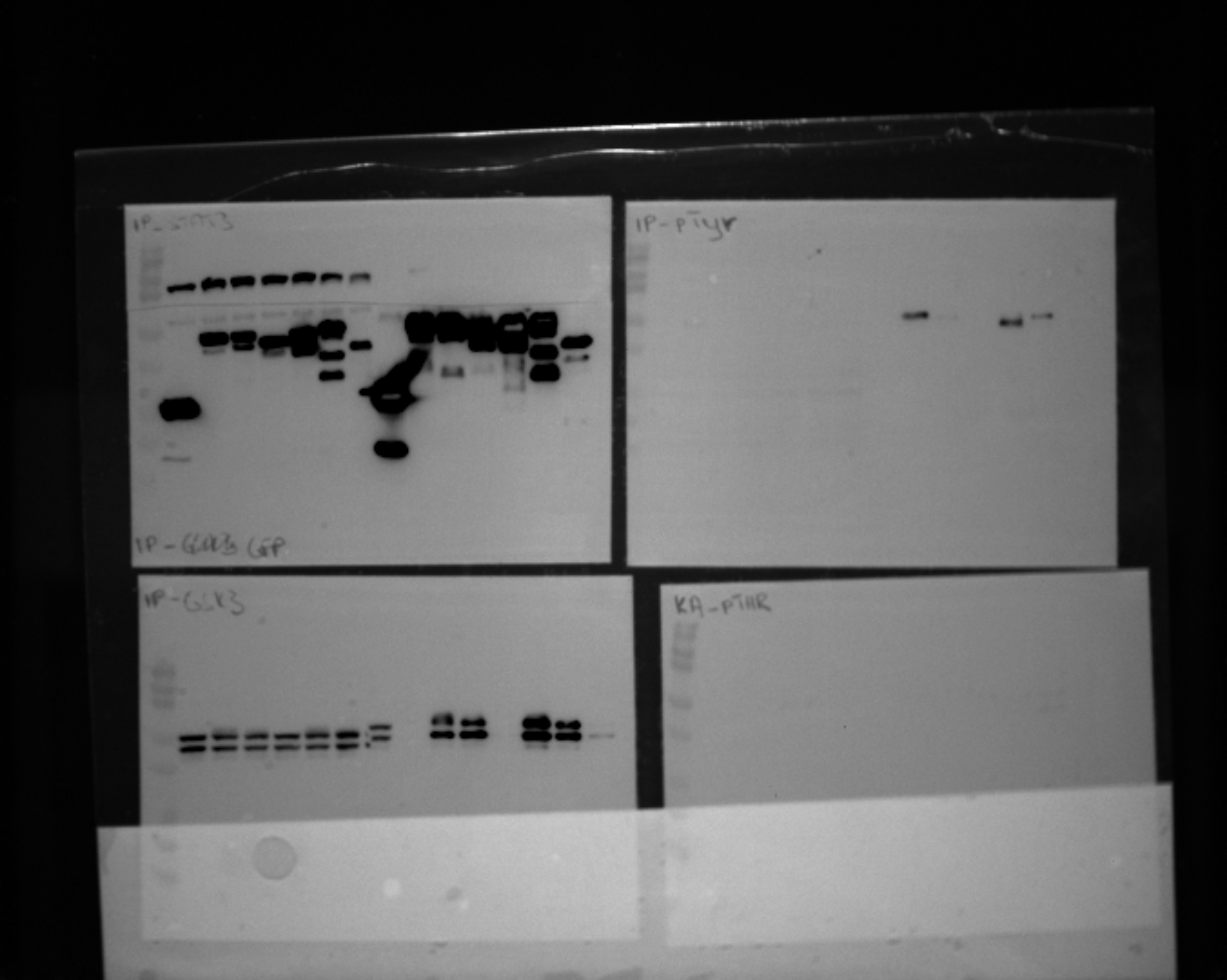

Supplement: Supplementary file 3 — Source data Fig. 1 [file 44319_2025_472_MOESM3_ESM.zip › Figure 1/1B/Ladder+GSK3/LadderGSK3_composite.tif]

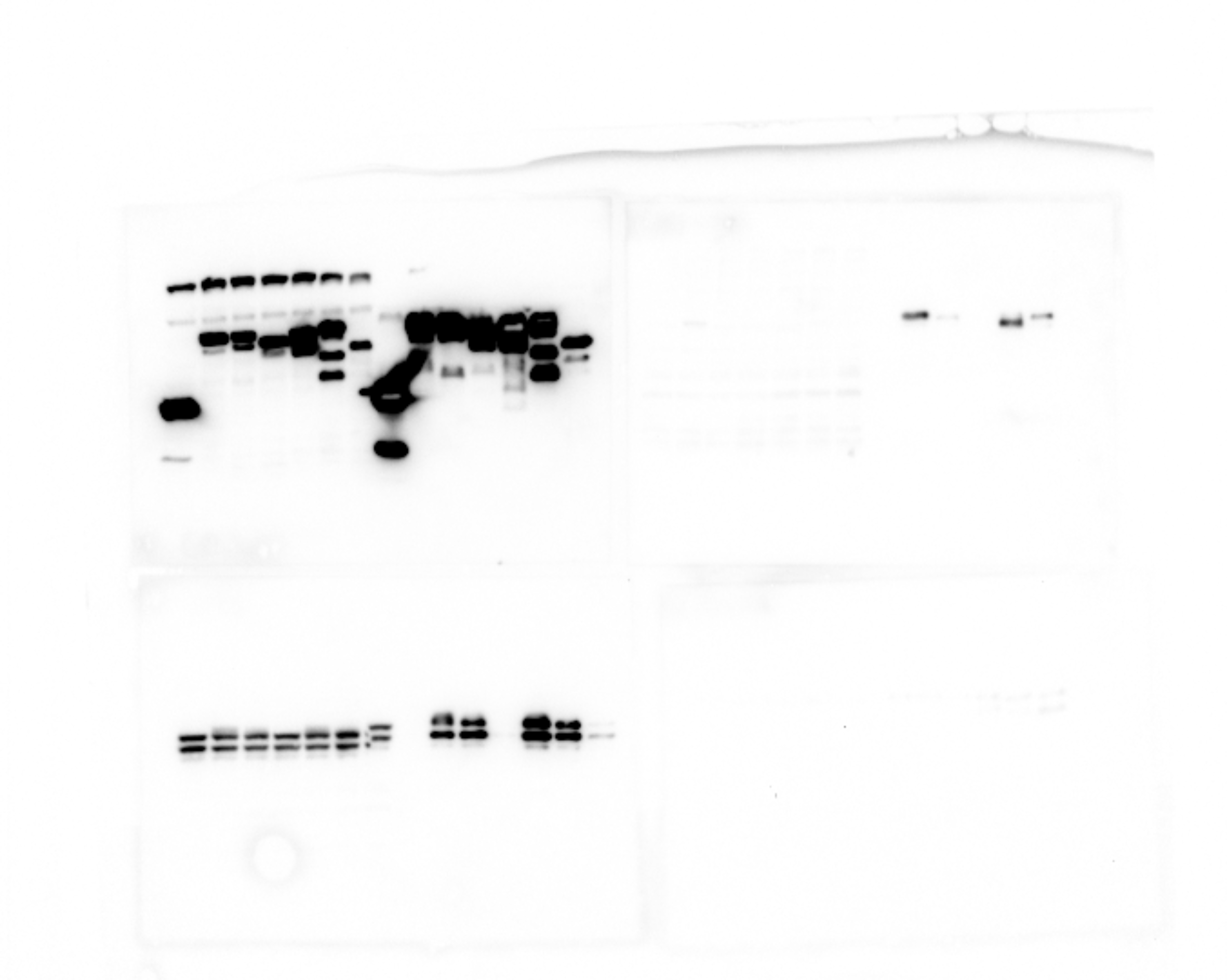

Supplement: Supplementary file 3 — Source data Fig. 1 [file 44319_2025_472_MOESM3_ESM.zip › Figure 1/1B/Ladder+GSK3/LadderGSK3Chemi.tif]

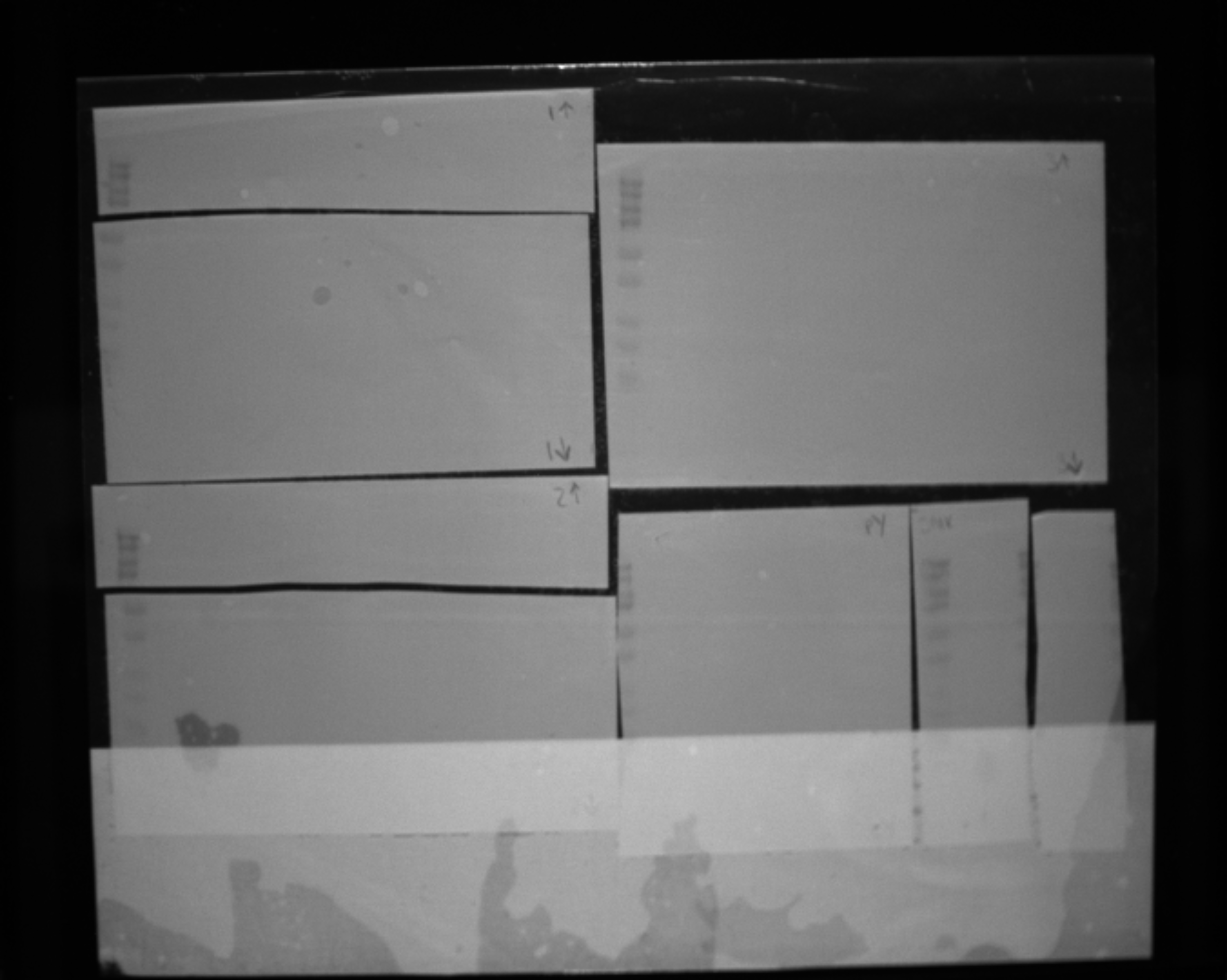

Supplement: Supplementary file 3 — Source data Fig. 1 [file 44319_2025_472_MOESM3_ESM.zip › Figure 1/1C/Ladder+GSK3+GFP_Overlay/LadderGSK3GFP_OverlayMembrane.tif]

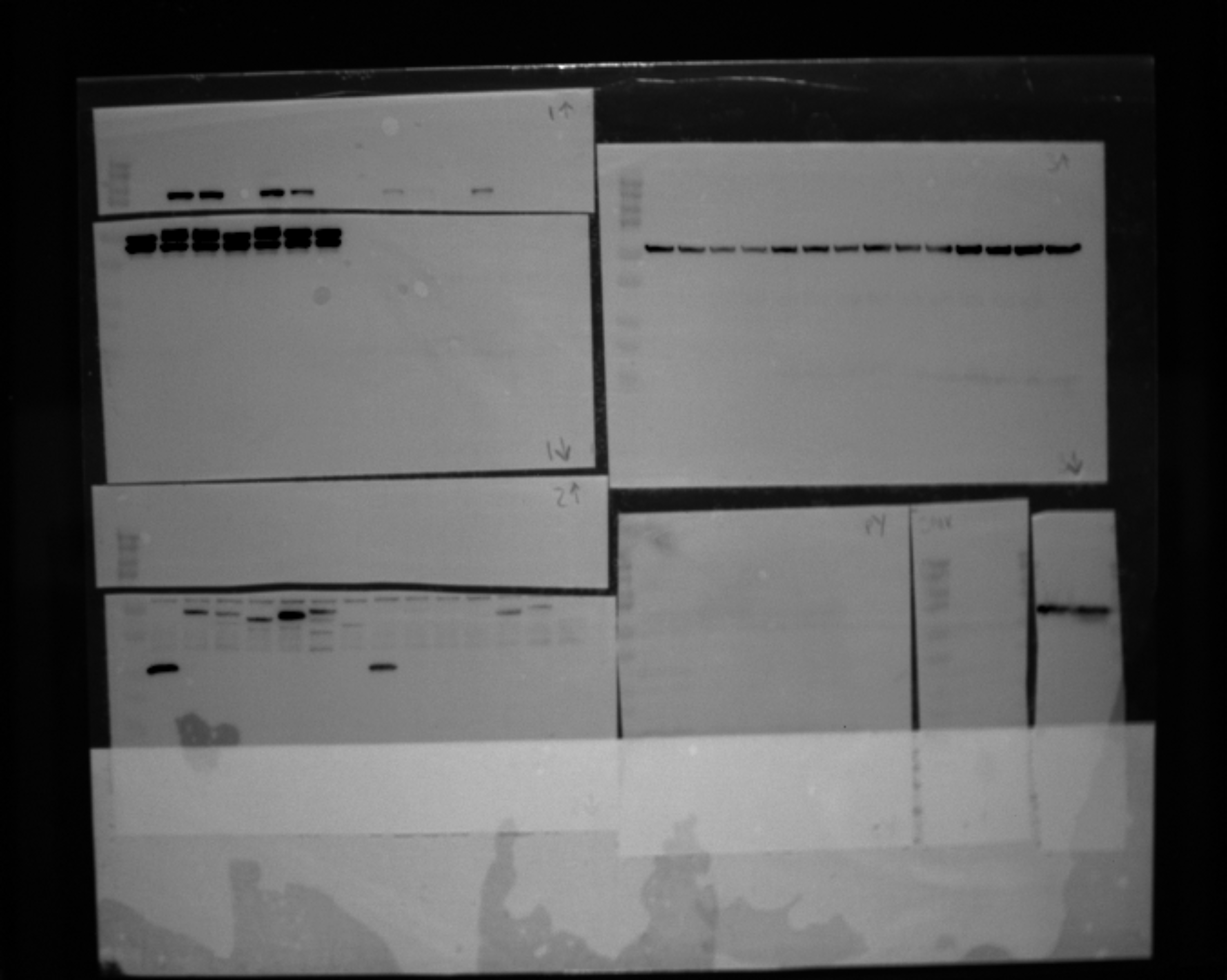

Supplement: Supplementary file 3 — Source data Fig. 1 [file 44319_2025_472_MOESM3_ESM.zip › Figure 1/1C/Ladder+GSK3+GFP_Overlay/LadderGSK3GFP_Overlay_composite.tif]

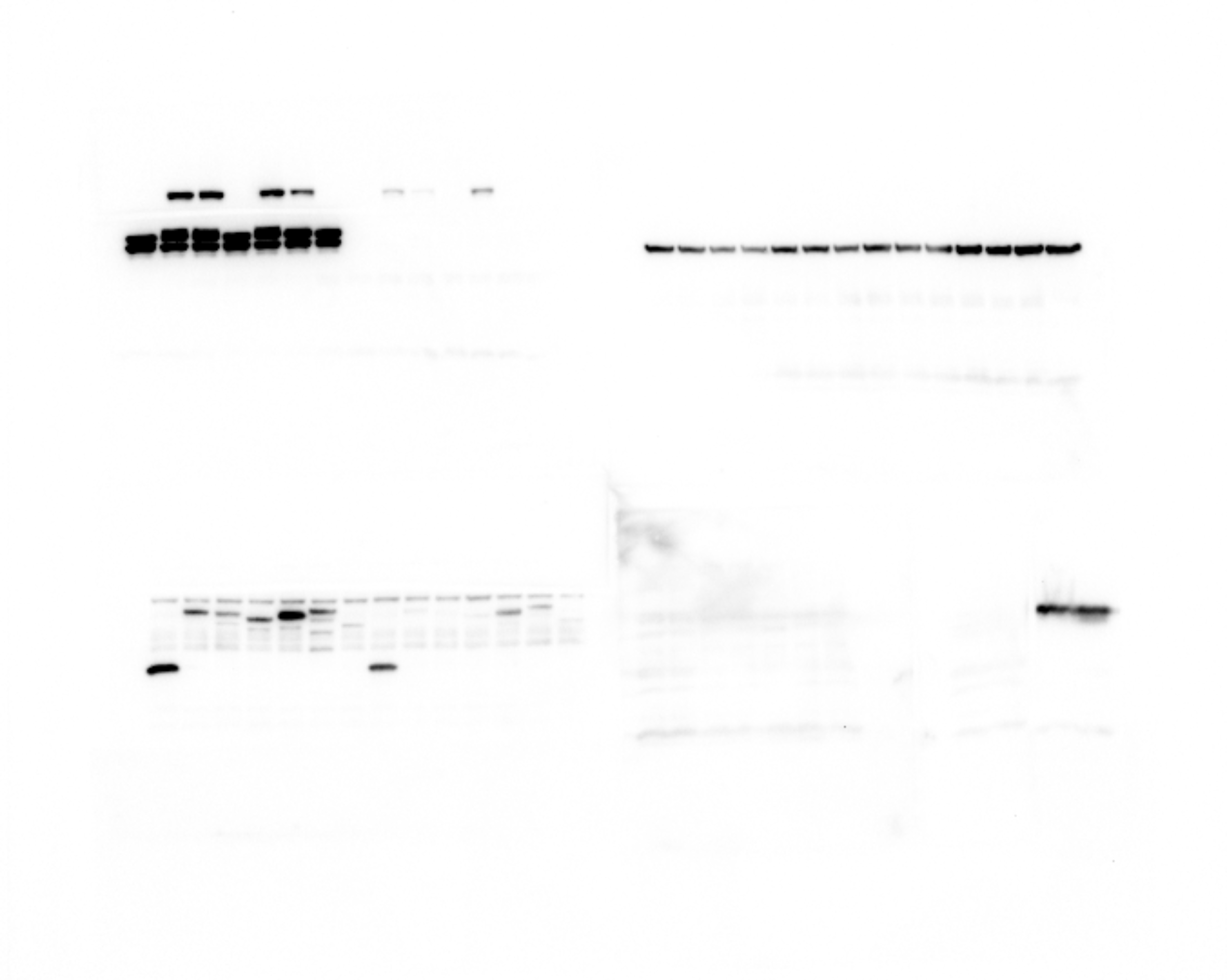

Supplement: Supplementary file 3 — Source data Fig. 1 [file 44319_2025_472_MOESM3_ESM.zip › Figure 1/1C/Ladder+GSK3+GFP_Overlay/LadderGSK3GFP_OverlayChemi.tif]

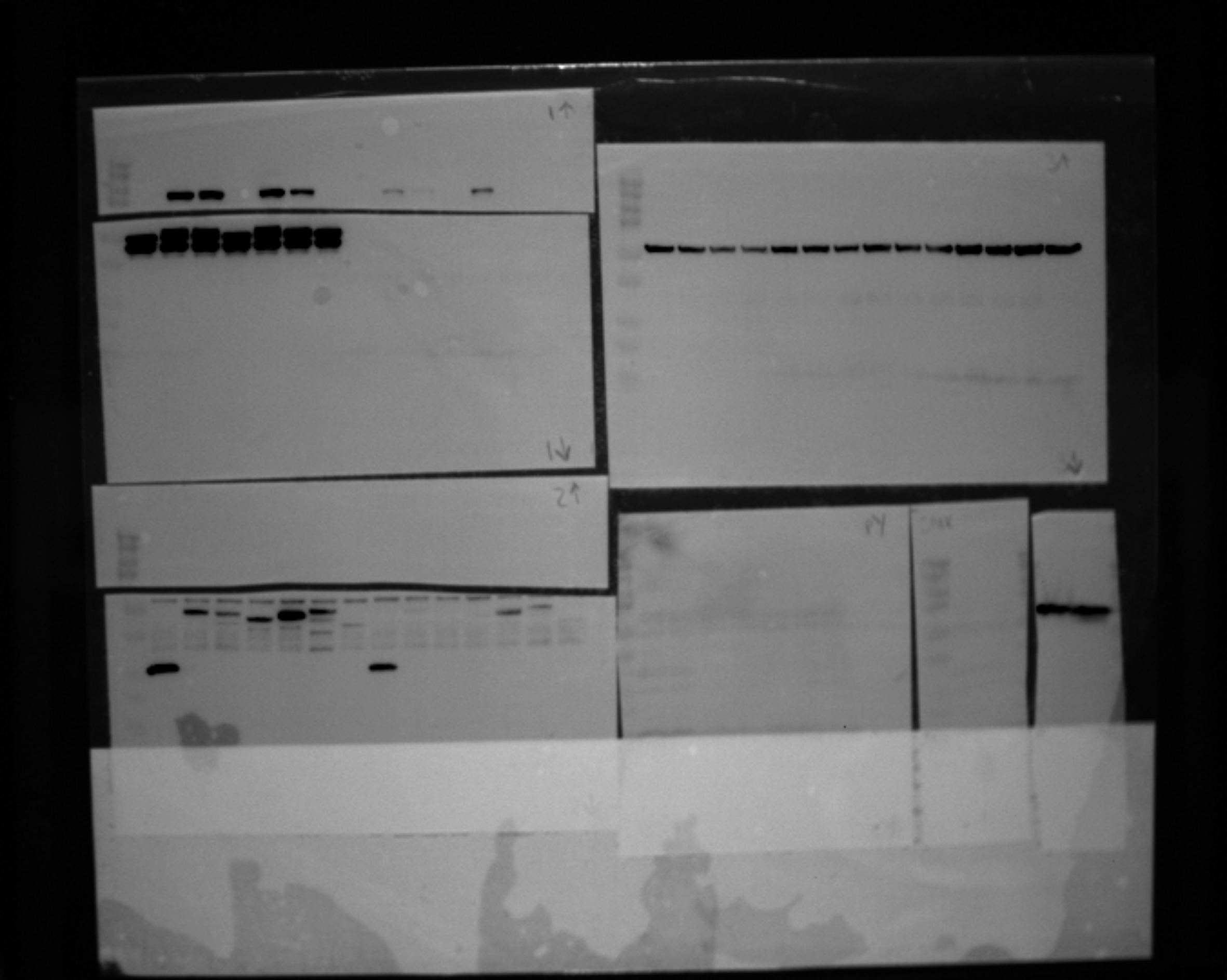

Supplement: Supplementary file 3 — Source data Fig. 1 [file 44319_2025_472_MOESM3_ESM.zip › Figure 1/1C/Ladder+STAT3+GSK3+GFP/LadderSTAT3GSK3GFP_composite.tif]

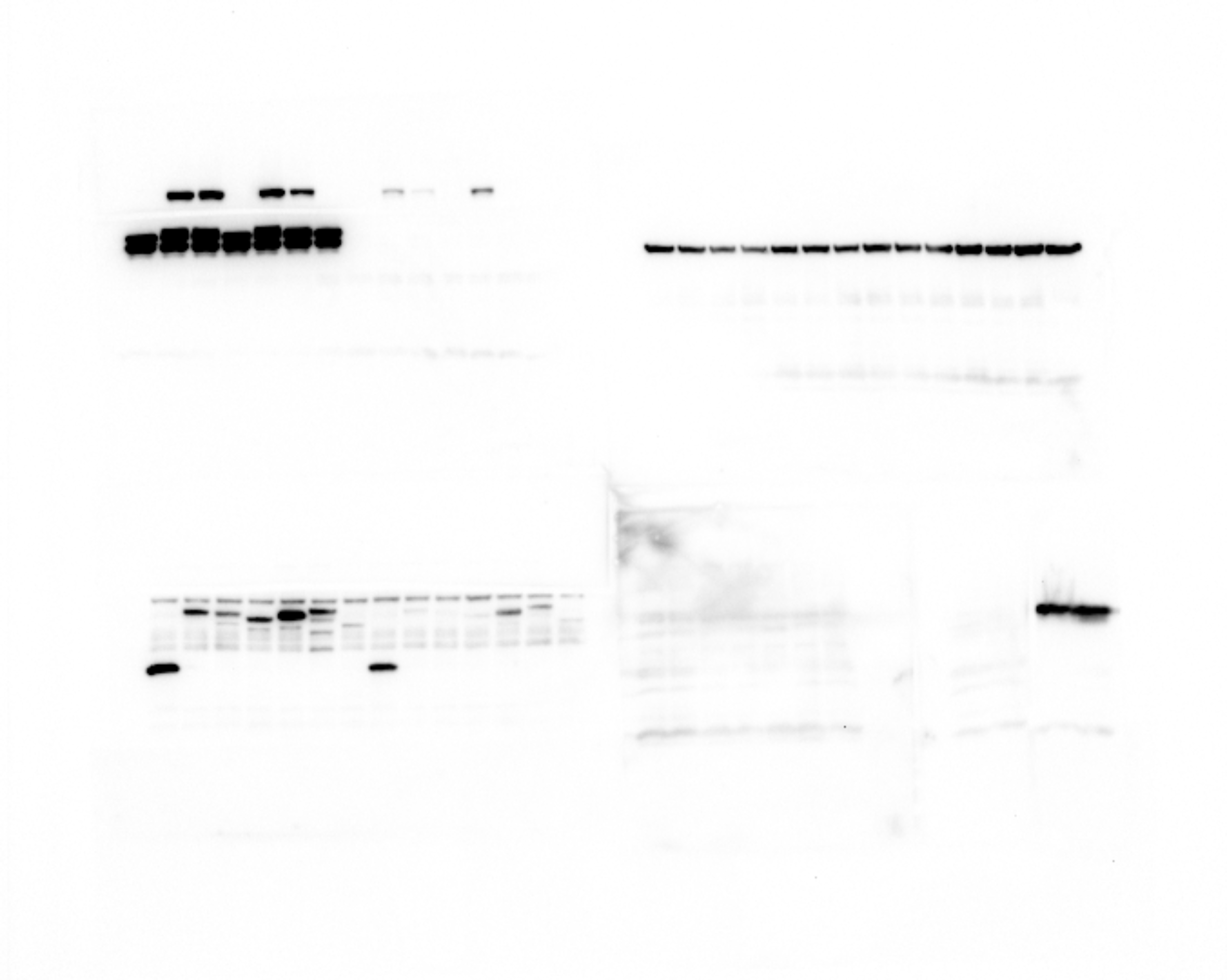

Supplement: Supplementary file 3 — Source data Fig. 1 [file 44319_2025_472_MOESM3_ESM.zip › Figure 1/1C/Ladder+STAT3+GSK3+GFP/LadderSTAT3GSK3GFPChemi.tif]

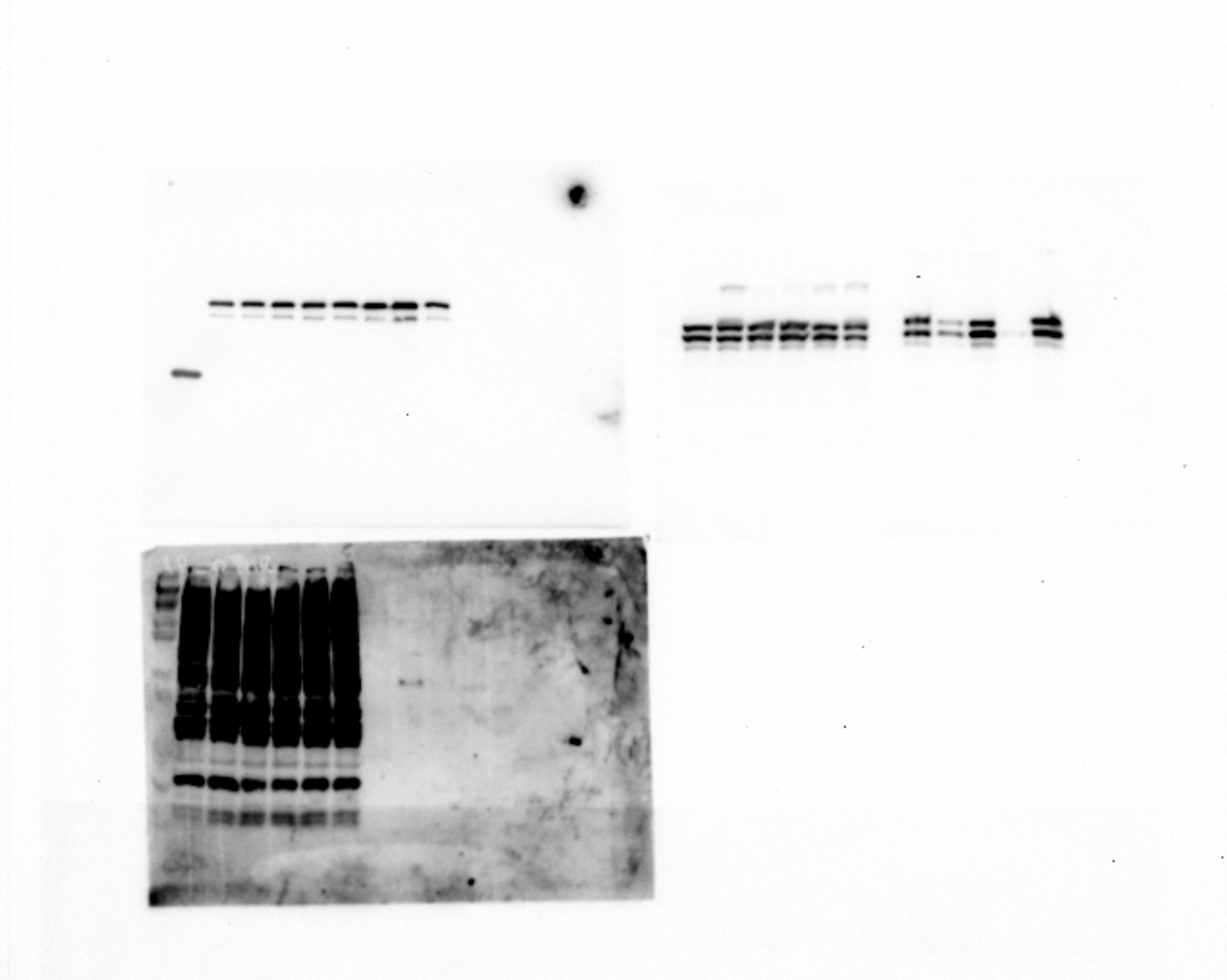

Supplement: Supplementary file 4 — Source data Fig. 2 [file 44319_2025_472_MOESM4_ESM.zip › Figure 2/2F/Ladder+GFPtop/LadderGFPtopChemi.tif]

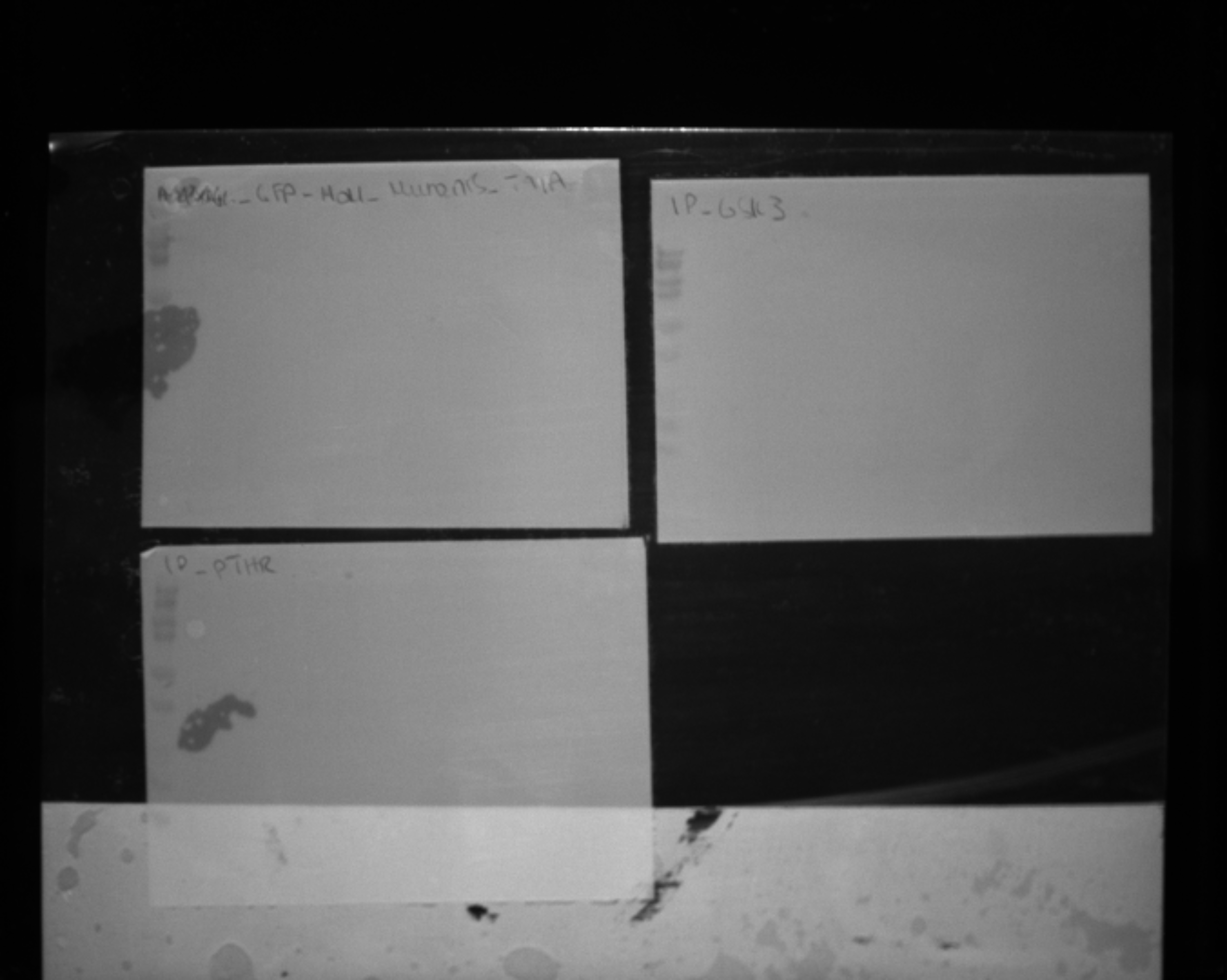

Supplement: Supplementary file 4 — Source data Fig. 2 [file 44319_2025_472_MOESM4_ESM.zip › Figure 2/2F/Ladder+GFPtop/LadderGFPtopMembrane.tif]

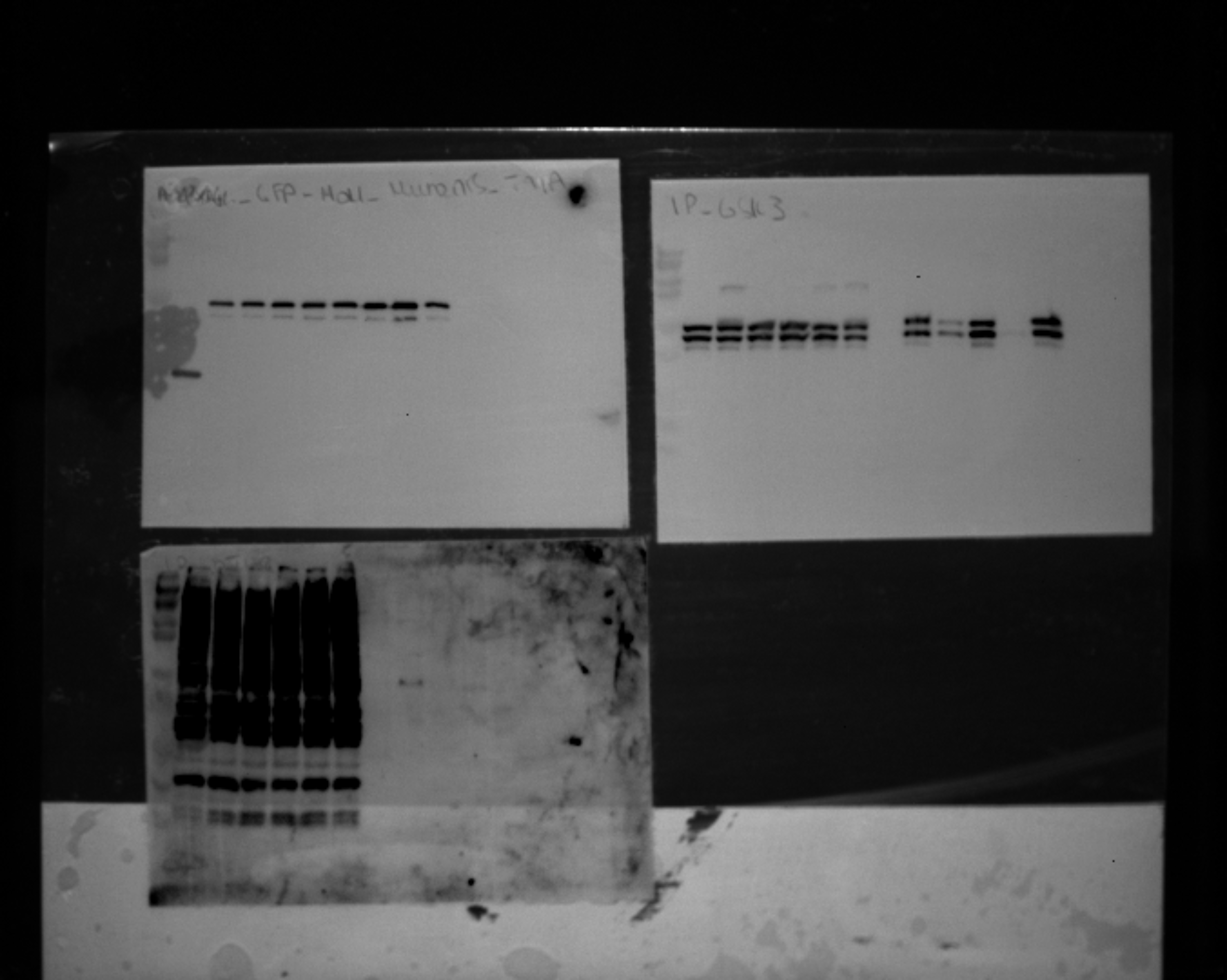

Supplement: Supplementary file 4 — Source data Fig. 2 [file 44319_2025_472_MOESM4_ESM.zip › Figure 2/2F/Ladder+GFPtop/LadderGFPtop_composite.tif]

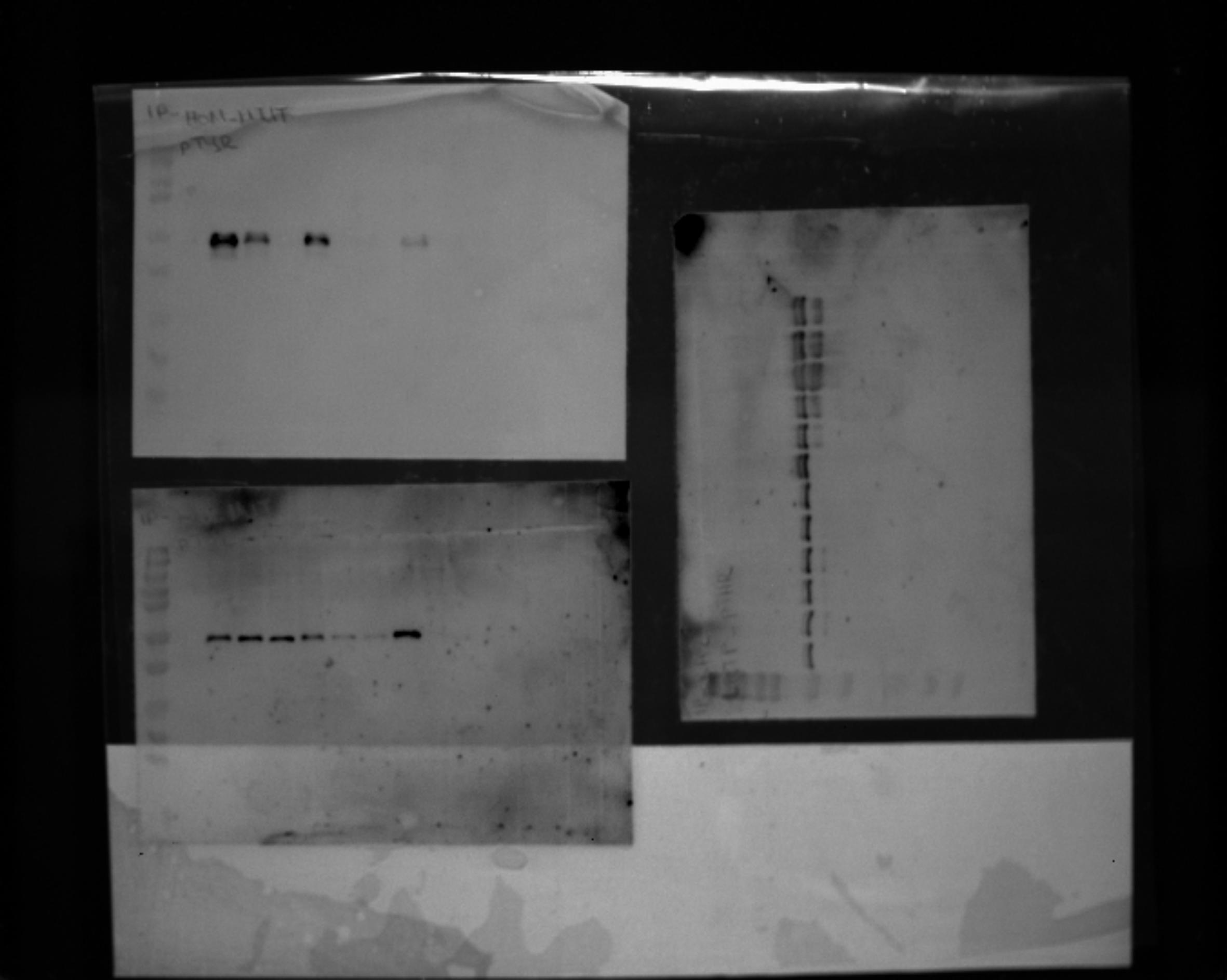

Supplement: Supplementary file 4 — Source data Fig. 2 [file 44319_2025_472_MOESM4_ESM.zip › Figure 2/2F/Ladder+pTyrtop+pThrbottom/LadderpTyrtoppThrbottom_composite.tif]

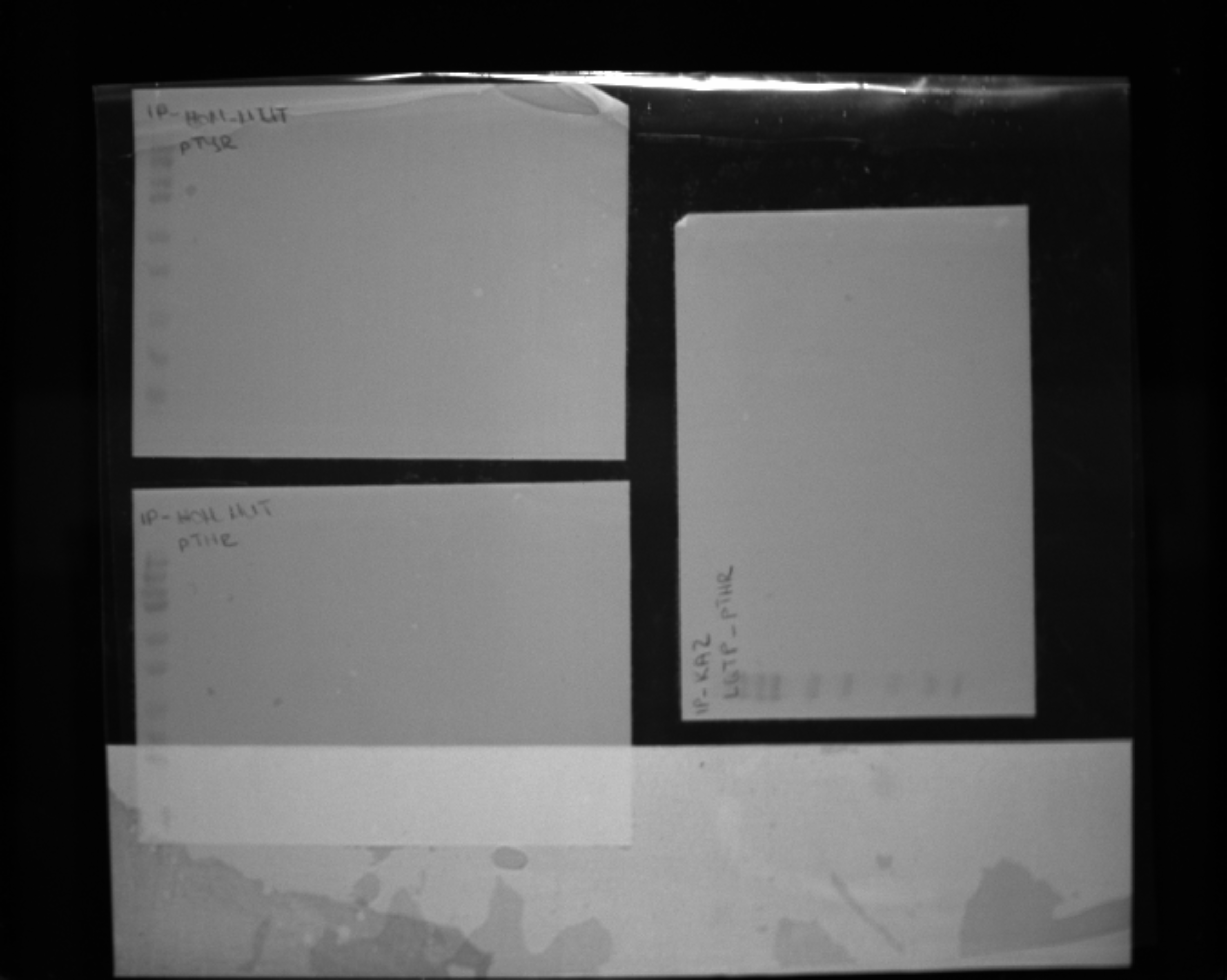

Supplement: Supplementary file 4 — Source data Fig. 2 [file 44319_2025_472_MOESM4_ESM.zip › Figure 2/2F/Ladder+pTyrtop+pThrbottom/LadderpTyrtoppThrbottomMembrane.tif]

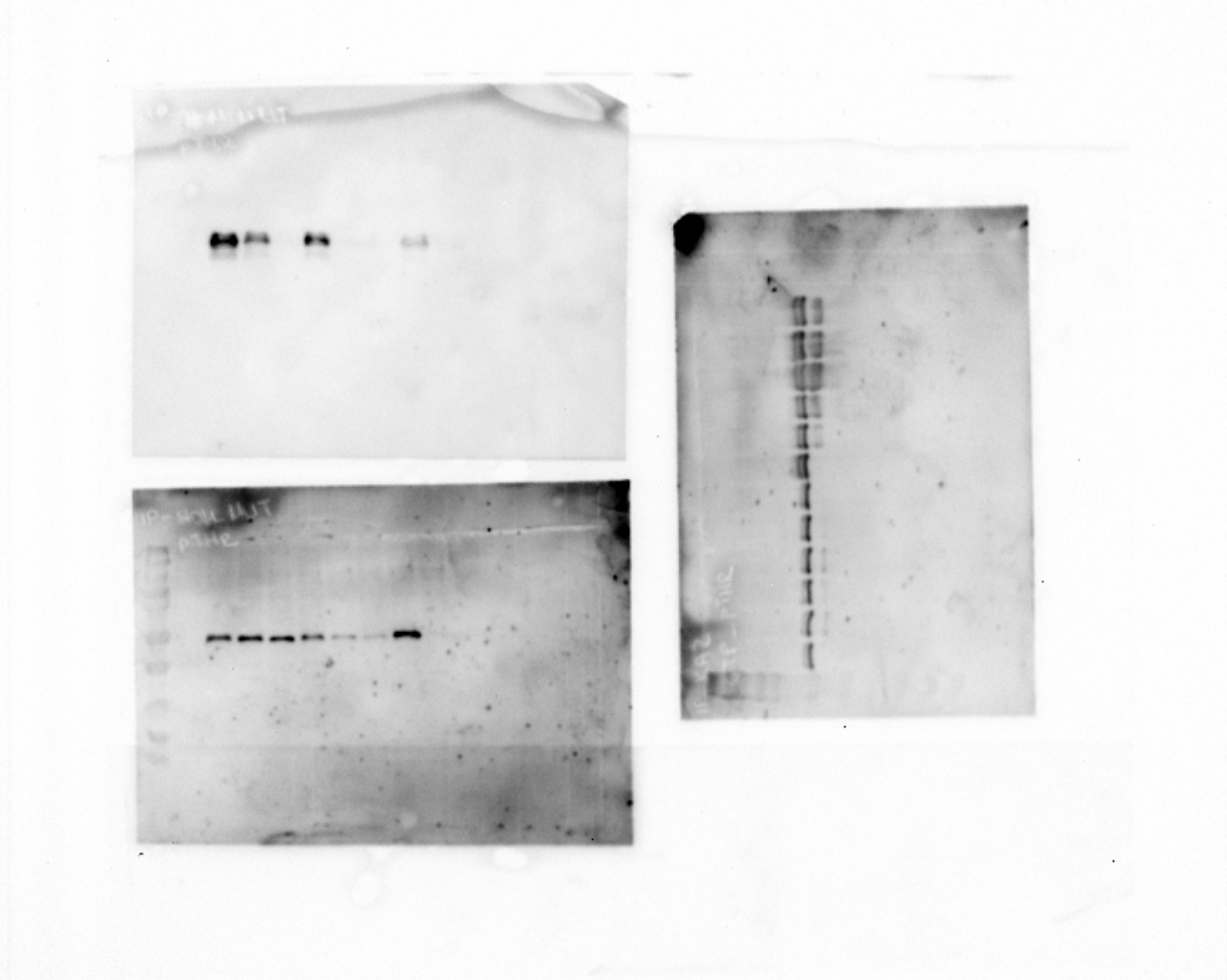

Supplement: Supplementary file 4 — Source data Fig. 2 [file 44319_2025_472_MOESM4_ESM.zip › Figure 2/2F/Ladder+pTyrtop+pThrbottom/LadderpTyrtoppThrbottomChemi.tif]

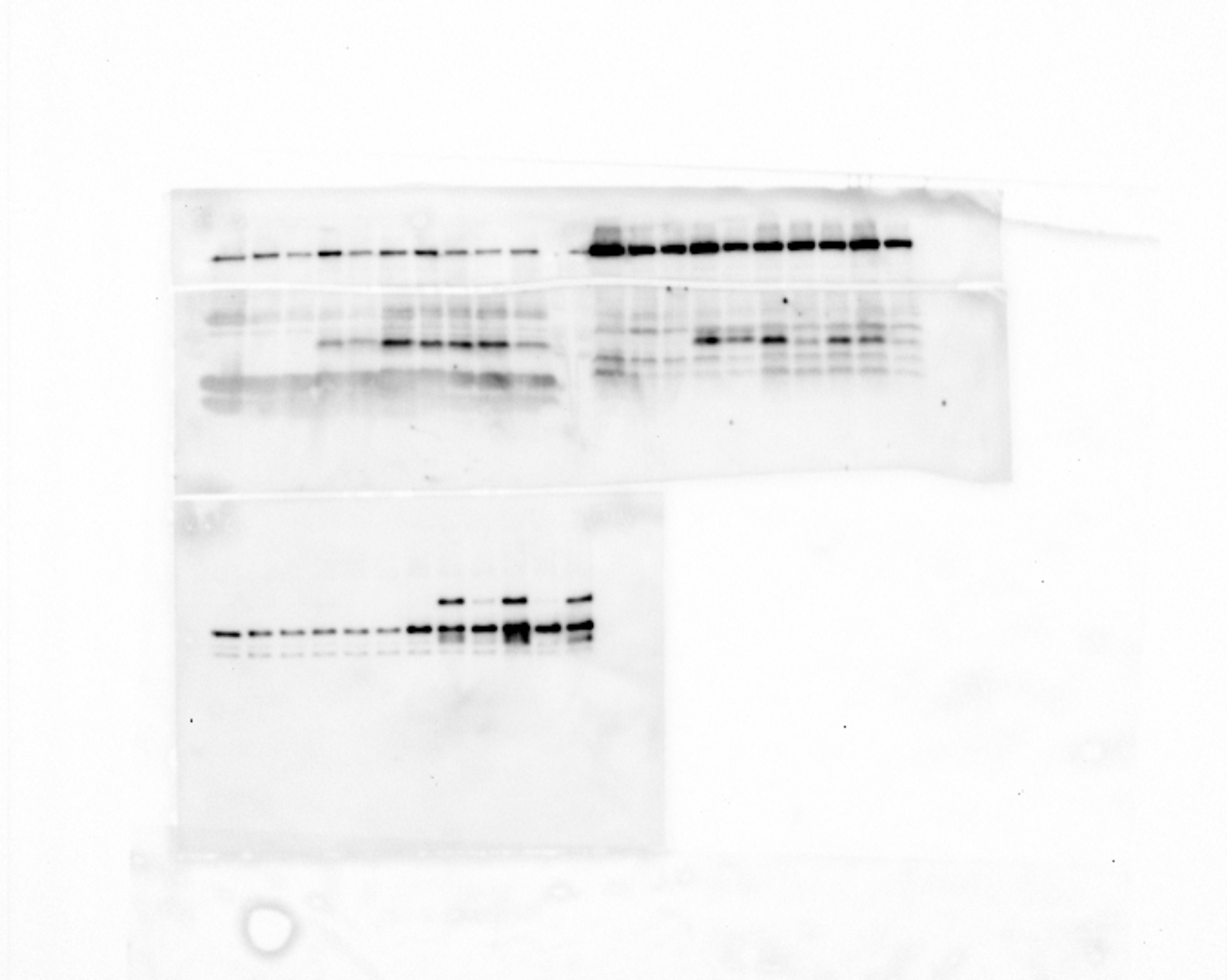

Supplement: Supplementary file 4 — Source data Fig. 2 [file 44319_2025_472_MOESM4_ESM.zip › Figure 2/2C/Ladder+STAT3/LadderSTAT3_V2Chemi.tif]

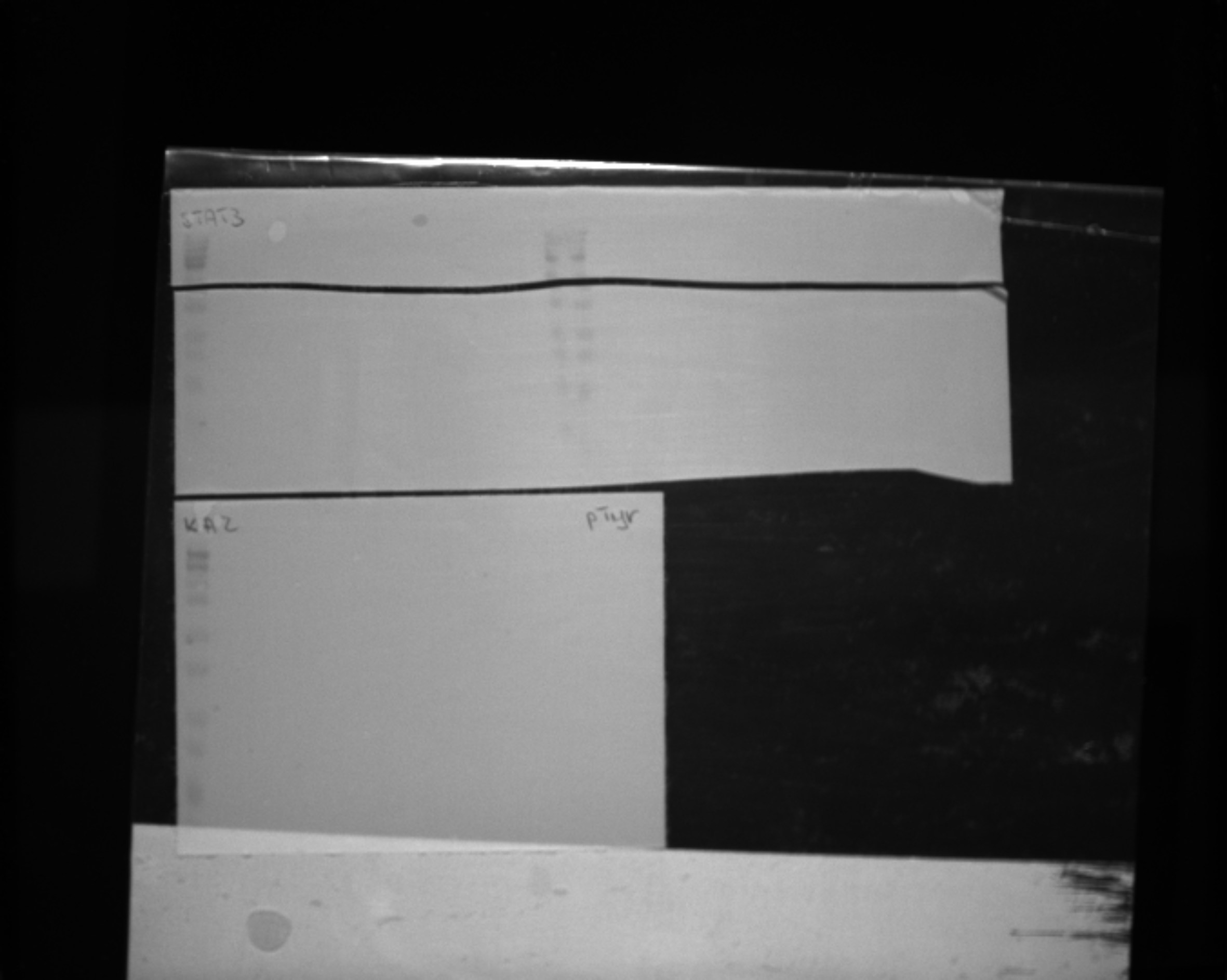

Supplement: Supplementary file 4 — Source data Fig. 2 [file 44319_2025_472_MOESM4_ESM.zip › Figure 2/2C/Ladder+STAT3/LadderSTAT3_V2Membrane.tif]

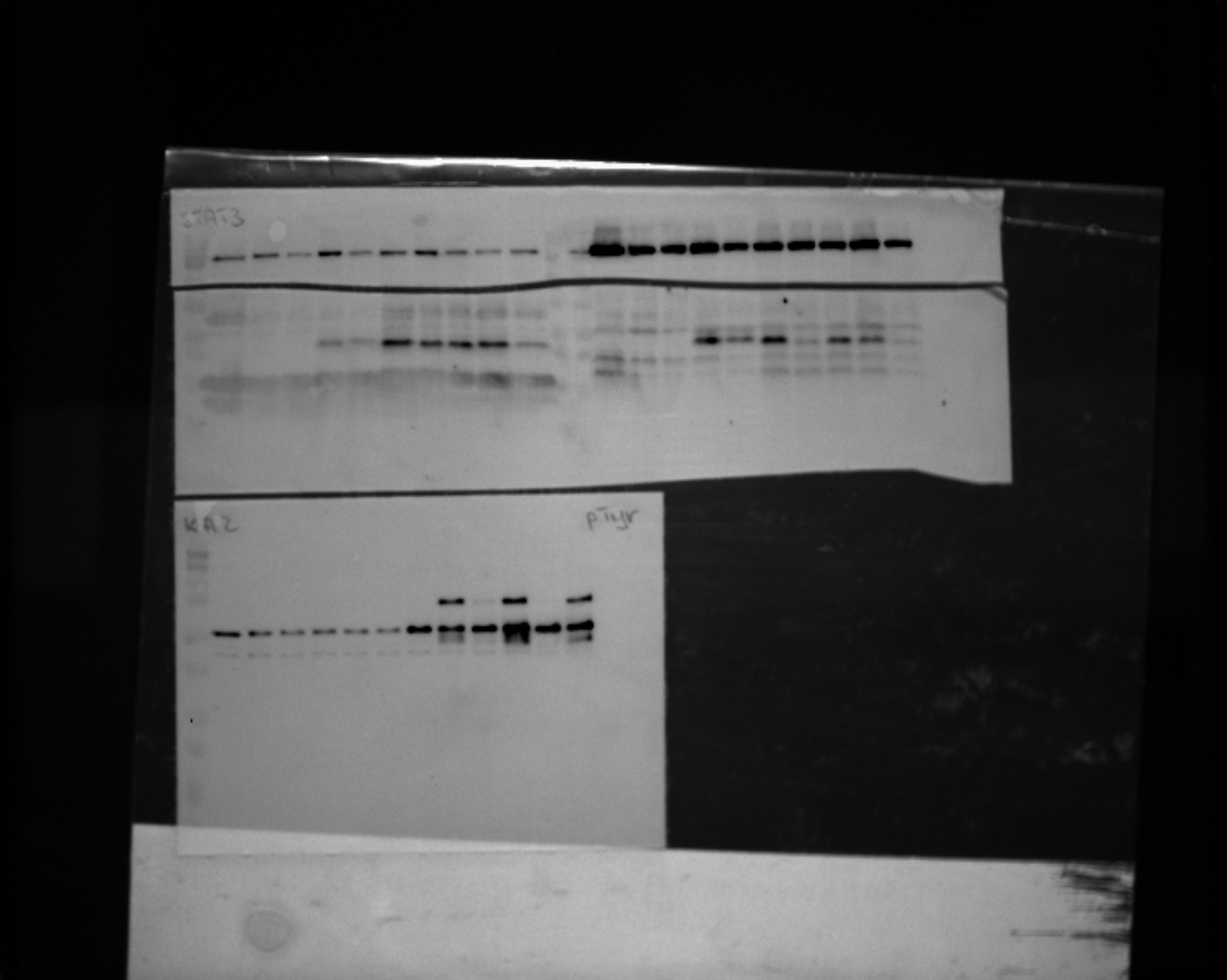

Supplement: Supplementary file 4 — Source data Fig. 2 [file 44319_2025_472_MOESM4_ESM.zip › Figure 2/2C/Ladder+STAT3/LadderSTAT3_V2_composite.tif]

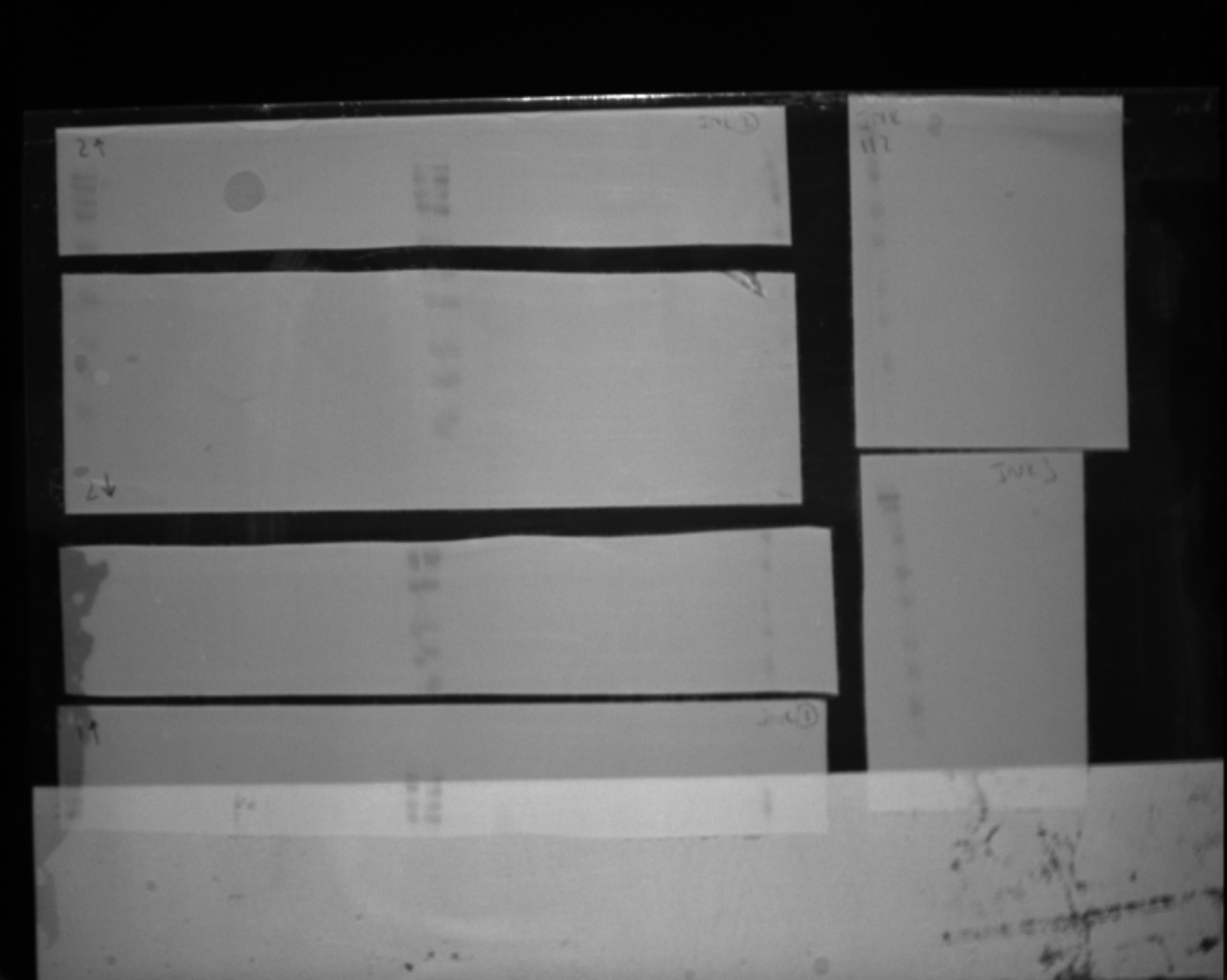

Supplement: Supplementary file 4 — Source data Fig. 2 [file 44319_2025_472_MOESM4_ESM.zip › Figure 2/2C/Ladder+pY705-STAT3/LadderpY705-STAT3Membrane.tif]

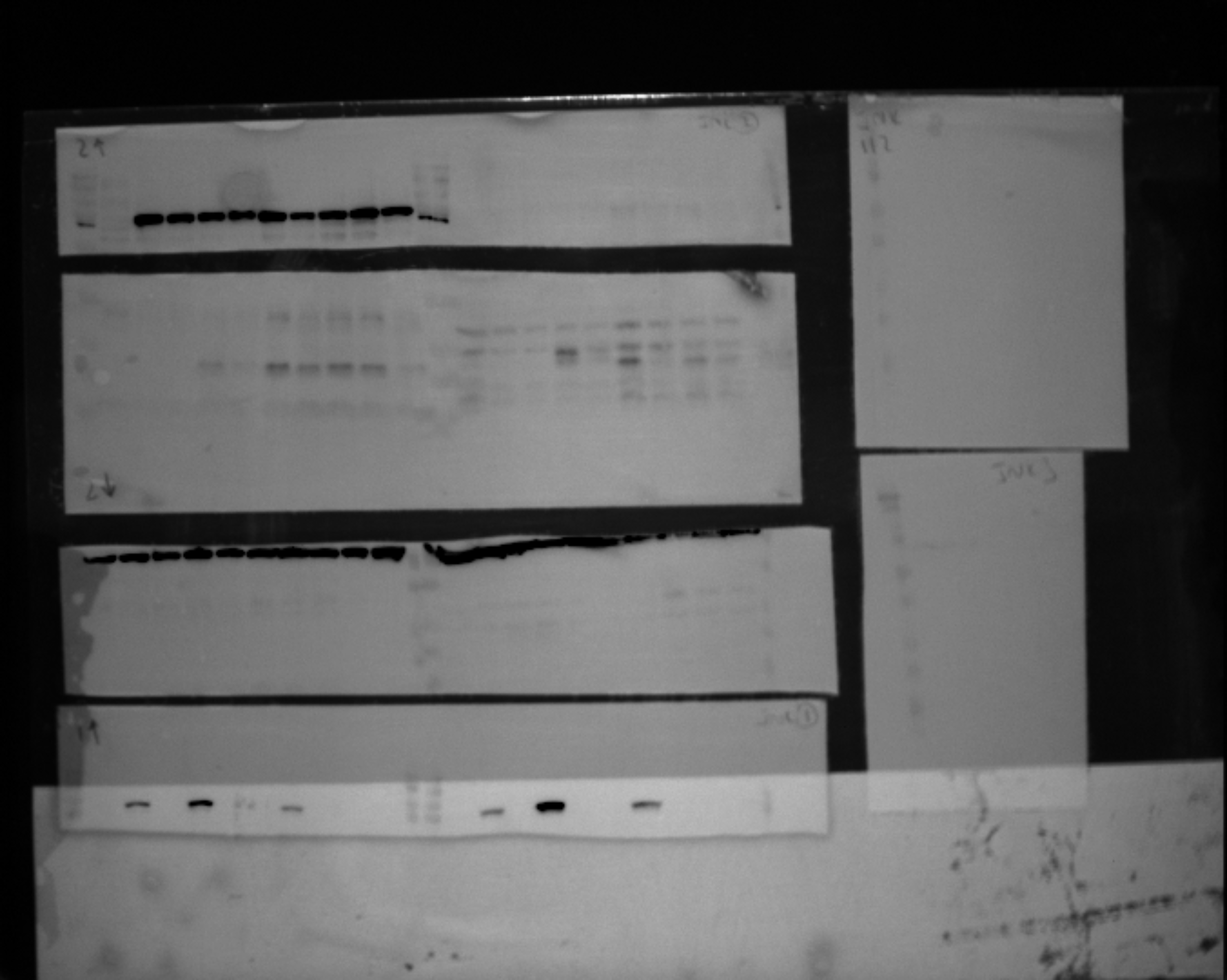

Supplement: Supplementary file 4 — Source data Fig. 2 [file 44319_2025_472_MOESM4_ESM.zip › Figure 2/2C/Ladder+pY705-STAT3/LadderpY705-STAT3_composite.tif]

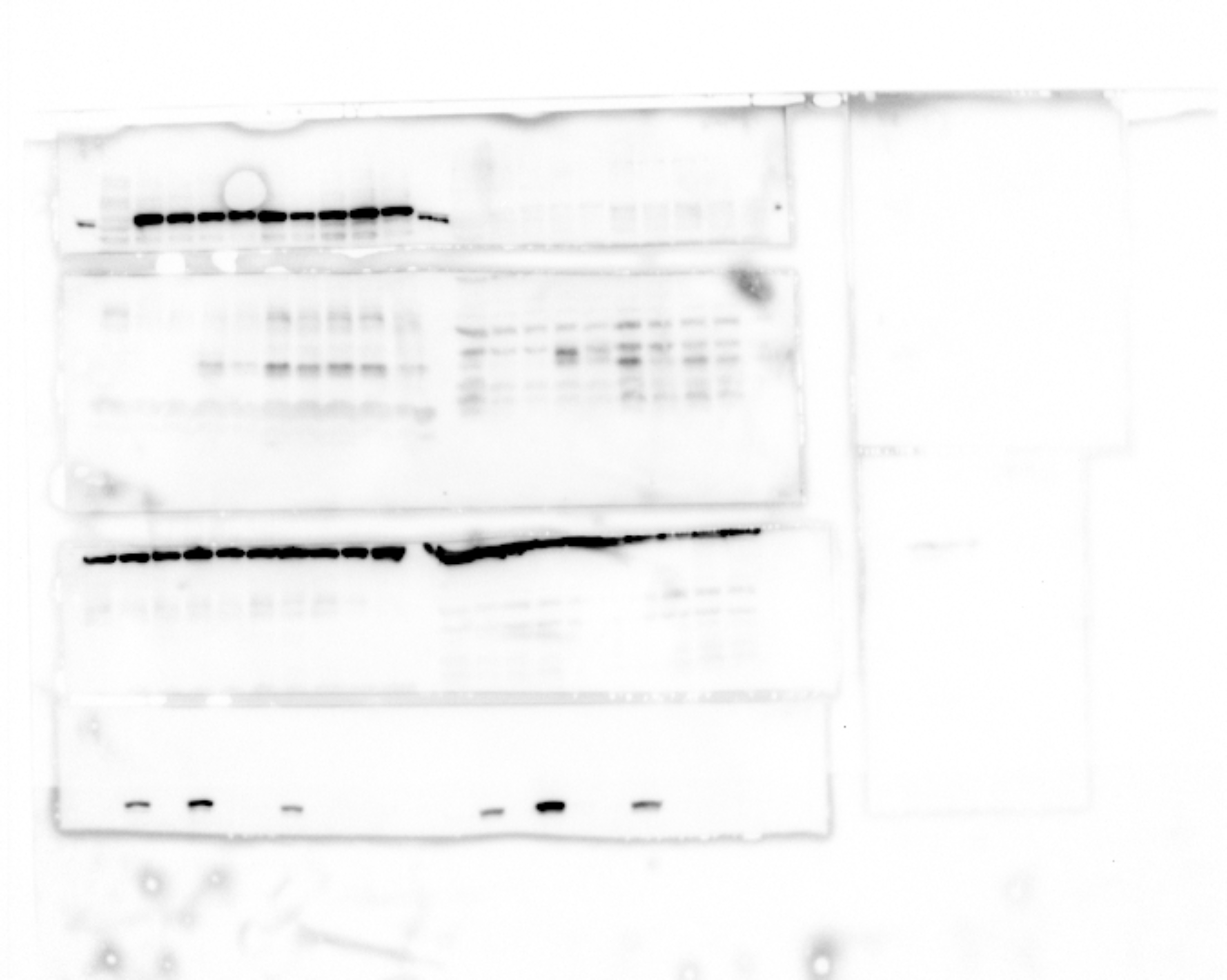

Supplement: Supplementary file 4 — Source data Fig. 2 [file 44319_2025_472_MOESM4_ESM.zip › Figure 2/2C/Ladder+pY705-STAT3/LadderpY705-STAT3Chemi.tif]

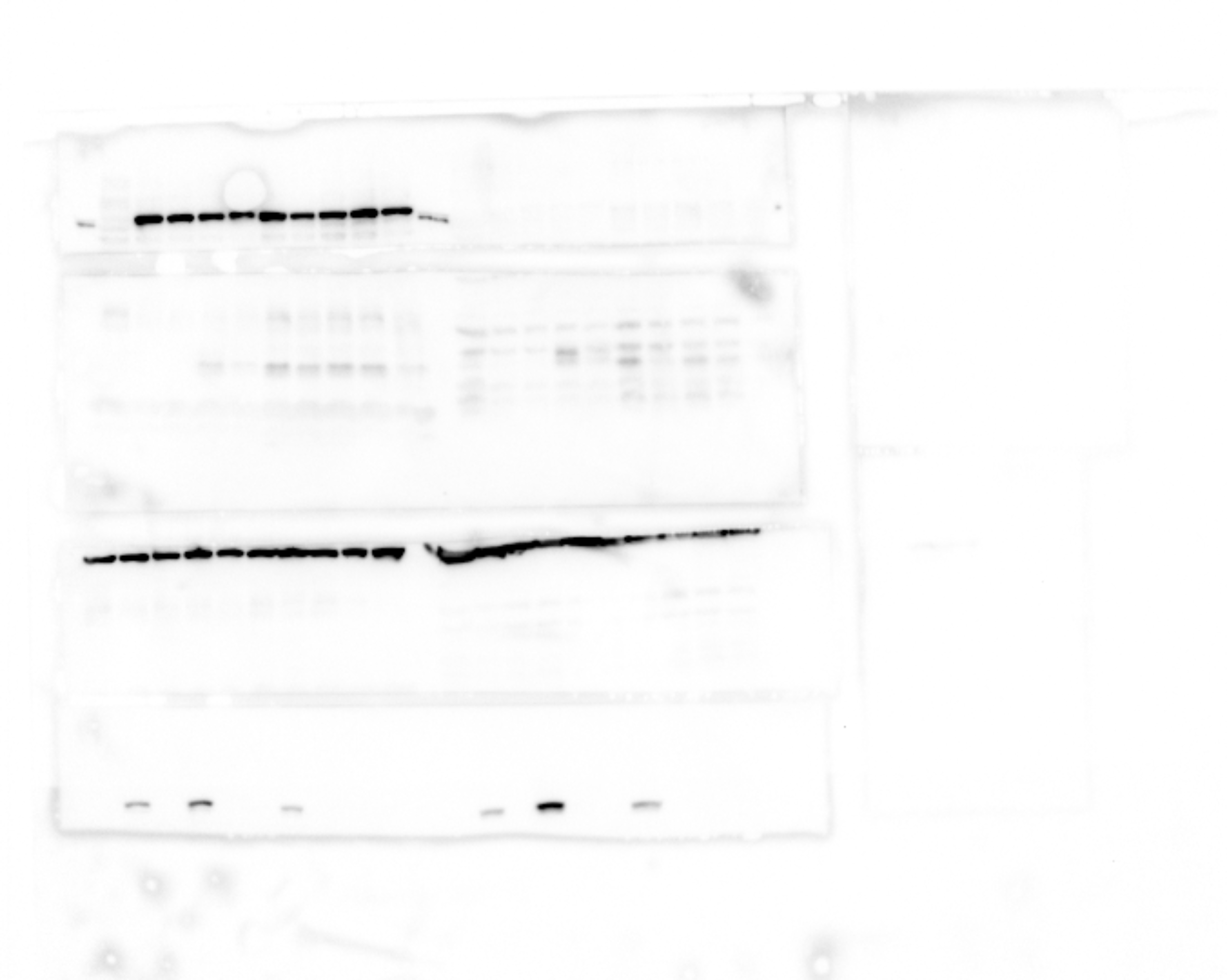

Supplement: Supplementary file 4 — Source data Fig. 2 [file 44319_2025_472_MOESM4_ESM.zip › Figure 2/2C/Ladder+DnaK/LadderDnaKChemi.tif]

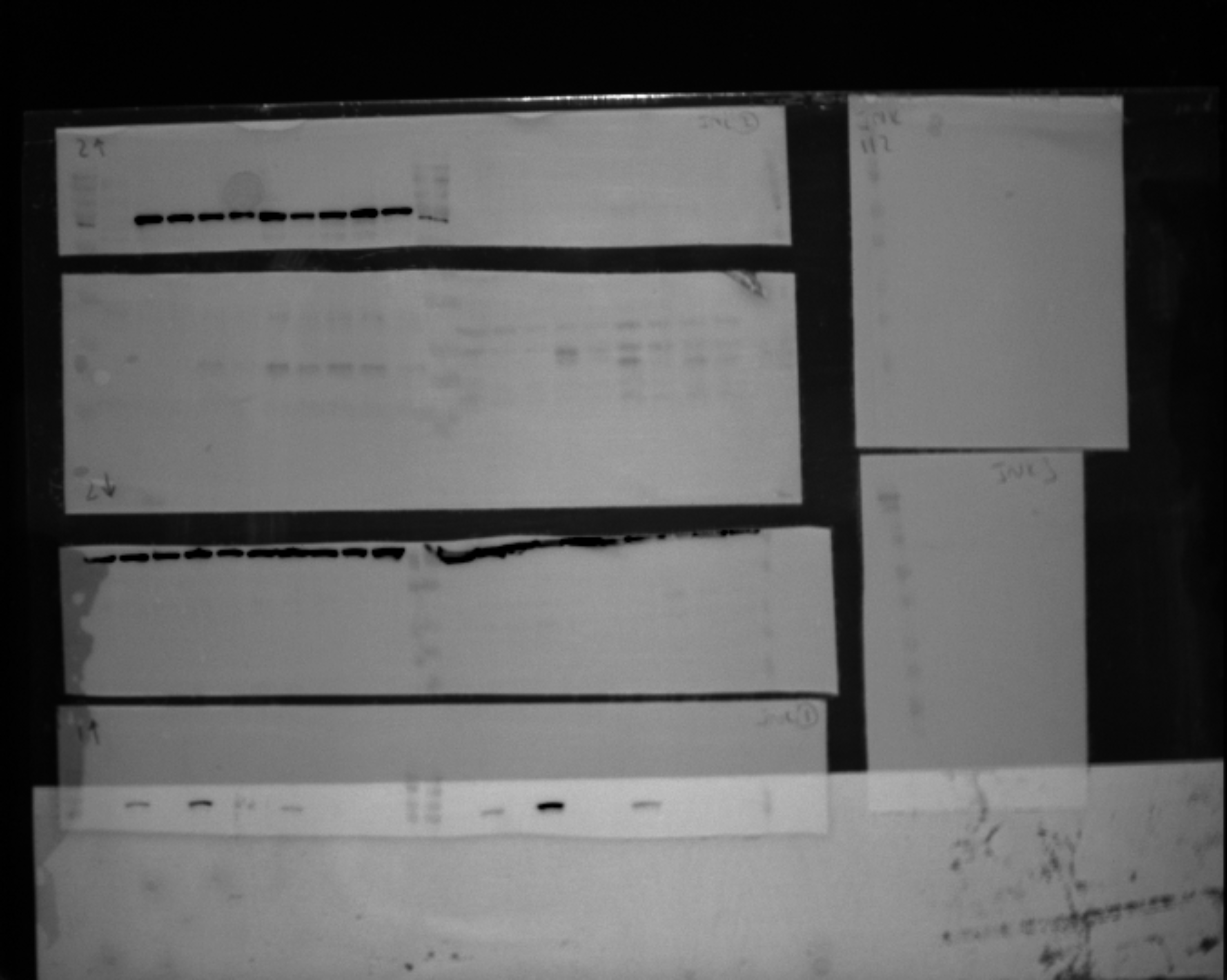

Supplement: Supplementary file 4 — Source data Fig. 2 [file 44319_2025_472_MOESM4_ESM.zip › Figure 2/2C/Ladder+DnaK/LadderDnaK_composite.tif]

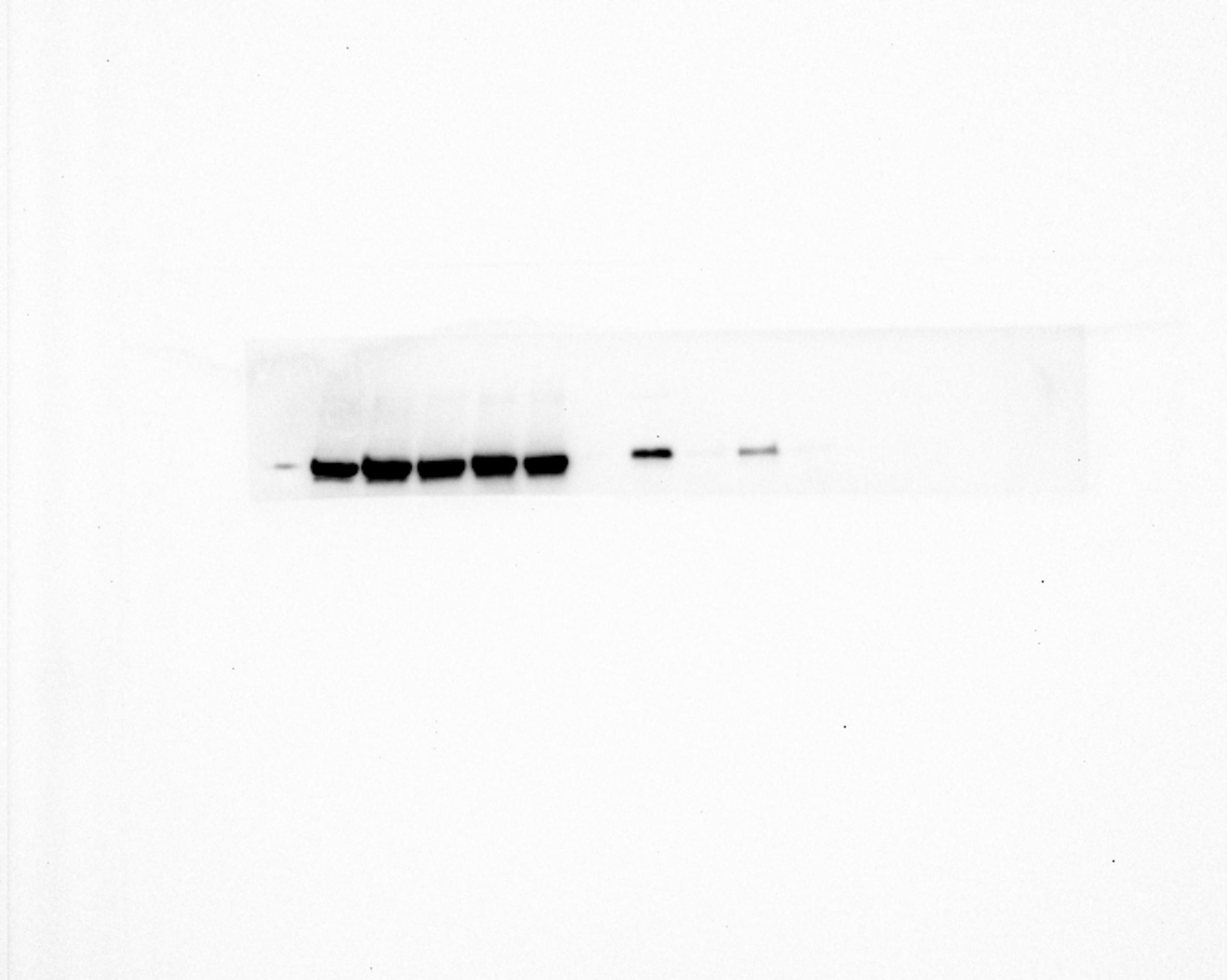

Supplement: Supplementary file 4 — Source data Fig. 2 [file 44319_2025_472_MOESM4_ESM.zip › Figure 2/2D/Ladder+STAT3/LadderSTAT3Chemi.tif]

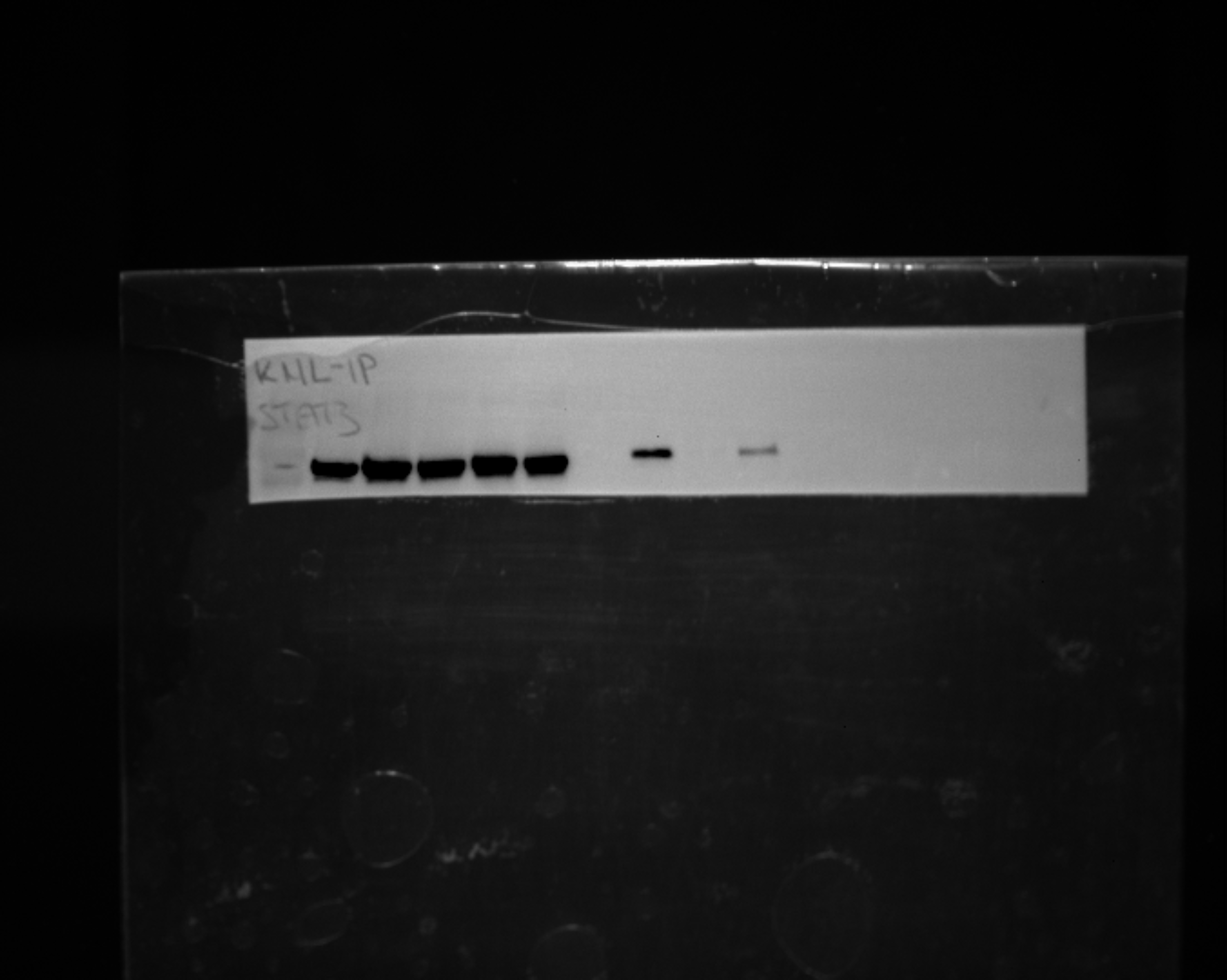

Supplement: Supplementary file 4 — Source data Fig. 2 [file 44319_2025_472_MOESM4_ESM.zip › Figure 2/2D/Ladder+STAT3/LadderSTAT3_composite.tif]

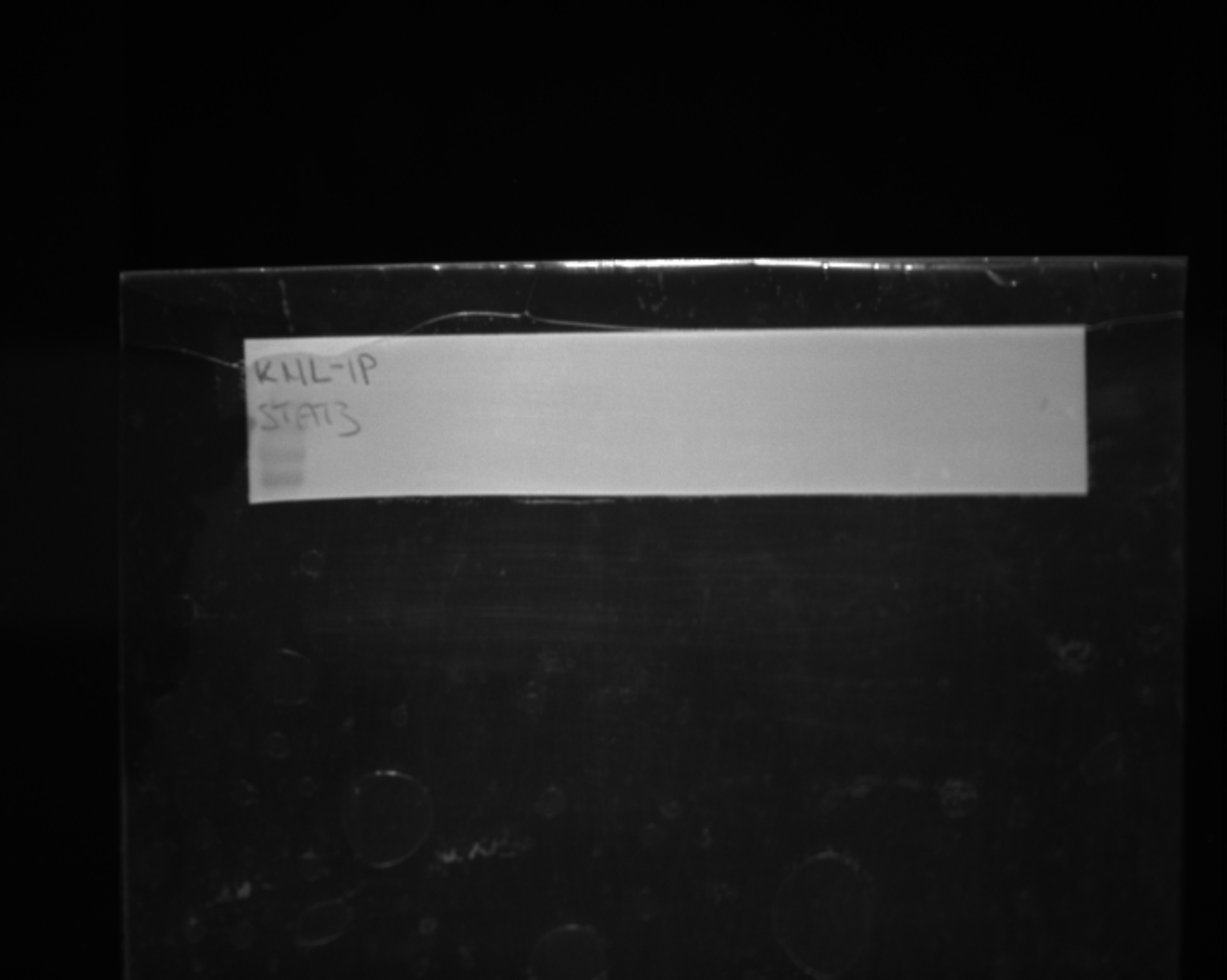

Supplement: Supplementary file 4 — Source data Fig. 2 [file 44319_2025_472_MOESM4_ESM.zip › Figure 2/2D/Ladder+STAT3/LadderSTAT3Membrane.tif]

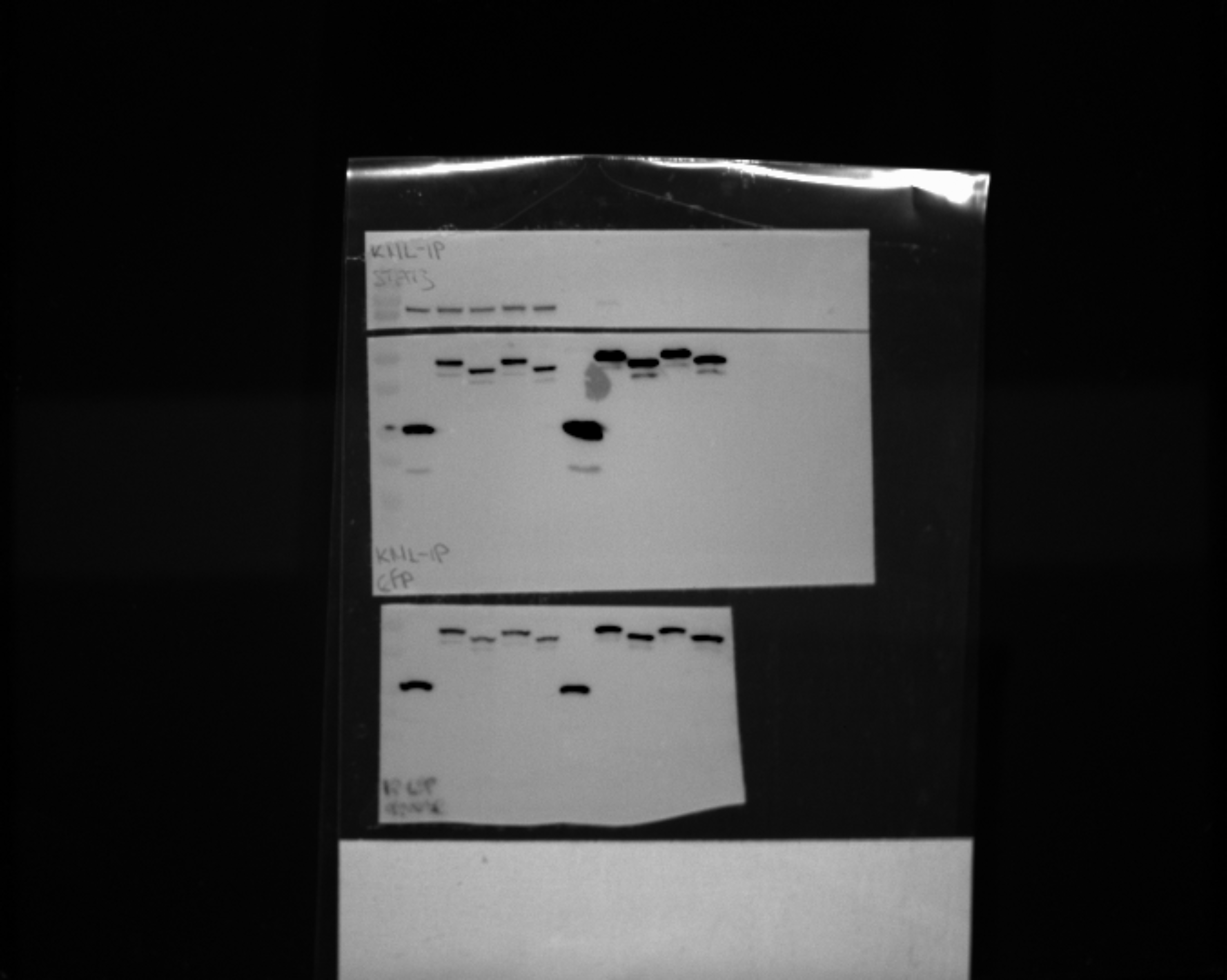

Supplement: Supplementary file 4 — Source data Fig. 2 [file 44319_2025_472_MOESM4_ESM.zip › Figure 2/2D/Ladder+GFP_lower_v2/LadderGFP_lower_v2_composite.tif]

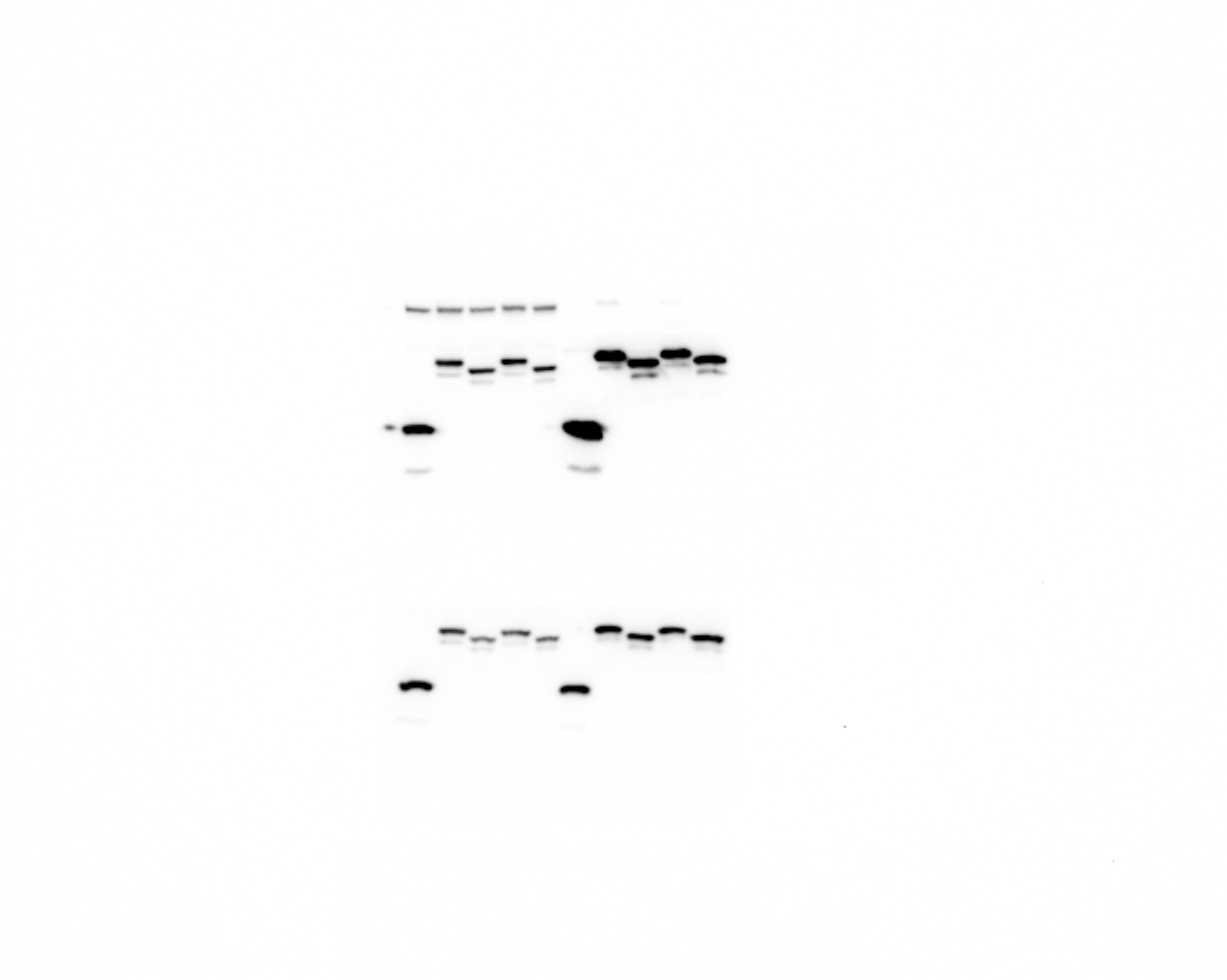

Supplement: Supplementary file 4 — Source data Fig. 2 [file 44319_2025_472_MOESM4_ESM.zip › Figure 2/2D/Ladder+GFP_lower_v2/LadderGFP_lower_v2Chemi.tif]

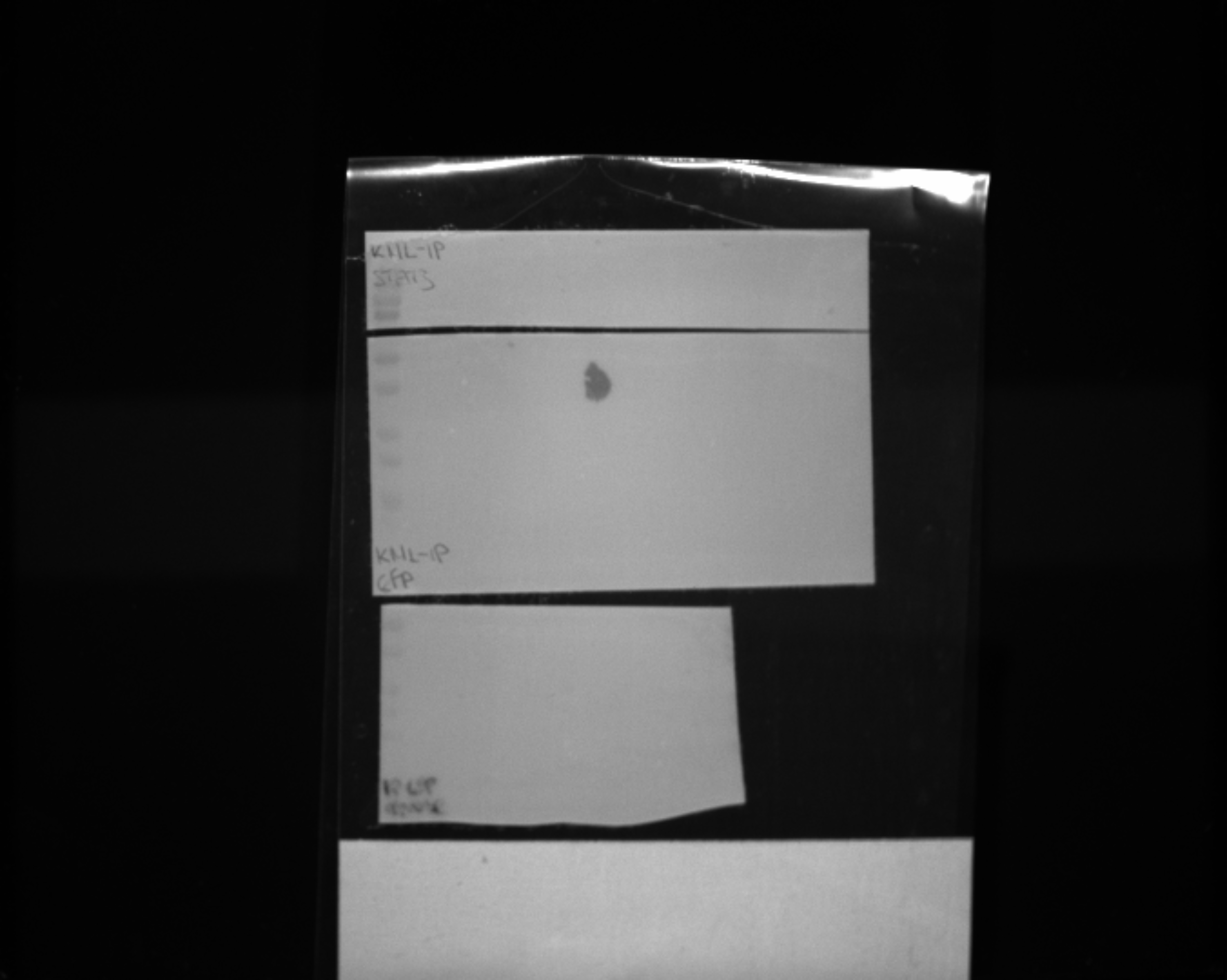

Supplement: Supplementary file 4 — Source data Fig. 2 [file 44319_2025_472_MOESM4_ESM.zip › Figure 2/2D/Ladder+GFP_lower_v2/LadderGFP_lower_v2Membrane.tif]

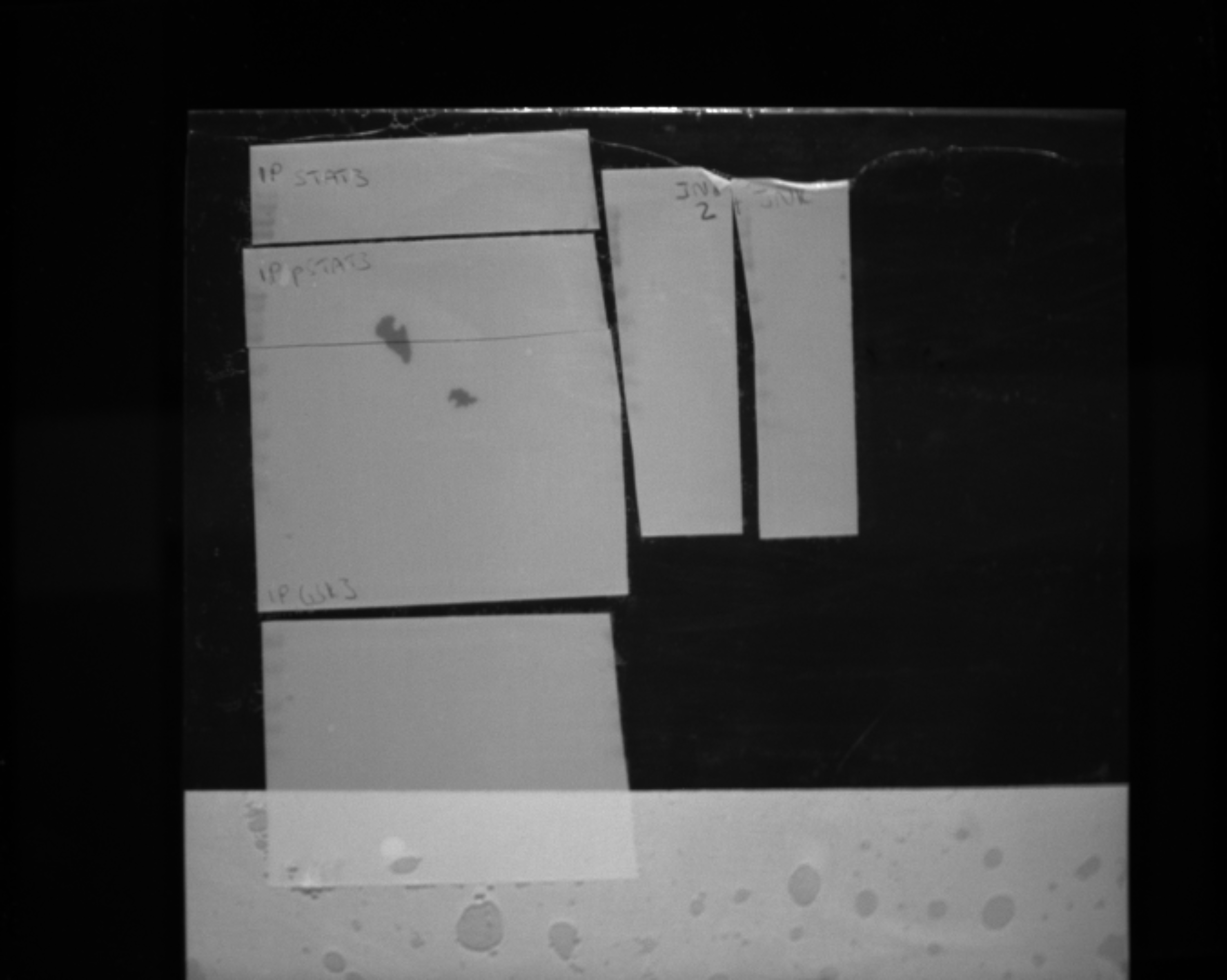

Supplement: Supplementary file 4 — Source data Fig. 2 [file 44319_2025_472_MOESM4_ESM.zip › Figure 2/2D/Ladder+GSK3/LadderGSK3Membrane.tif]

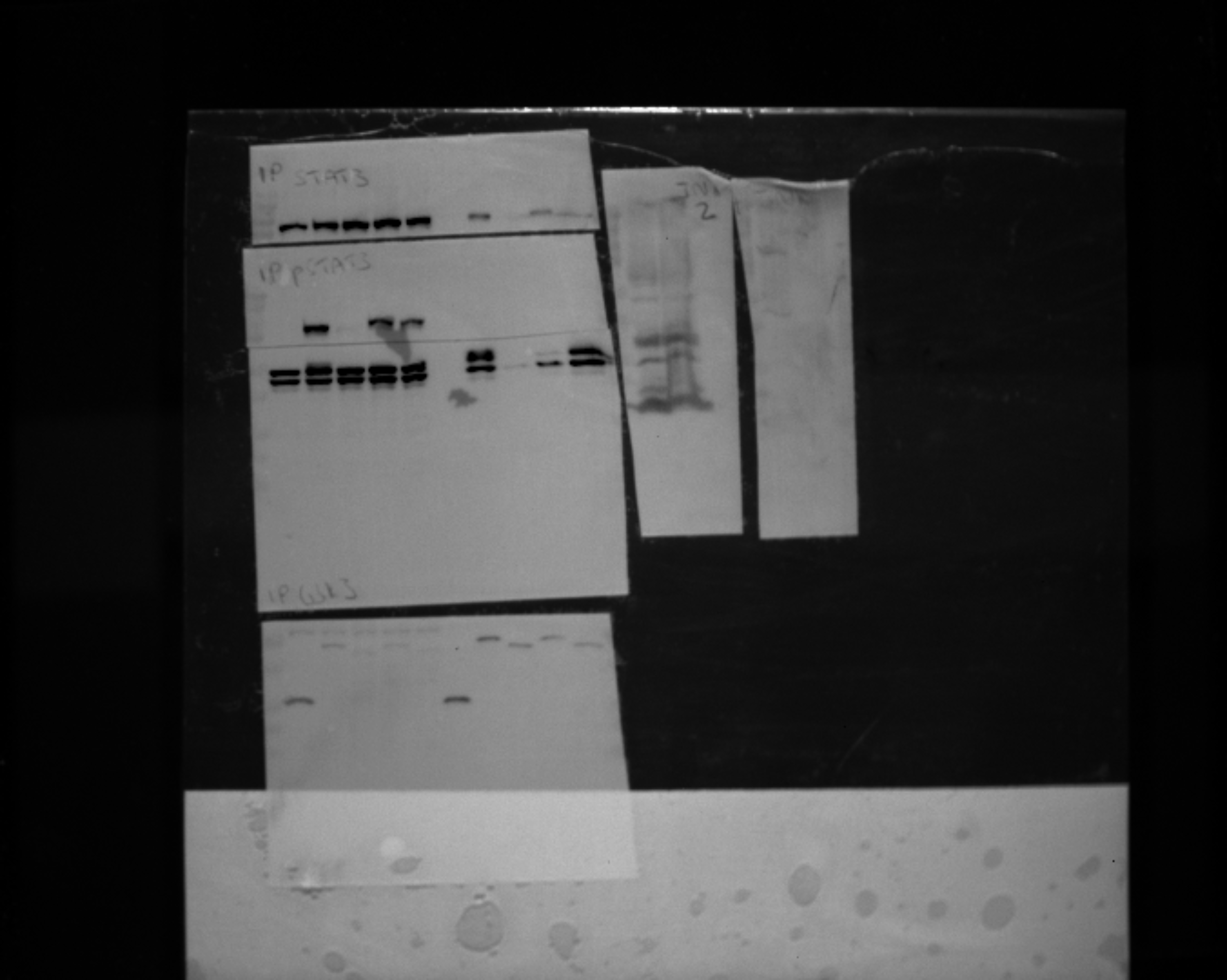

Supplement: Supplementary file 4 — Source data Fig. 2 [file 44319_2025_472_MOESM4_ESM.zip › Figure 2/2D/Ladder+GSK3/LadderGSK3_composite.tif]

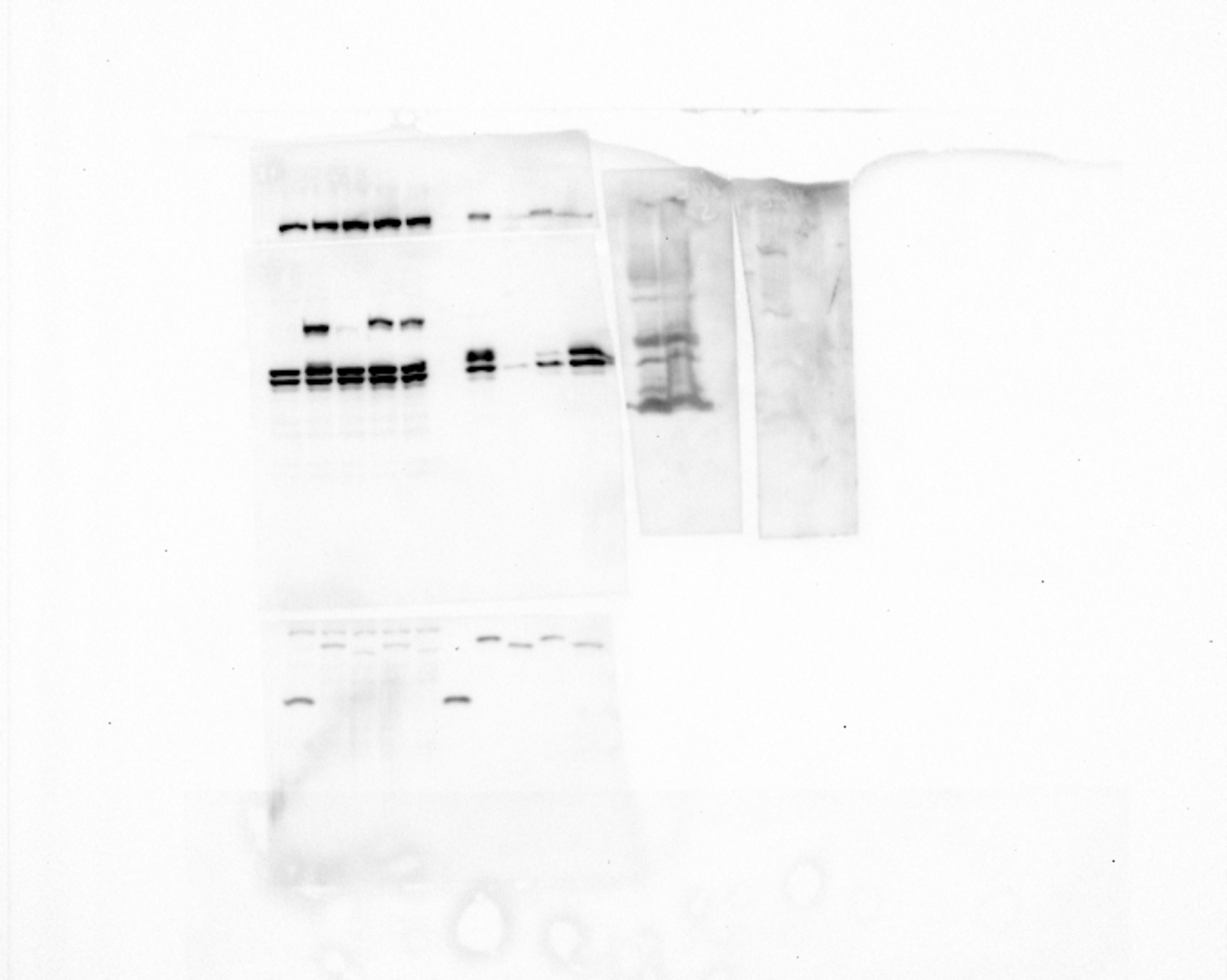

Supplement: Supplementary file 4 — Source data Fig. 2 [file 44319_2025_472_MOESM4_ESM.zip › Figure 2/2D/Ladder+GSK3/LadderGSK3Chemi.tif]

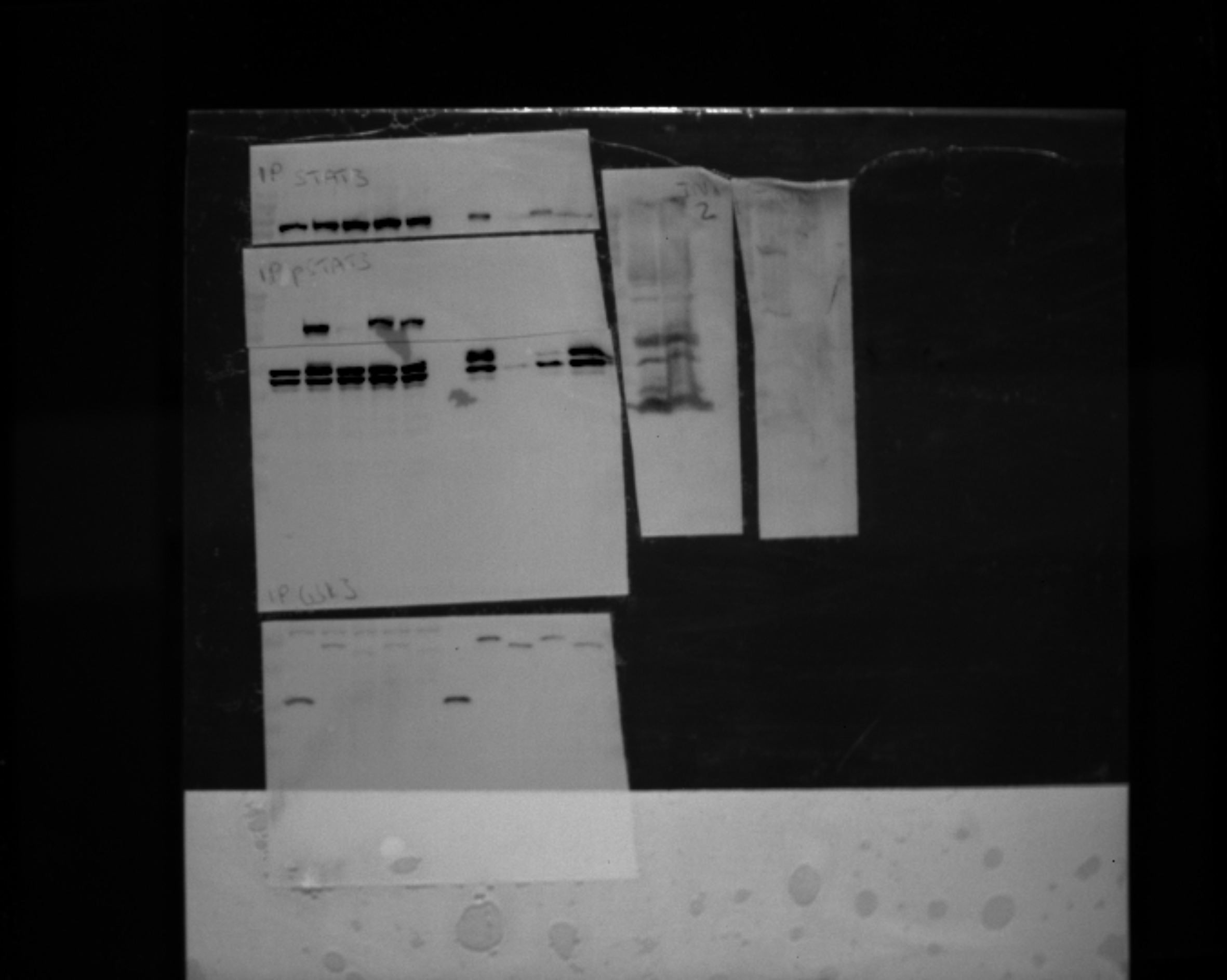

Supplement: Supplementary file 4 — Source data Fig. 2 [file 44319_2025_472_MOESM4_ESM.zip › Figure 2/2E/Ladder+STAT3_top/LadderSTAT3_top_composite.tif]

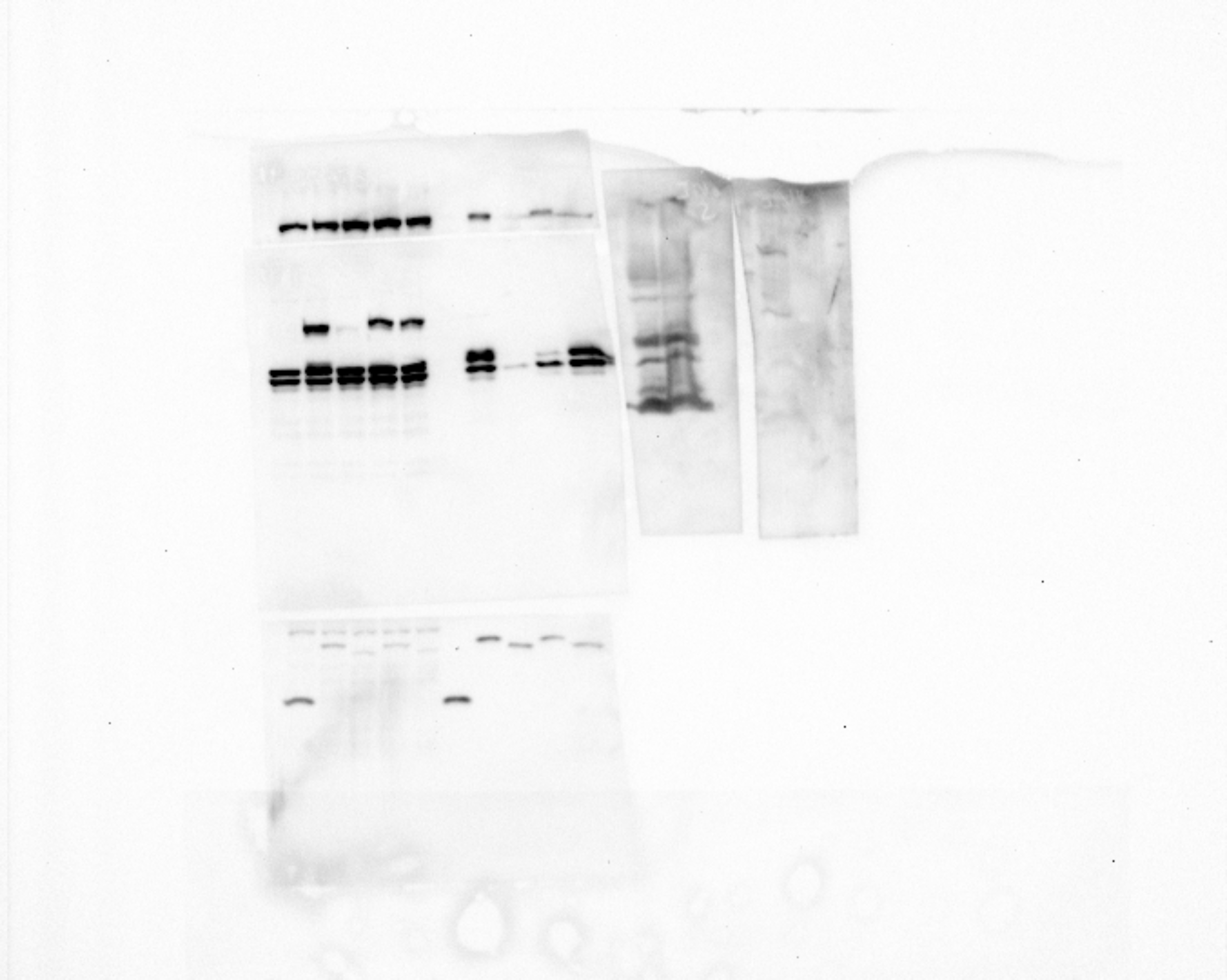

Supplement: Supplementary file 4 — Source data Fig. 2 [file 44319_2025_472_MOESM4_ESM.zip › Figure 2/2E/Ladder+STAT3_top/LadderSTAT3_topChemi.tif]

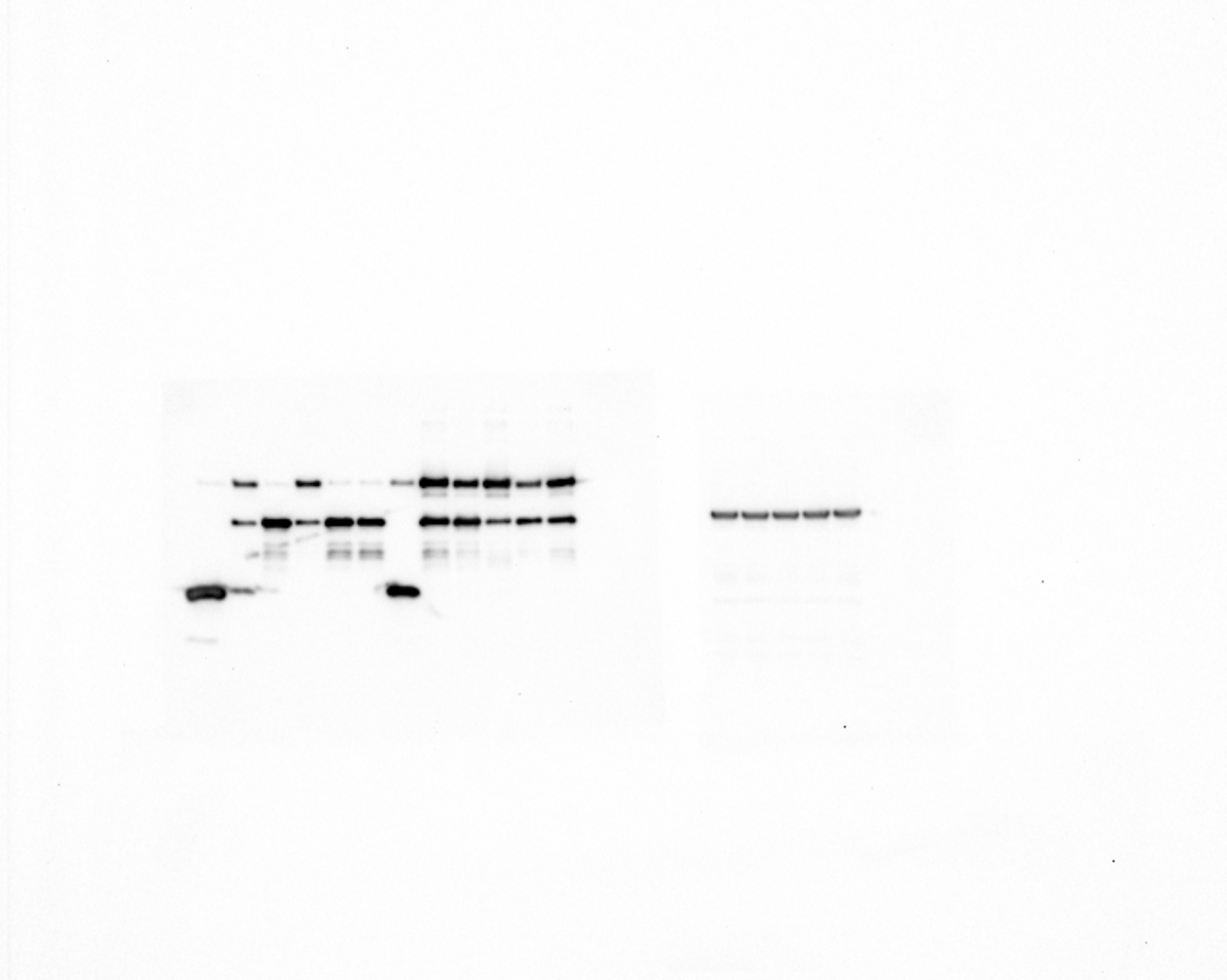

Supplement: Supplementary file 4 — Source data Fig. 2 [file 44319_2025_472_MOESM4_ESM.zip › Figure 2/2E/Ladder+Tub/LadderTubChemi.tif]

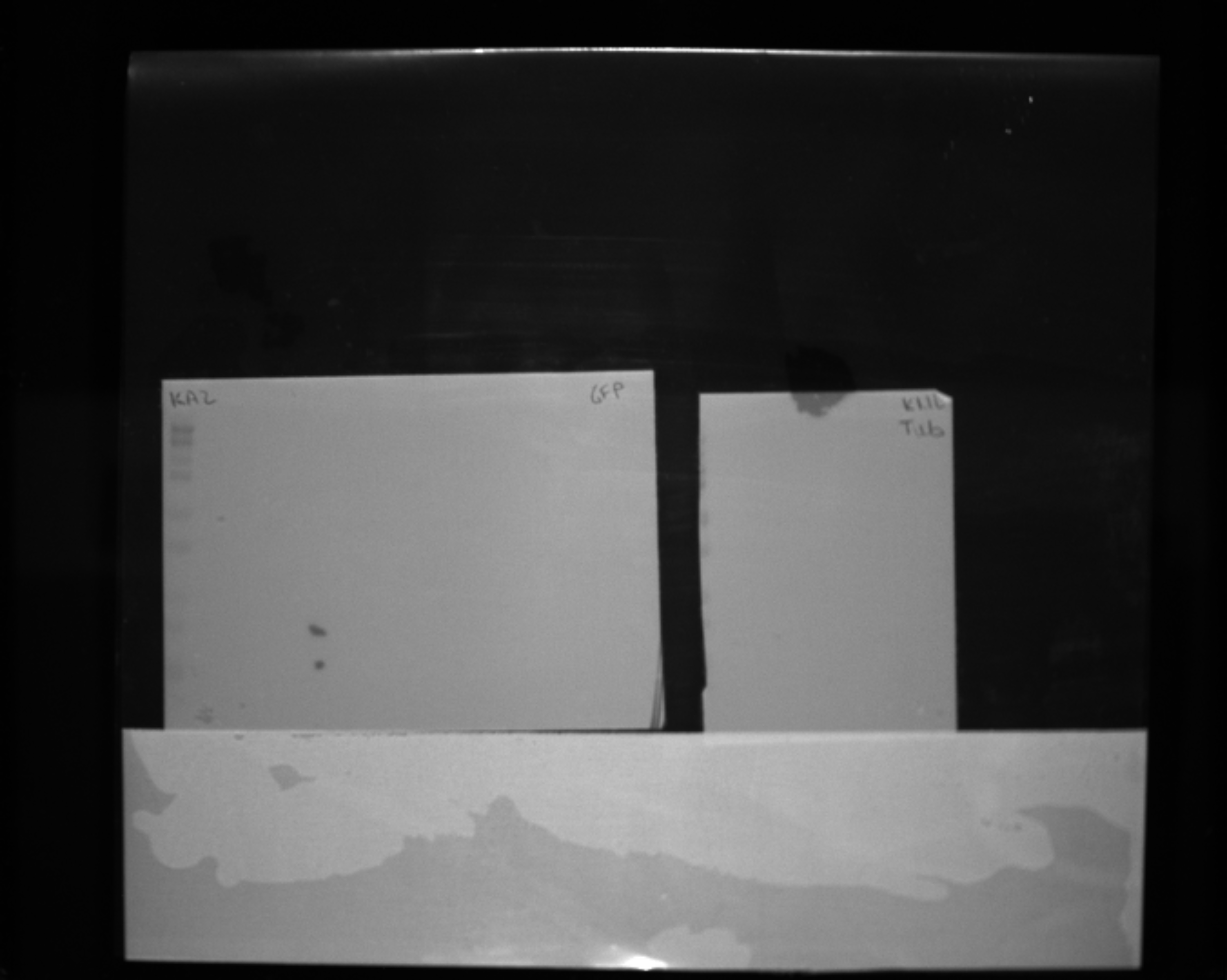

Supplement: Supplementary file 4 — Source data Fig. 2 [file 44319_2025_472_MOESM4_ESM.zip › Figure 2/2E/Ladder+Tub/LadderTubMembrane.tif]

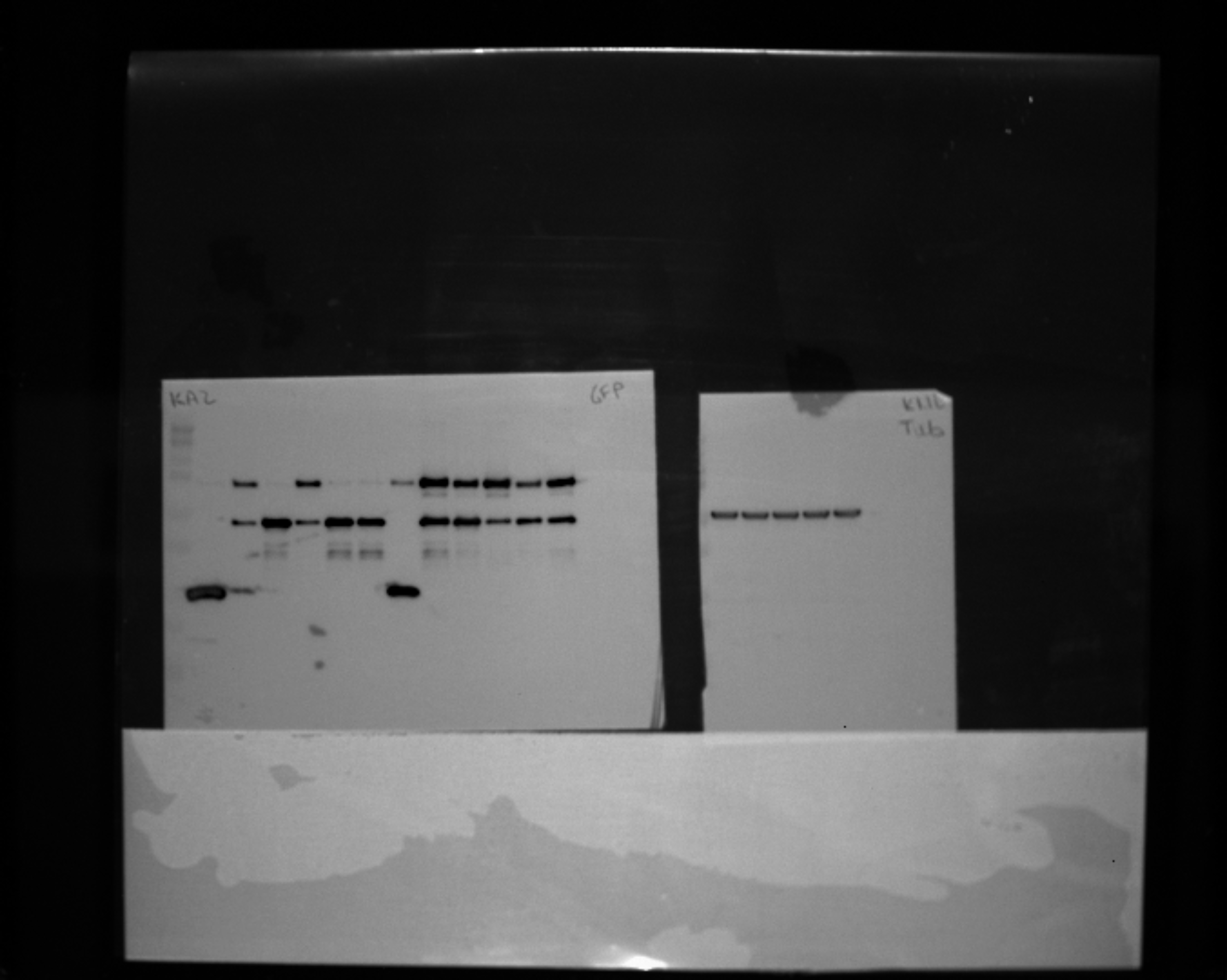

Supplement: Supplementary file 4 — Source data Fig. 2 [file 44319_2025_472_MOESM4_ESM.zip › Figure 2/2E/Ladder+Tub/LadderTub_composite.tif]

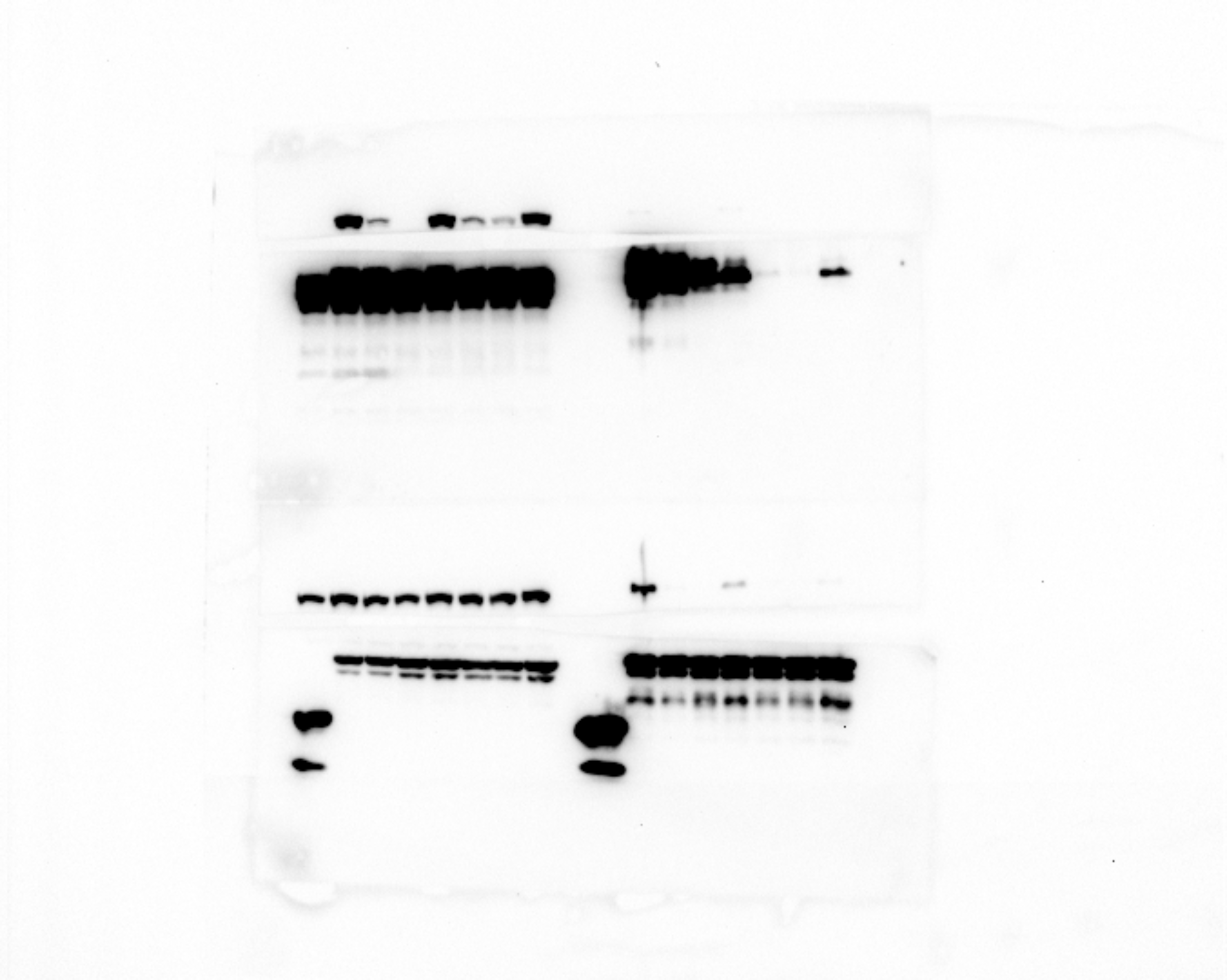

Supplement: Supplementary file 4 — Source data Fig. 2 [file 44319_2025_472_MOESM4_ESM.zip › Figure 2/2B/Ladder+STAT3/LadderSTAT3Chemi.tif]

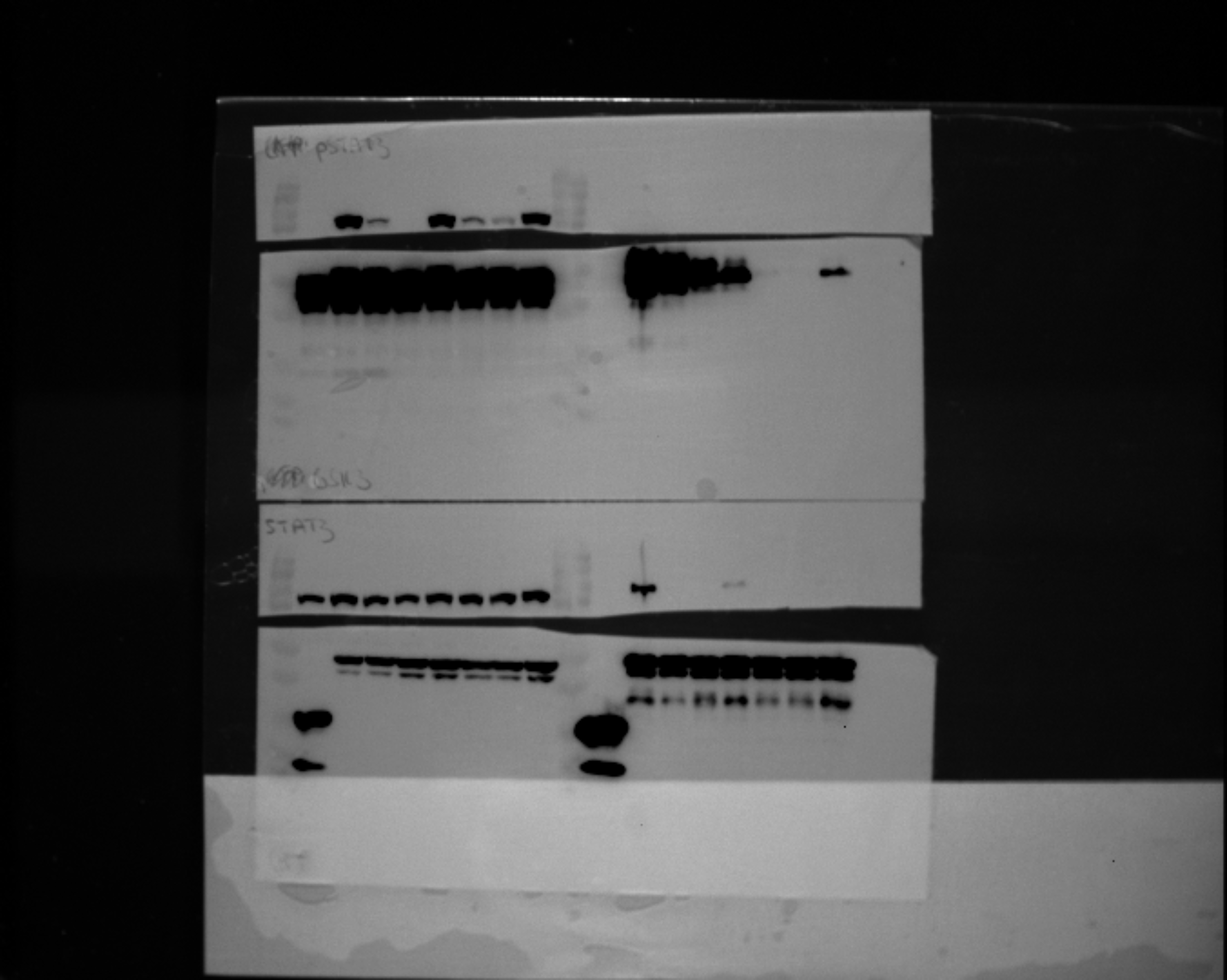

Supplement: Supplementary file 4 — Source data Fig. 2 [file 44319_2025_472_MOESM4_ESM.zip › Figure 2/2B/Ladder+STAT3/LadderSTAT3_composite.tif]

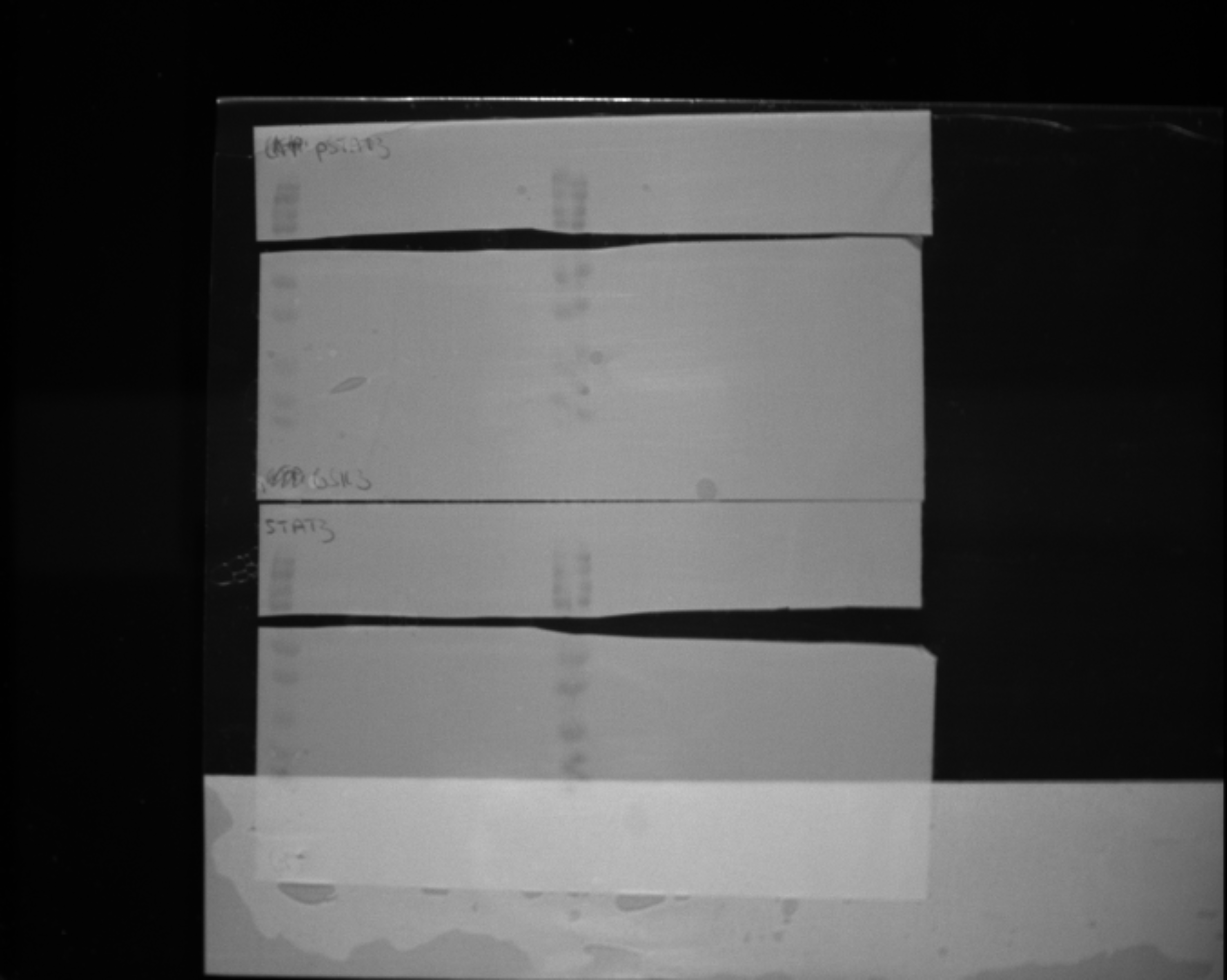

Supplement: Supplementary file 4 — Source data Fig. 2 [file 44319_2025_472_MOESM4_ESM.zip › Figure 2/2B/Ladder+STAT3/LadderSTAT3Membrane.tif]

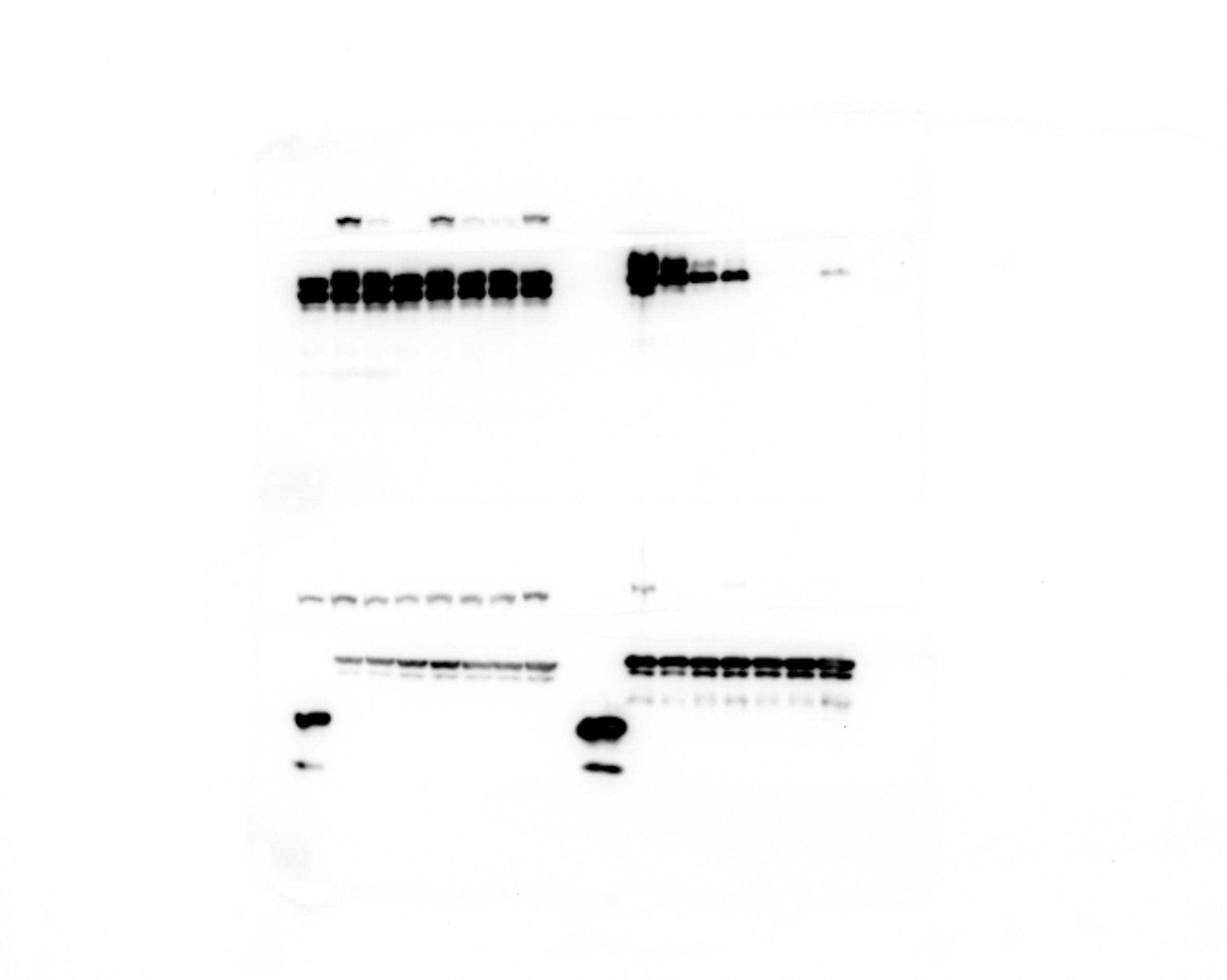

Supplement: Supplementary file 4 — Source data Fig. 2 [file 44319_2025_472_MOESM4_ESM.zip › Figure 2/2B/Ladder+GFP/LadderGFPChemi.tif]

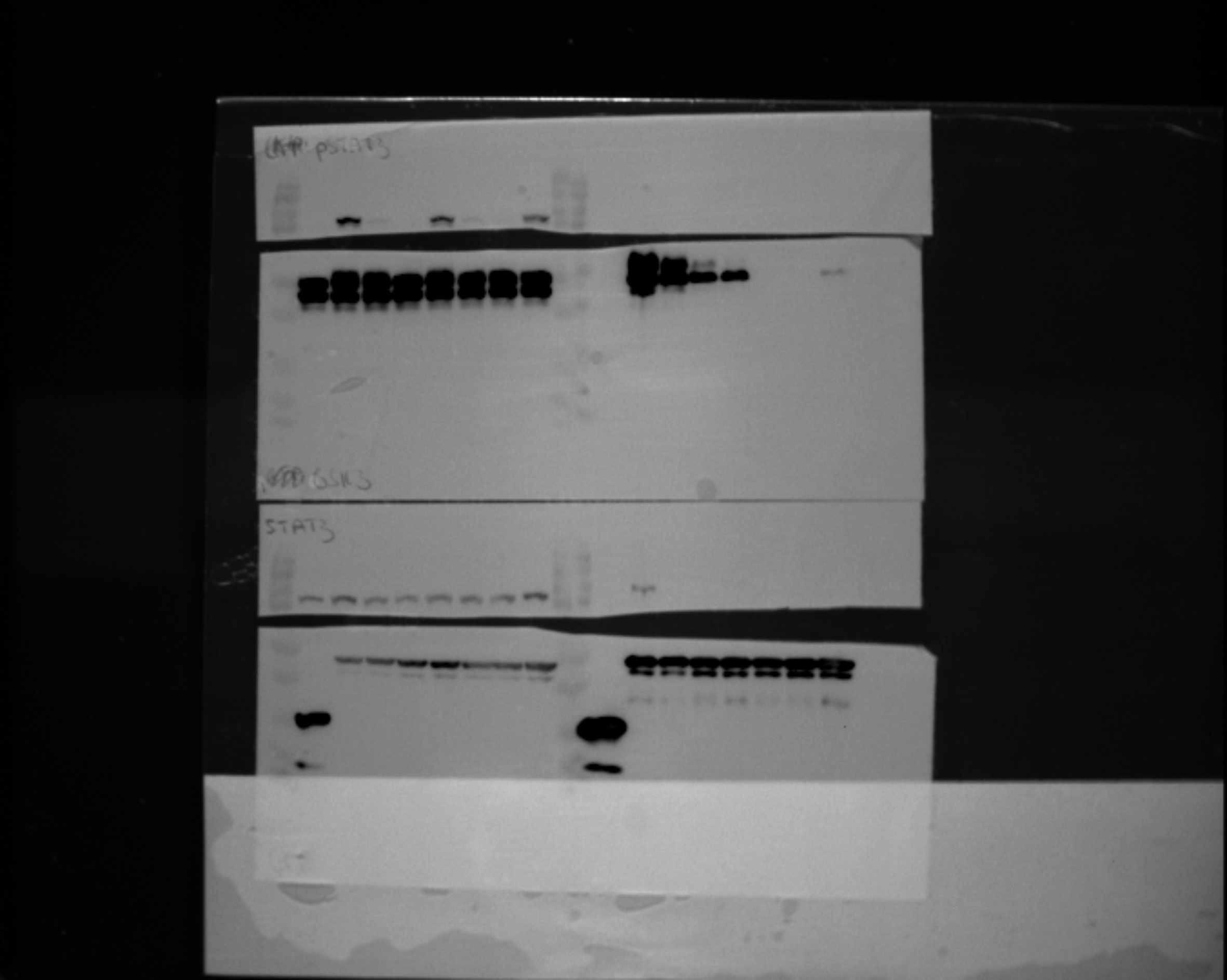

Supplement: Supplementary file 4 — Source data Fig. 2 [file 44319_2025_472_MOESM4_ESM.zip › Figure 2/2B/Ladder+GFP/LadderGFP_composite.tif]

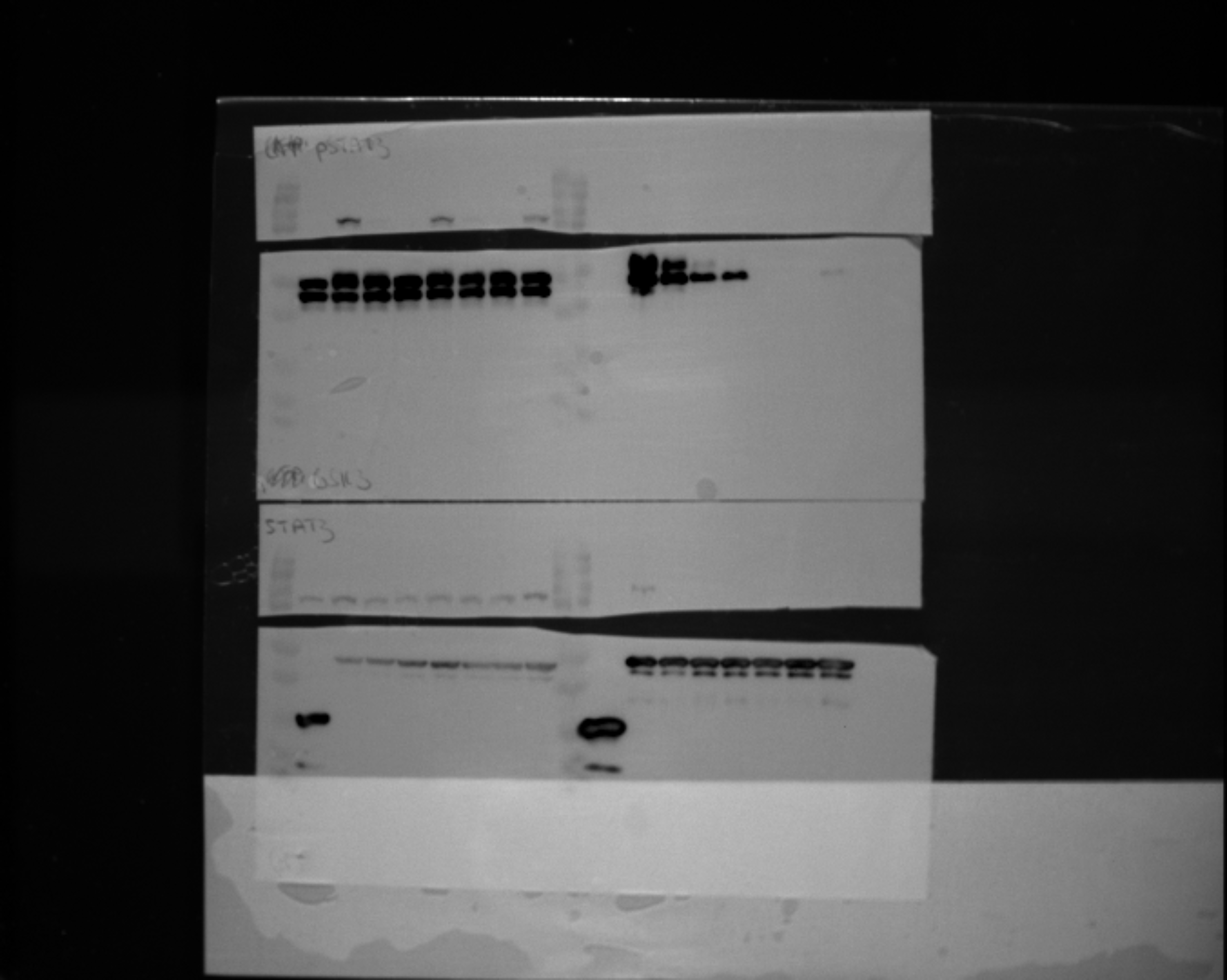

Supplement: Supplementary file 4 — Source data Fig. 2 [file 44319_2025_472_MOESM4_ESM.zip › Figure 2/2B/Ladder+GSK3/LadderGSK3_composite.tif]

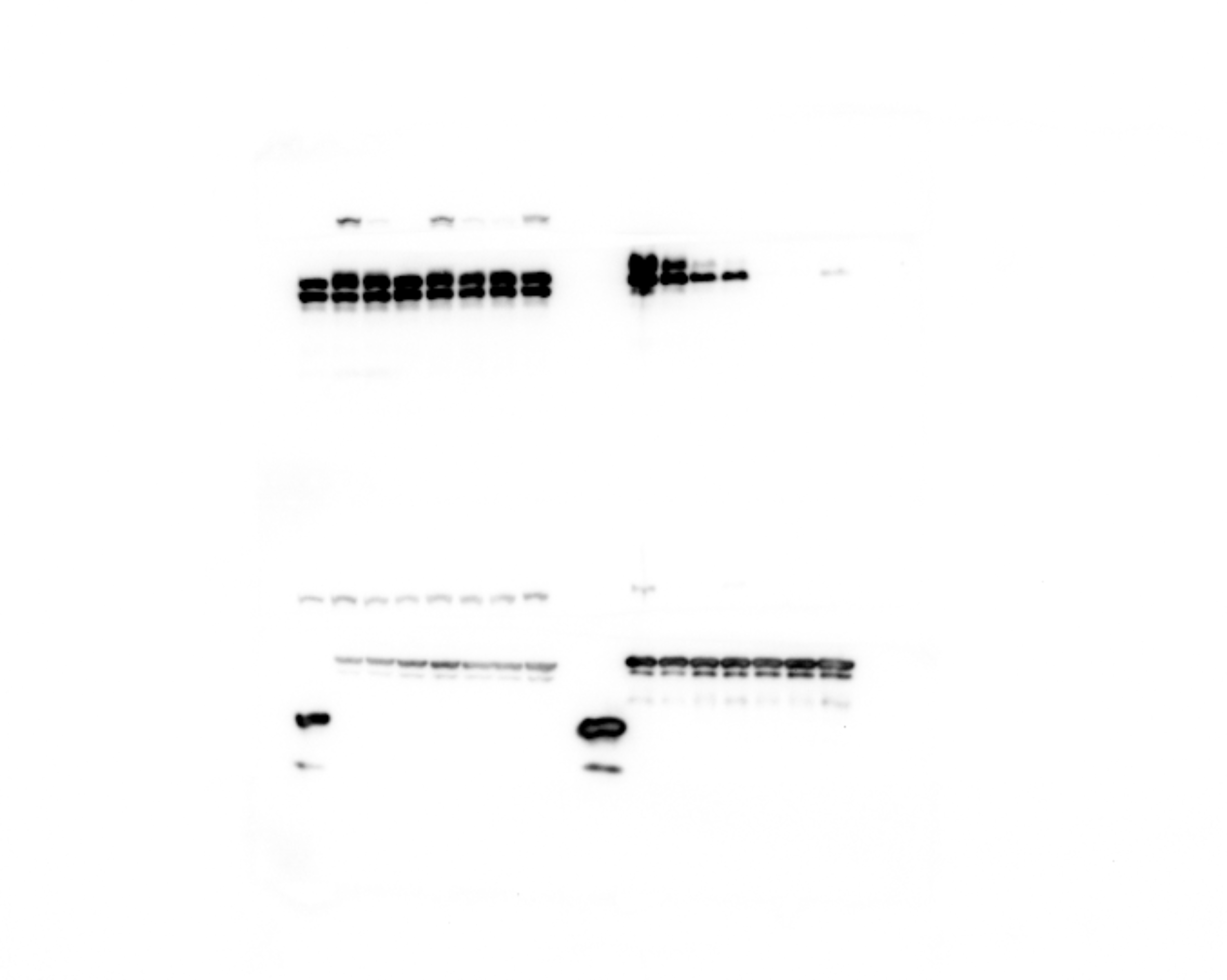

Supplement: Supplementary file 4 — Source data Fig. 2 [file 44319_2025_472_MOESM4_ESM.zip › Figure 2/2B/Ladder+GSK3/LadderGSK3Chemi.tif]

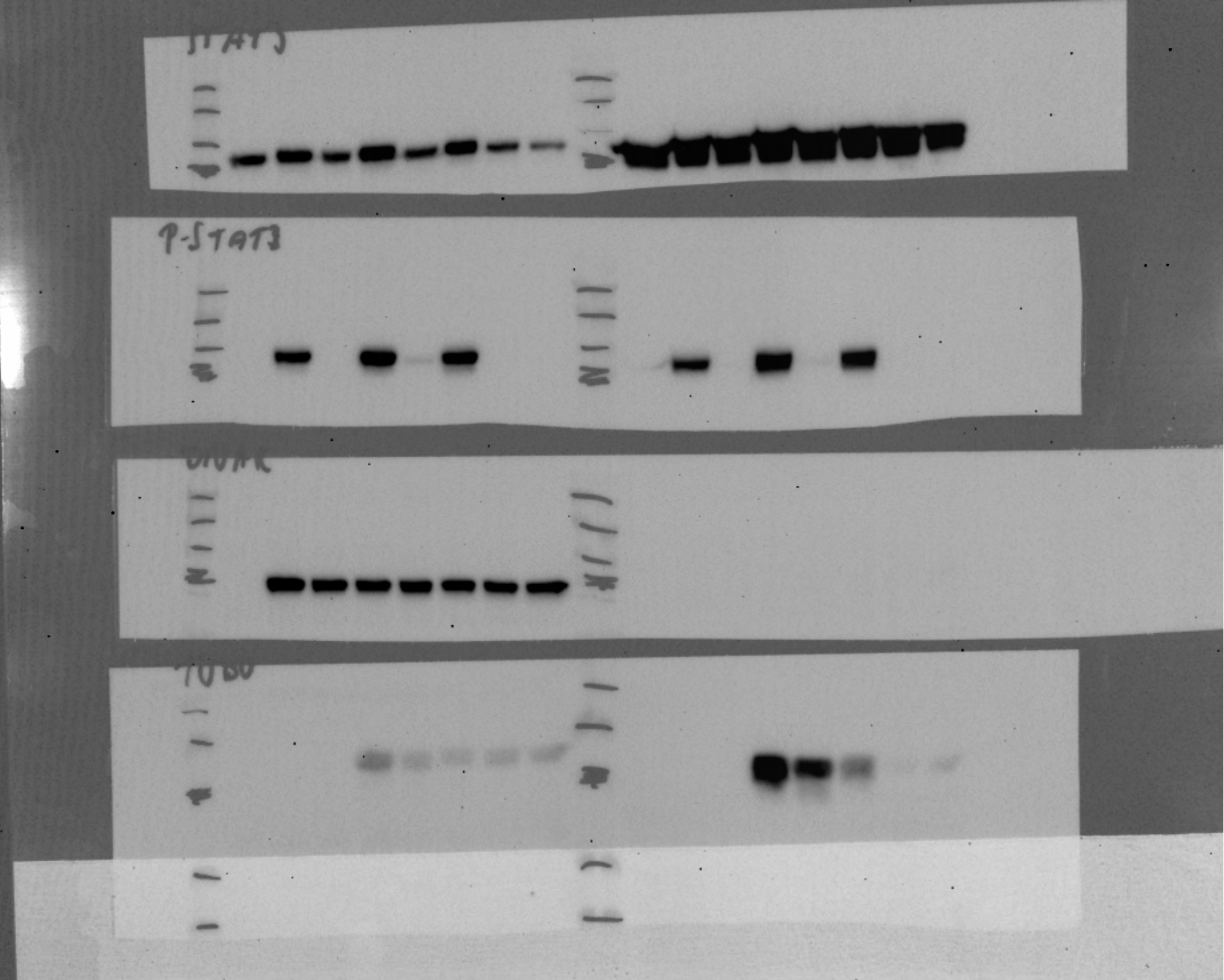

Supplement: Supplementary file 5 — Source data Fig. 3 [file 44319_2025_472_MOESM5_ESM.zip › Figure 3/3E/STAT3_memb_overlay.tif]

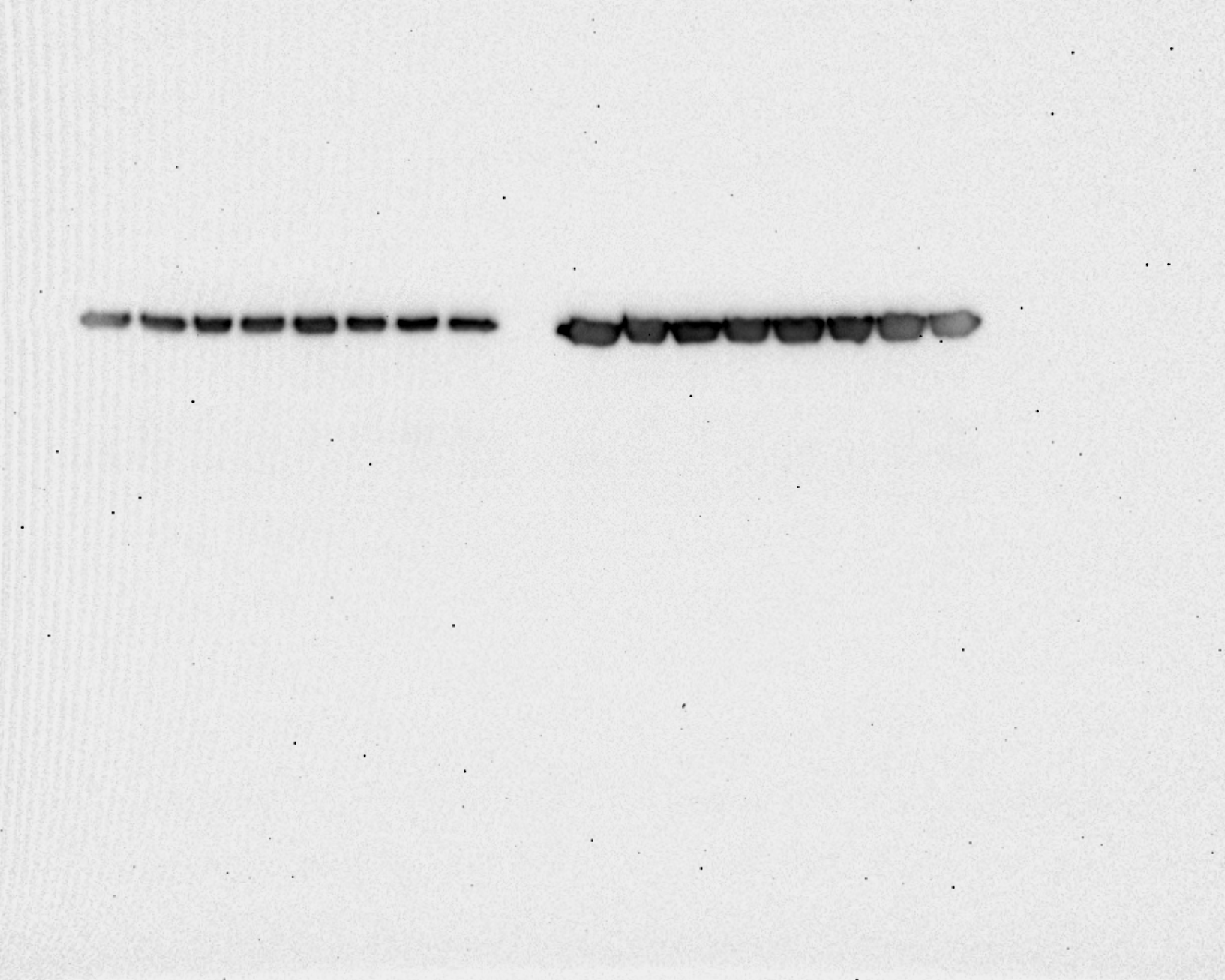

Supplement: Supplementary file 5 — Source data Fig. 3 [file 44319_2025_472_MOESM5_ESM.zip › Figure 3/3E/Tubulin.tif]

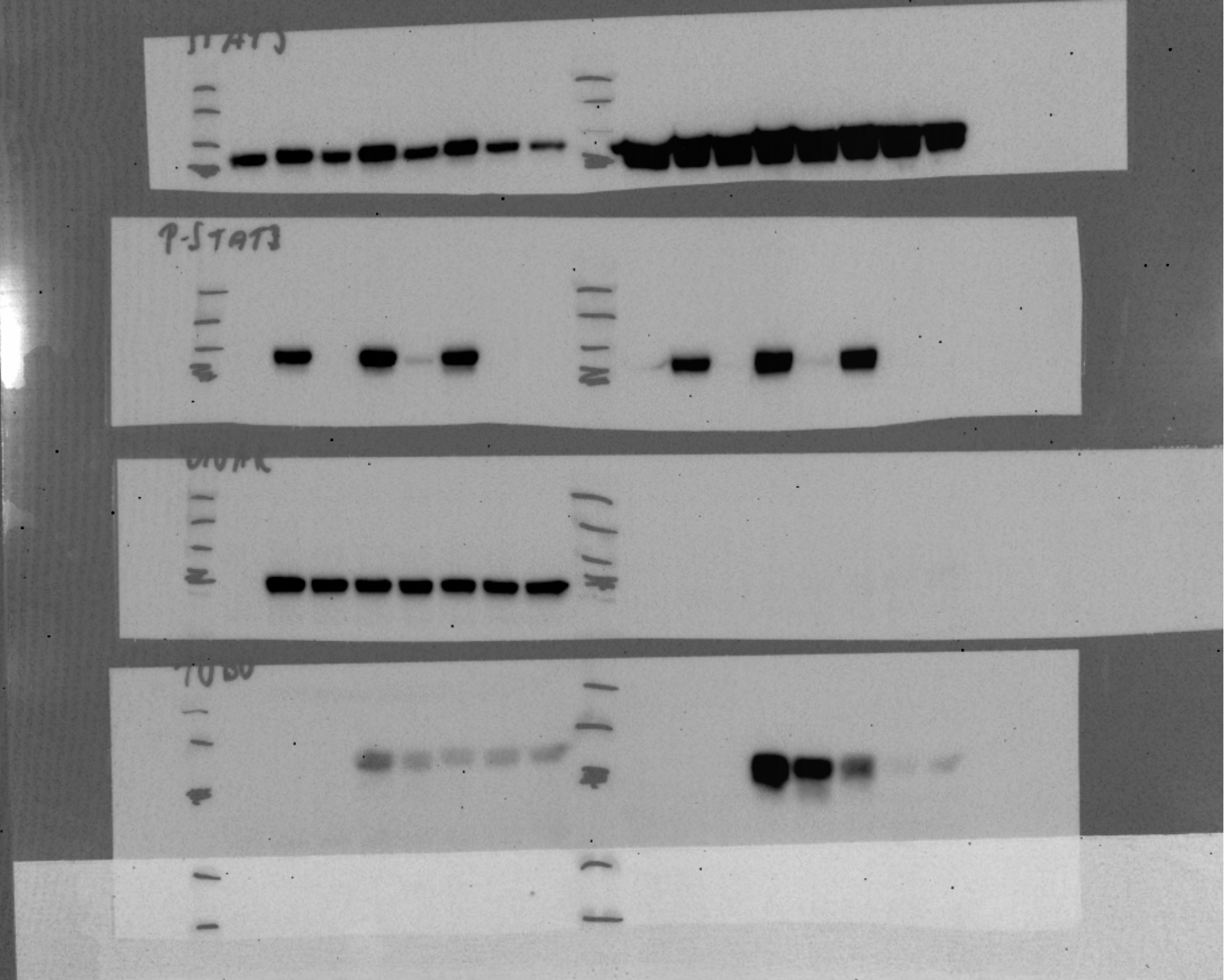

Supplement: Supplementary file 5 — Source data Fig. 3 [file 44319_2025_472_MOESM5_ESM.zip › Figure 3/3E/HA_memb_overlay.tif]

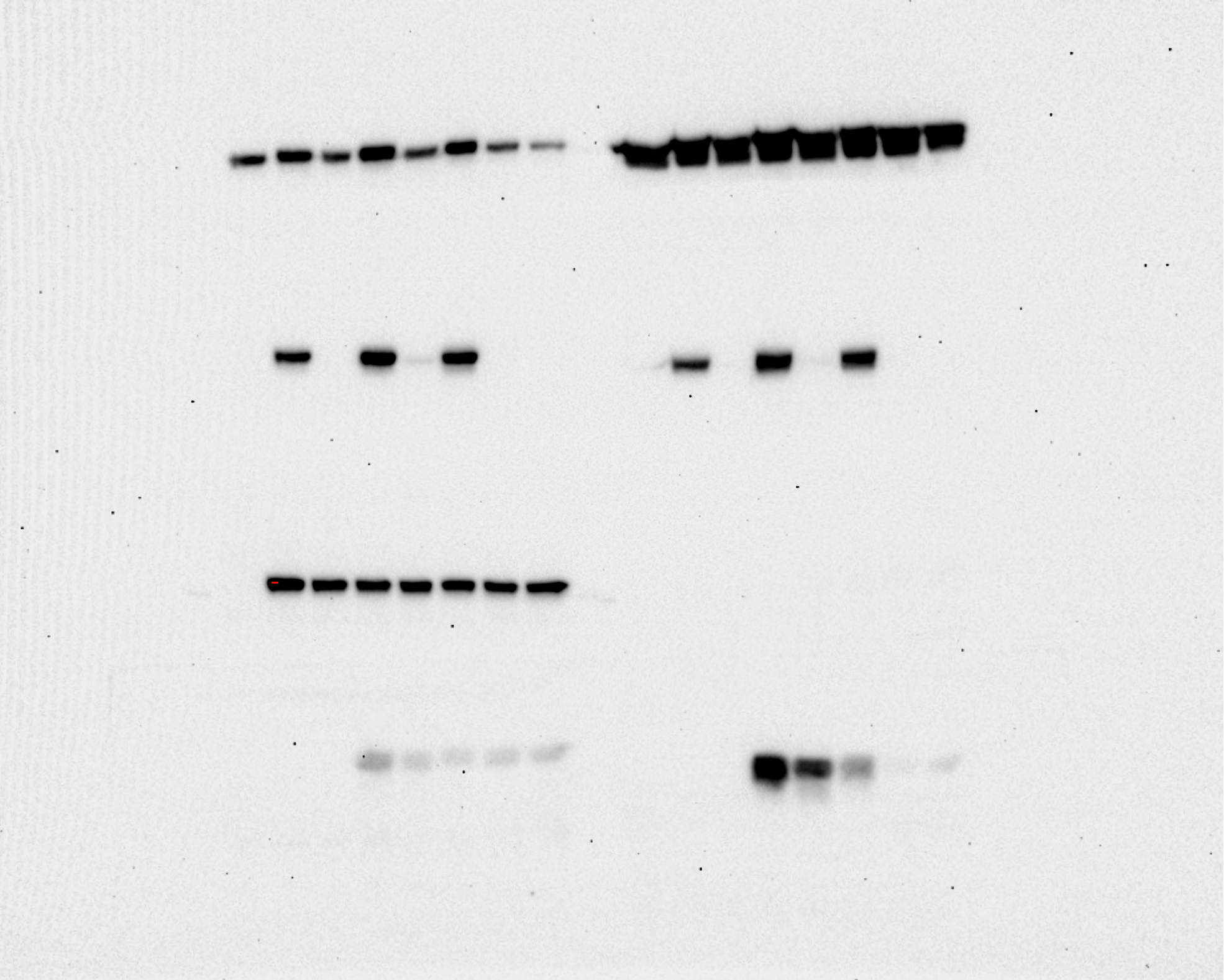

Supplement: Supplementary file 5 — Source data Fig. 3 [file 44319_2025_472_MOESM5_ESM.zip › Figure 3/3E/pY705-STAT3_2nd.tif]

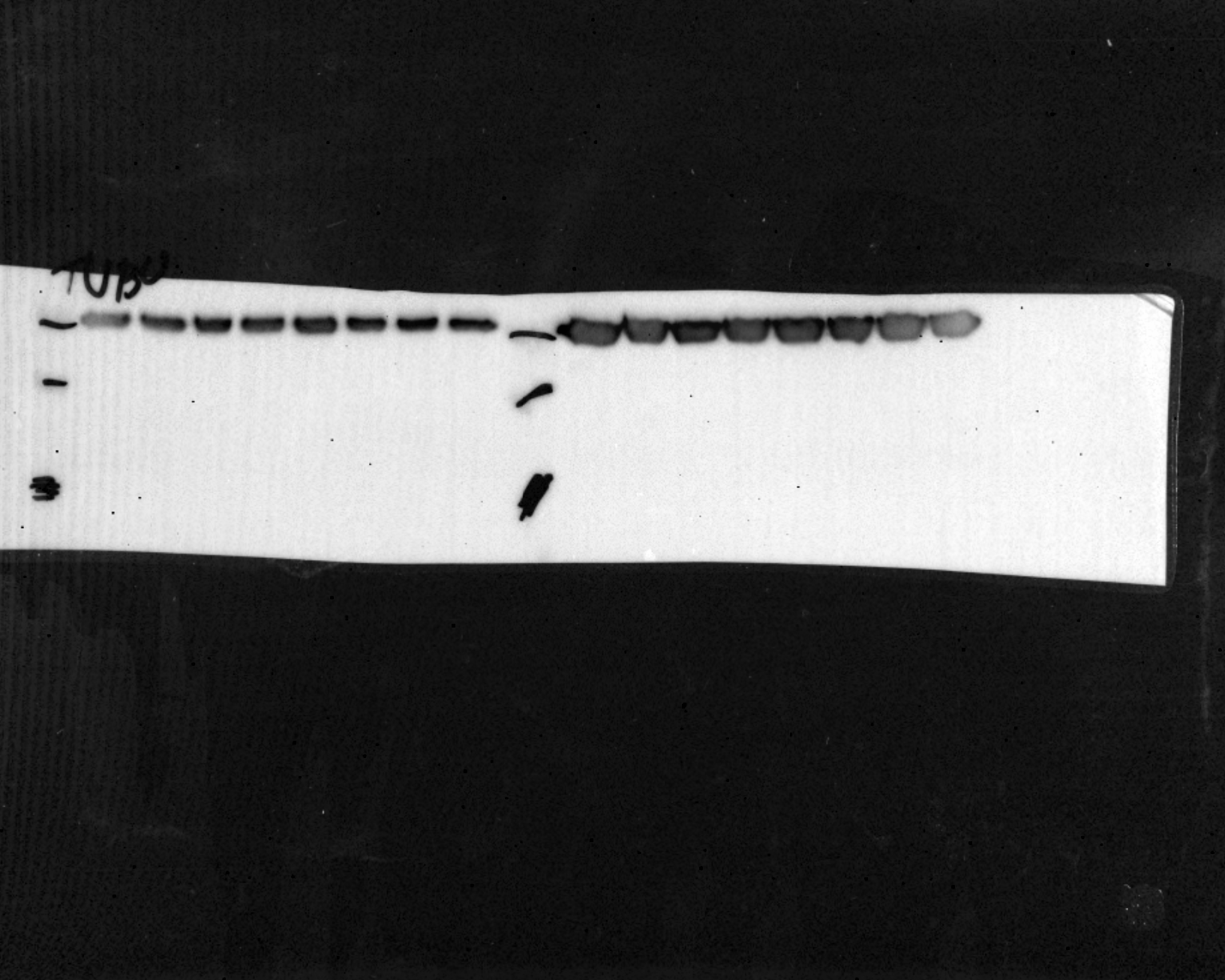

Supplement: Supplementary file 5 — Source data Fig. 3 [file 44319_2025_472_MOESM5_ESM.zip › Figure 3/3E/Tubulin_memb_overlay.tif]

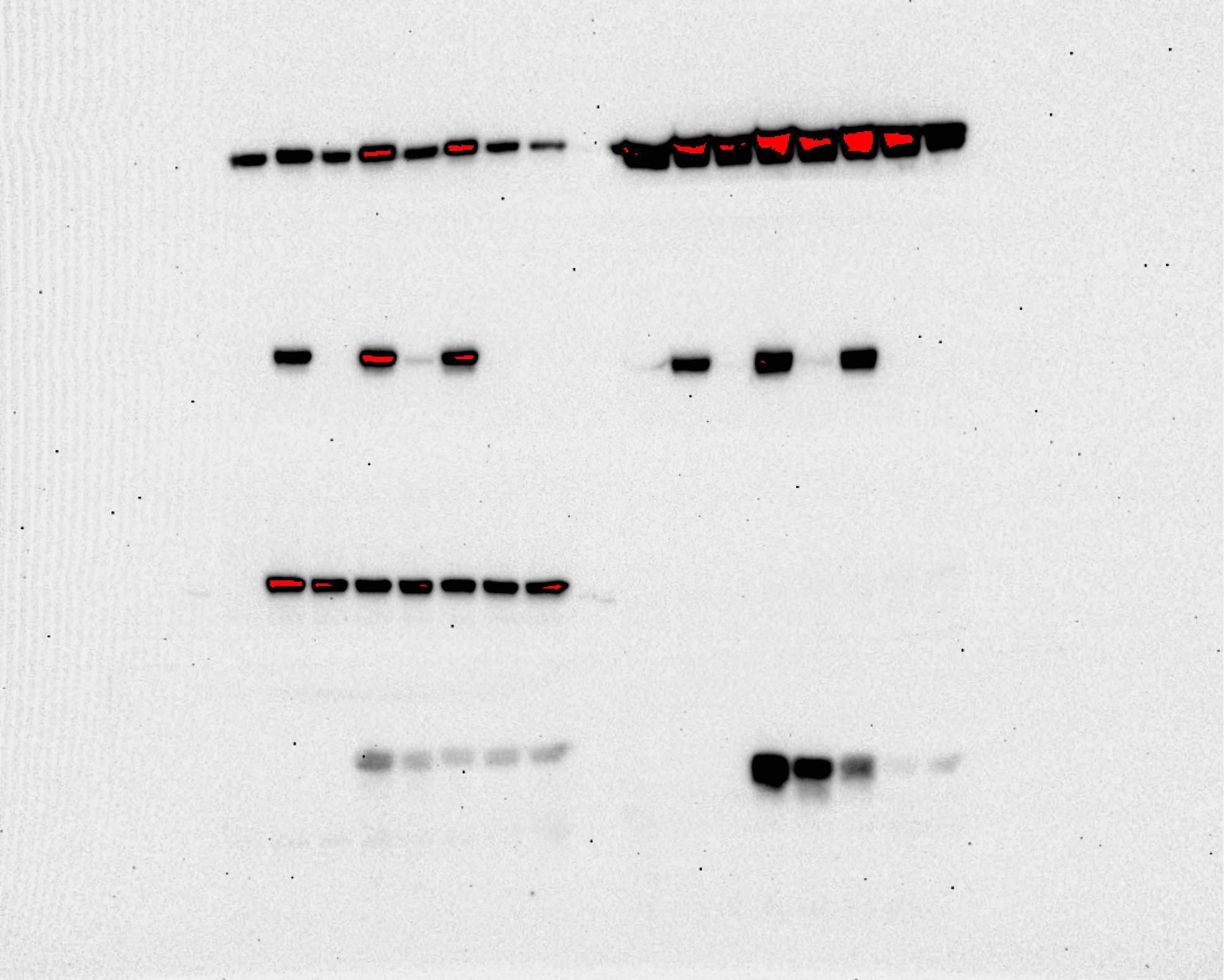

Supplement: Supplementary file 5 — Source data Fig. 3 [file 44319_2025_472_MOESM5_ESM.zip › Figure 3/3E/HA_last.tif]

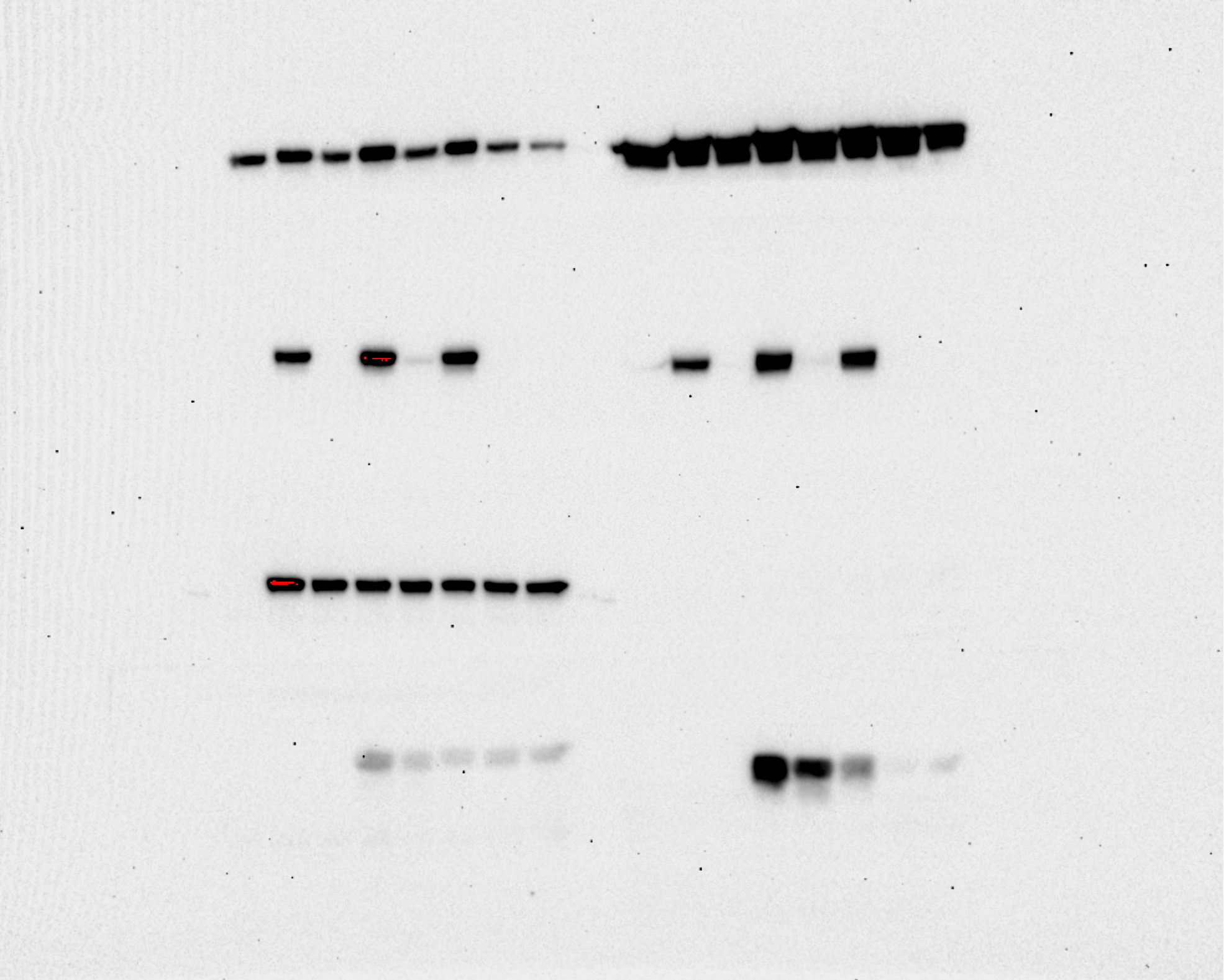

Supplement: Supplementary file 5 — Source data Fig. 3 [file 44319_2025_472_MOESM5_ESM.zip › Figure 3/3E/STAT3_1st.tif]

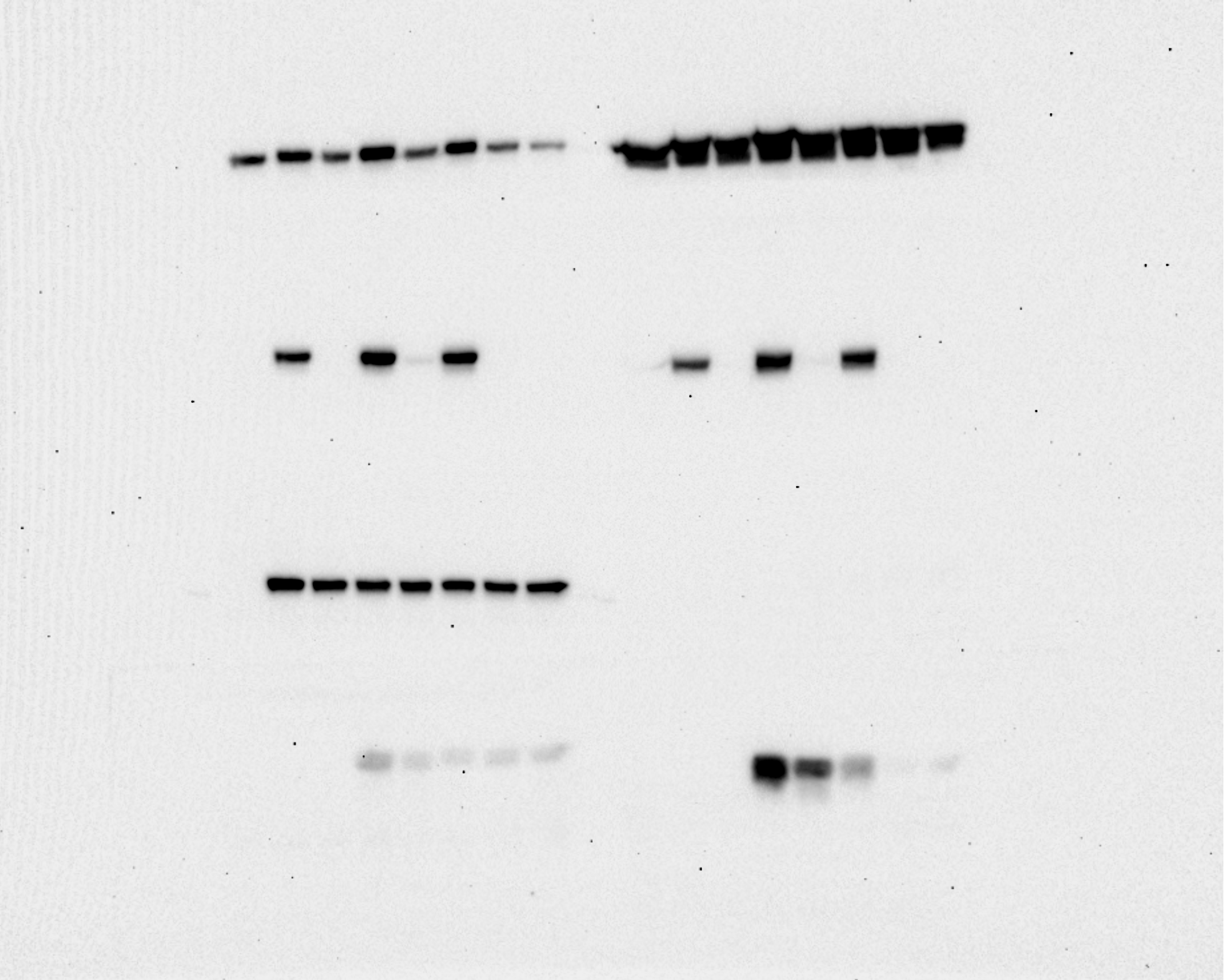

Supplement: Supplementary file 5 — Source data Fig. 3 [file 44319_2025_472_MOESM5_ESM.zip › Figure 3/3E/DnaK_3rd.tif]

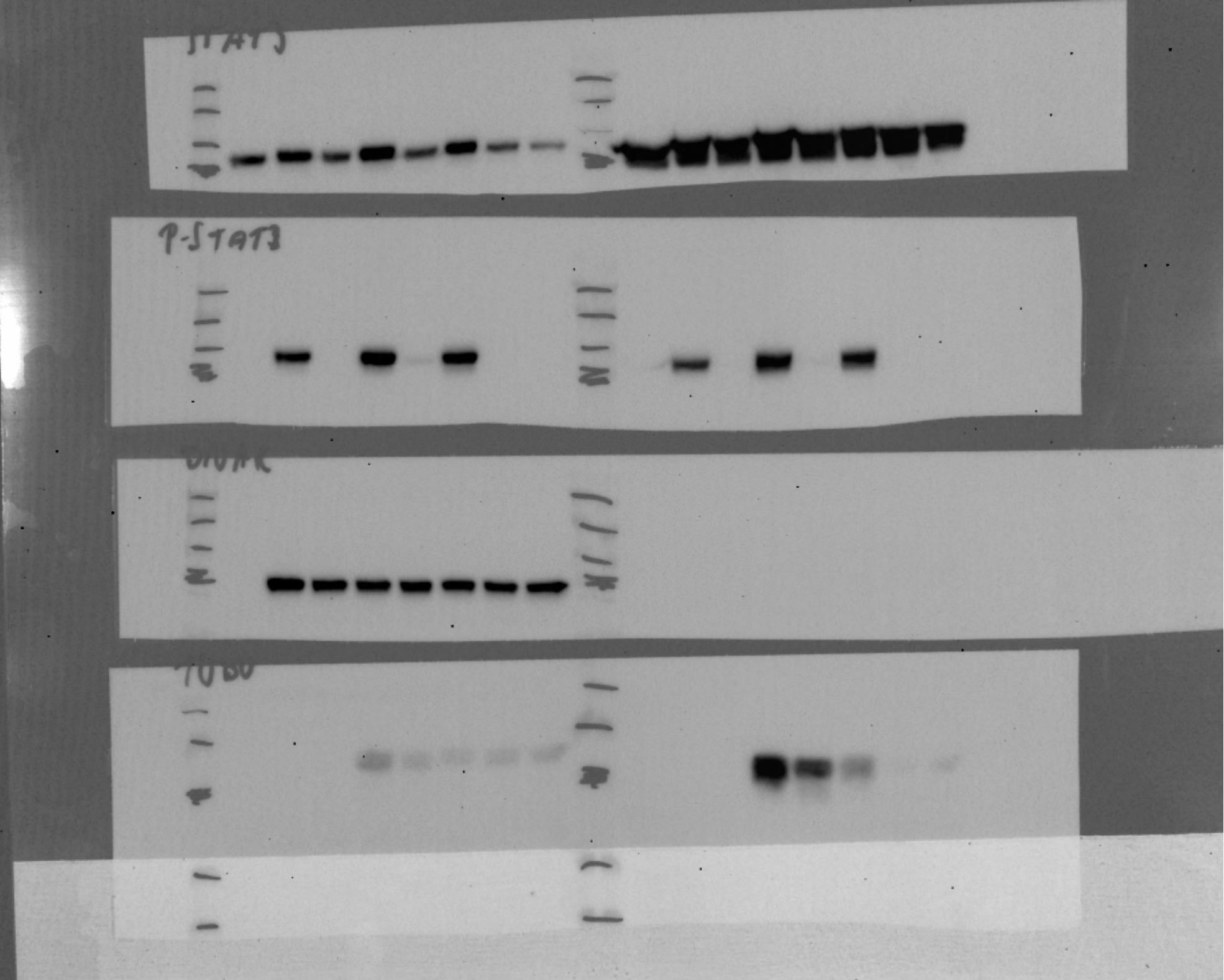

Supplement: Supplementary file 5 — Source data Fig. 3 [file 44319_2025_472_MOESM5_ESM.zip › Figure 3/3E/DnaK_memb_overlay.tif]

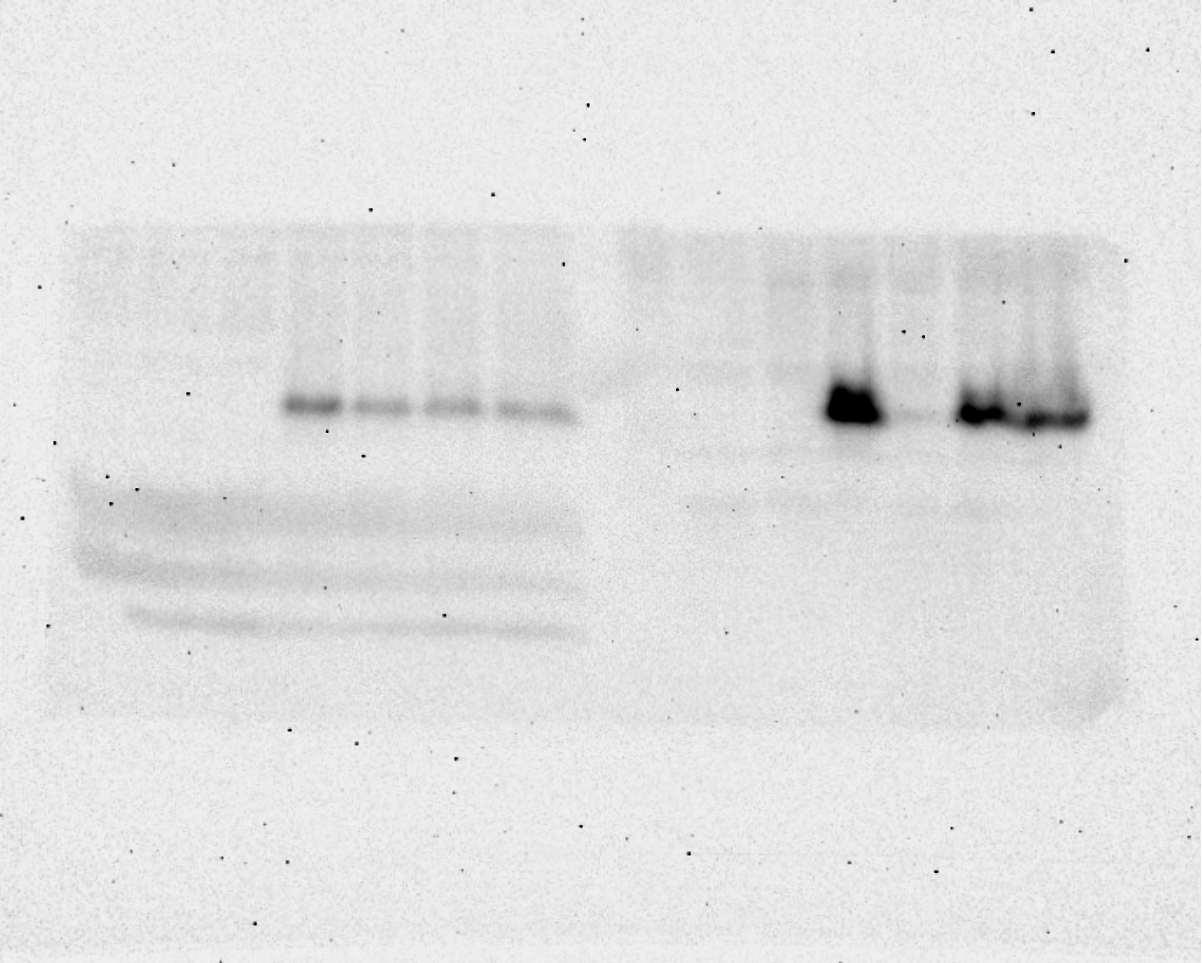

Supplement: Supplementary file 5 — Source data Fig. 3 [file 44319_2025_472_MOESM5_ESM.zip › Figure 3/3A/HA_raw.tif]

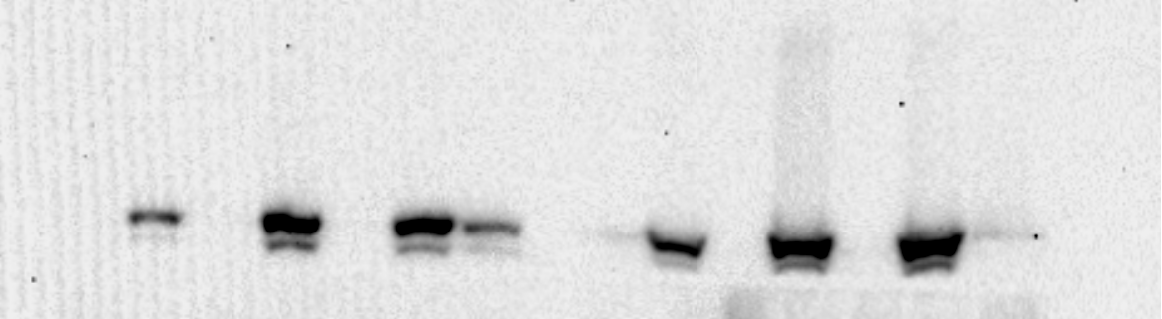

Supplement: Supplementary file 5 — Source data Fig. 3 [file 44319_2025_472_MOESM5_ESM.zip › Figure 3/3A/pY705-STAT3_raw.tif]

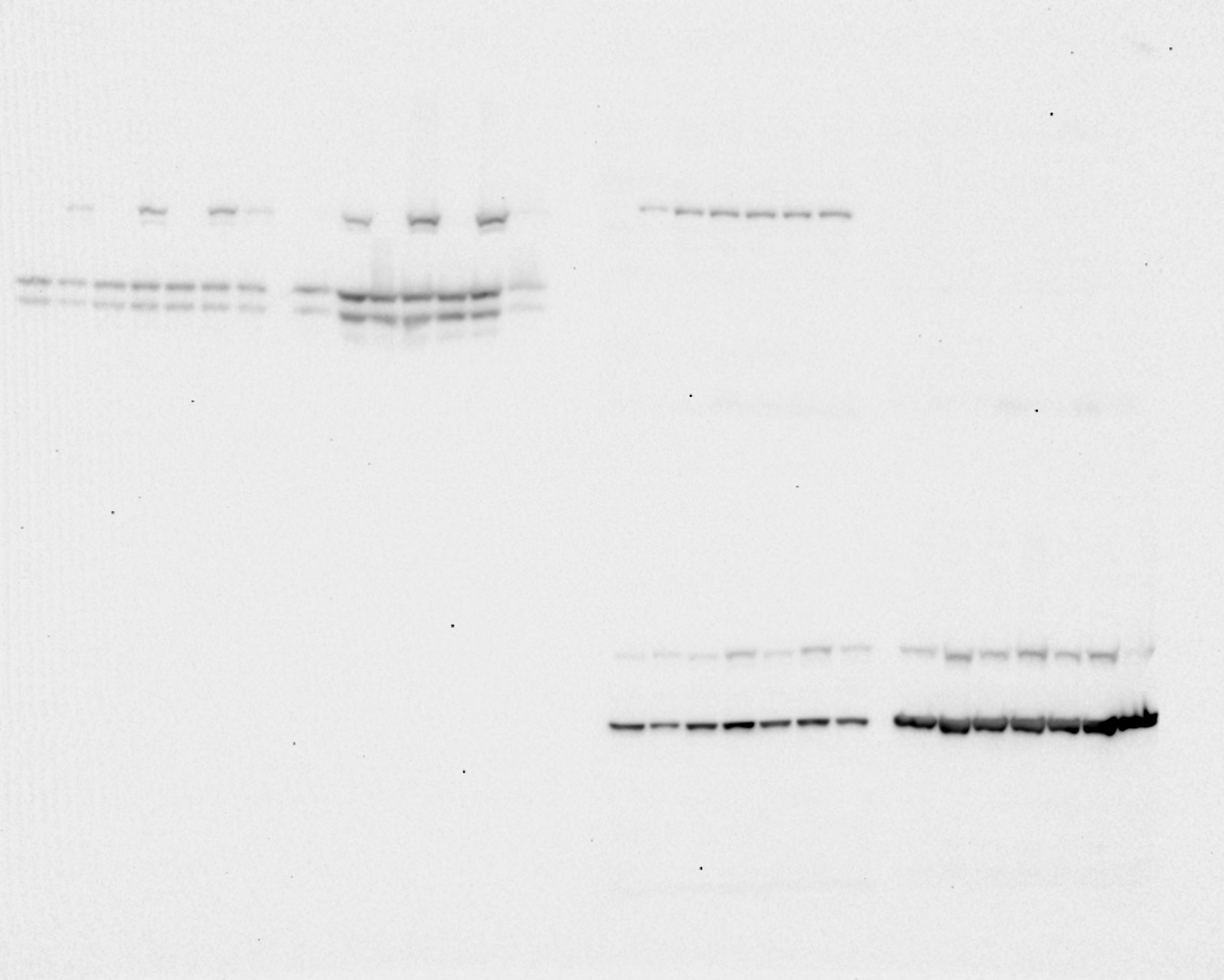

Supplement: Supplementary file 5 — Source data Fig. 3 [file 44319_2025_472_MOESM5_ESM.zip › Figure 3/3A/Tubulin_raw.tif]

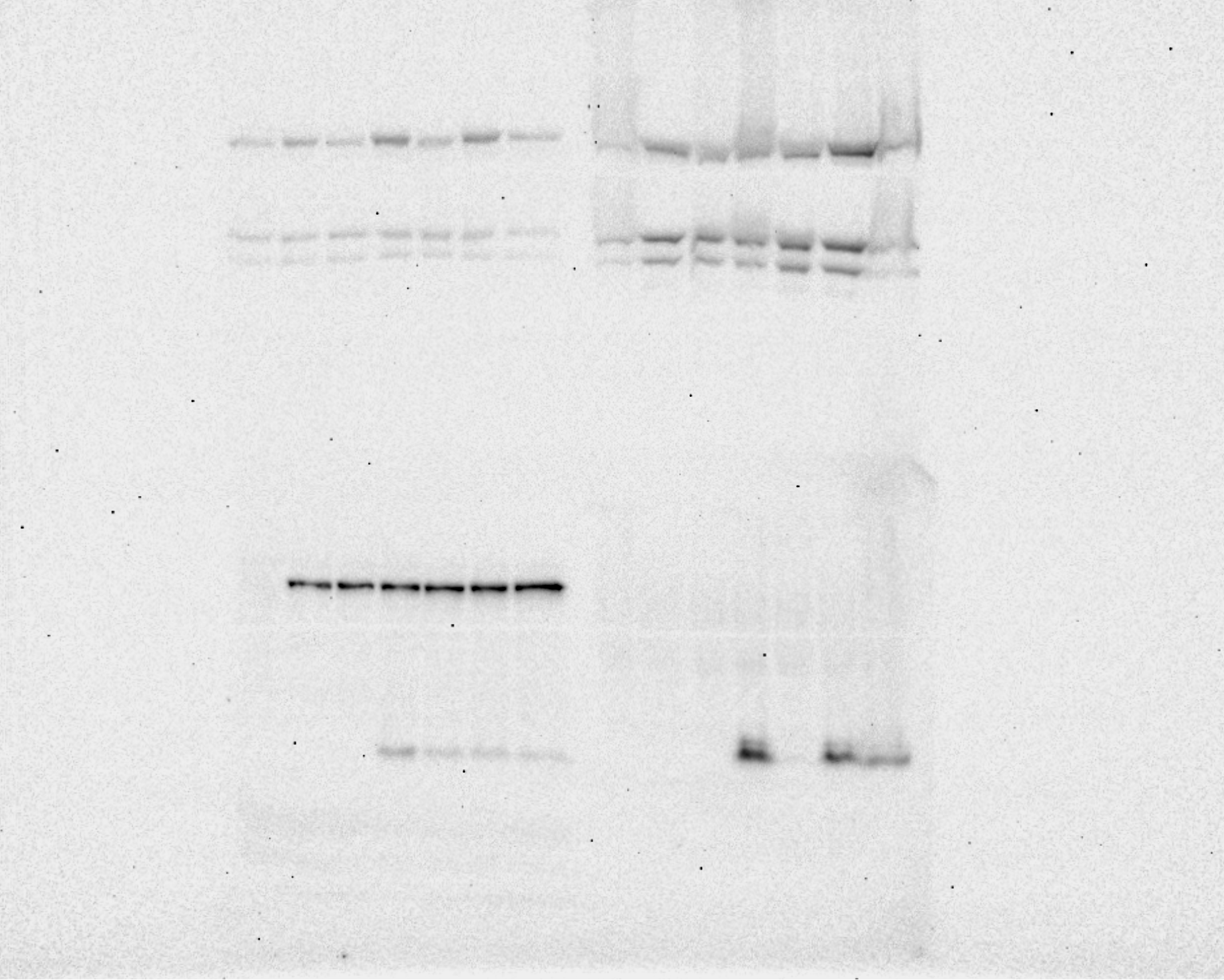

Supplement: Supplementary file 5 — Source data Fig. 3 [file 44319_2025_472_MOESM5_ESM.zip › Figure 3/3A/DnaK_raw.tif]

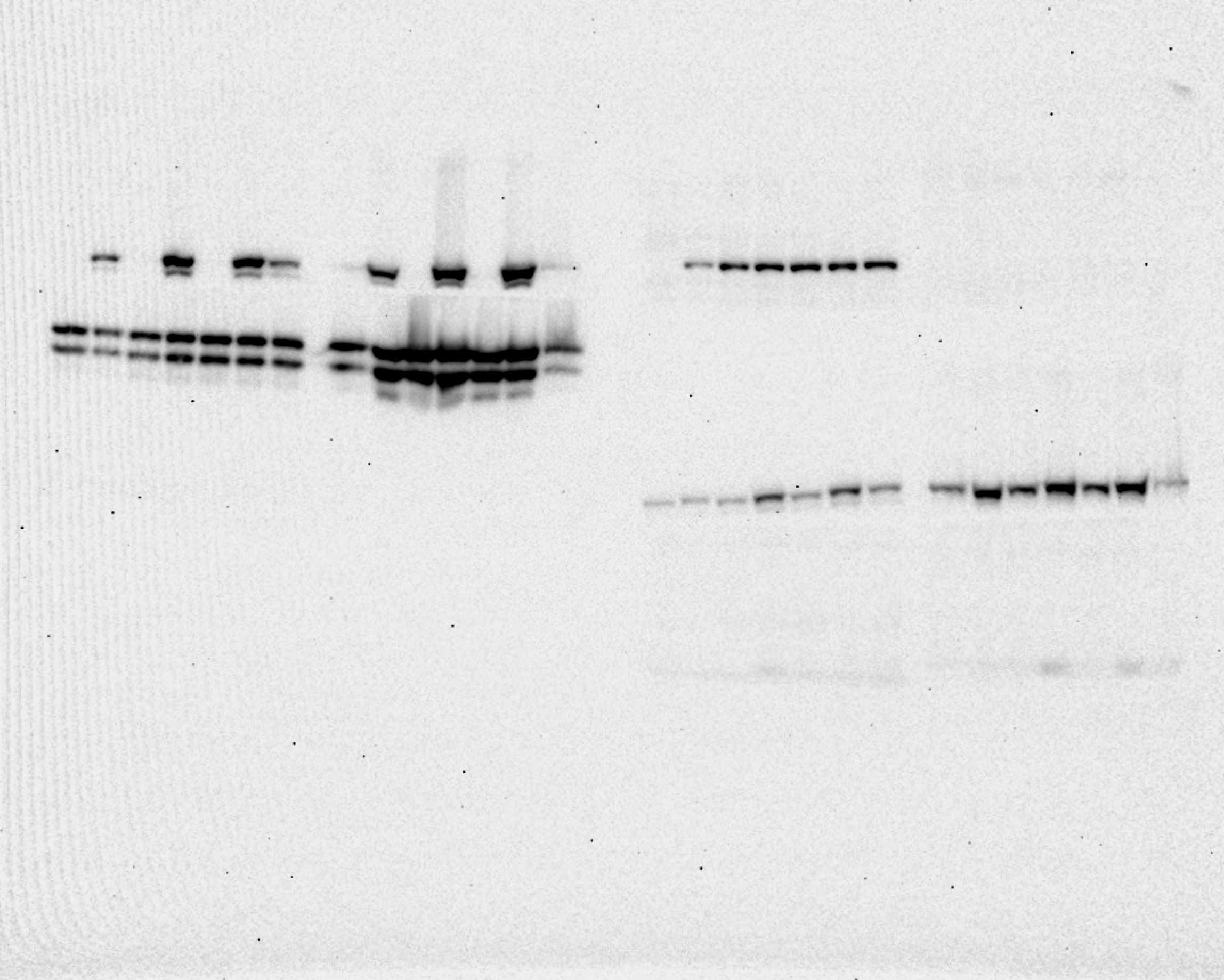

Supplement: Supplementary file 5 — Source data Fig. 3 [file 44319_2025_472_MOESM5_ESM.zip › Figure 3/3A/STAT3_raw_bottom_right.tif]

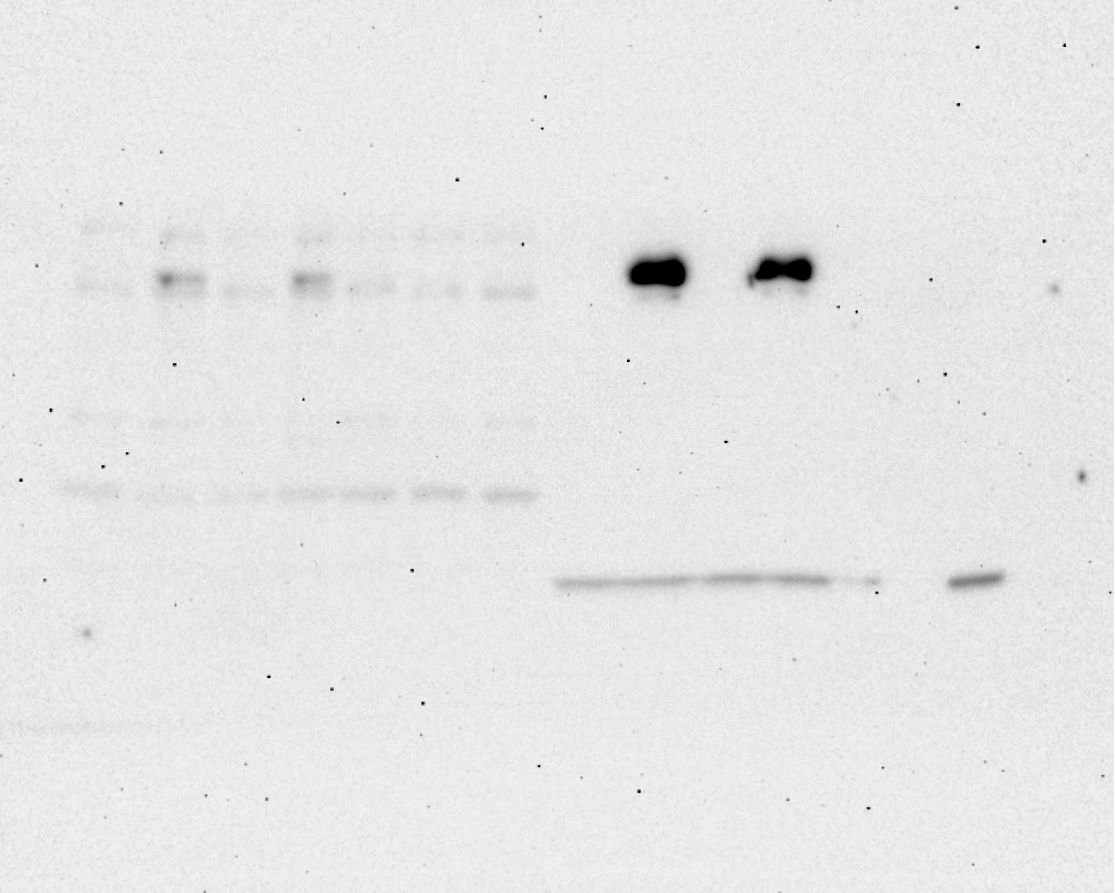

Supplement: Supplementary file 5 — Source data Fig. 3 [file 44319_2025_472_MOESM5_ESM.zip › Figure 3/3F/pTyrosine.tif]

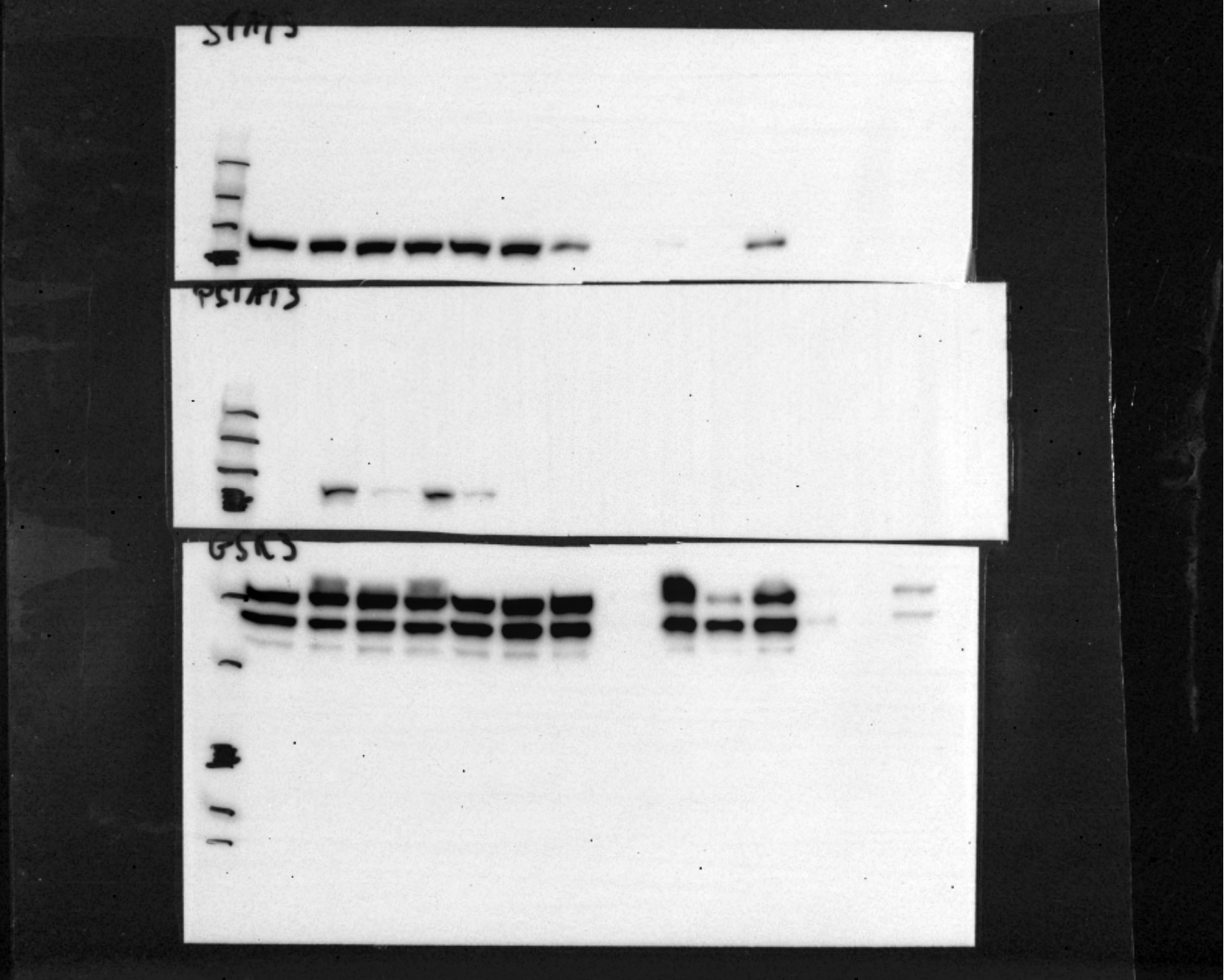

Supplement: Supplementary file 5 — Source data Fig. 3 [file 44319_2025_472_MOESM5_ESM.zip › Figure 3/3F/GSK3_memb_overlay_lower.tif]

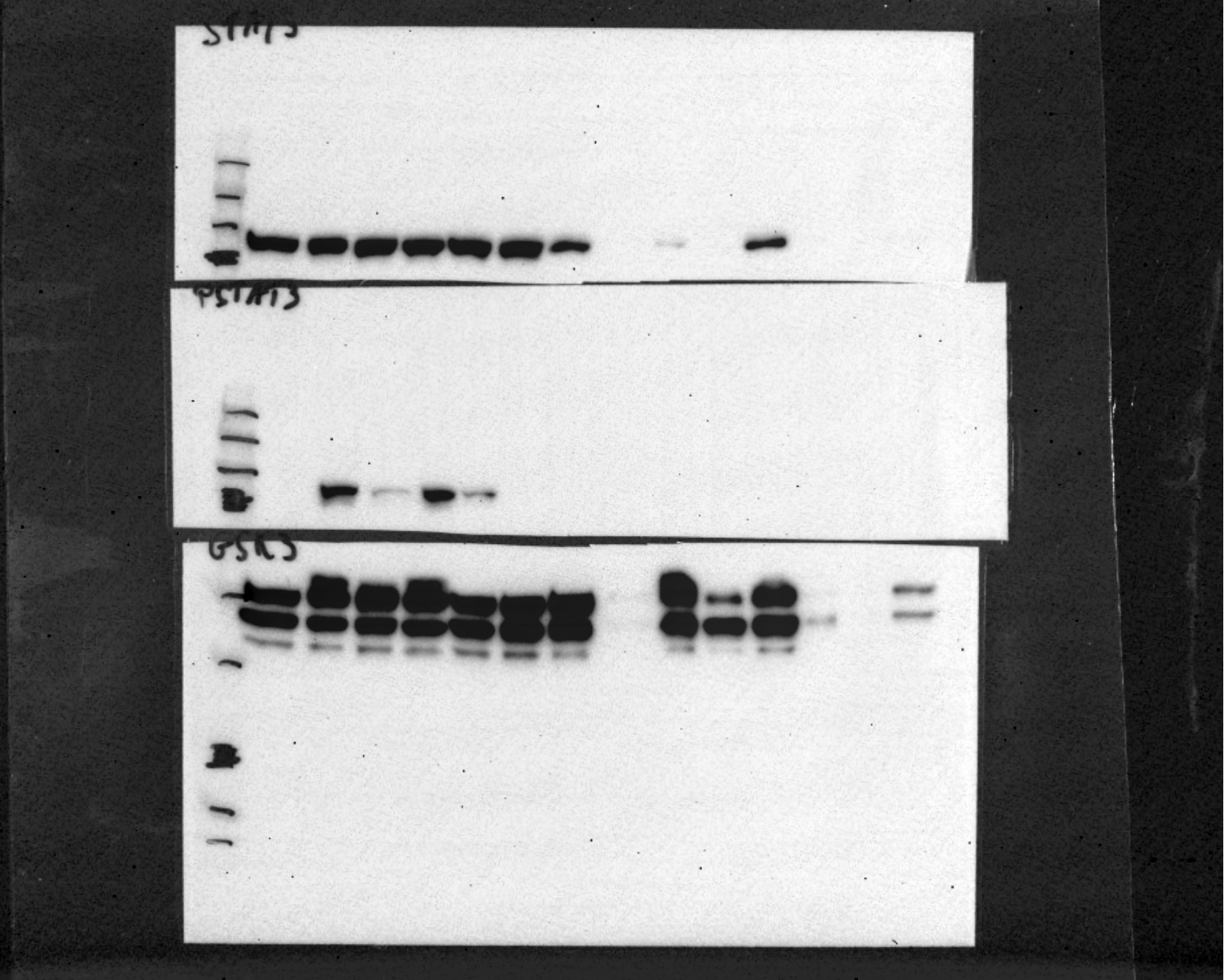

Supplement: Supplementary file 5 — Source data Fig. 3 [file 44319_2025_472_MOESM5_ESM.zip › Figure 3/3F/STAT3_memb_overlay_TOP.tif]

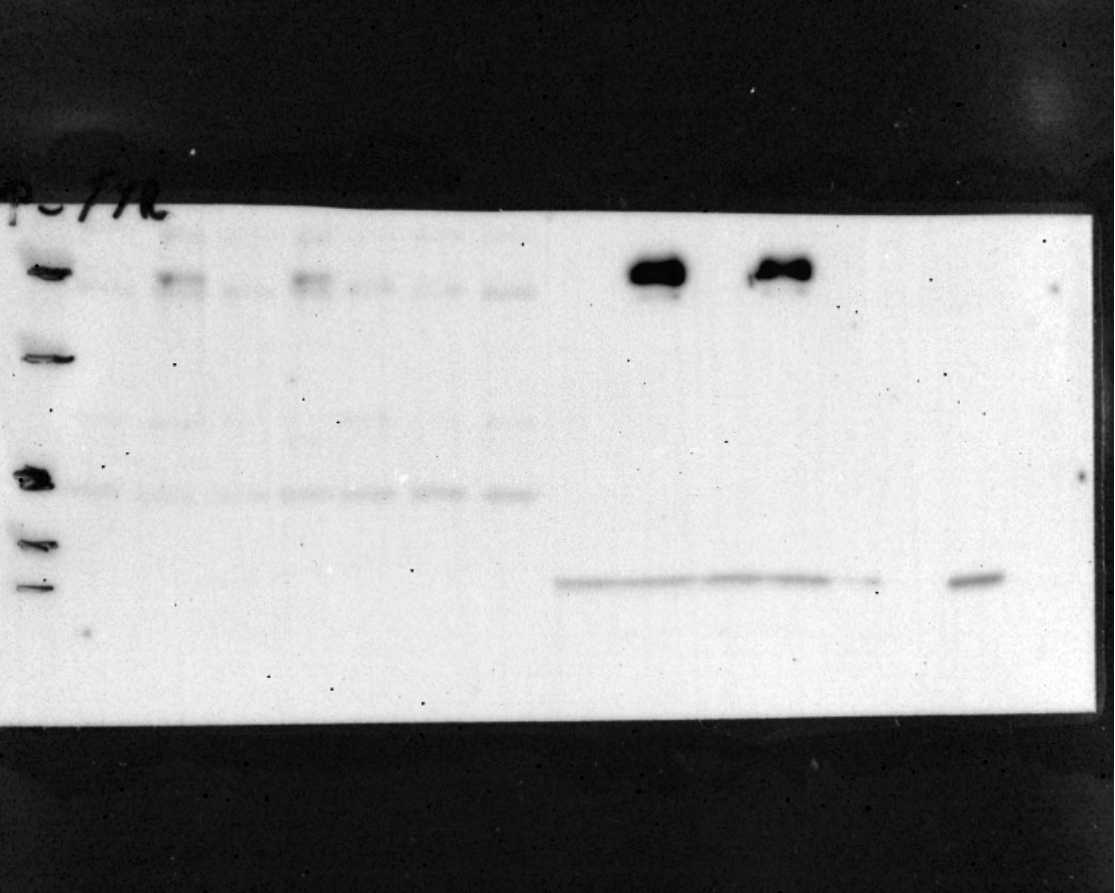

Supplement: Supplementary file 5 — Source data Fig. 3 [file 44319_2025_472_MOESM5_ESM.zip › Figure 3/3F/pTyrosine_memb_overlay.tif]

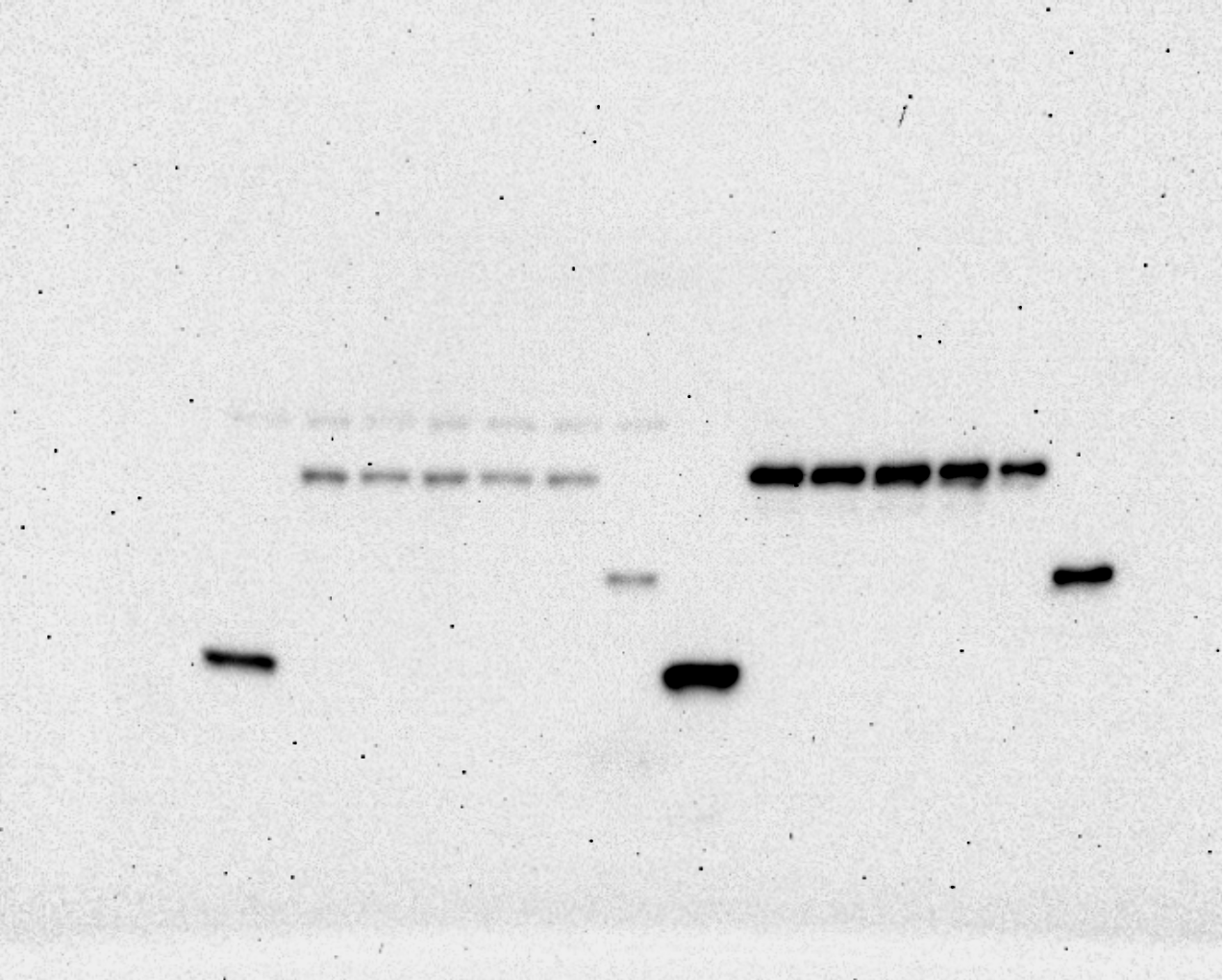

Supplement: Supplementary file 5 — Source data Fig. 3 [file 44319_2025_472_MOESM5_ESM.zip › Figure 3/3F/GFP.tif]

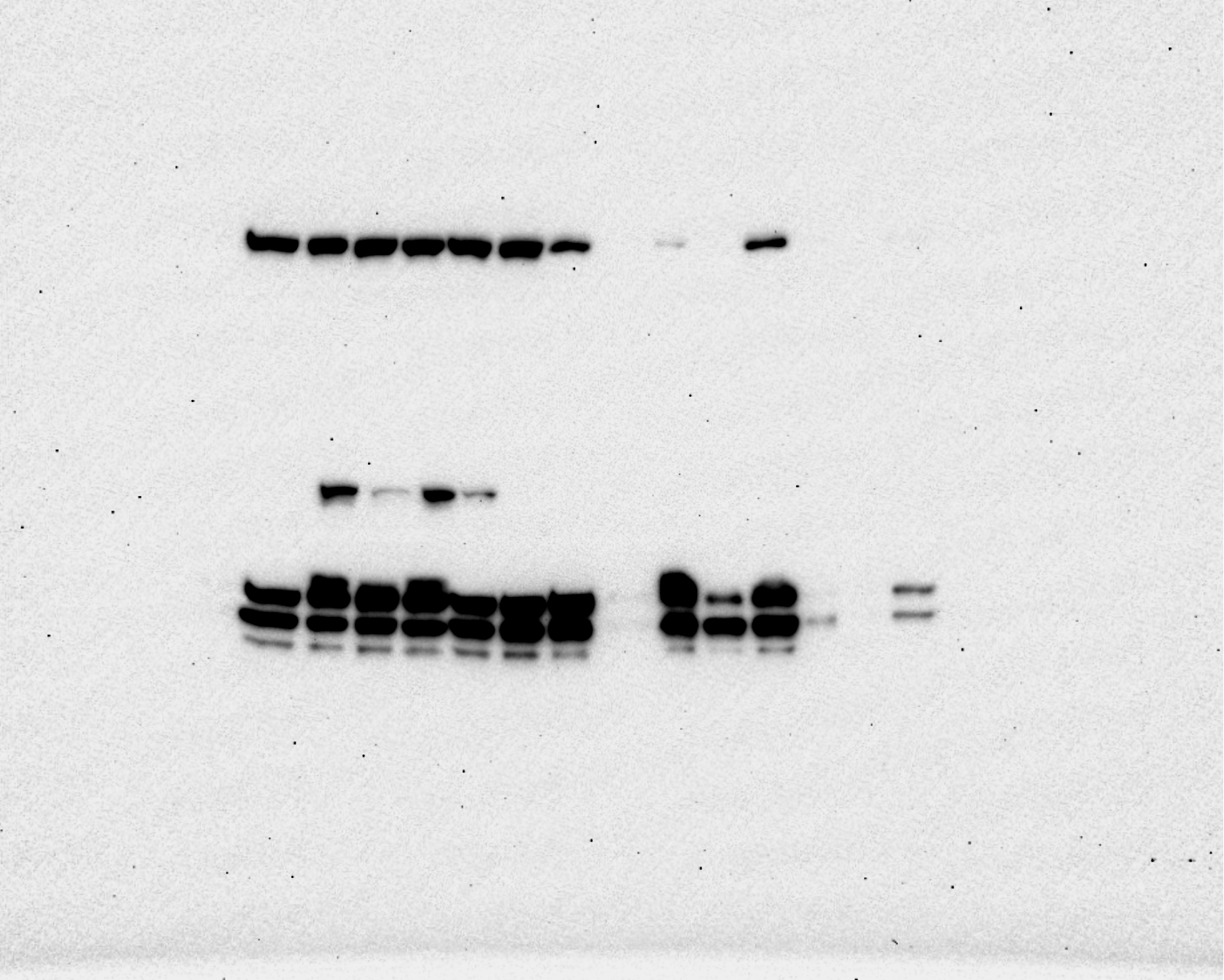

Supplement: Supplementary file 5 — Source data Fig. 3 [file 44319_2025_472_MOESM5_ESM.zip › Figure 3/3F/STAT3_top.tif]

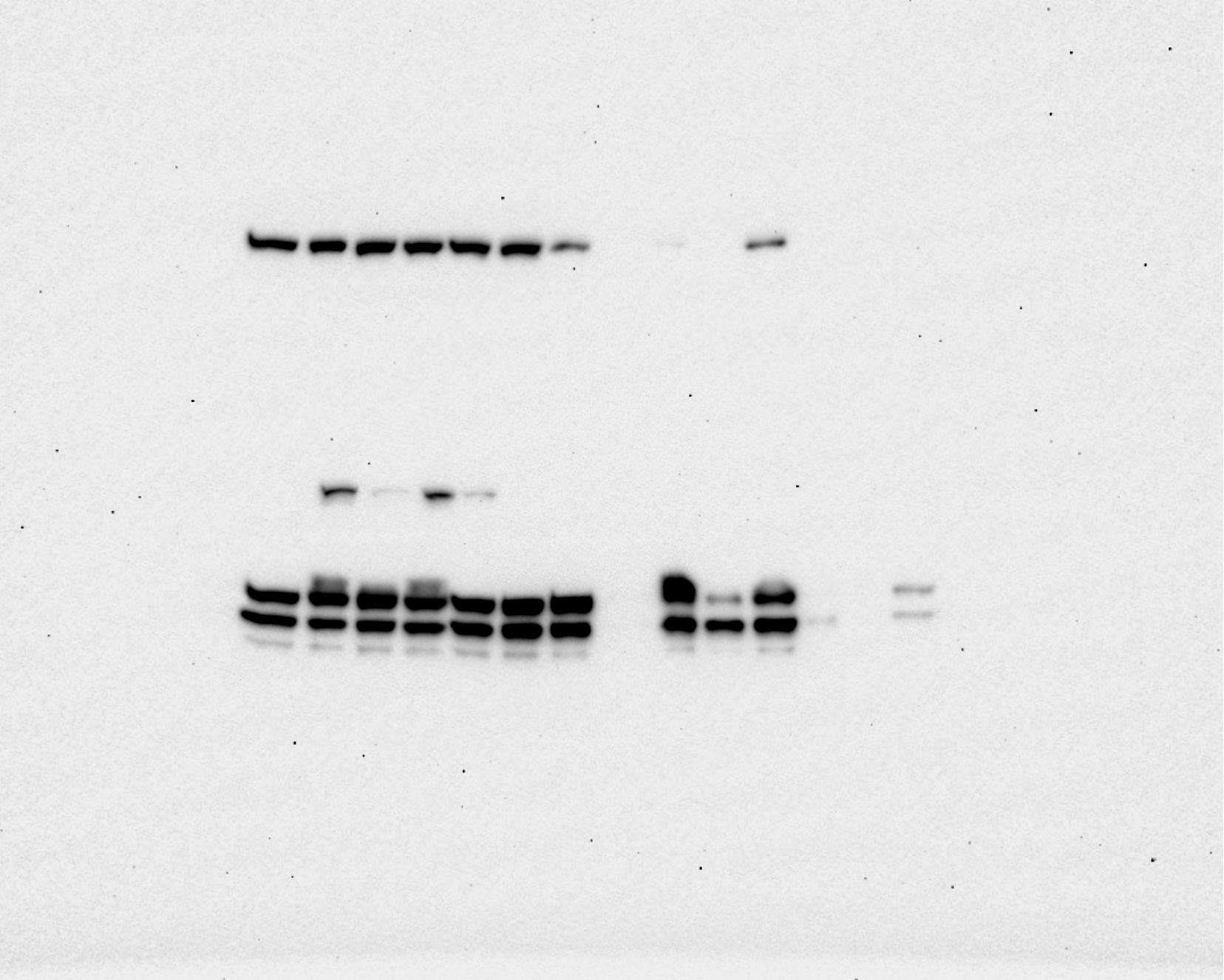

Supplement: Supplementary file 5 — Source data Fig. 3 [file 44319_2025_472_MOESM5_ESM.zip › Figure 3/3F/GSK3_lower.tif]

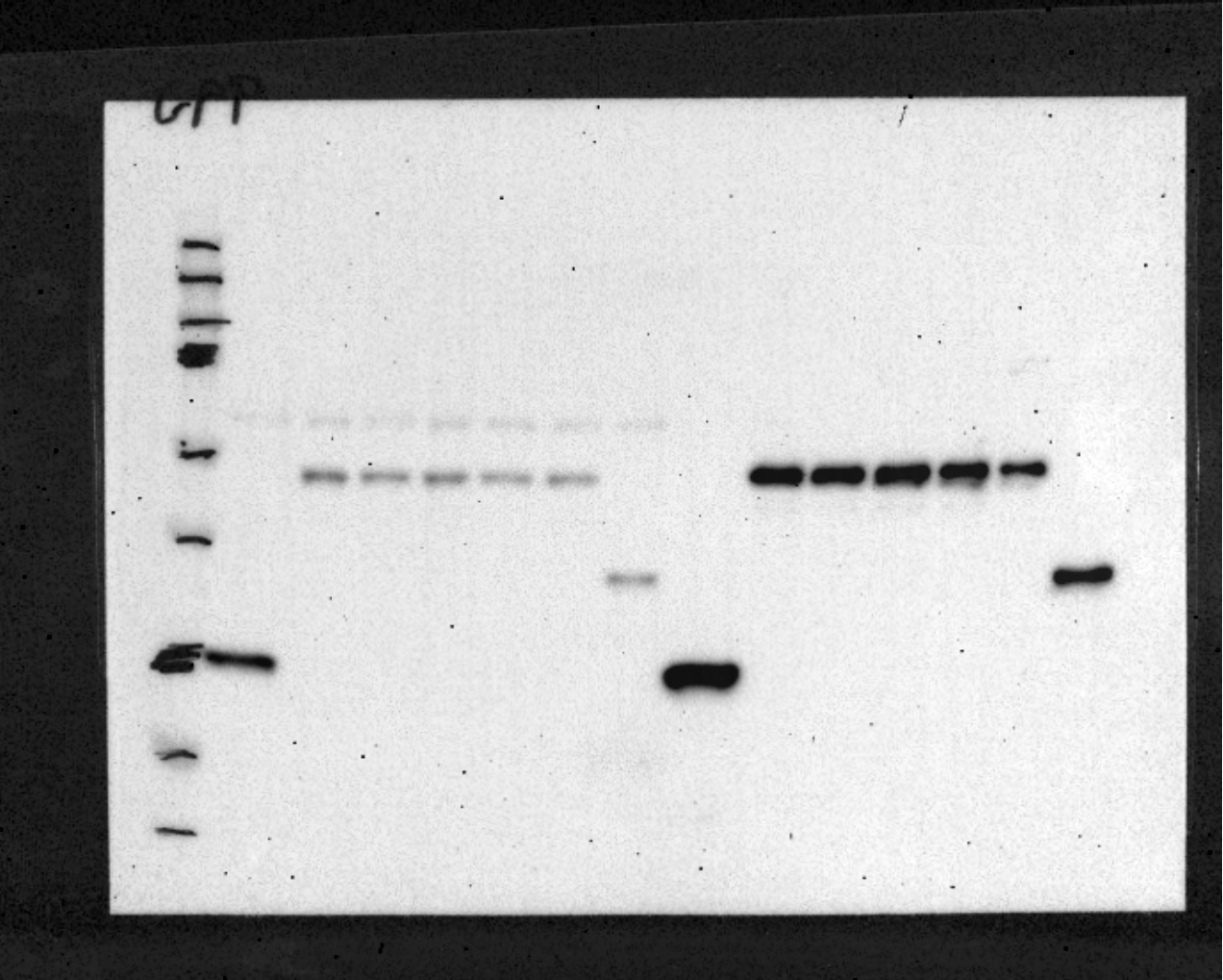

Supplement: Supplementary file 5 — Source data Fig. 3 [file 44319_2025_472_MOESM5_ESM.zip › Figure 3/3F/GFP_memb_overlay.tif]

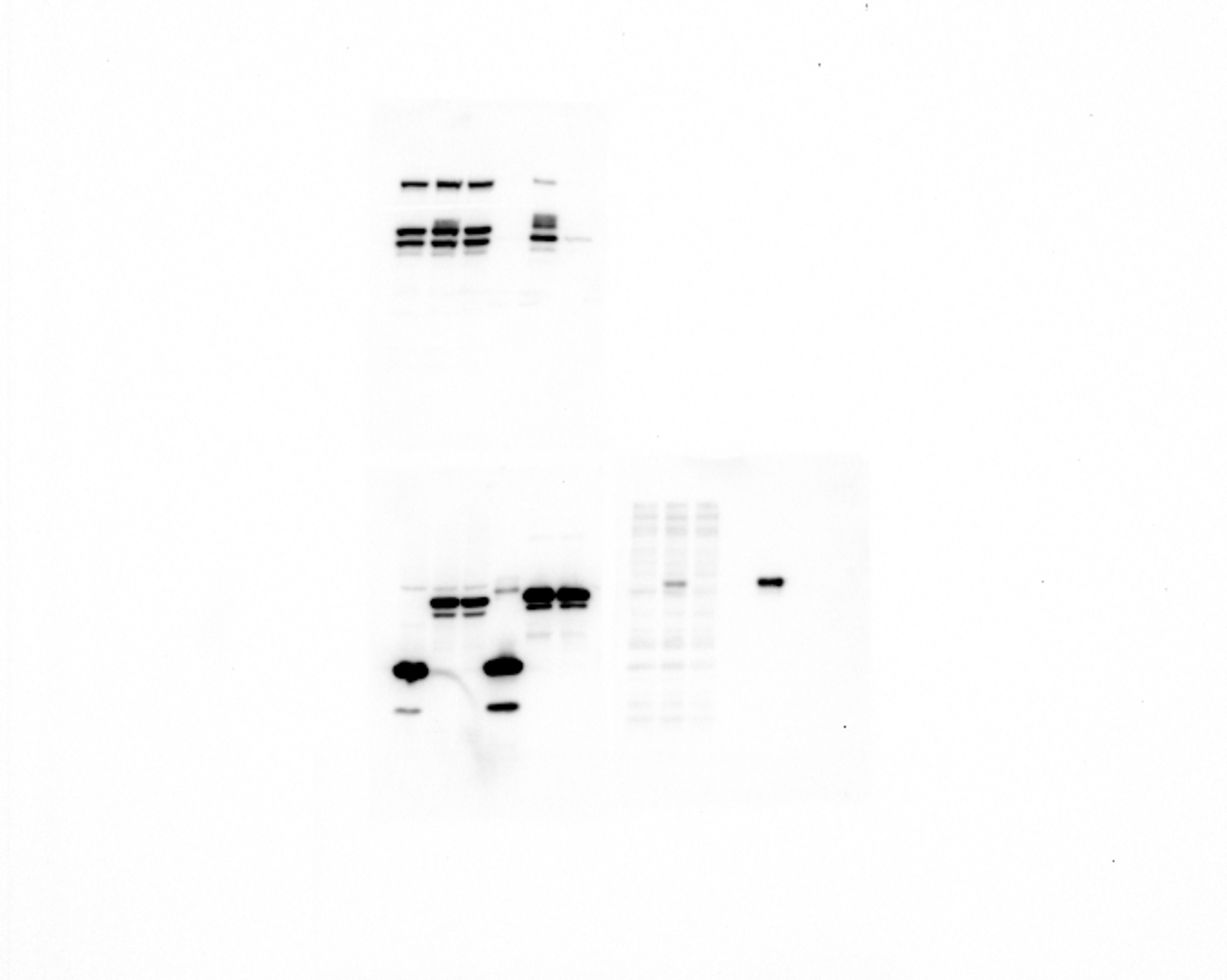

Supplement: Supplementary file 5 — Source data Fig. 3 [file 44319_2025_472_MOESM5_ESM.zip › Figure 3/3B/Ladder+STAT3_+GSK3+GFP+pTyr/LadderSTAT3_GSK3GFPpTyrChemi.tif]

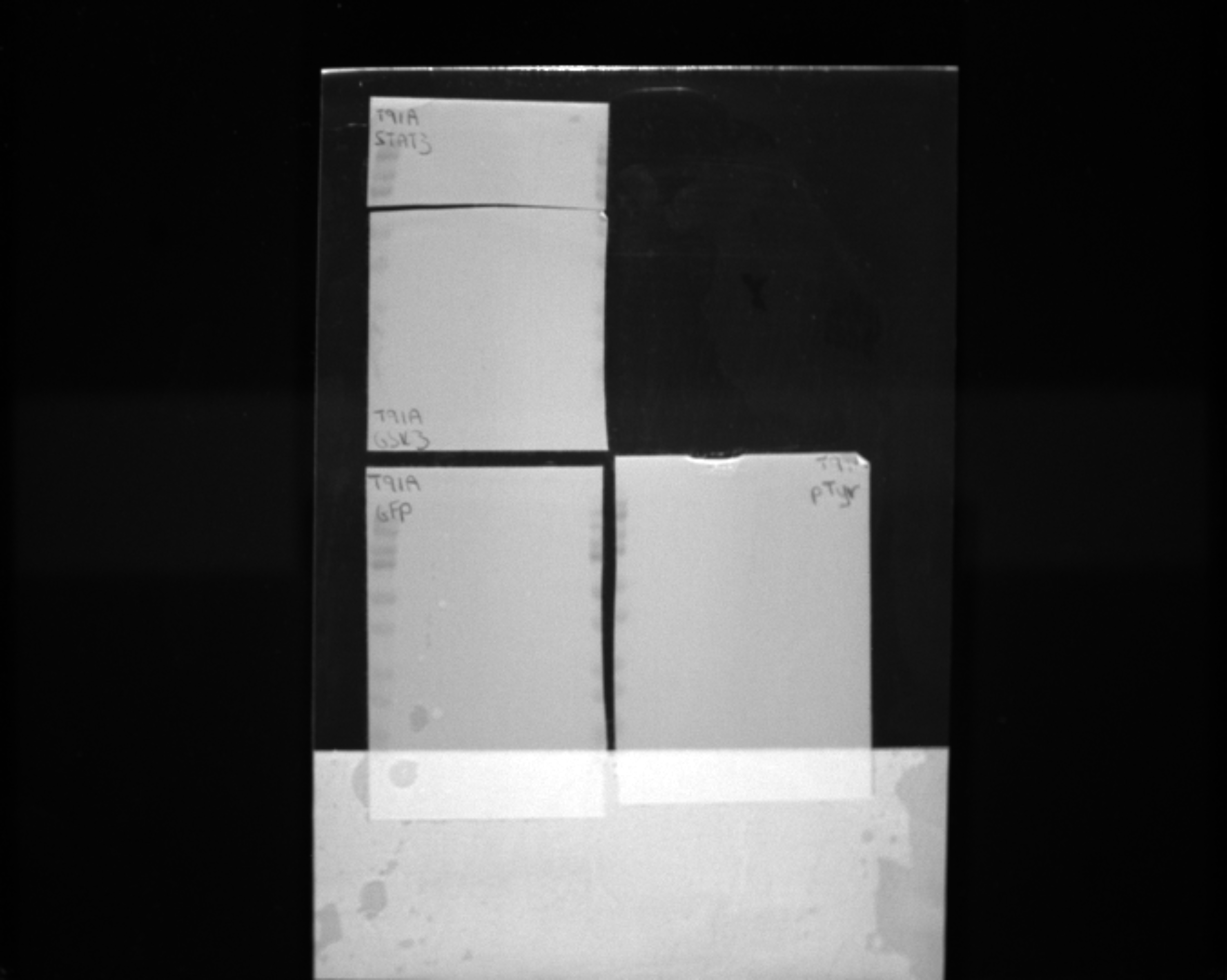

Supplement: Supplementary file 5 — Source data Fig. 3 [file 44319_2025_472_MOESM5_ESM.zip › Figure 3/3B/Ladder+STAT3_+GSK3+GFP+pTyr/LadderSTAT3_GSK3GFPpTyrMembrane.tif]

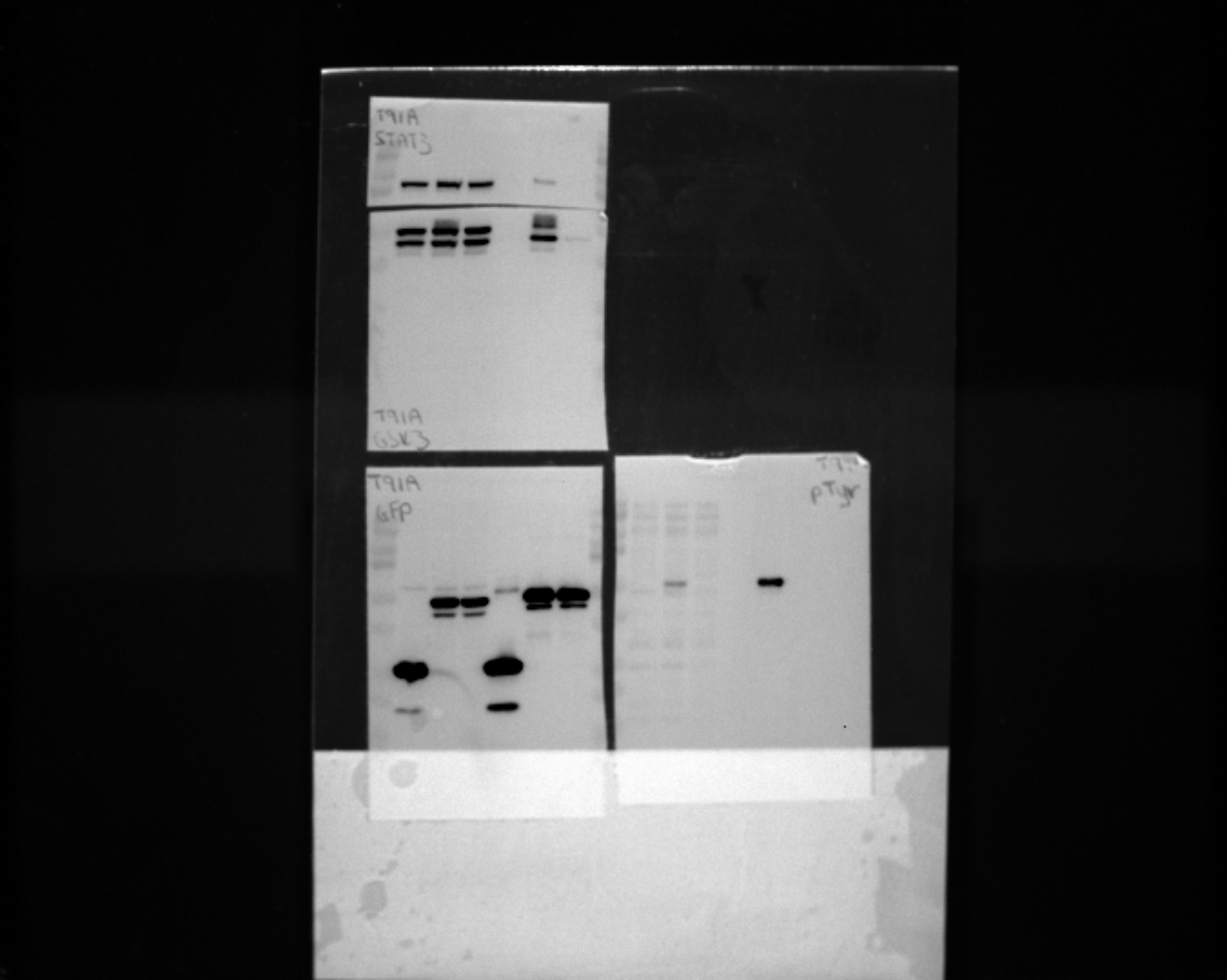

Supplement: Supplementary file 5 — Source data Fig. 3 [file 44319_2025_472_MOESM5_ESM.zip › Figure 3/3B/Ladder+STAT3_+GSK3+GFP+pTyr/LadderSTAT3_GSK3GFPpTyr_composite.tif]

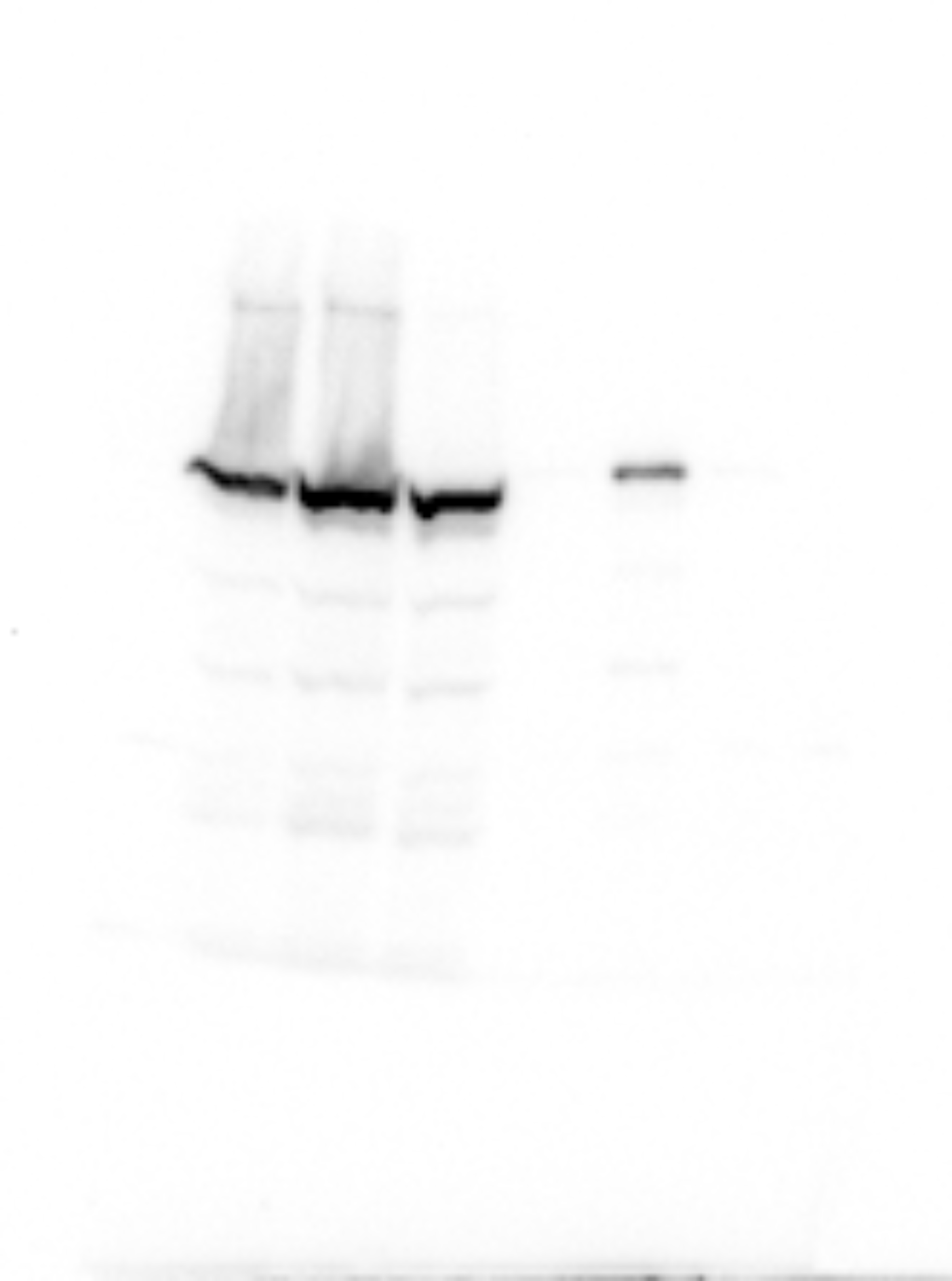

Supplement: Supplementary file 5 — Source data Fig. 3 [file 44319_2025_472_MOESM5_ESM.zip › Figure 3/3H/Ladder+STAT3/LadderSTAT3Chemi.tif]

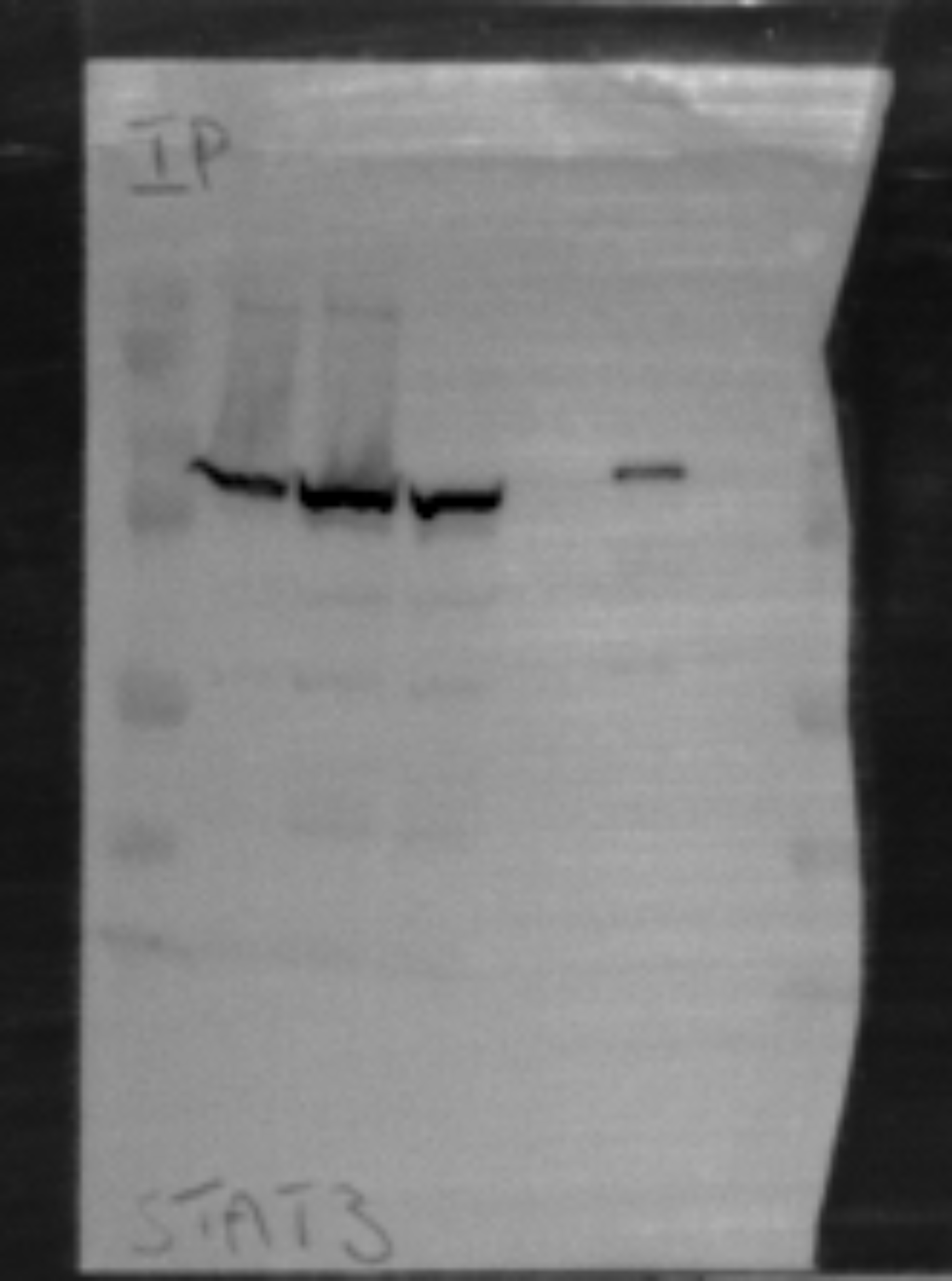

Supplement: Supplementary file 5 — Source data Fig. 3 [file 44319_2025_472_MOESM5_ESM.zip › Figure 3/3H/Ladder+STAT3/LadderSTAT3_composite.tif]

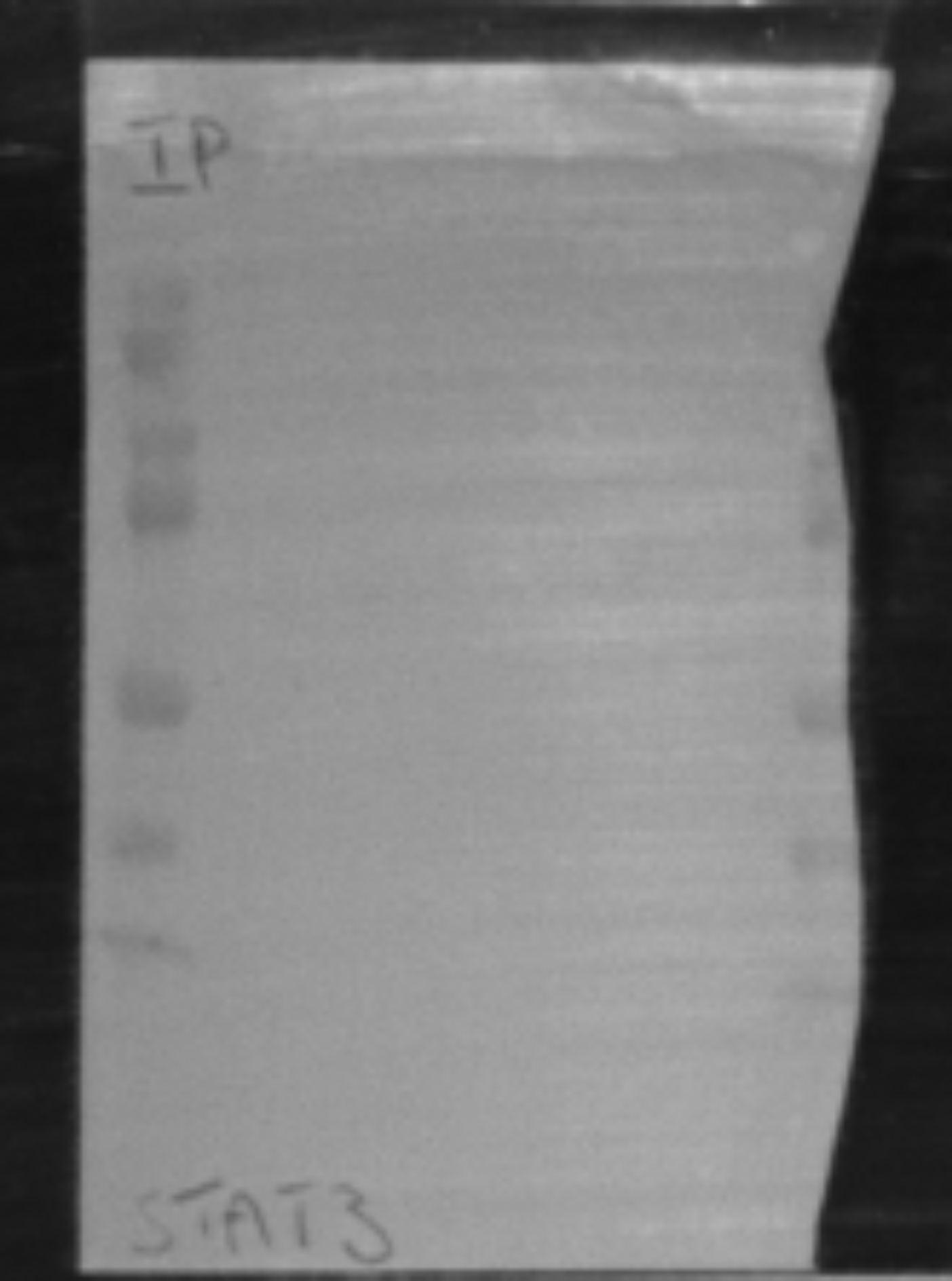

Supplement: Supplementary file 5 — Source data Fig. 3 [file 44319_2025_472_MOESM5_ESM.zip › Figure 3/3H/Ladder+STAT3/LadderSTAT3Membrane.tif]

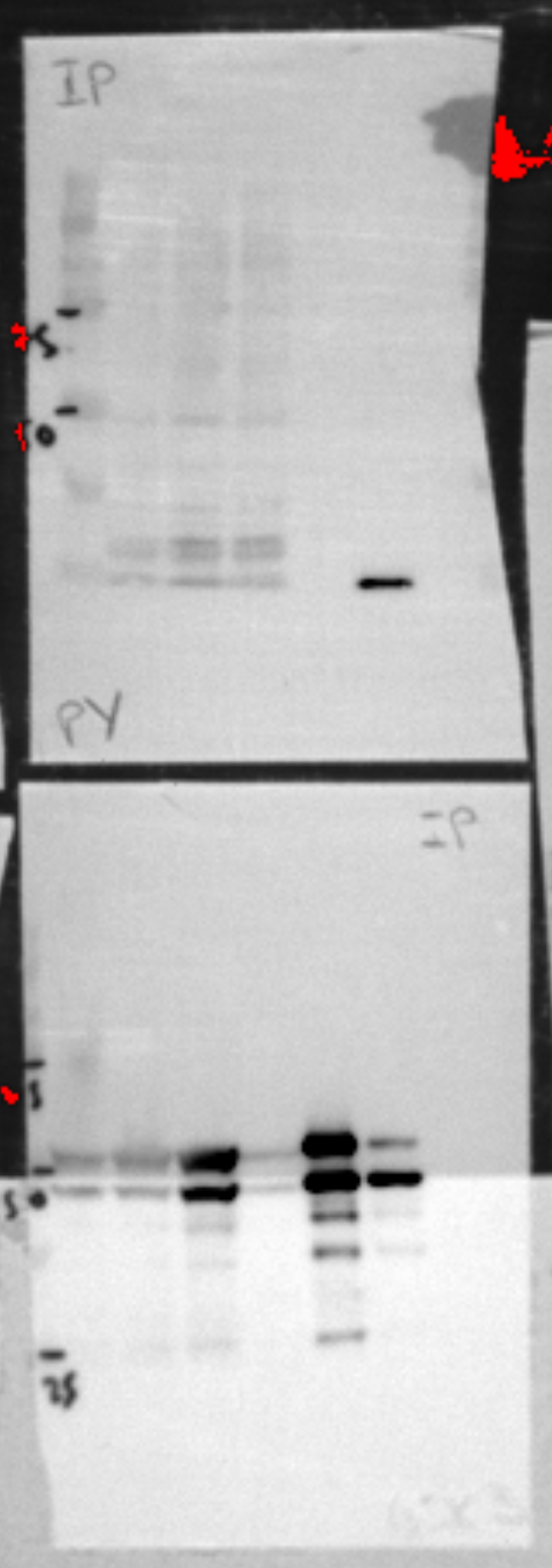

Supplement: Supplementary file 5 — Source data Fig. 3 [file 44319_2025_472_MOESM5_ESM.zip › Figure 3/3H/Ladder+pTyr_top_right/LadderGSK3_lower_right_composite.tif]

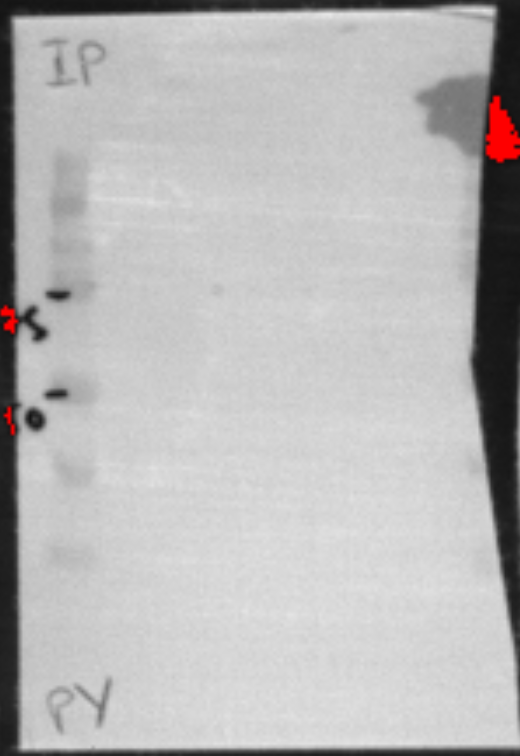

Supplement: Supplementary file 5 — Source data Fig. 3 [file 44319_2025_472_MOESM5_ESM.zip › Figure 3/3H/Ladder+pTyr_top_right/LadderGSK3_lower_rightMembrane.tif]

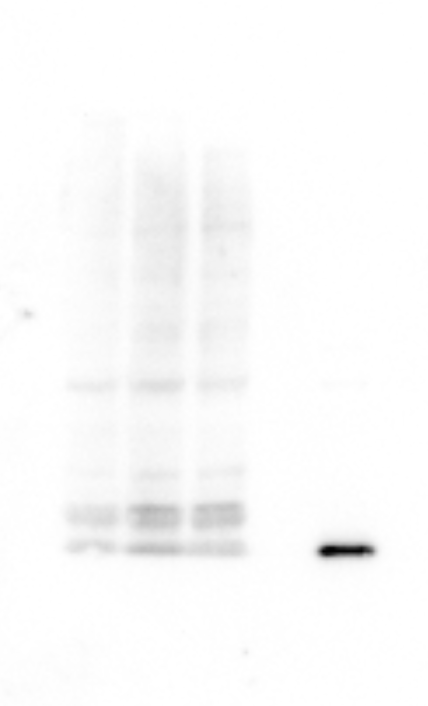

Supplement: Supplementary file 5 — Source data Fig. 3 [file 44319_2025_472_MOESM5_ESM.zip › Figure 3/3H/Ladder+pTyr_top_right/LadderGSK3_lower_rightChemi.tif]

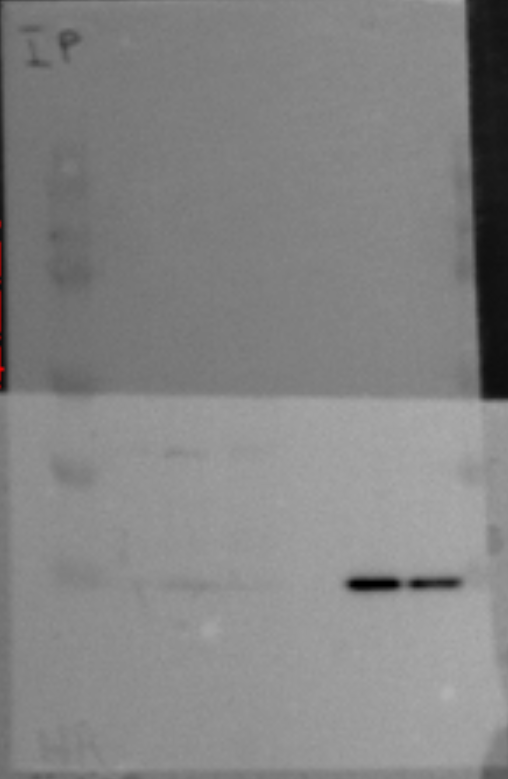

Supplement: Supplementary file 5 — Source data Fig. 3 [file 44319_2025_472_MOESM5_ESM.zip › Figure 3/3H/Ladder+HA/LadderHA_composite.tif]

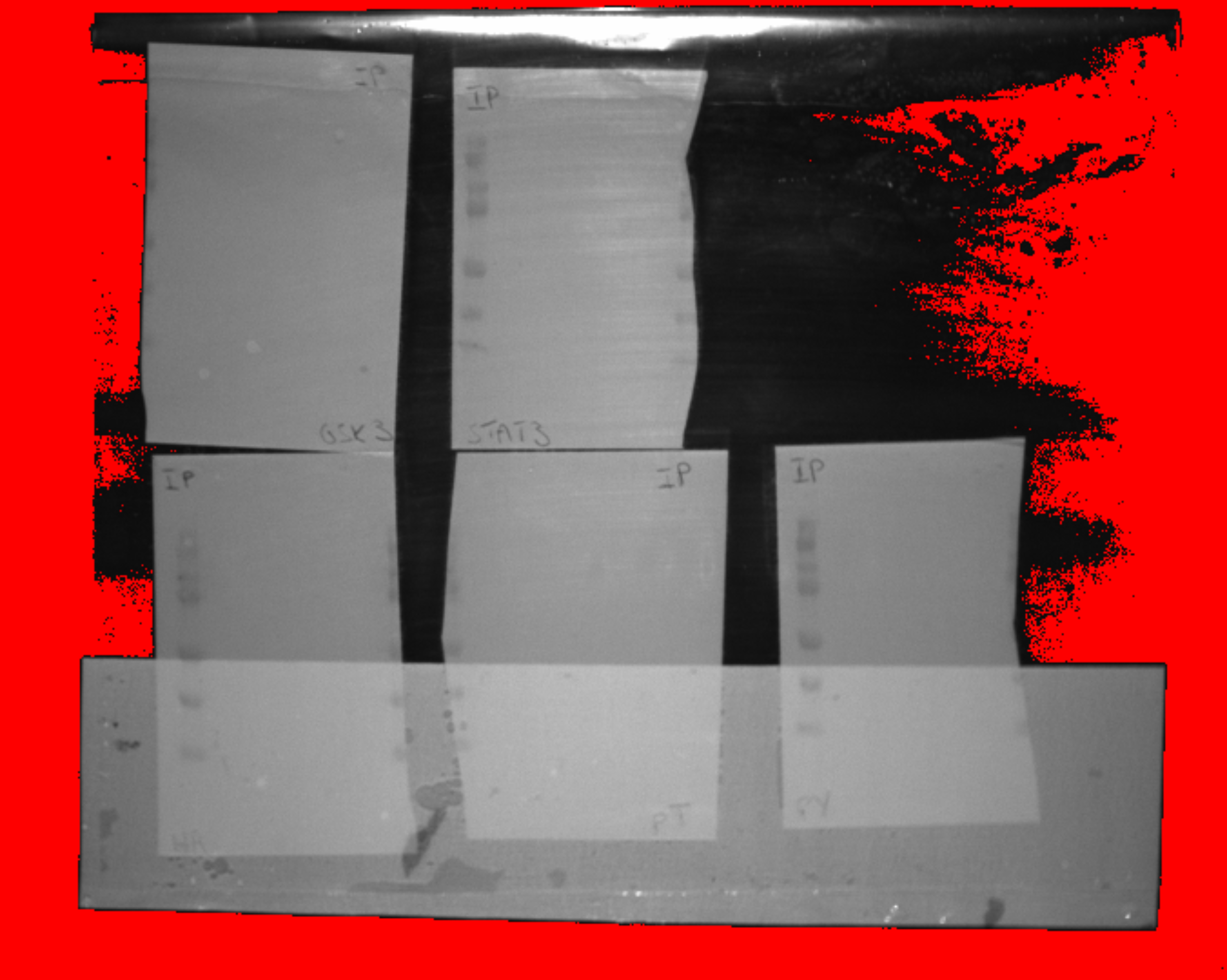

Supplement: Supplementary file 5 — Source data Fig. 3 [file 44319_2025_472_MOESM5_ESM.zip › Figure 3/3H/Ladder+HA/LadderHAMembrane.tif]

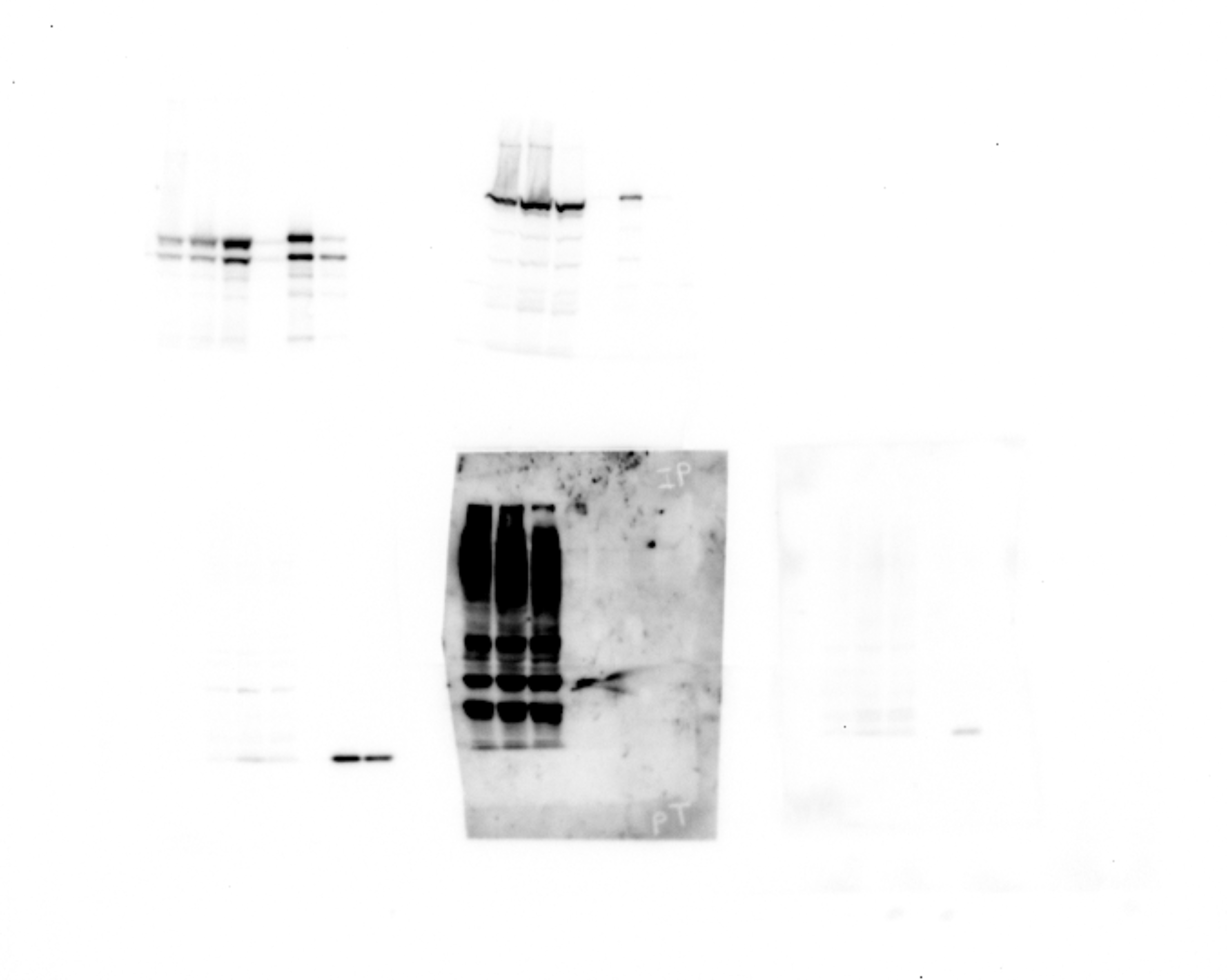

Supplement: Supplementary file 5 — Source data Fig. 3 [file 44319_2025_472_MOESM5_ESM.zip › Figure 3/3H/Ladder+HA/LadderHAChemi.tif]

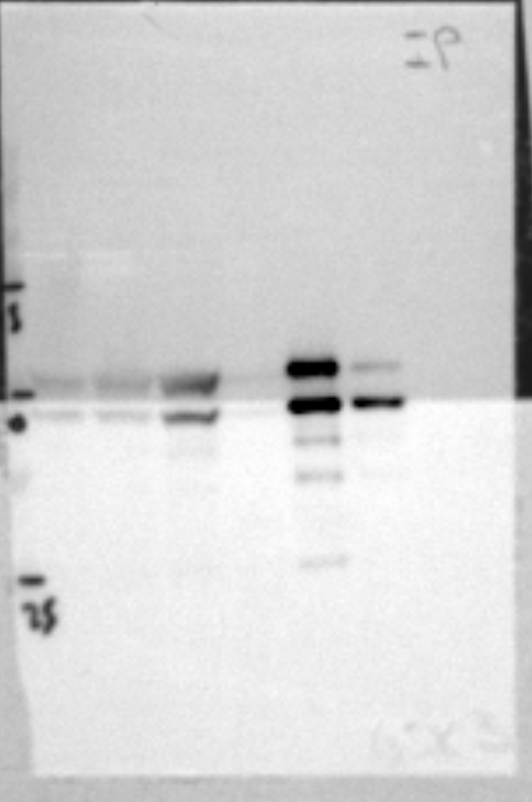

Supplement: Supplementary file 5 — Source data Fig. 3 [file 44319_2025_472_MOESM5_ESM.zip › Figure 3/3H/Ladder+GSK3_lower_right/LadderGSK3_lower_right_composite.tif]

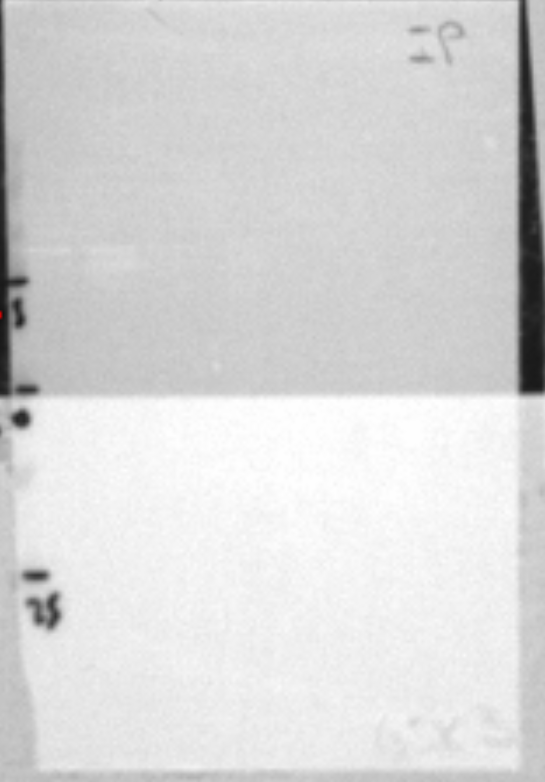

Supplement: Supplementary file 5 — Source data Fig. 3 [file 44319_2025_472_MOESM5_ESM.zip › Figure 3/3H/Ladder+GSK3_lower_right/LadderGSK3_lower_rightMembrane.tif]

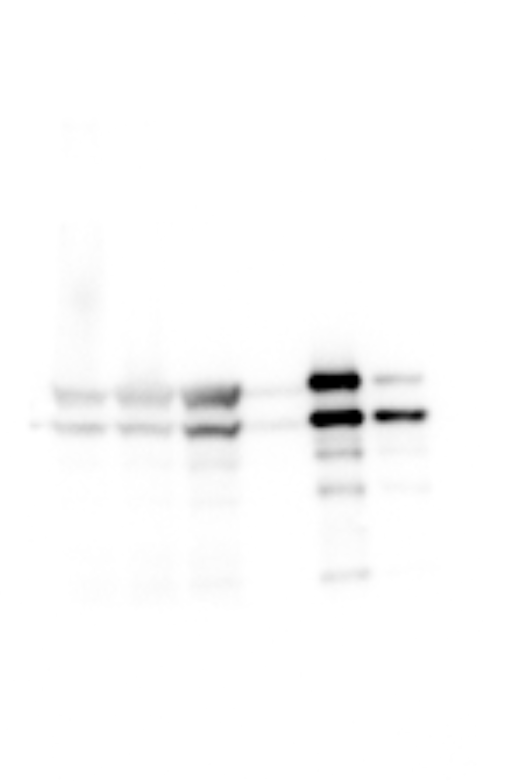

Supplement: Supplementary file 5 — Source data Fig. 3 [file 44319_2025_472_MOESM5_ESM.zip › Figure 3/3H/Ladder+GSK3_lower_right/LadderGSK3_lower_rightChemi.tif]

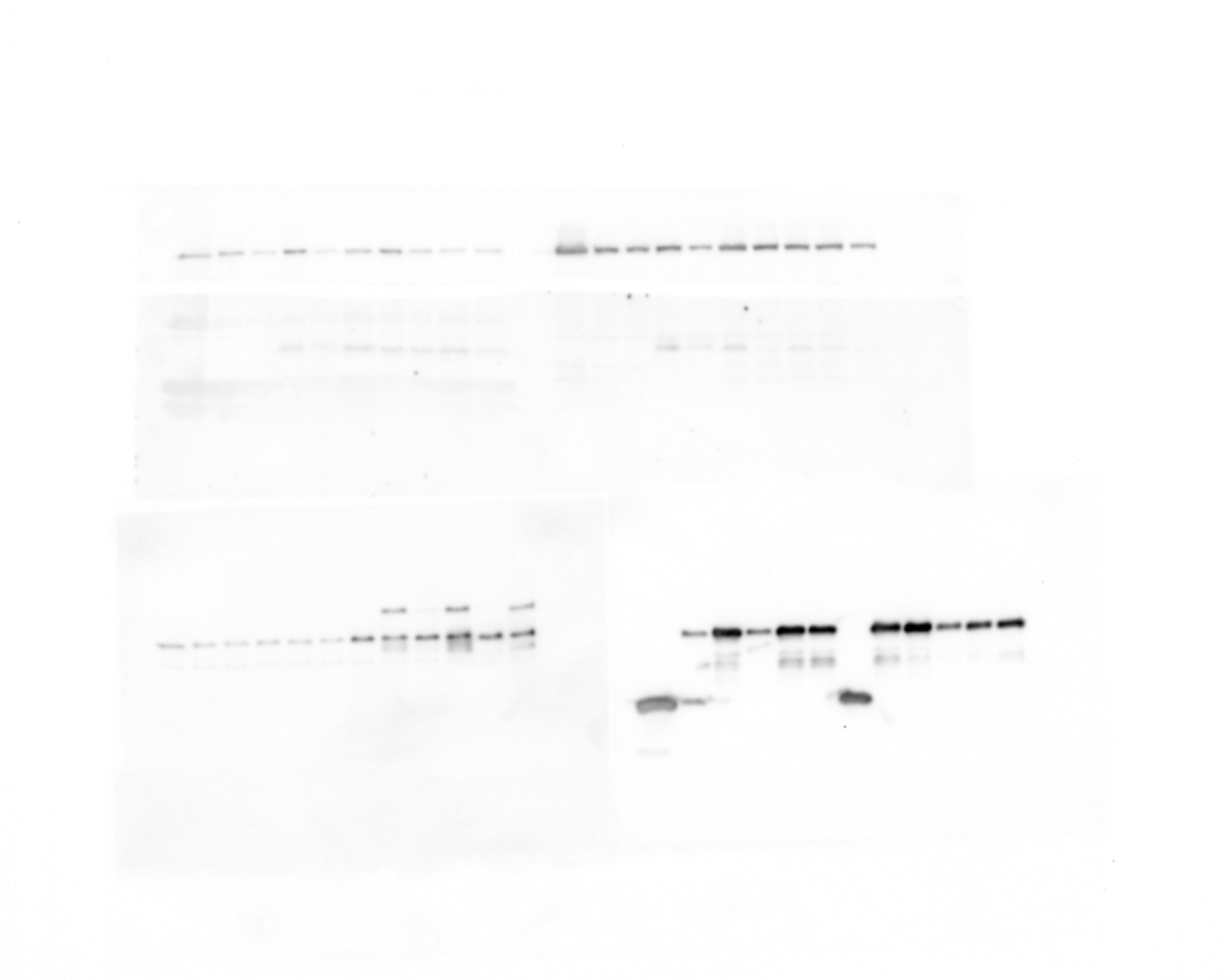

Supplement: Supplementary file 5 — Source data Fig. 3 [file 44319_2025_472_MOESM5_ESM.zip › Figure 3/3G/Ladder+GFP_bottom_right/LadderGFP_bottom_rightChemi.tif]

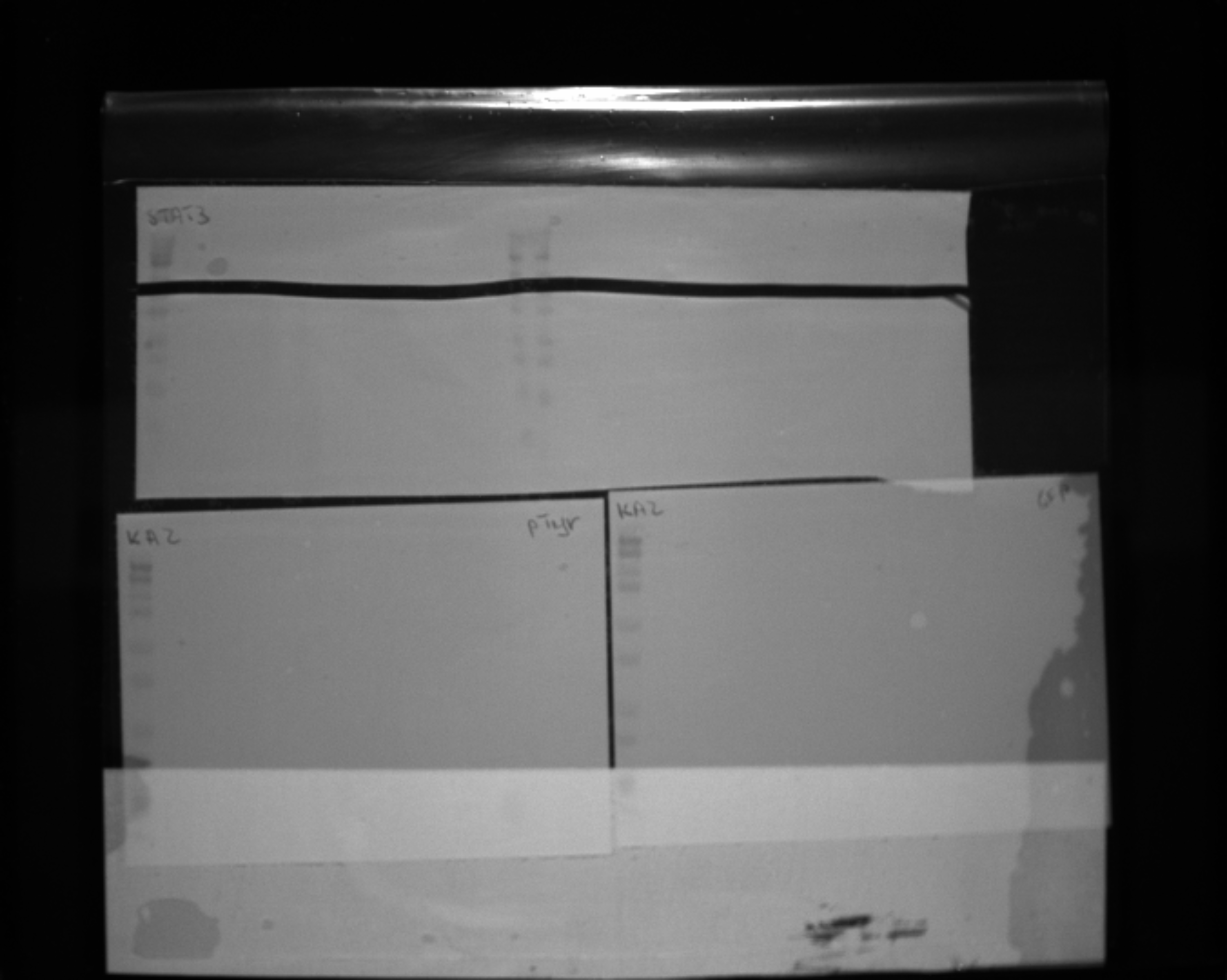

Supplement: Supplementary file 5 — Source data Fig. 3 [file 44319_2025_472_MOESM5_ESM.zip › Figure 3/3G/Ladder+GFP_bottom_right/LadderGFP_bottom_rightMembrane.tif]

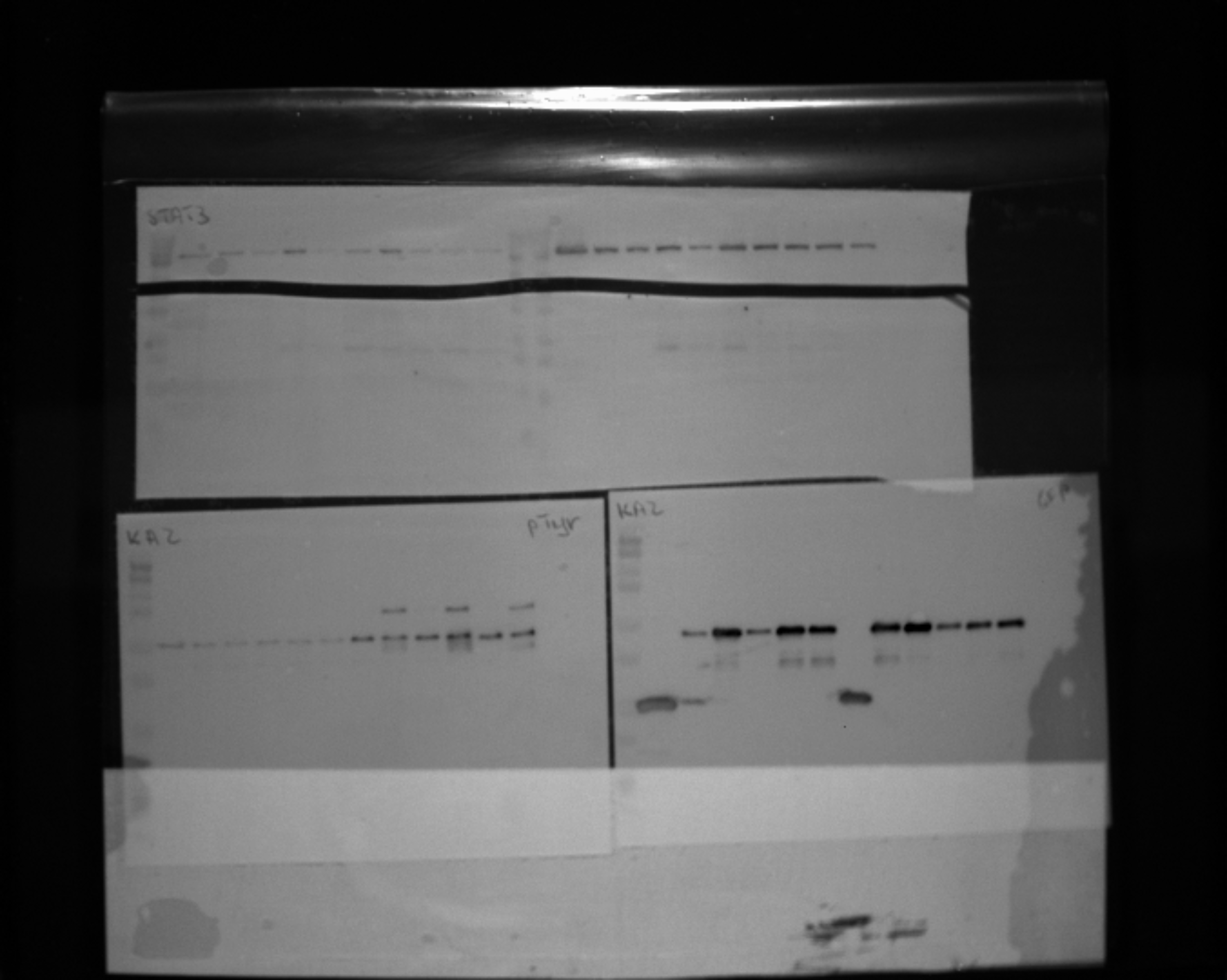

Supplement: Supplementary file 5 — Source data Fig. 3 [file 44319_2025_472_MOESM5_ESM.zip › Figure 3/3G/Ladder+GFP_bottom_right/LadderGFP_bottom_right_composite.tif]
