# Supplementary material for: Bacterial effectors mediate kinase reprogramming through mimicry of conserved eukaryotic motifs
Source: EMBO Rep. 2025 May 12;26(14):3529–53. doi: 10.1038/s44319-025-00472-y (PMC12287357; doi:10.1038/s44319-025-00472-y)
Supplement: Supplementary file 4 — Source data Fig. 2 [file 44319_2025_472_MOESM4_ESM.zip › Figure 2/2F/2F_readme.pptx]

## Slide 1
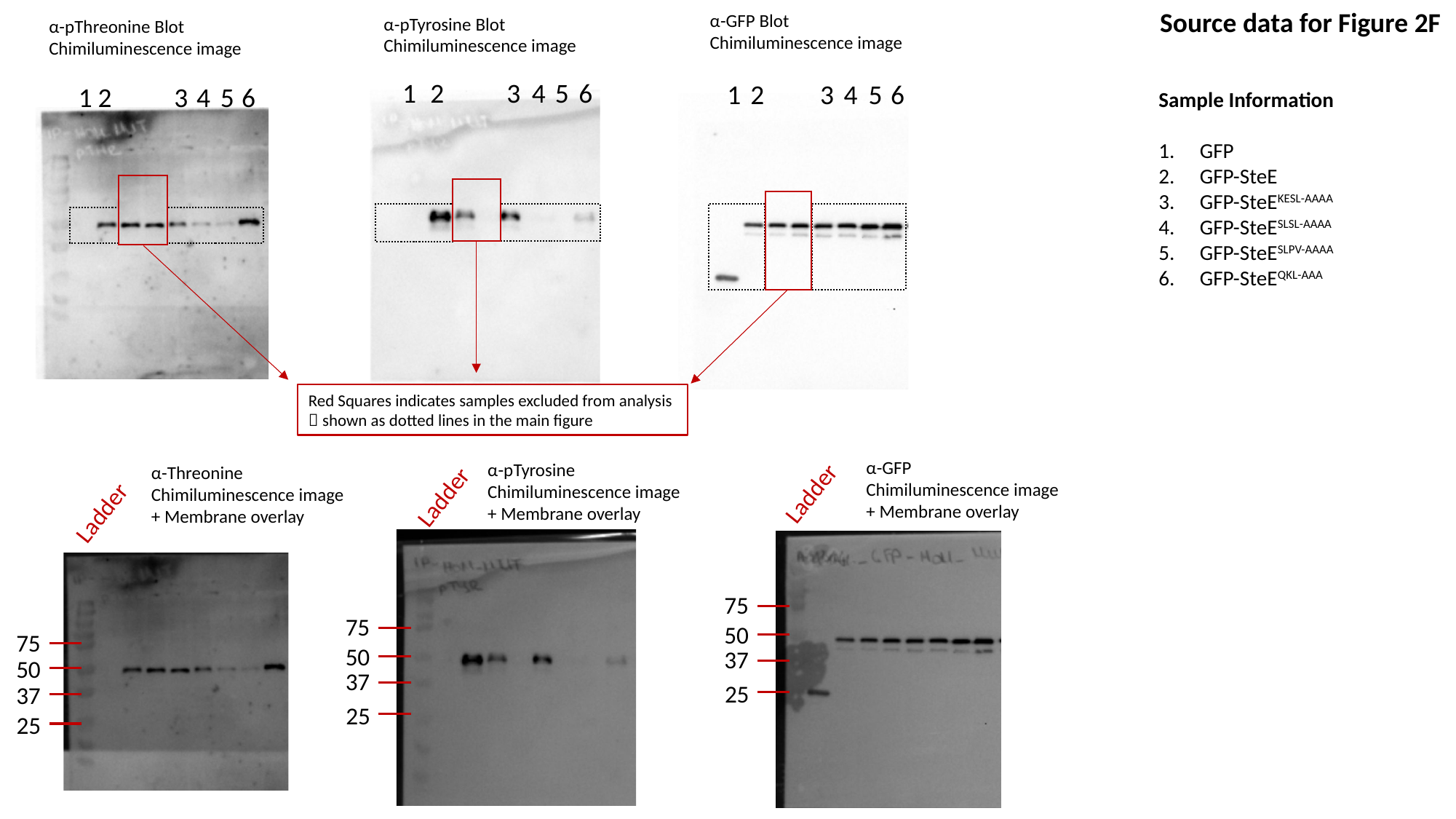

Source data for Figure 2F
α-GFP Blot
Chimiluminescence image
2
3
4
5
6
1
α-pTyrosine Blot
Chimiluminescence image
2
3
4
5
6
1
α-pThreonine Blot
Chimiluminescence image
1
2
3
4
5
6
Red Squares indicates samples excluded from analysis
 shown as dotted lines in the main figure
Sample Information
GFP
GFP-SteE
GFP-SteEKESL-AAAA
GFP-SteESLSL-AAAA
GFP-SteESLPV-AAAA
GFP-SteEQKL-AAA
α-GFP
Chimiluminescence image
+ Membrane overlay
Ladder
75
50
37
25
α-pTyrosine
Chimiluminescence image
+ Membrane overlay
Ladder
50
37
75
25
α-Threonine
Chimiluminescence image
+ Membrane overlay
Ladder
75
50
37
25
