# Supplementary material for: Bacterial effectors mediate kinase reprogramming through mimicry of conserved eukaryotic motifs
Source: EMBO Rep. 2025 May 12;26(14):3529–53. doi: 10.1038/s44319-025-00472-y (PMC12287357; doi:10.1038/s44319-025-00472-y)
Supplement: Supplementary file 4 — Source data Fig. 2 [file 44319_2025_472_MOESM4_ESM.zip › Figure 2/2C/2C_readme.pptx]

## Slide 1
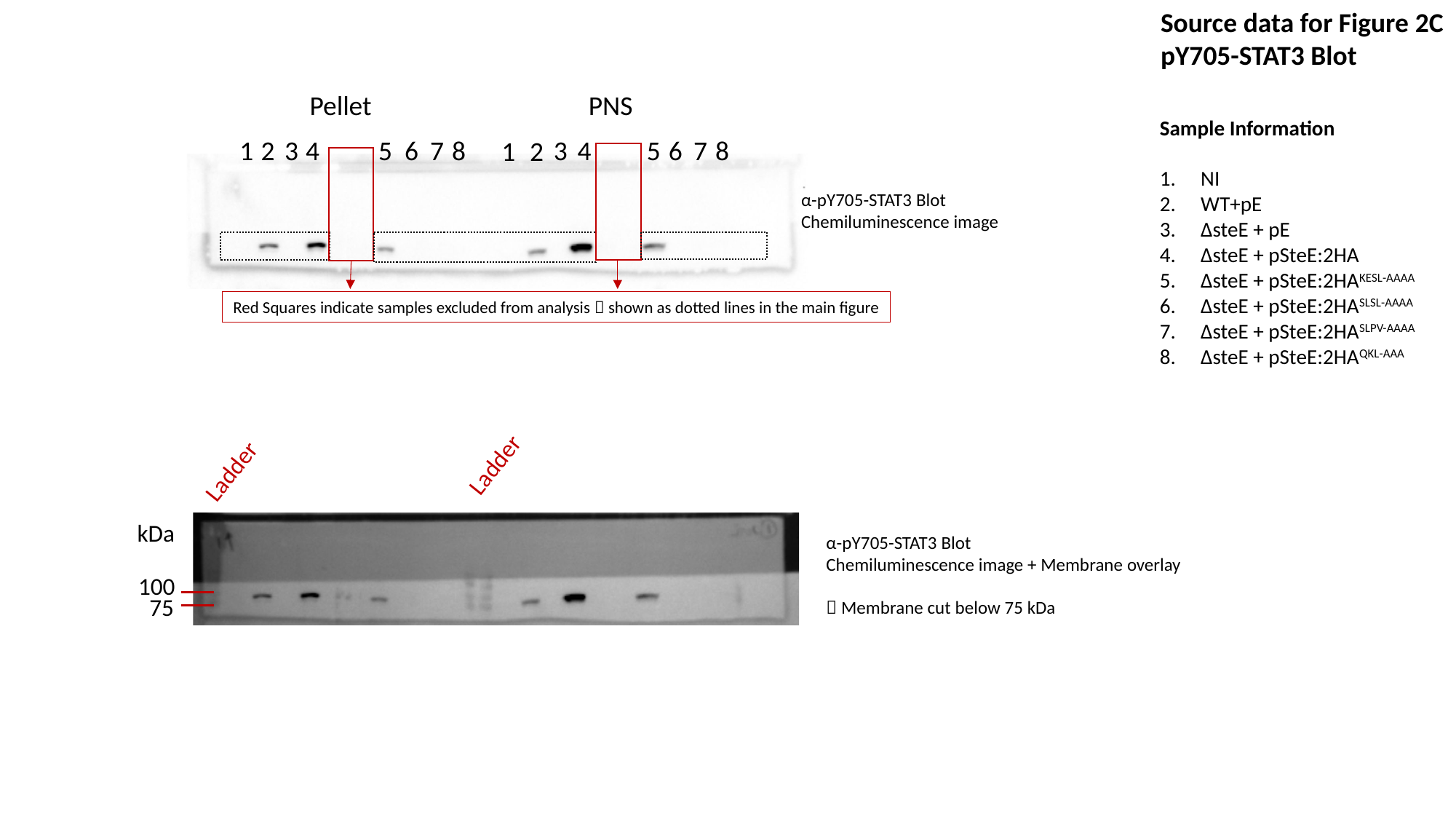

Source data for Figure 2C
pY705-STAT3 Blot
Pellet
PNS
1
2
3
4
5
6
3
4
5
6
2
1
α-pY705-STAT3 Blot
Chemiluminescence image
Sample Information
NI
WT+pE
ΔsteE + pE
ΔsteE + pSteE:2HA
ΔsteE + pSteE:2HAKESL-AAAA
ΔsteE + pSteE:2HASLSL-AAAA
ΔsteE + pSteE:2HASLPV-AAAA
ΔsteE + pSteE:2HAQKL-AAA
7
8
7
8
Red Squares indicate samples excluded from analysis  shown as dotted lines in the main figure
Ladder
kDa
α-pY705-STAT3 Blot
Chemiluminescence image + Membrane overlay
 Membrane cut below 75 kDa
100
75
Ladder

## Slide 2
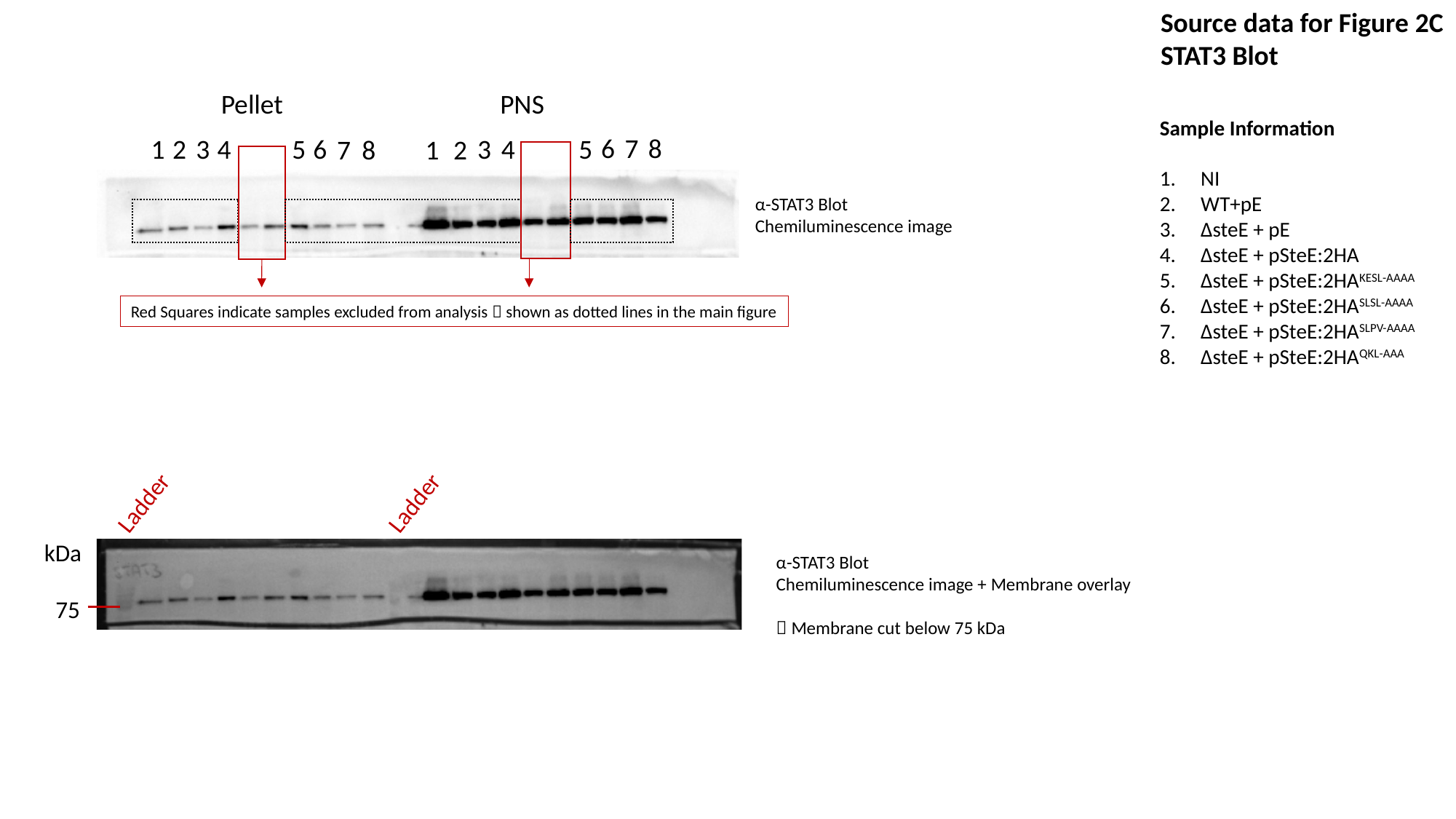

Source data for Figure 2C
STAT3 Blot
Pellet
PNS
6
1
2
3
4
5
6
3
4
5
2
1
α-STAT3 Blot
Chemiluminescence image
8
7
7
8
Sample Information
NI
WT+pE
ΔsteE + pE
ΔsteE + pSteE:2HA
ΔsteE + pSteE:2HAKESL-AAAA
ΔsteE + pSteE:2HASLSL-AAAA
ΔsteE + pSteE:2HASLPV-AAAA
ΔsteE + pSteE:2HAQKL-AAA
Red Squares indicate samples excluded from analysis  shown as dotted lines in the main figure
Ladder
kDa
α-STAT3 Blot
Chemiluminescence image + Membrane overlay
 Membrane cut below 75 kDa
75
Ladder

## Slide 3
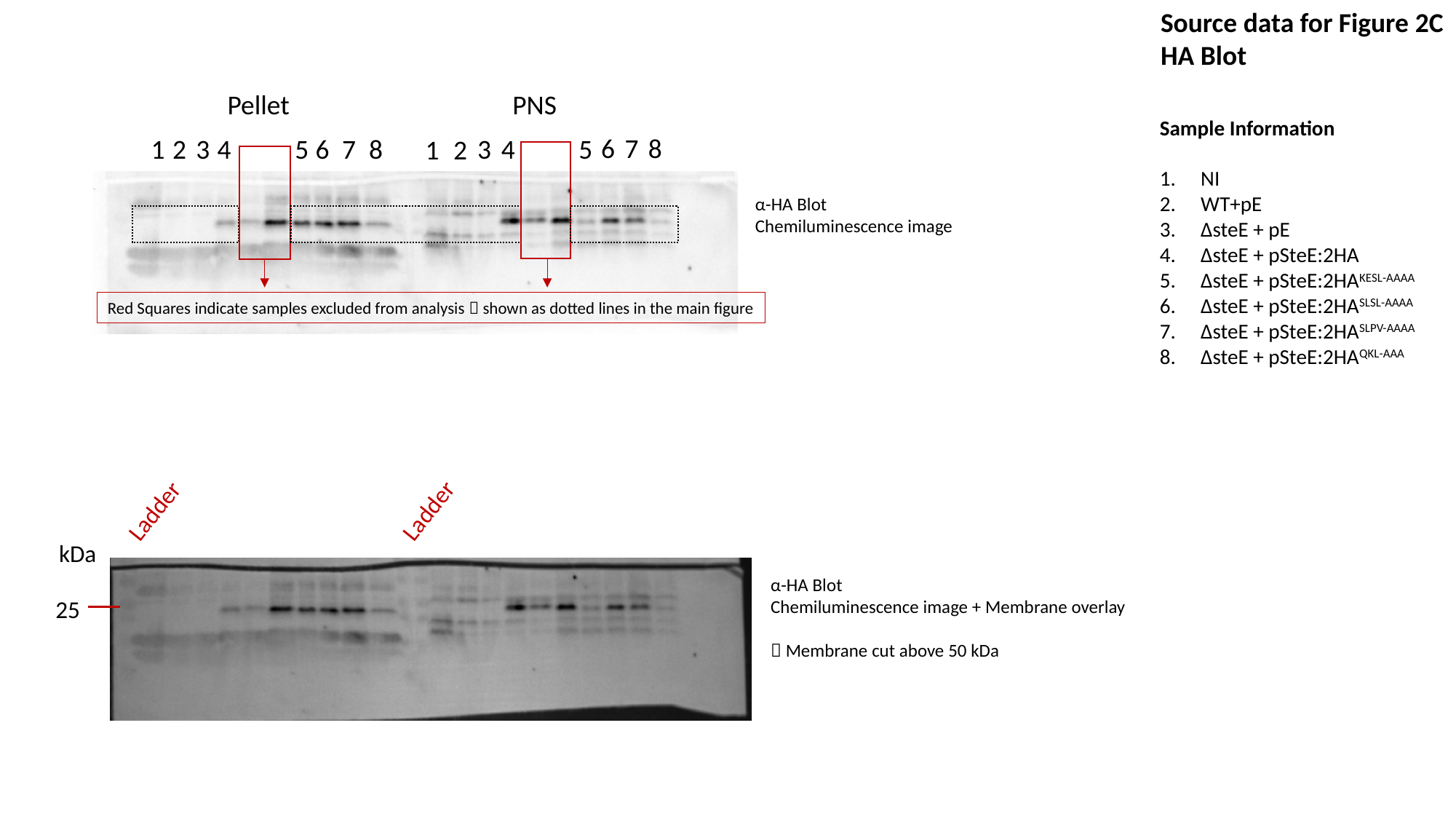

Source data for Figure 2C
HA Blot
Pellet
PNS
6
1
2
3
4
5
6
3
4
5
2
1
α-HA Blot
Chemiluminescence image
8
7
7
8
Sample Information
NI
WT+pE
ΔsteE + pE
ΔsteE + pSteE:2HA
ΔsteE + pSteE:2HAKESL-AAAA
ΔsteE + pSteE:2HASLSL-AAAA
ΔsteE + pSteE:2HASLPV-AAAA
ΔsteE + pSteE:2HAQKL-AAA
Red Squares indicate samples excluded from analysis  shown as dotted lines in the main figure
Ladder
kDa
α-HA Blot
Chemiluminescence image + Membrane overlay
 Membrane cut above 50 kDa
25
Ladder

## Slide 4
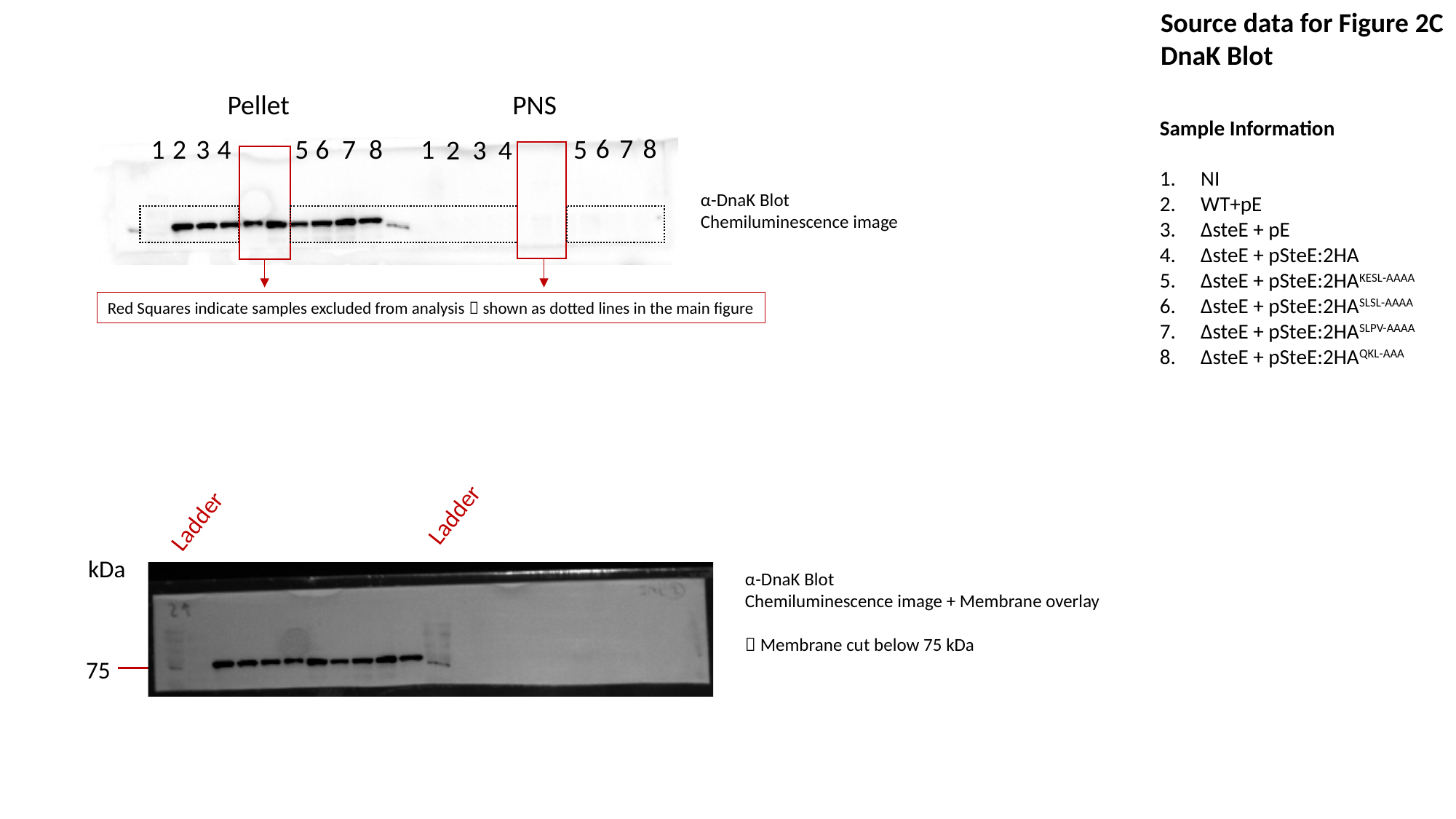

Source data for Figure 2C
DnaK Blot
Pellet
PNS
6
1
2
3
4
5
6
5
1
2
4
3
α-DnaK Blot
Chemiluminescence image
8
7
7
8
Sample Information
NI
WT+pE
ΔsteE + pE
ΔsteE + pSteE:2HA
ΔsteE + pSteE:2HAKESL-AAAA
ΔsteE + pSteE:2HASLSL-AAAA
ΔsteE + pSteE:2HASLPV-AAAA
ΔsteE + pSteE:2HAQKL-AAA
Red Squares indicate samples excluded from analysis  shown as dotted lines in the main figure
Ladder
kDa
α-DnaK Blot
Chemiluminescence image + Membrane overlay
 Membrane cut below 75 kDa
75
Ladder
