# Supplementary material for: Bacterial effectors mediate kinase reprogramming through mimicry of conserved eukaryotic motifs
Source: EMBO Rep. 2025 May 12;26(14):3529–53. doi: 10.1038/s44319-025-00472-y (PMC12287357; doi:10.1038/s44319-025-00472-y)
Supplement: Supplementary file 4 — Source data Fig. 2 [file 44319_2025_472_MOESM4_ESM.zip › Figure 2/2D/2D_readme.pptx]

## Slide 1
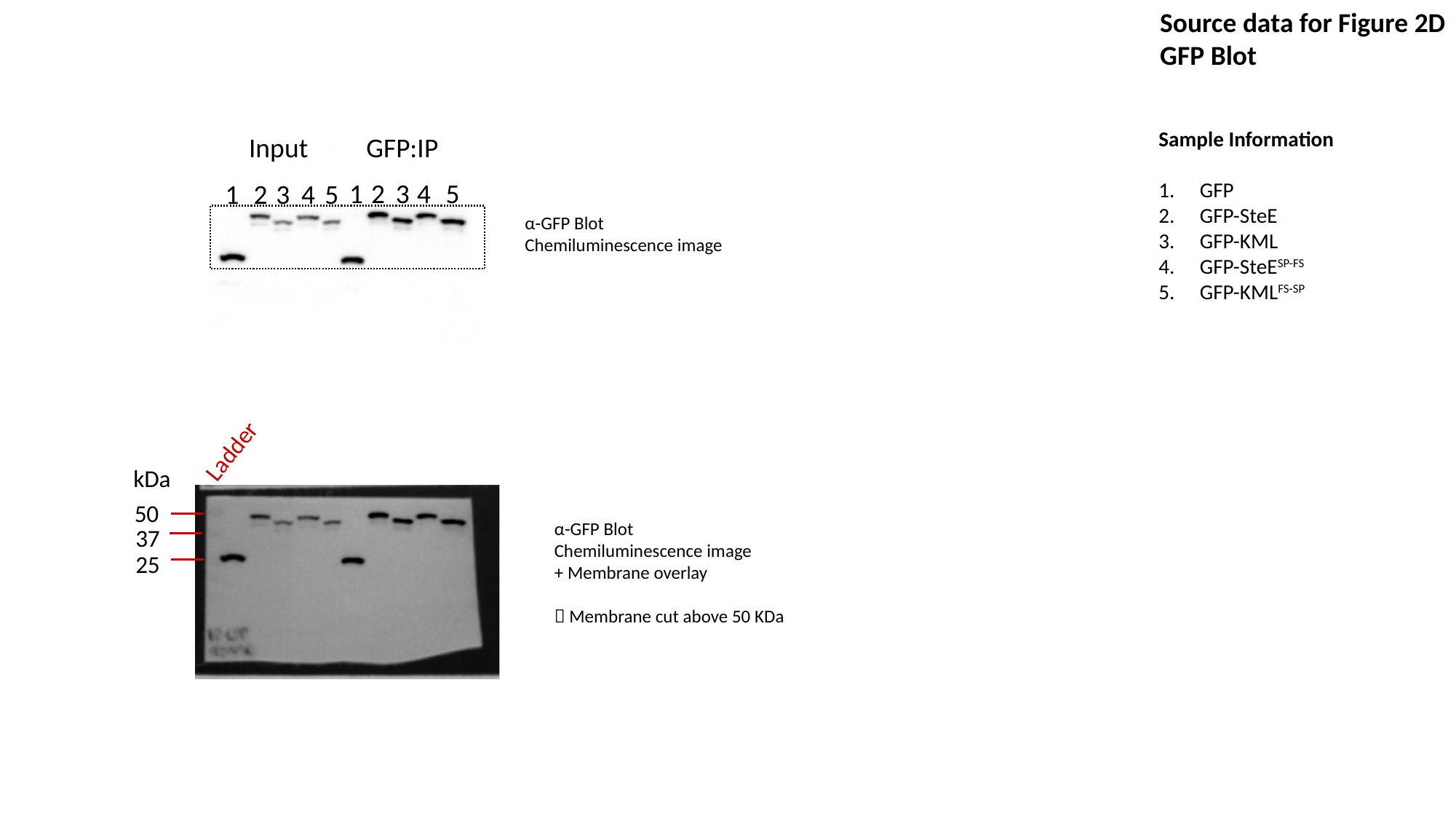

Source data for Figure 2D
GFP Blot
Sample Information
GFP
GFP-SteE
GFP-KML
GFP-SteESP-FS
GFP-KMLFS-SP
Input
GFP:IP
1
2
3
4
5
1
2
3
4
5
α-GFP Blot
Chemiluminescence image
Ladder
kDa
50
α-GFP Blot
Chemiluminescence image
+ Membrane overlay
 Membrane cut above 50 KDa
37
25

## Slide 2
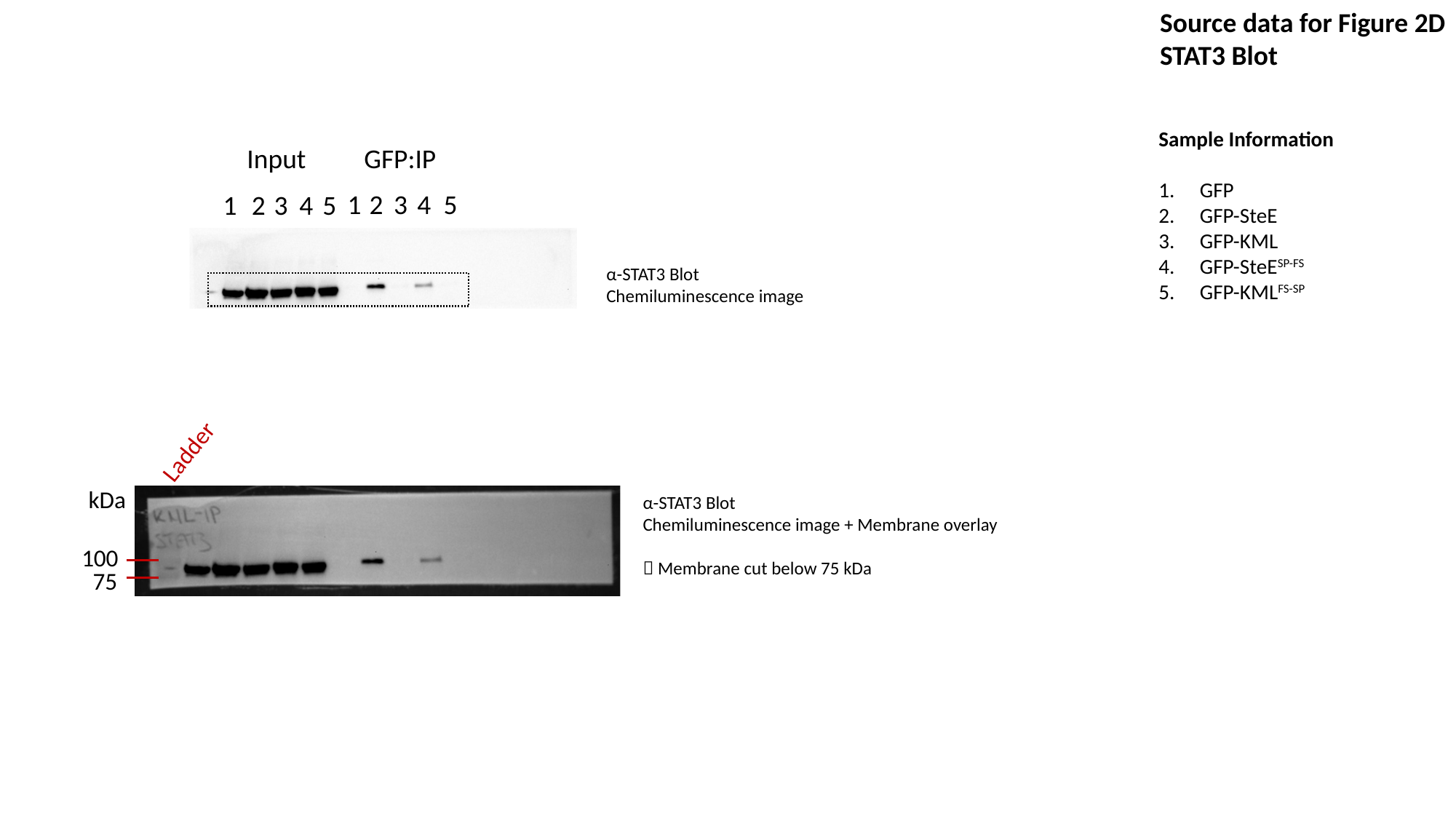

Source data for Figure 2D
STAT3 Blot
Sample Information
GFP
GFP-SteE
GFP-KML
GFP-SteESP-FS
GFP-KMLFS-SP
Input
GFP:IP
1
2
3
4
5
1
2
3
4
5
α-STAT3 Blot
Chemiluminescence image
Ladder
kDa
α-STAT3 Blot
Chemiluminescence image + Membrane overlay
 Membrane cut below 75 kDa
100
75

## Slide 3
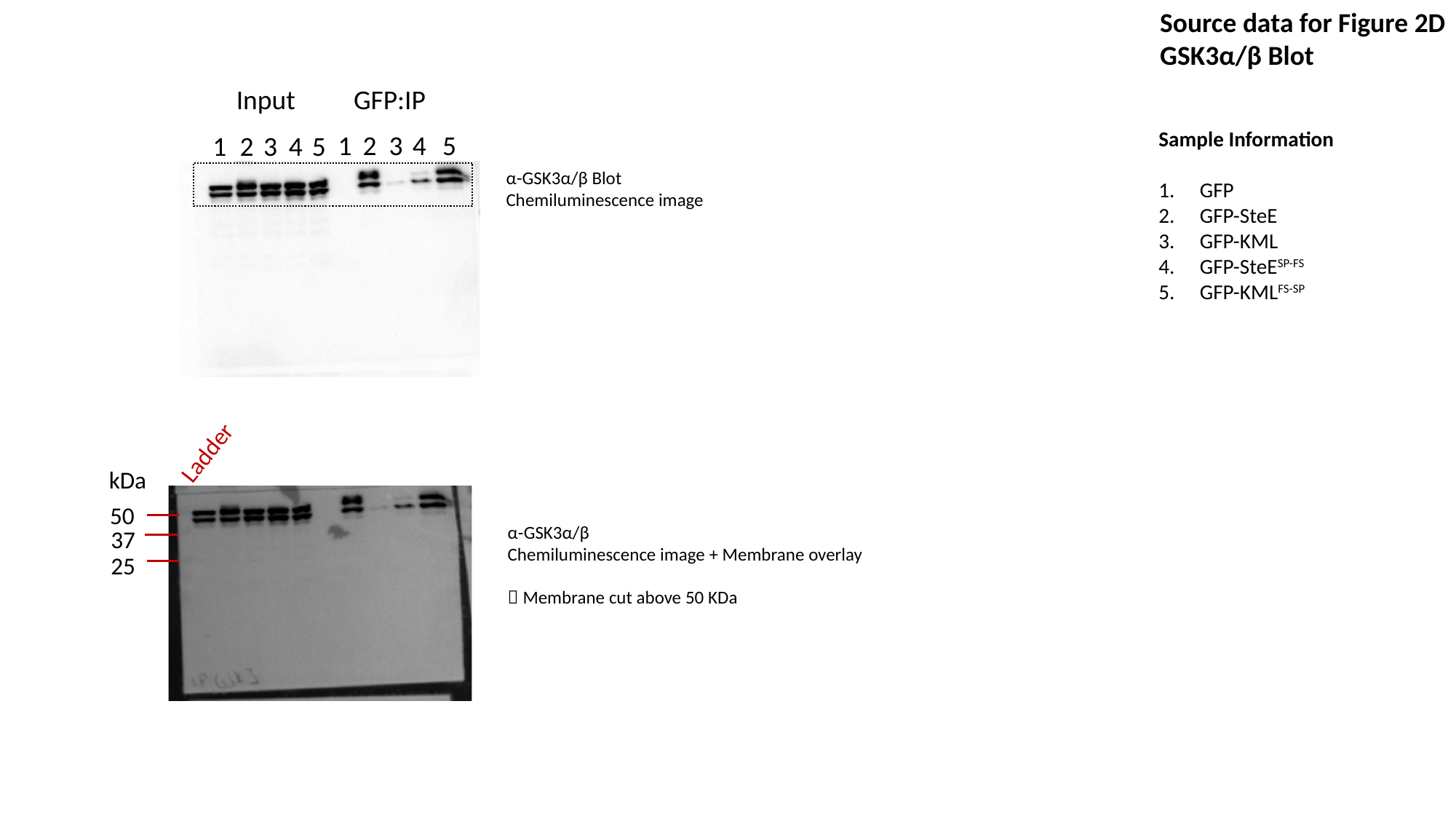

Source data for Figure 2D
GSK3α/β Blot
Input
GFP:IP
1
2
3
4
5
1
2
3
4
5
α-GSK3α/β Blot
Chemiluminescence image
Sample Information
GFP
GFP-SteE
GFP-KML
GFP-SteESP-FS
GFP-KMLFS-SP
Ladder
kDa
50
α-GSK3α/β
Chemiluminescence image + Membrane overlay
 Membrane cut above 50 KDa
37
25
