# Supplementary material for: Bacterial effectors mediate kinase reprogramming through mimicry of conserved eukaryotic motifs
Source: EMBO Rep. 2025 May 12;26(14):3529–53. doi: 10.1038/s44319-025-00472-y (PMC12287357; doi:10.1038/s44319-025-00472-y)
Supplement: Supplementary file 4 — Source data Fig. 2 [file 44319_2025_472_MOESM4_ESM.zip › Figure 2/2E/2E_readme.pptx]

## Slide 1
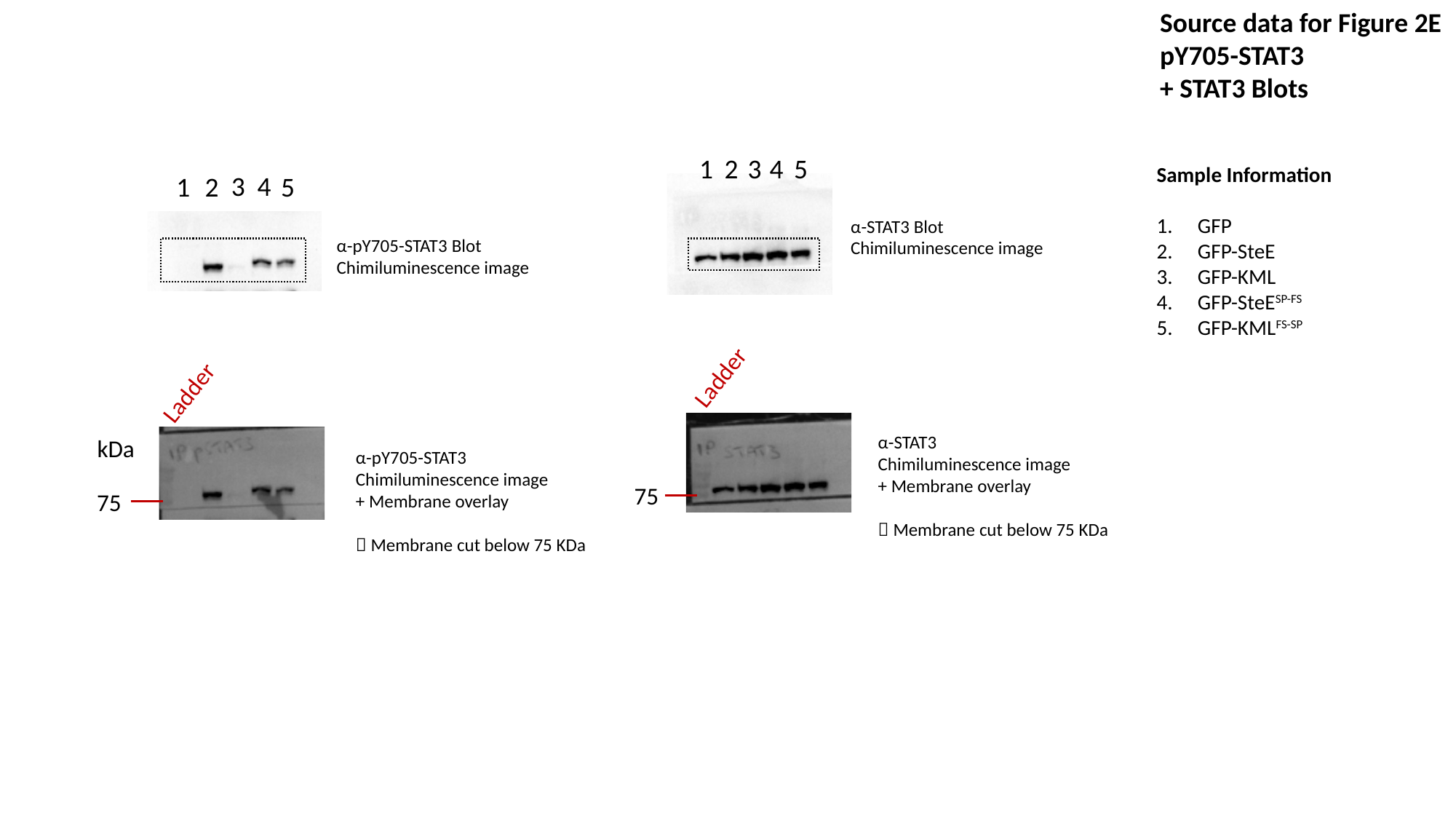

Source data for Figure 2E
pY705-STAT3
+ STAT3 Blots
1
2
3
4
5
α-STAT3 Blot
Chimiluminescence image
Sample Information
GFP
GFP-SteE
GFP-KML
GFP-SteESP-FS
GFP-KMLFS-SP
4
3
2
1
5
α-pY705-STAT3 Blot
Chimiluminescence image
Ladder
α-STAT3
Chimiluminescence image
+ Membrane overlay
 Membrane cut below 75 KDa
75
Ladder
kDa
α-pY705-STAT3
Chimiluminescence image
+ Membrane overlay
 Membrane cut below 75 KDa
75

## Slide 2
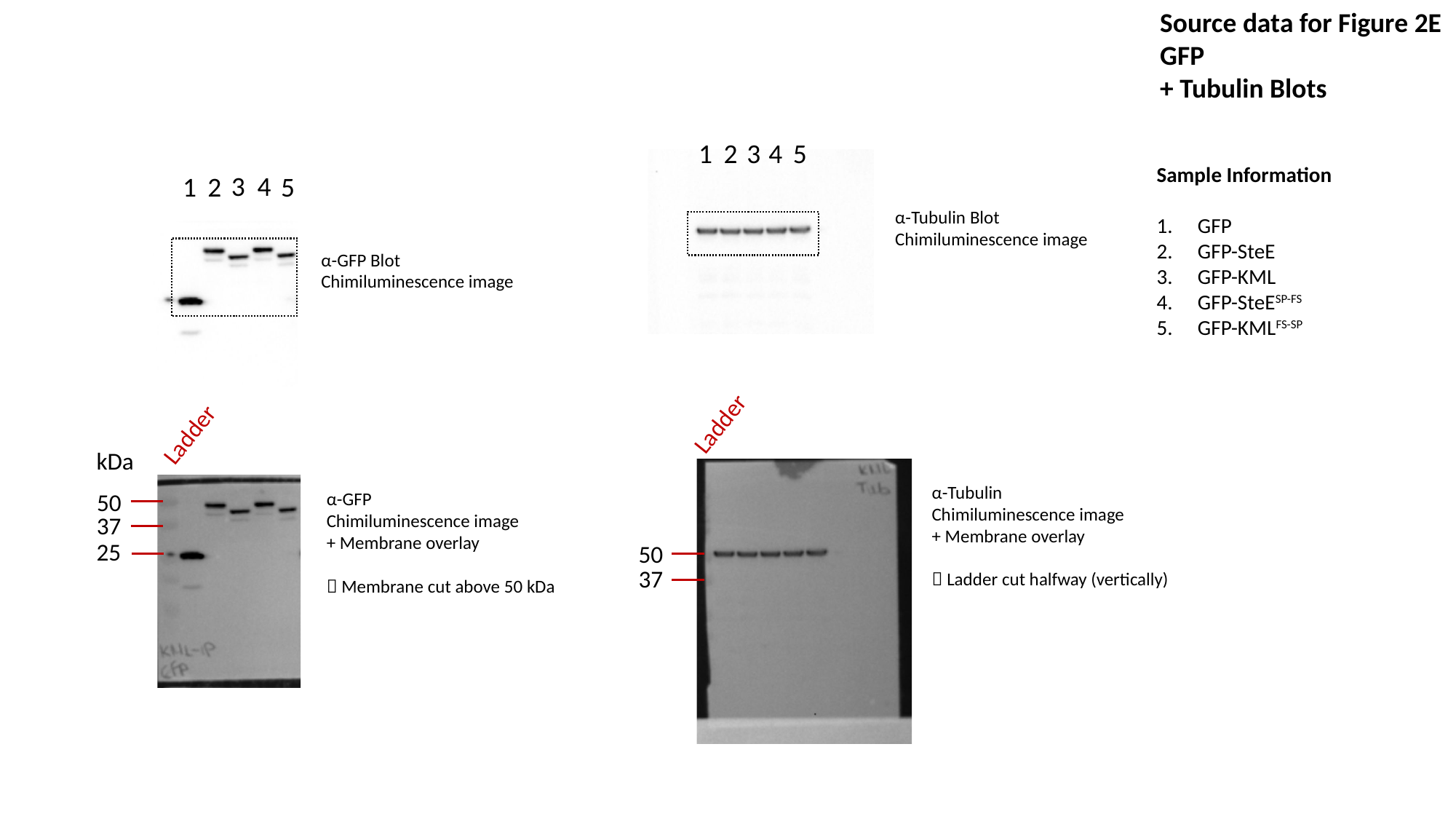

Source data for Figure 2E
GFP
+ Tubulin Blots
1
2
3
4
5
α-Tubulin Blot
Chimiluminescence image
Sample Information
GFP
GFP-SteE
GFP-KML
GFP-SteESP-FS
GFP-KMLFS-SP
4
3
2
1
5
α-GFP Blot
Chimiluminescence image
Ladder
α-Tubulin
Chimiluminescence image
+ Membrane overlay
 Ladder cut halfway (vertically)
50
37
Ladder
kDa
50
α-GFP
Chimiluminescence image
+ Membrane overlay
 Membrane cut above 50 kDa
37
25
