# Supplementary material for: Bacterial effectors mediate kinase reprogramming through mimicry of conserved eukaryotic motifs
Source: EMBO Rep. 2025 May 12;26(14):3529–53. doi: 10.1038/s44319-025-00472-y (PMC12287357; doi:10.1038/s44319-025-00472-y)
Supplement: Supplementary file 4 — Source data Fig. 2 [file 44319_2025_472_MOESM4_ESM.zip › Figure 2/2B/2B_readme.pptx]

## Slide 1
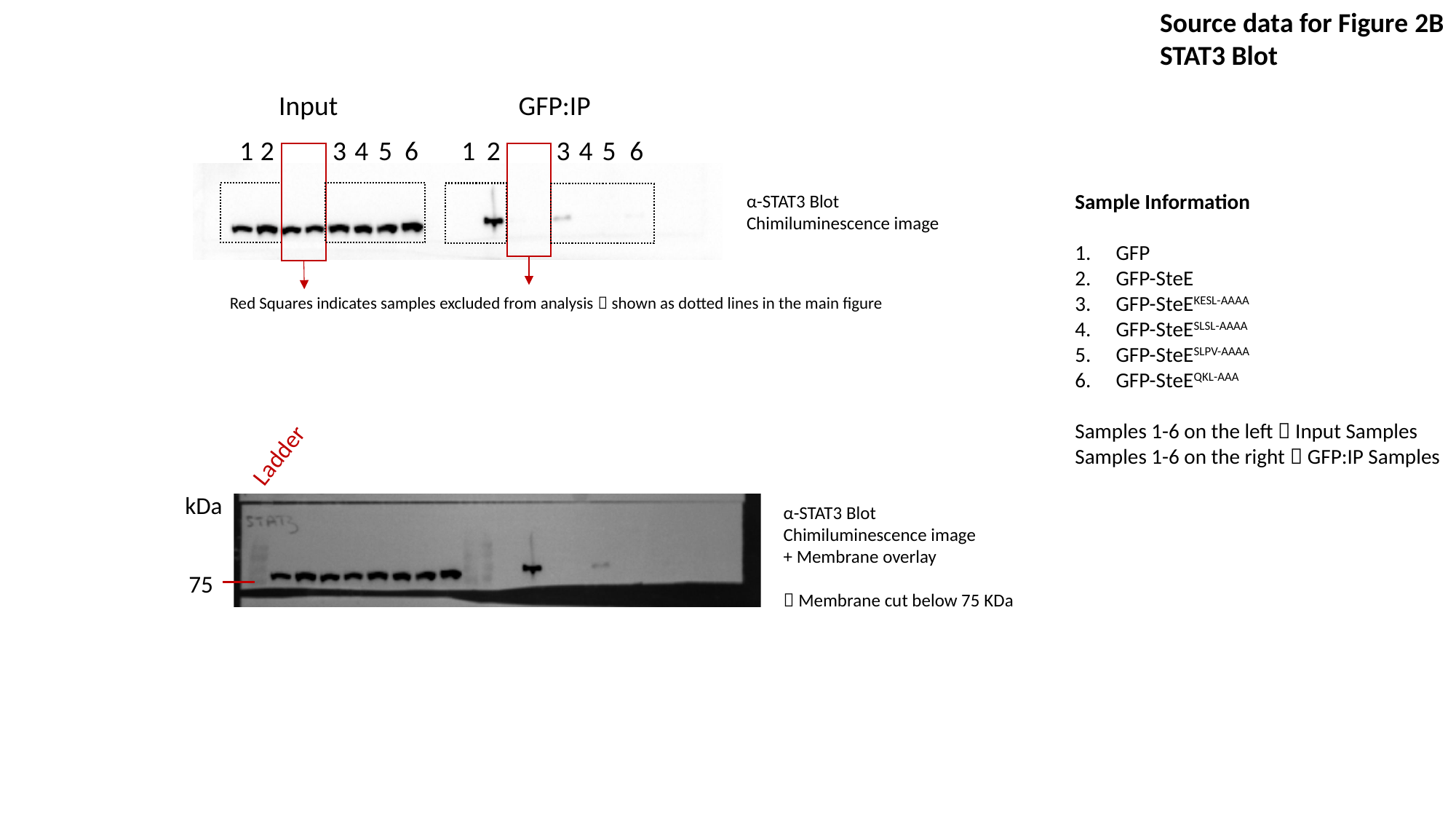

Source data for Figure 2B
STAT3 Blot
Input
GFP:IP
1
2
3
4
5
6
1
2
3
4
5
6
α-STAT3 Blot
Chimiluminescence image
Red Squares indicates samples excluded from analysis  shown as dotted lines in the main figure
Sample Information
GFP
GFP-SteE
GFP-SteEKESL-AAAA
GFP-SteESLSL-AAAA
GFP-SteESLPV-AAAA
GFP-SteEQKL-AAA
Samples 1-6 on the left  Input Samples
Samples 1-6 on the right  GFP:IP Samples
Ladder
kDa
α-STAT3 Blot
Chimiluminescence image
+ Membrane overlay
 Membrane cut below 75 KDa
75

## Slide 2
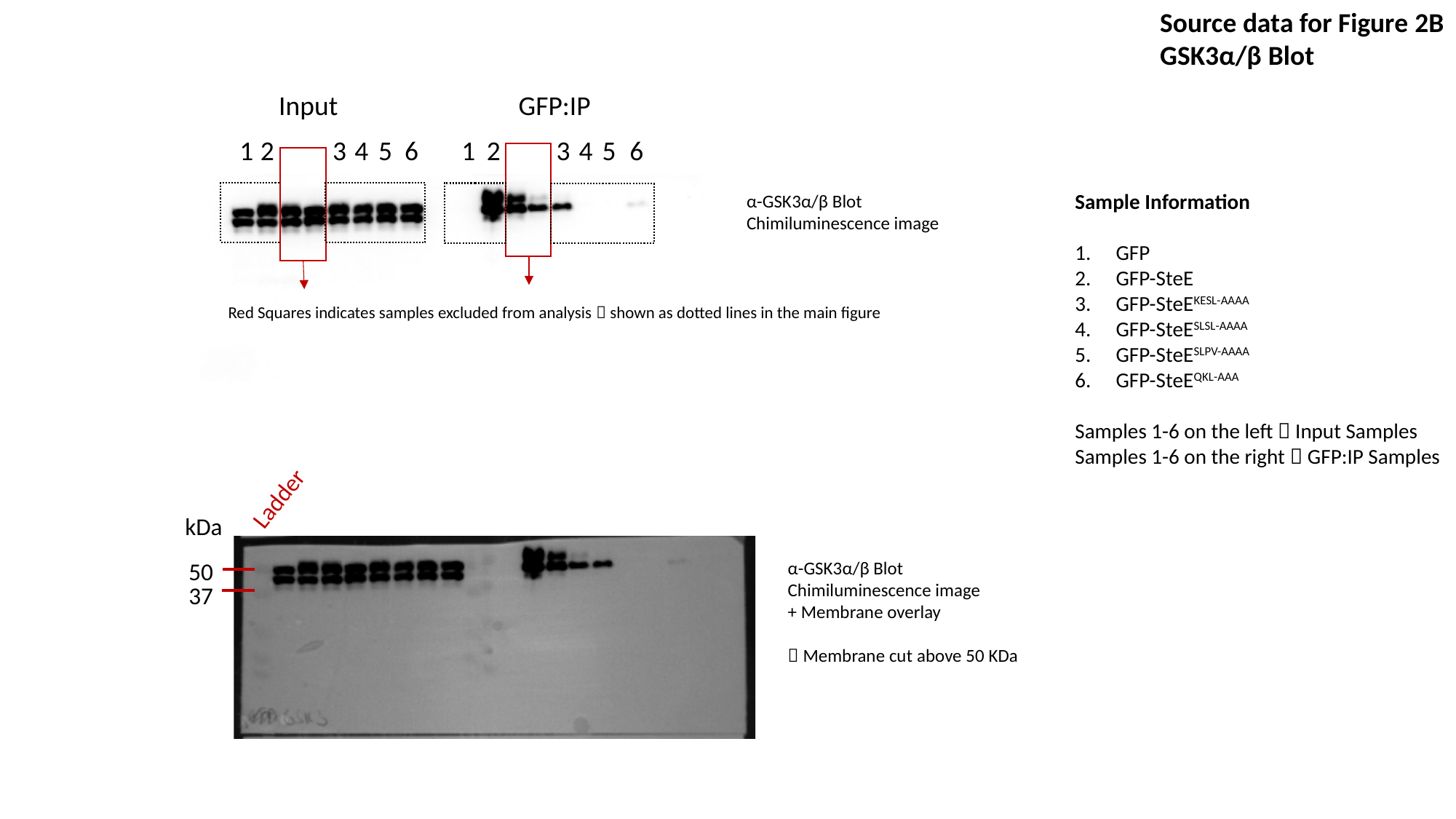

Source data for Figure 2B
GSK3α/β Blot
Input
GFP:IP
1
2
3
4
5
6
1
2
3
4
5
6
α-GSK3α/β Blot
Chimiluminescence image
Red Squares indicates samples excluded from analysis  shown as dotted lines in the main figure
Sample Information
GFP
GFP-SteE
GFP-SteEKESL-AAAA
GFP-SteESLSL-AAAA
GFP-SteESLPV-AAAA
GFP-SteEQKL-AAA
Samples 1-6 on the left  Input Samples
Samples 1-6 on the right  GFP:IP Samples
Ladder
kDa
50
α-GSK3α/β Blot
Chimiluminescence image
+ Membrane overlay
 Membrane cut above 50 KDa
37

## Slide 3
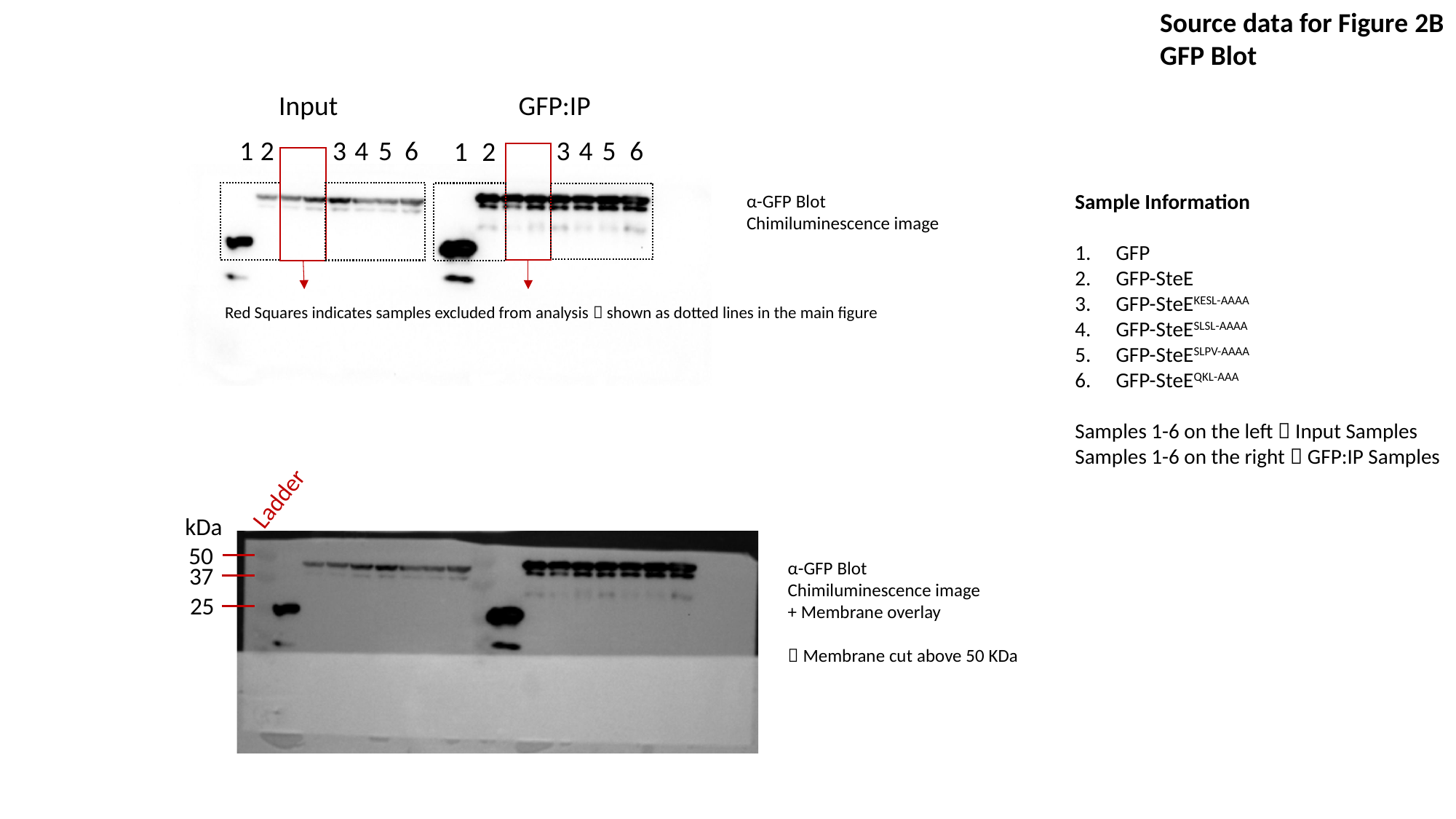

Source data for Figure 2B
GFP Blot
Input
GFP:IP
1
2
3
4
5
6
3
4
5
6
2
1
α-GFP Blot
Chimiluminescence image
Red Squares indicates samples excluded from analysis  shown as dotted lines in the main figure
Sample Information
GFP
GFP-SteE
GFP-SteEKESL-AAAA
GFP-SteESLSL-AAAA
GFP-SteESLPV-AAAA
GFP-SteEQKL-AAA
Samples 1-6 on the left  Input Samples
Samples 1-6 on the right  GFP:IP Samples
Ladder
kDa
50
α-GFP Blot
Chimiluminescence image
+ Membrane overlay
 Membrane cut above 50 KDa
37
25
