# Supplementary material for: Bacterial effectors mediate kinase reprogramming through mimicry of conserved eukaryotic motifs
Source: EMBO Rep. 2025 May 12;26(14):3529–53. doi: 10.1038/s44319-025-00472-y (PMC12287357; doi:10.1038/s44319-025-00472-y)
Supplement: Supplementary file 5 — Source data Fig. 3 [file 44319_2025_472_MOESM5_ESM.zip › Figure 3/3E/3E_readme.pptx]

## Slide 1
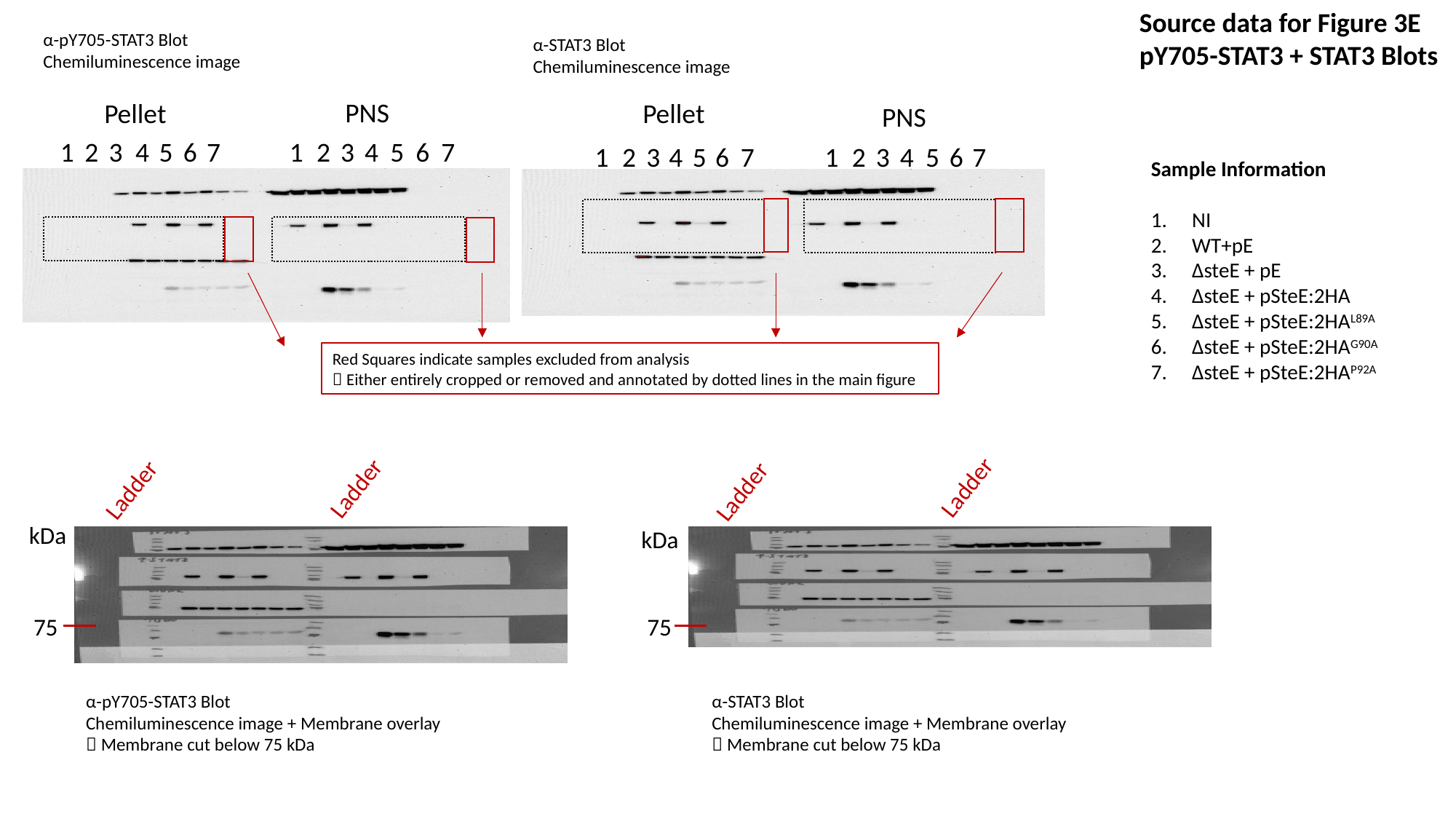

Source data for Figure 3E
pY705-STAT3 + STAT3 Blots
α-pY705-STAT3 Blot
Chemiluminescence image
PNS
Pellet
1
2
3
4
5
6
7
1
2
3
4
5
6
7
α-STAT3 Blot
Chemiluminescence image
Pellet
PNS
1
2
3
4
5
6
7
1
2
3
4
5
6
7
Sample Information
NI
WT+pE
ΔsteE + pE
ΔsteE + pSteE:2HA
ΔsteE + pSteE:2HAL89A
ΔsteE + pSteE:2HAG90A
ΔsteE + pSteE:2HAP92A
Red Squares indicate samples excluded from analysis
 Either entirely cropped or removed and annotated by dotted lines in the main figure
Ladder
Ladder
75
α-STAT3 Blot
Chemiluminescence image + Membrane overlay
 Membrane cut below 75 kDa
kDa
Ladder
Ladder
α-pY705-STAT3 Blot
Chemiluminescence image + Membrane overlay
 Membrane cut below 75 kDa
kDa
75

## Slide 2
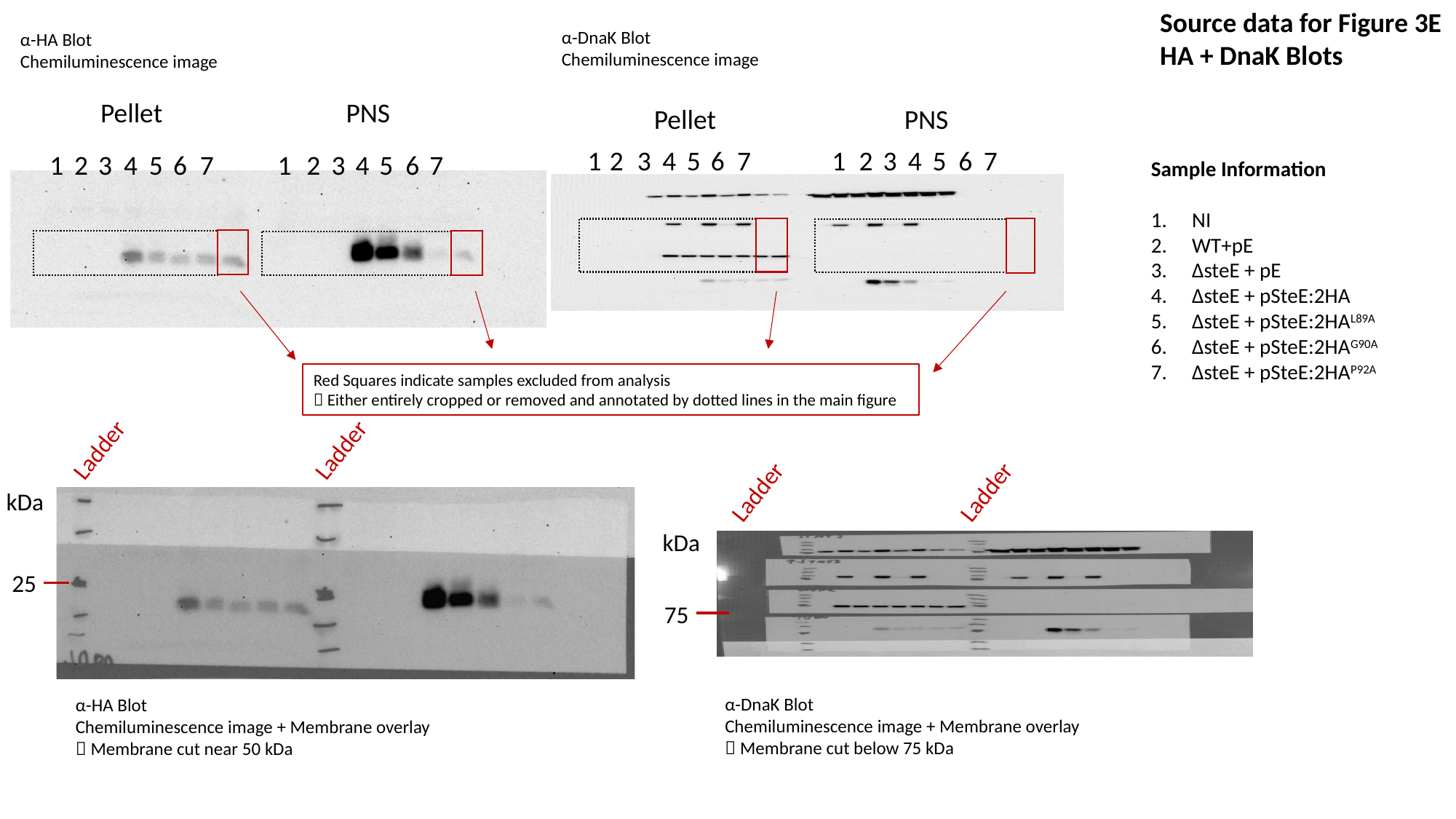

Source data for Figure 3E
HA + DnaK Blots
α-DnaK Blot
Chemiluminescence image
PNS
Pellet
1
2
3
4
5
6
7
1
2
3
4
5
6
7
α-HA Blot
Chemiluminescence image
Pellet
PNS
1
2
3
4
5
6
7
1
2
3
4
5
6
7
Sample Information
NI
WT+pE
ΔsteE + pE
ΔsteE + pSteE:2HA
ΔsteE + pSteE:2HAL89A
ΔsteE + pSteE:2HAG90A
ΔsteE + pSteE:2HAP92A
Red Squares indicate samples excluded from analysis
 Either entirely cropped or removed and annotated by dotted lines in the main figure
Ladder
Ladder
α-HA Blot
Chemiluminescence image + Membrane overlay
 Membrane cut near 50 kDa
25
kDa
Ladder
Ladder
75
α-DnaK Blot
Chemiluminescence image + Membrane overlay
 Membrane cut below 75 kDa
75
kDa

## Slide 3
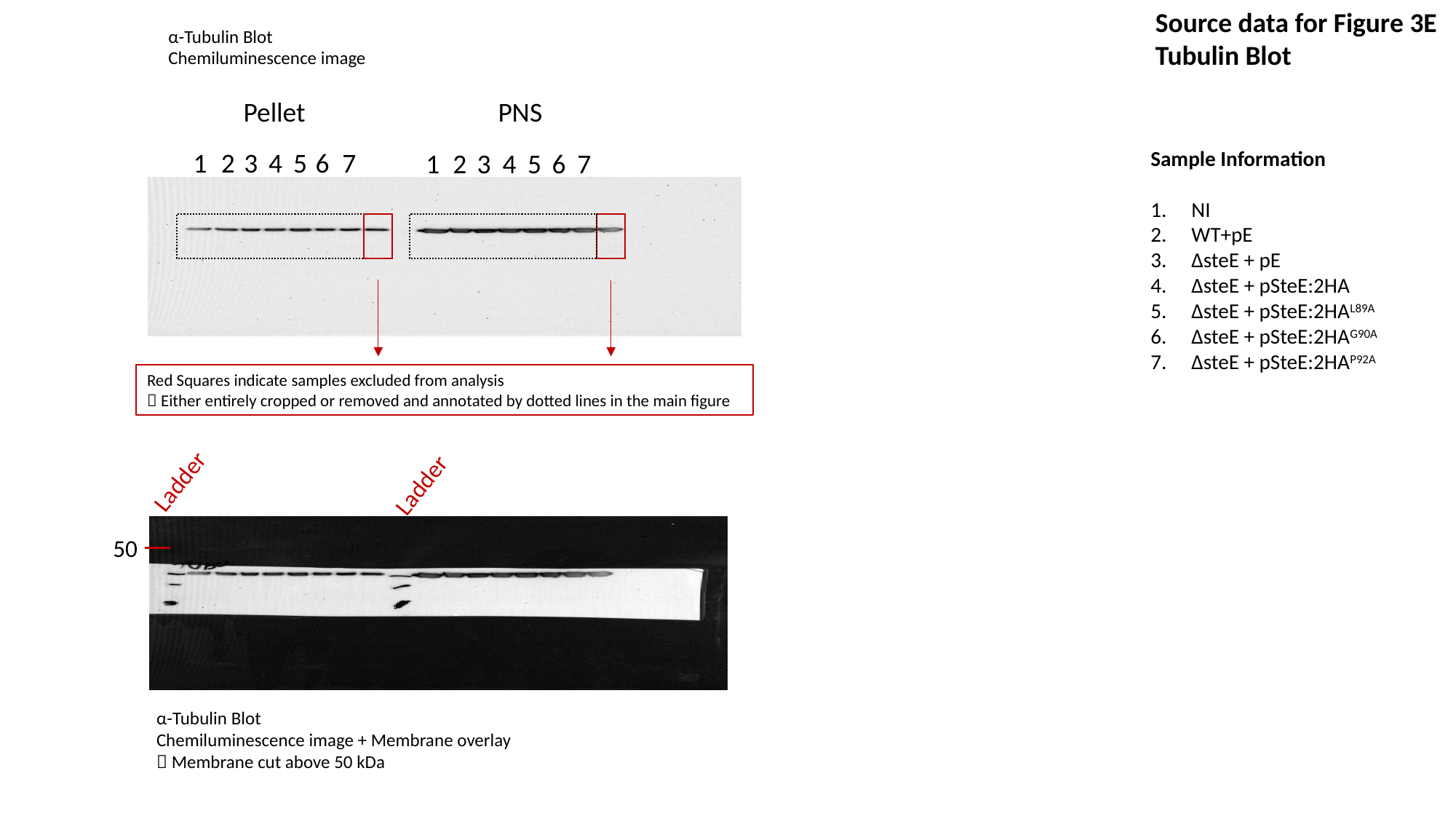

Source data for Figure 3E
Tubulin Blot
α-Tubulin Blot
Chemiluminescence image
Pellet
PNS
1
2
3
4
5
6
7
1
2
3
4
5
6
7
Red Squares indicate samples excluded from analysis
 Either entirely cropped or removed and annotated by dotted lines in the main figure
Sample Information
NI
WT+pE
ΔsteE + pE
ΔsteE + pSteE:2HA
ΔsteE + pSteE:2HAL89A
ΔsteE + pSteE:2HAG90A
ΔsteE + pSteE:2HAP92A
Ladder
Ladder
50
α-Tubulin Blot
Chemiluminescence image + Membrane overlay
 Membrane cut above 50 kDa
