# Supplementary material for: Bacterial effectors mediate kinase reprogramming through mimicry of conserved eukaryotic motifs
Source: EMBO Rep. 2025 May 12;26(14):3529–53. doi: 10.1038/s44319-025-00472-y (PMC12287357; doi:10.1038/s44319-025-00472-y)
Supplement: Supplementary file 5 — Source data Fig. 3 [file 44319_2025_472_MOESM5_ESM.zip › Figure 3/3B/3B_readme.pptx]

## Slide 1
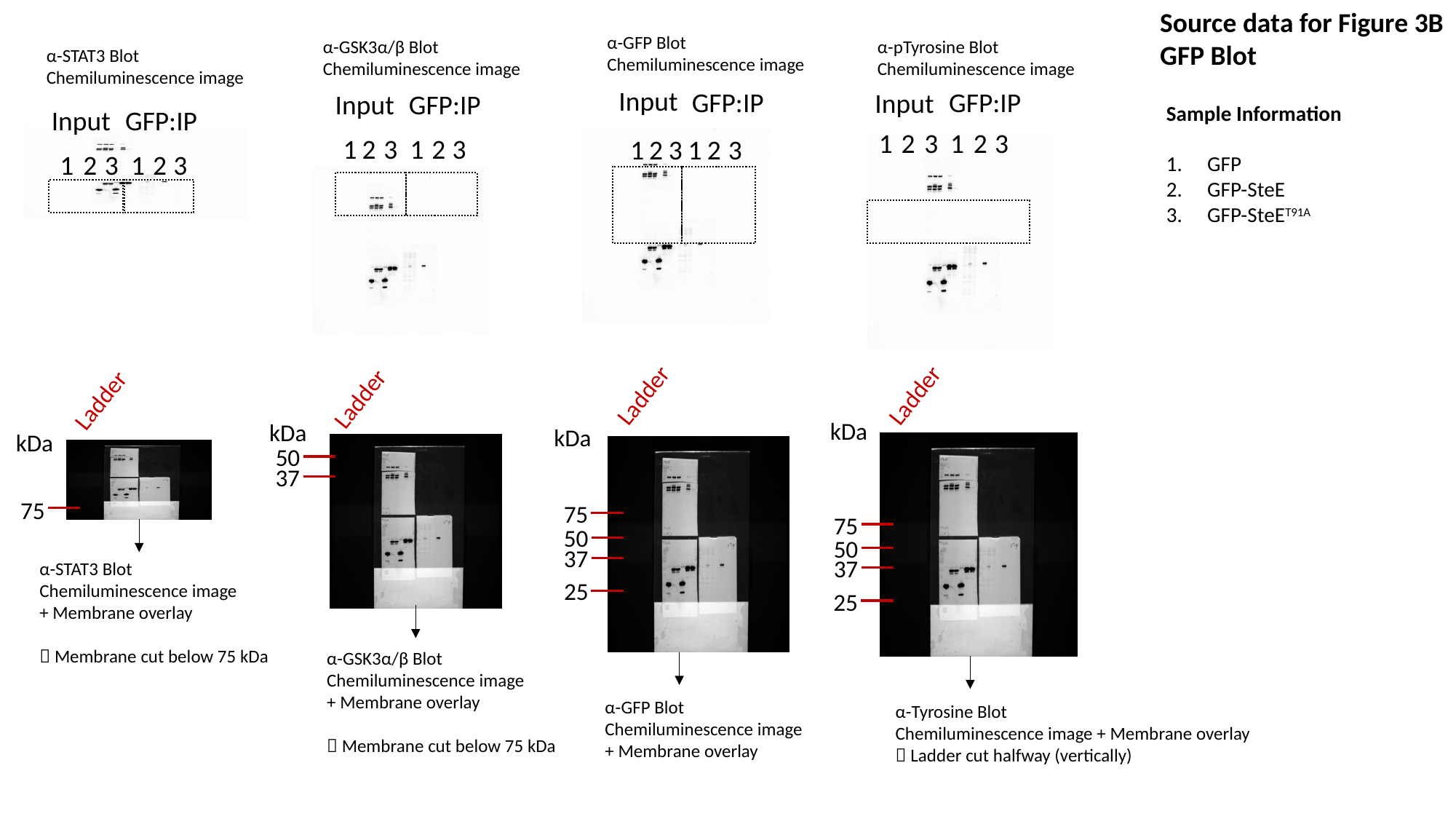

Source data for Figure 3B
GFP Blot
α-GFP Blot
Chemiluminescence image
Input
GFP:IP
1
2
3
1
2
3
α-GSK3α/β Blot
Chemiluminescence image
Input
GFP:IP
1
2
3
1
2
3
α-pTyrosine Blot
Chemiluminescence image
GFP:IP
Input
1
2
3
1
2
3
α-STAT3 Blot
Chemiluminescence image
Input
GFP:IP
1
2
3
1
2
3
Sample Information
GFP
GFP-SteE
GFP-SteET91A
Ladder
kDa
75
50
37
25
α-GFP Blot
Chemiluminescence image
+ Membrane overlay
Ladder
75
50
37
25
α-Tyrosine Blot
Chemiluminescence image + Membrane overlay
 Ladder cut halfway (vertically)
kDa
Ladder
kDa
50
37
α-GSK3α/β Blot
Chemiluminescence image
+ Membrane overlay
 Membrane cut below 75 kDa
Ladder
kDa
75
α-STAT3 Blot
Chemiluminescence image
+ Membrane overlay
 Membrane cut below 75 kDa
