# Supplementary material for: Bacterial effectors mediate kinase reprogramming through mimicry of conserved eukaryotic motifs
Source: EMBO Rep. 2025 May 12;26(14):3529–53. doi: 10.1038/s44319-025-00472-y (PMC12287357; doi:10.1038/s44319-025-00472-y)
Supplement: Supplementary file 5 — Source data Fig. 3 [file 44319_2025_472_MOESM5_ESM.zip › Figure 3/3H/3H_readme.pptx]

## Slide 1
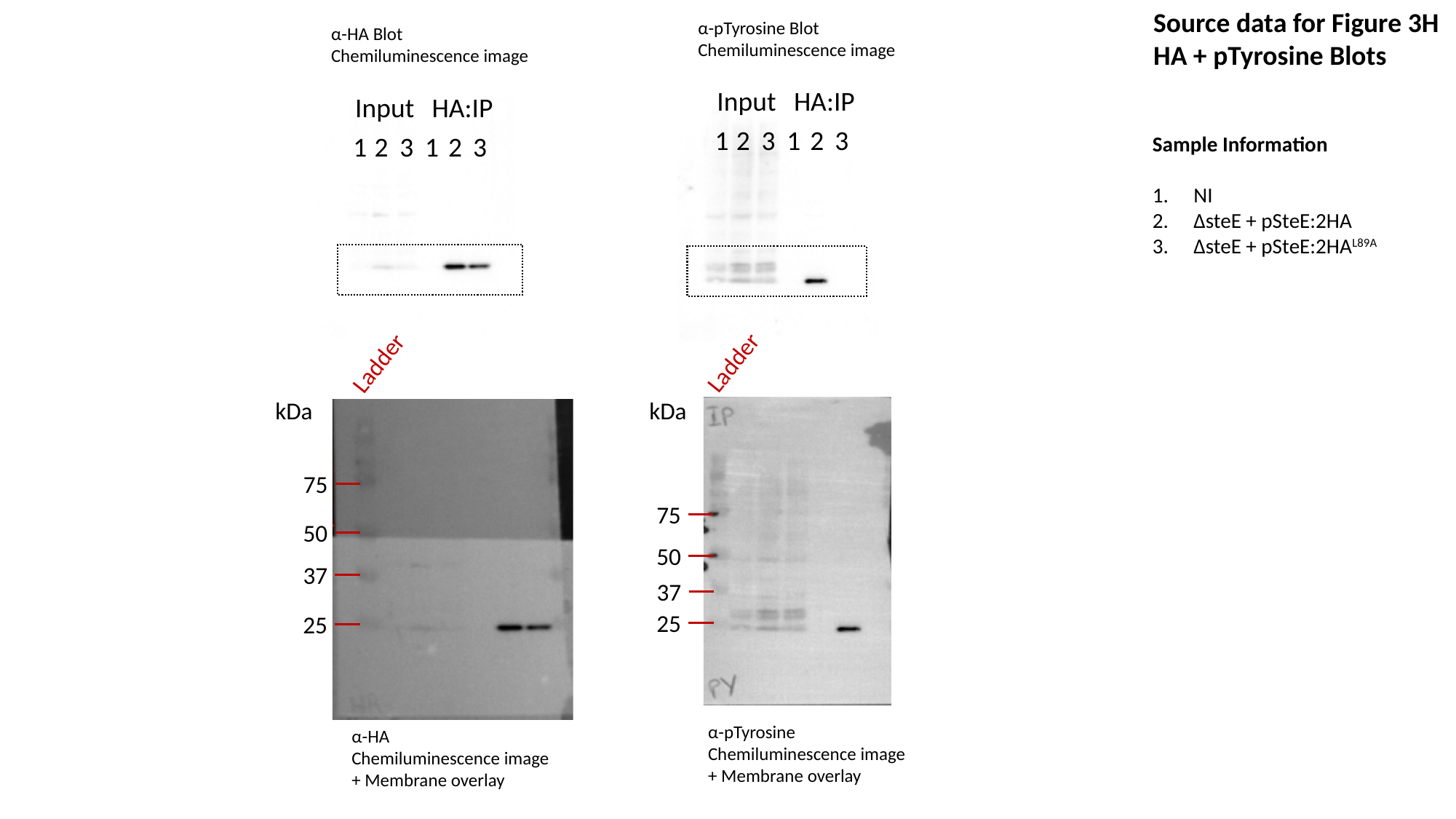

Source data for Figure 3H
HA + pTyrosine Blots
α-pTyrosine Blot
Chemiluminescence image
Input
HA:IP
1
2
3
1
2
3
α-HA Blot
Chemiluminescence image
Input
HA:IP
1
2
3
1
2
3
Sample Information
NI
ΔsteE + pSteE:2HA
ΔsteE + pSteE:2HAL89A
Ladder
75
50
37
25
α-pTyrosine
Chemiluminescence image
+ Membrane overlay
Ladder
kDa
kDa
75
50
37
25
α-HA
Chemiluminescence image
+ Membrane overlay

## Slide 2
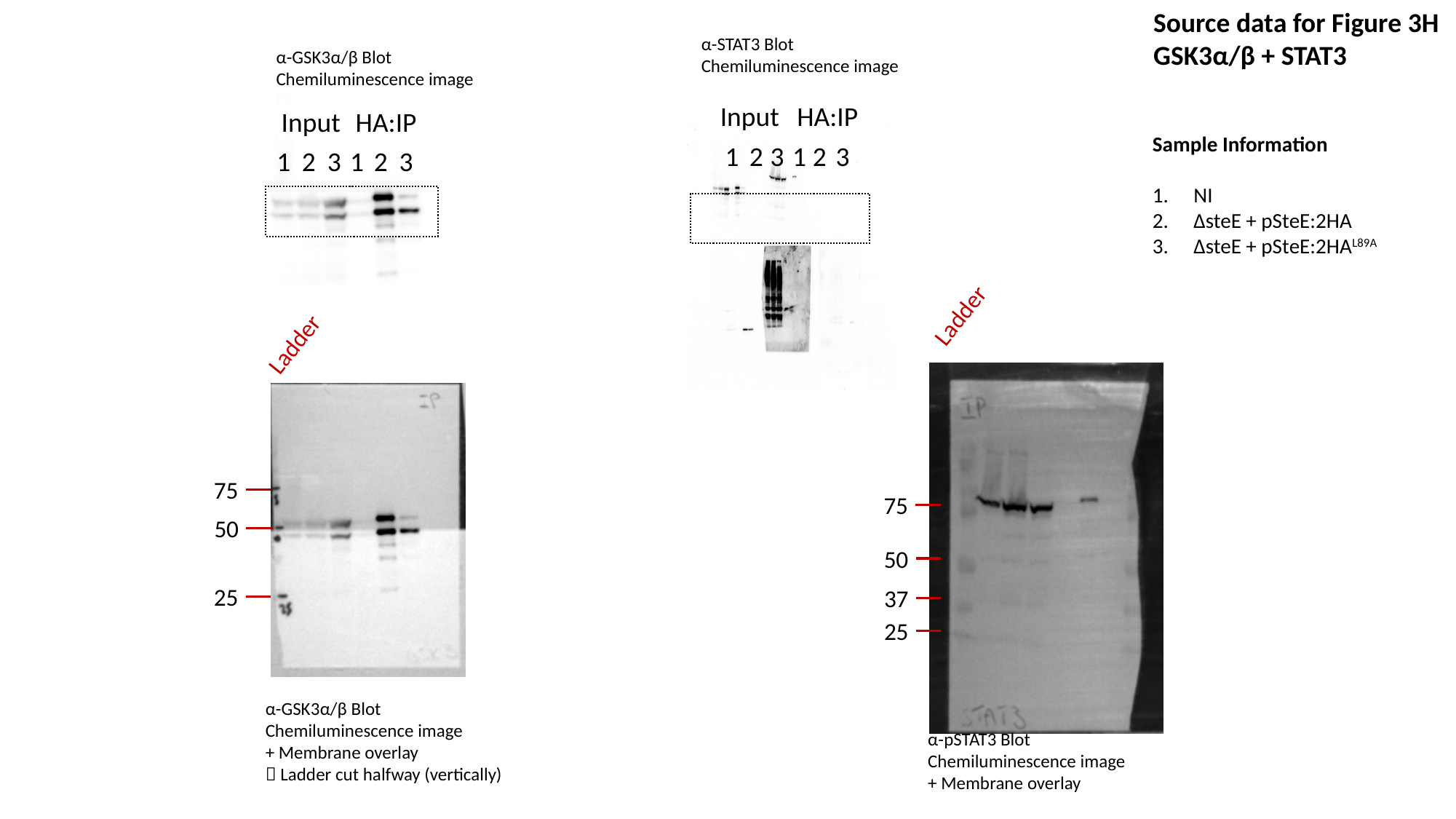

Source data for Figure 3H
GSK3α/β + STAT3
α-STAT3 Blot
Chemiluminescence image
Input
HA:IP
1
2
3
1
2
3
α-GSK3α/β Blot
Chemiluminescence image
Input
HA:IP
1
2
3
1
2
3
Sample Information
NI
ΔsteE + pSteE:2HA
ΔsteE + pSteE:2HAL89A
Ladder
75
50
37
25
α-pSTAT3 Blot
Chemiluminescence image
+ Membrane overlay
Ladder
75
50
25
α-GSK3α/β Blot
Chemiluminescence image
+ Membrane overlay
 Ladder cut halfway (vertically)
