# Supplementary material for: Bacterial effectors mediate kinase reprogramming through mimicry of conserved eukaryotic motifs
Source: EMBO Rep. 2025 May 12;26(14):3529–53. doi: 10.1038/s44319-025-00472-y (PMC12287357; doi:10.1038/s44319-025-00472-y)
Supplement: Supplementary file 5 — Source data Fig. 3 [file 44319_2025_472_MOESM5_ESM.zip › Figure 3/3A/3A_readme.pptx]

## Slide 1
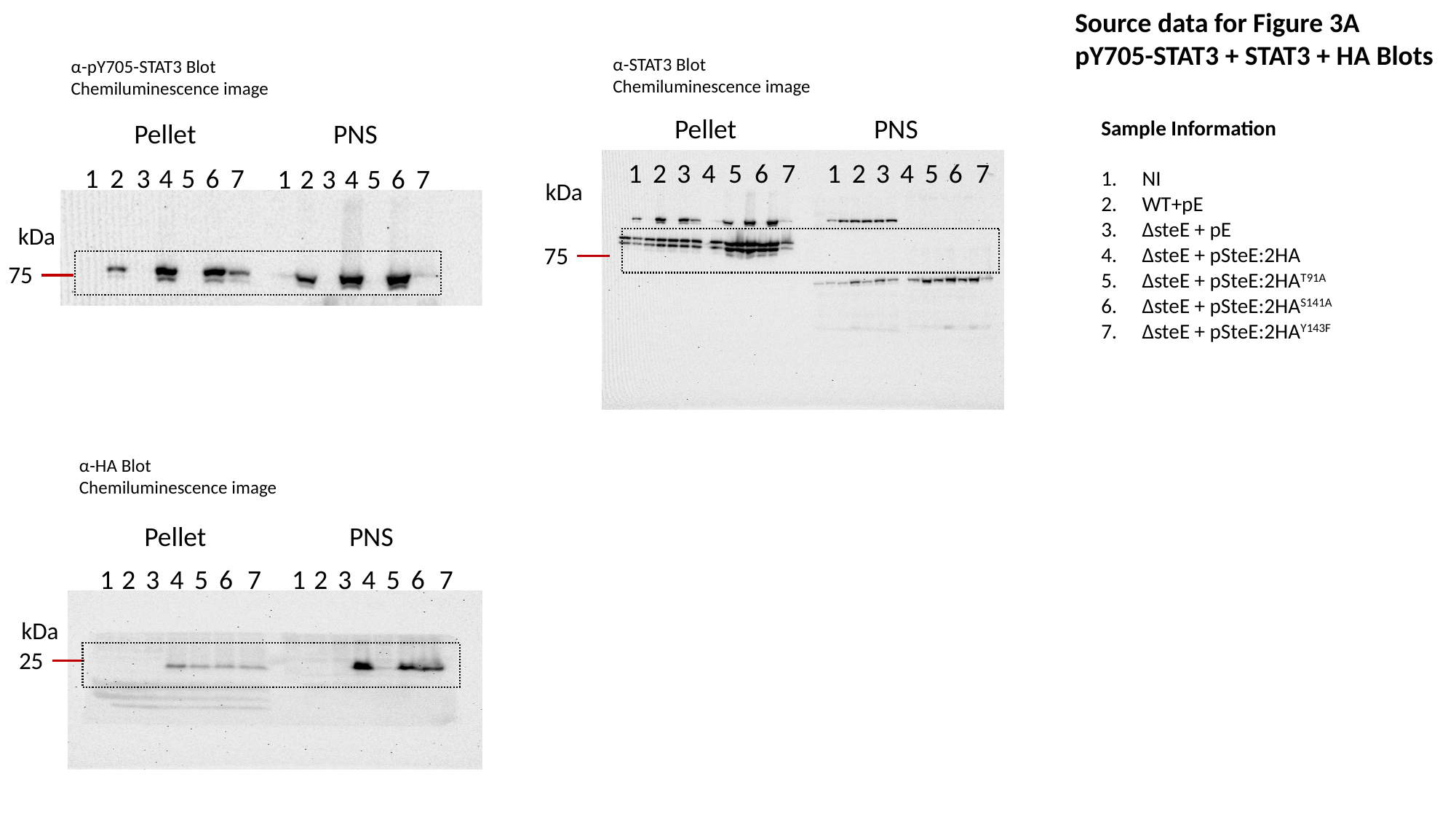

Source data for Figure 3A
pY705-STAT3 + STAT3 + HA Blots
α-STAT3 Blot
Chemiluminescence image
Pellet
PNS
1
2
3
4
5
6
7
1
2
3
4
5
6
7
kDa
75
α-pY705-STAT3 Blot
Chemiluminescence image
Pellet
PNS
1
2
3
4
5
6
7
1
2
3
4
5
6
7
kDa
75
Sample Information
NI
WT+pE
ΔsteE + pE
ΔsteE + pSteE:2HA
ΔsteE + pSteE:2HAT91A
ΔsteE + pSteE:2HAS141A
ΔsteE + pSteE:2HAY143F
α-HA Blot
Chemiluminescence image
1
2
3
4
5
6
7
1
2
3
4
5
6
7
PNS
Pellet
kDa
25

## Slide 2
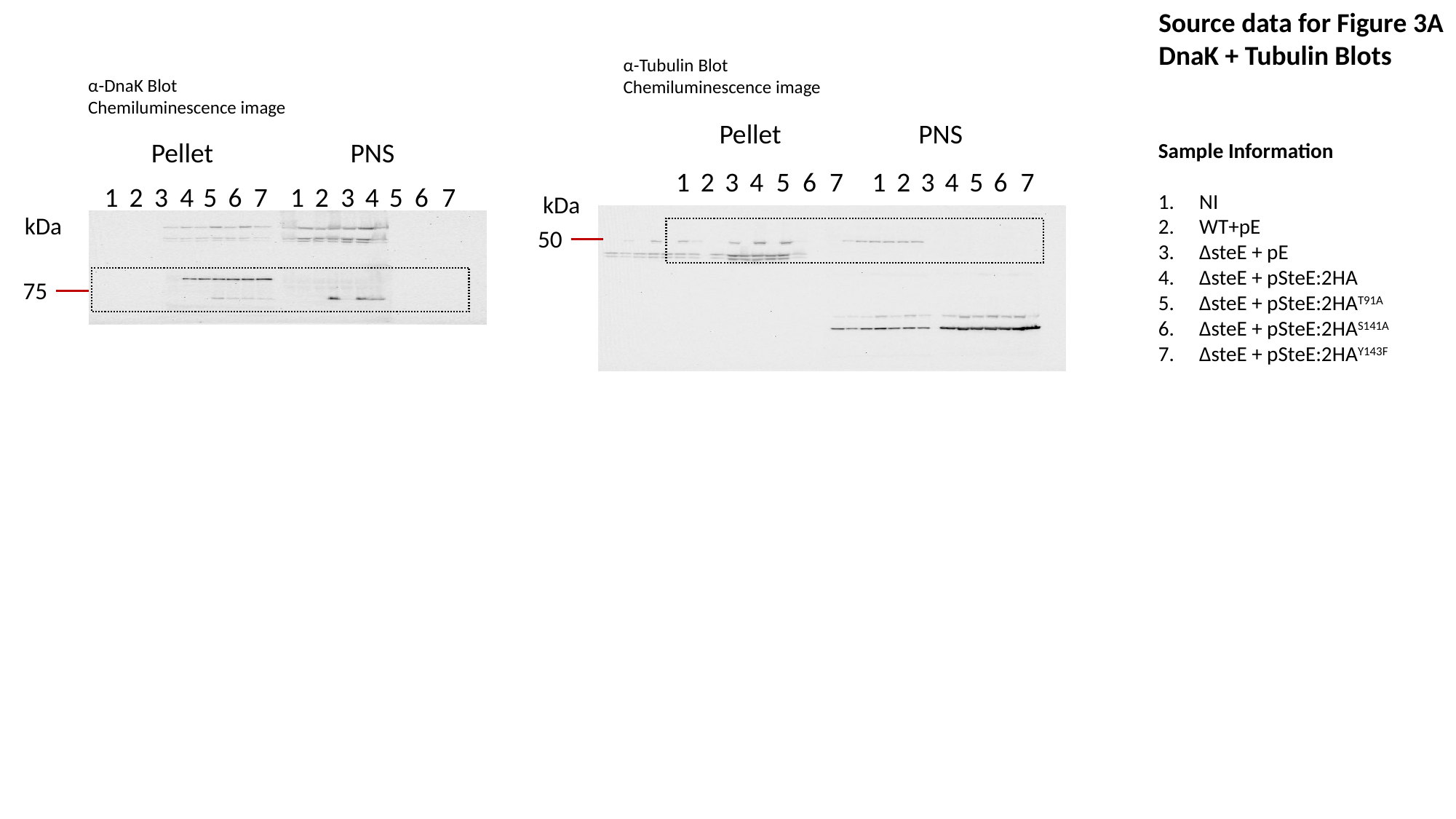

α-Tubulin Blot
Chemiluminescence image
Pellet
PNS
1
2
3
4
5
6
7
1
2
3
4
5
6
7
kDa
50
Source data for Figure 3A
DnaK + Tubulin Blots
α-DnaK Blot
Chemiluminescence image
Pellet
PNS
1
2
3
4
5
6
7
1
2
3
4
5
6
7
kDa
75
Sample Information
NI
WT+pE
ΔsteE + pE
ΔsteE + pSteE:2HA
ΔsteE + pSteE:2HAT91A
ΔsteE + pSteE:2HAS141A
ΔsteE + pSteE:2HAY143F
