# Supplementary material for: Bacterial effectors mediate kinase reprogramming through mimicry of conserved eukaryotic motifs
Source: EMBO Rep. 2025 May 12;26(14):3529–53. doi: 10.1038/s44319-025-00472-y (PMC12287357; doi:10.1038/s44319-025-00472-y)
Supplement: Supplementary file 5 — Source data Fig. 3 [file 44319_2025_472_MOESM5_ESM.zip › Figure 3/3F/3F_readme.pptx]

## Slide 1
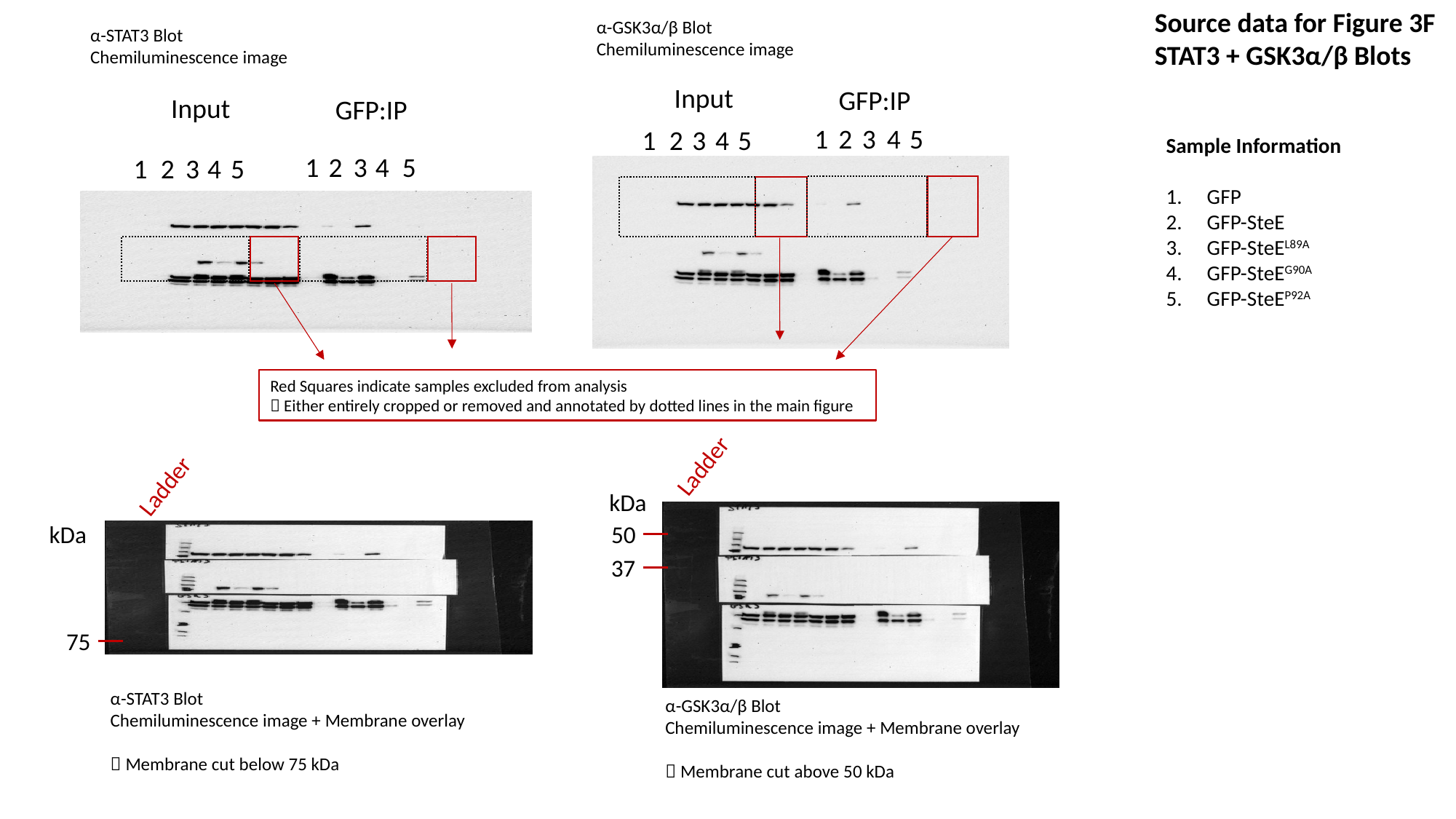

Source data for Figure 3F
STAT3 + GSK3α/β Blots
α-GSK3α/β Blot
Chemiluminescence image
α-STAT3 Blot
Chemiluminescence image
Input
GFP:IP
1
2
3
4
5
1
2
3
4
5
Input
GFP:IP
Sample Information
GFP
GFP-SteE
GFP-SteEL89A
GFP-SteEG90A
GFP-SteEP92A
1
2
3
4
5
1
2
3
4
5
Red Squares indicate samples excluded from analysis
 Either entirely cropped or removed and annotated by dotted lines in the main figure
Ladder
kDa
50
37
α-GSK3α/β Blot
Chemiluminescence image + Membrane overlay
 Membrane cut above 50 kDa
Ladder
kDa
75
α-STAT3 Blot
Chemiluminescence image + Membrane overlay
 Membrane cut below 75 kDa

## Slide 2
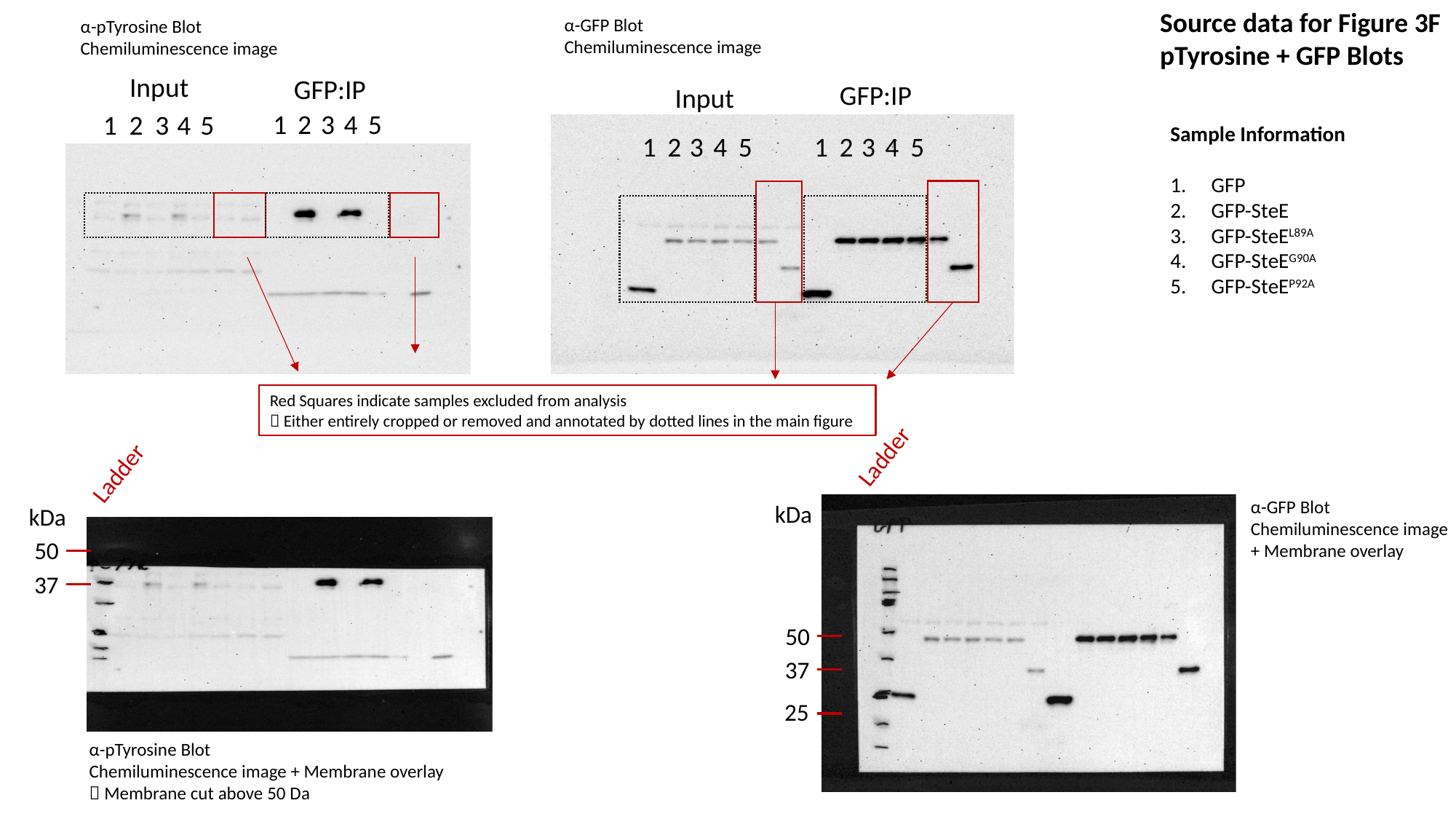

Source data for Figure 3F
pTyrosine + GFP Blots
α-GFP Blot
Chemiluminescence image
GFP:IP
Input
1
2
3
4
5
1
2
3
4
5
α-pTyrosine Blot
Chemiluminescence image
Input
GFP:IP
1
2
3
4
5
1
2
3
4
5
Sample Information
GFP
GFP-SteE
GFP-SteEL89A
GFP-SteEG90A
GFP-SteEP92A
Red Squares indicate samples excluded from analysis
 Either entirely cropped or removed and annotated by dotted lines in the main figure
Ladder
α-GFP Blot
Chemiluminescence image
+ Membrane overlay
kDa
50
37
25
Ladder
kDa
50
37
α-pTyrosine Blot
Chemiluminescence image + Membrane overlay
 Membrane cut above 50 Da
