# Supplementary material for: Bacterial effectors mediate kinase reprogramming through mimicry of conserved eukaryotic motifs
Source: EMBO Rep. 2025 May 12;26(14):3529–53. doi: 10.1038/s44319-025-00472-y (PMC12287357; doi:10.1038/s44319-025-00472-y)
Supplement: Supplementary file 5 — Source data Fig. 3 [file 44319_2025_472_MOESM5_ESM.zip › Figure 3/3G/3G_readme.pptx]

## Slide 1
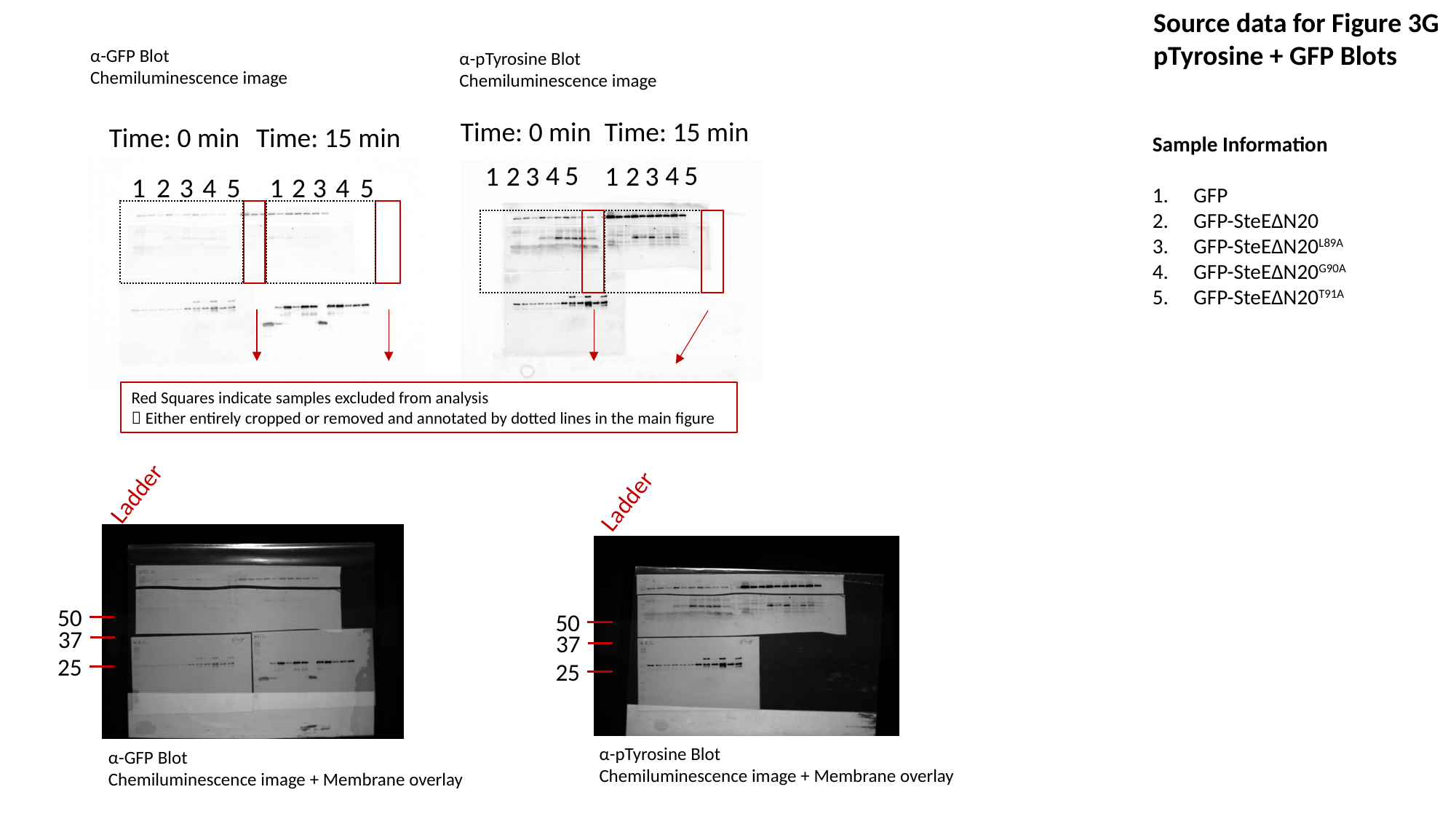

Source data for Figure 3G
pTyrosine + GFP Blots
α-GFP Blot
Chemiluminescence image
α-pTyrosine Blot
Chemiluminescence image
Time: 0 min
Time: 15 min
4
5
4
5
1
2
3
1
2
3
Time: 0 min
Time: 15 min
1
2
3
4
5
1
2
3
4
5
Red Squares indicate samples excluded from analysis
 Either entirely cropped or removed and annotated by dotted lines in the main figure
Sample Information
GFP
GFP-SteEΔN20
GFP-SteEΔN20L89A
GFP-SteEΔN20G90A
GFP-SteEΔN20T91A
Ladder
50
37
25
α-GFP Blot
Chemiluminescence image + Membrane overlay
Ladder
50
37
25
α-pTyrosine Blot
Chemiluminescence image + Membrane overlay
