# Supplementary material for: Bacterial effectors mediate kinase reprogramming through mimicry of conserved eukaryotic motifs
Source: EMBO Rep. 2025 May 12;26(14):3529–53. doi: 10.1038/s44319-025-00472-y (PMC12287357; doi:10.1038/s44319-025-00472-y)
Supplement: Supplementary file 7 — Expanded View Figures [file 44319_2025_472_MOESM7_ESM.pdf]

Expanded View Figures

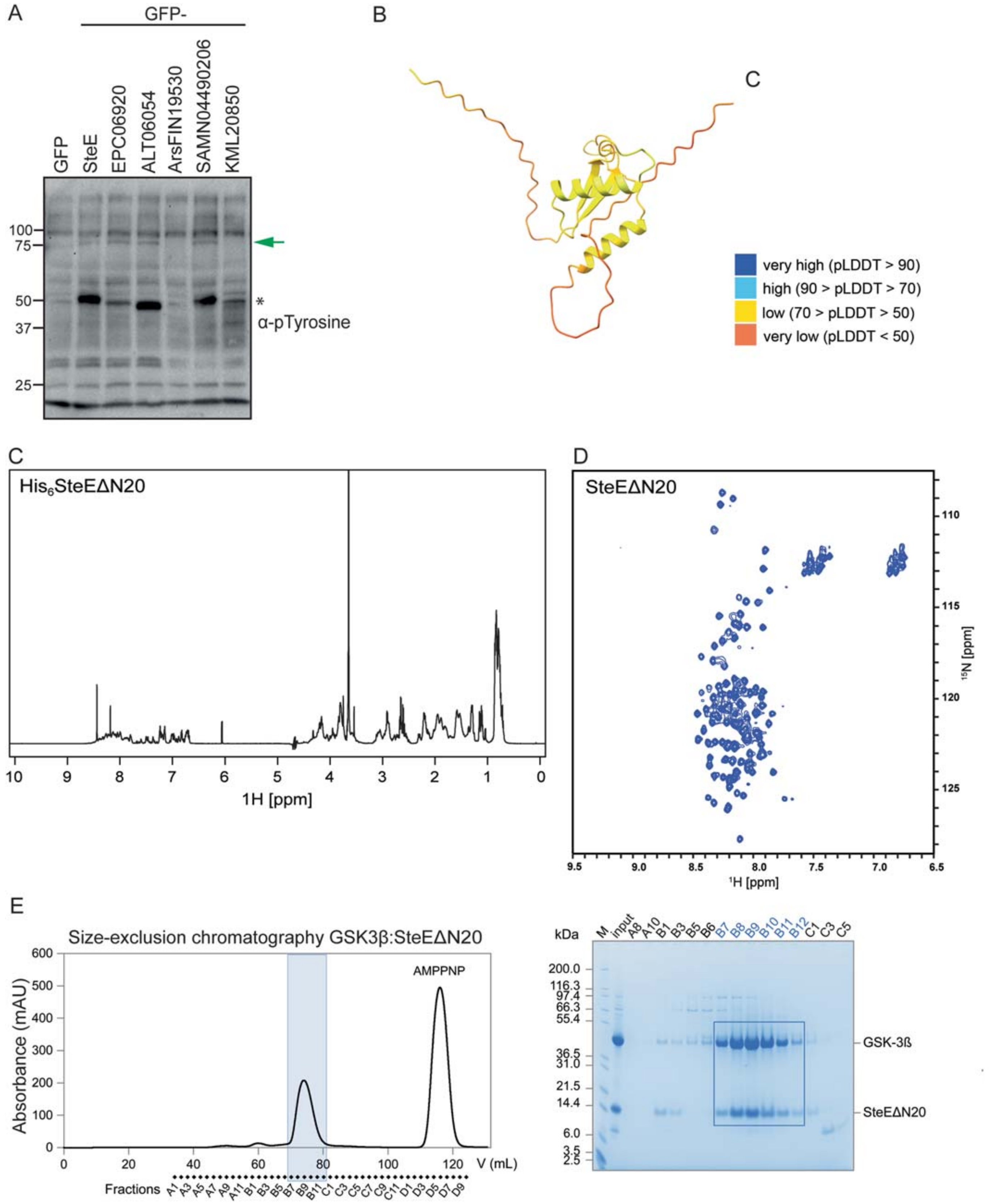

Figure EV1. SteE is a mainly disordered protein in solution.

(**A**) 293ET cells expressing GFP or GFP-tagged SteE homologues were lysed and analysed by immunoblotting for tyrosine phosphorylation status. Data represent at least three independent biological repeats. * Indicates bands at the expected molecular weight of the GFP-tagged SteE homologues whilst the green arrow represents a band at the expected molecular weight of STAT3. (**B**) AlphaFold3 prediction of SteE (STM2585), coloured according to pLDDT values. (**C**) 1D ^{1}H NMR spectrum of ^{15}N-isotopically labelled $His_6SteE^{\Delta N20}$ at 266 µM in 18 mM Tris-HCl, 90 mM NaCl, 0.9 mM DTT, 0.9 mM $MgCl^{2+}$, 10% (v/v) D_2O, pH 7.0 was recorded at 700 MHz at 298 K. (**D**) 2D $^{1}H,^{15}N$-sofast HMQC spectrum of ^{15}N-$His_6SteE^{\Delta N20}$ at 266 µM (same buffer as 1B), recorded at 700 MHz at 298 K. (**E**) Size-exclusion chromatography of $SteE^{\Delta N20}$ incubated with GSK3β. Data represent two independent biological repeats.

 © The Author(s)

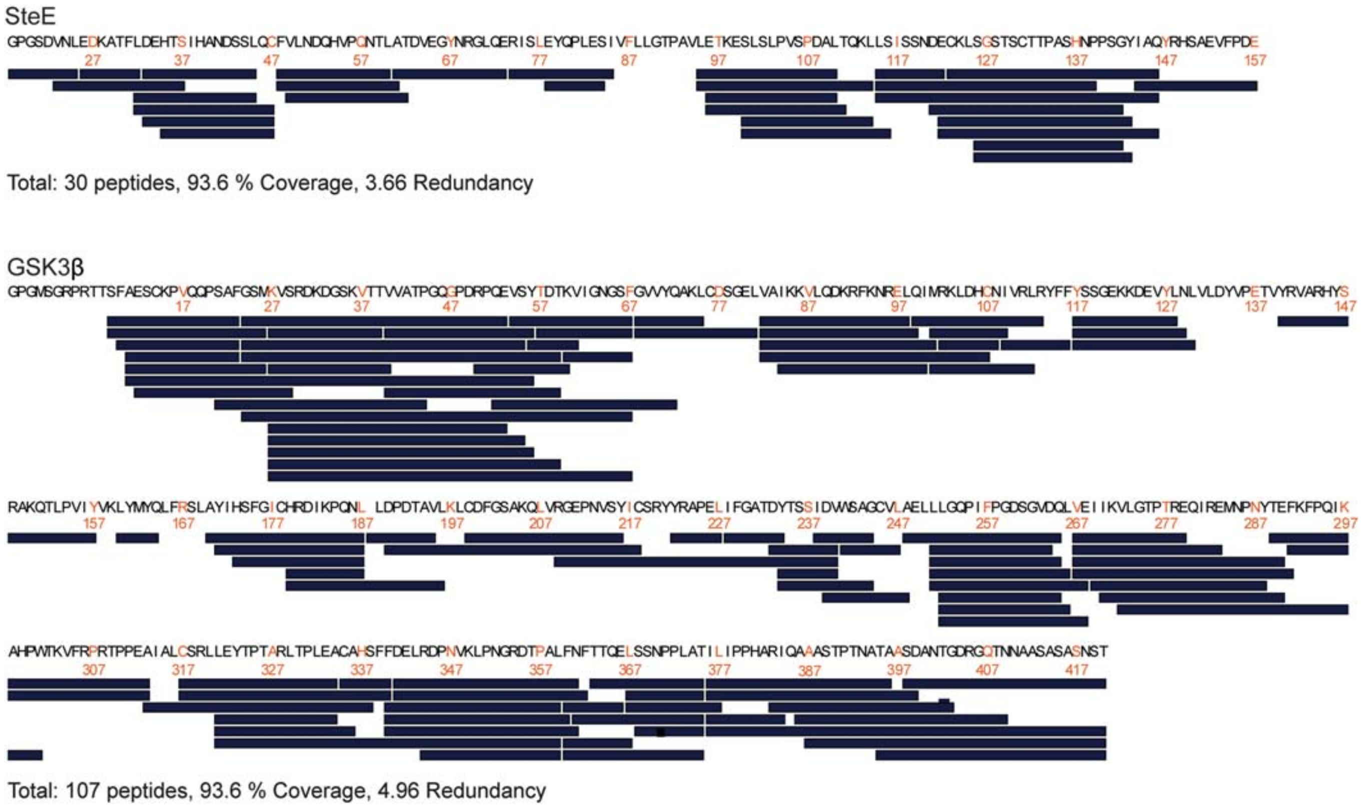

Figure EV2. Peptide coverage for HDX-MS.

Amino acid sequence and peptide coverage obtained for SteE and GSK3β. Related to Fig. 1E.

```
STM2585         -----------------------------------MFTIN-STNRVAST--------IAP  16
KML20850        MVE----------FVYNELNTLRKGTALGSVWESNMFTIITNADRMA-A--------AAA  41
EPC06920        MLR---------SQRFL--SIPKLDYICDHVMEENMLAI-HNLNRI--E--------TAV  38
ALT06054        ------------------------------------------------------------  0
ArsFIN19530     ------------------------------------------------------------  0
SAMN04490206    MLSSRFDSVISSNQVLSEHTVEGAGSQRASVLSENTLSVQQTSRSMCKANDAGVDRQCIT  60

STM2585         YACVSDVNLEDKATFLDEHTSI-------HANDSSLQCFVLNDQHVPQNTLATDVEGYN-  68
KML20850        DTHLNHVNVEGKSGVVNLVVSVKNDPINMLRPDNFSQCVILNNLHVPQGALVTDIDNYN-  100
EPC06920        VKHVNCANVEGKPTFVASVSSVENVSTDMLKPKIFAQCVILNNLHVPQNIPDTDIEGYN-  97
ALT06054        -----------------------------MISNNMIKCNVFHDLHIPNDAAESDVETYK-  30
ArsFIN19530     --MSNKINITKDRPLIKNSQ-QQNQLKTNALISKIKNCFVLNNIHISTTIGSSKAKFYHQ  57
SAMN04490206    NACEVNAENNGVRVLMSALVSVGNVVSTGFVRQEINKVIVRDDIHVPQGSMPHDRKMCD-  119
                                                .   :  :  .: *:       . . .

STM2585         ------------------RGLQERISLEYQPLESIVFLLGTPAVLETKESLSLPVSPDAL  110
KML20850        ------------------KGLQLRINLEYNPKGSIVFLLGSPEALDANESLSLPIFSHVL  142
EPC06920        ------------------KGMQVRINQEYQPQGKTVFLLGSPEVLEPDESLSLPASPHIL  139
ALT06054        ------------------NDLSERISCEYHTDSNEVFILASPEELEDTESLSLAVSPKAL  72
ArsFIN19530     LMLRLNLDSSIQIKNYHHYHLTSRLNLEYNSTTENVALQGTPKNIHSAESLSLPVCPFLL  117
SAMN04490206    ------------------EGQRIRLNDEYHPLHHTVFLQGTPERLGIHHQLSLPVSPSML  161
                                   *:. **:     * : .:*  :   ..***     *

STM2585         TQKLLSISSNDECKLSGSTSCTTPASHNPPSGYIAQYRHSAEVFPDE-------------  157
KML20850        TQKLLNISNSKLCELSVKSNGYV-------------------------------------  165
EPC06920        AQKLSSIANIKACAFSFESNGYVQRSE---DNFI--YRNGTS-LPL--------------  179
ALT06054        HQAISC-------ELAKMTDHDLRERD--------MVETGKEIKPEEDVTKLHEYIIRAN  117
ArsFIN19530     SQKLIQVKNKFT----KKKKCKMKELD---------------------------------  140
SAMN04490206    TEKLIEVIREKNE-------KEQRASQAEKNGYVCQVGSVMTT-----------------  197
                 : :

STM2585         ---     157
KML20850        ---     165
EPC06920        ---     179
ALT06054        GYV     120
ArsFIN19530     ---     140
SAMN04490206    ---     197
```

Figure EV3. Sequence alignment between SteE and its putative homologues.

Amino acid sequence alignment between SteE (STM2585) and its putative homologues as generated by Clustal Omega. Conserved motifs within residues 95–116 of SteE are shown with shading. Known phosphosites in SteE are shown in bold. Asterisk (*) = fully conserved residue, Colon (:) = amino acid residues with similar properties, Period (.) = amino acid with weakly similar properties. Amino acid sequences are listed in Appendix Table S3.

 © The Author(s)

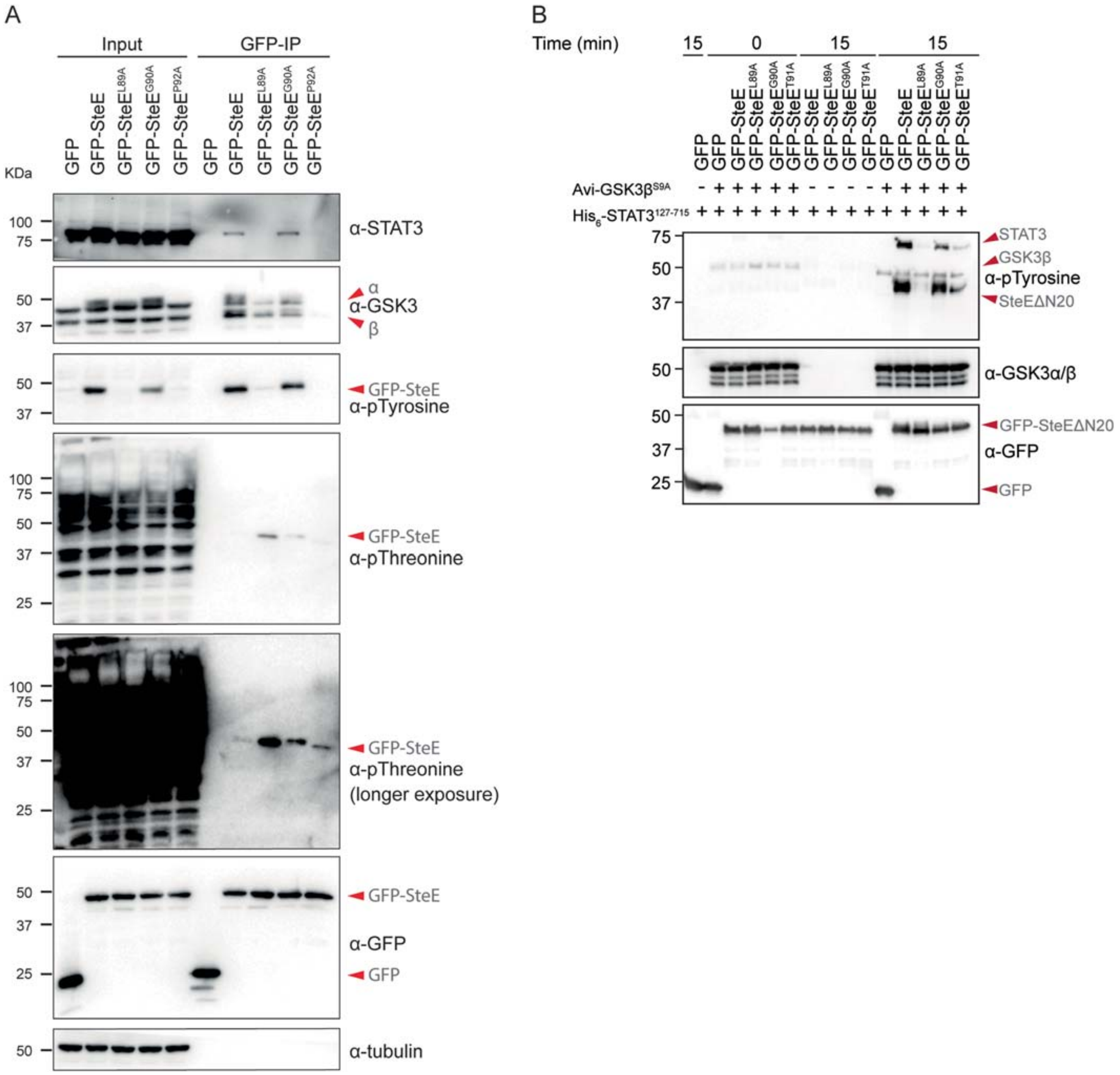

Figure EV4. Residue L89 of SteE is required for tyrosine phosphorylation mediated by GSK3.

(**A**) 293ET cells expressing GFP or the indicated GFP-tagged SteE variant were lysed, and post-nuclear supernatants were subjected to GFP-TRAP immunoprecipitation (GFP:IP). Samples were analysed by immunoblotting. Data represent three independent biological repeats. (**B**) The indicated GFP-tagged proteins were immobilised on beads after expression from *GSK3α/β*$^{-/-}$ 293ET cells. Tyrosine phosphorylation by GSK3β was analysed in an in vitro kinase reaction containing 1 mM ATP, 0.2 μM His$_6$-STAT3$^{127\text{-}715}$, with or without recombinant Avi-GSK3β^{S9A} (0.2 μM). Data represent two biological repeats.

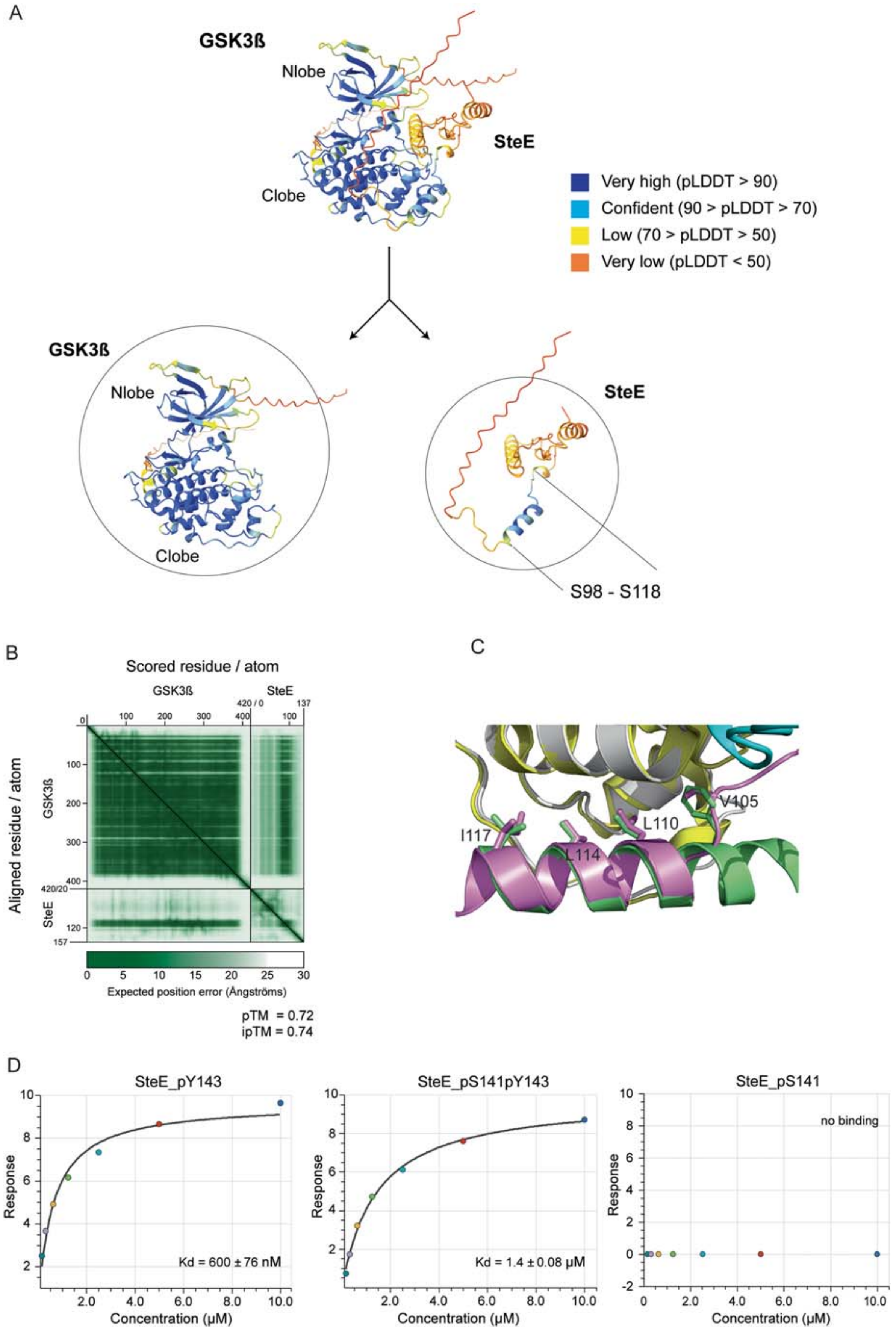
A
GSK3ß
Nlobe
Clobe
SteE
Very high (pLDDT > 90)
Confident (90 > pLDDT > 70)
Low (70 > pLDDT > 50)
Very low (pLDDT < 50)
S98 - S118
B
Scored residue / atom
GSK3ß
SteE
Aligned residue / atom
Expected position error (Ångströms)
pTM = 0.72
ipTM = 0.74
C
V105
L110
I117
L114
D
SteE_pY143
Kd = 600 ± 76 nM
SteE_pS141pY143
Kd = 1.4 ± 0.08 µM
SteE_pS141
no binding
Response
Concentration (µM)

Figure EV5. Model of the GSK3β-SteE complex and role of SteE phosphorylation on Y143.

(**A**) The polypeptide chains are coloured according to pLDDT values, shown in the same orientation as in Fig. 4B. For clarity, GSK3β and SteE are also shown separately to highlight regions on both proteins at various pLDDT confidence levels. (**B**) Expected position error matrix of the GSK3β-SteE complex AlphaFold3 prediction. Black lines indicate chain boundaries, with darker green regions showing higher confidence interactions. (**C**) Structure of GSK3β-axin peptide (yellow/green) superimposed with the predicted GSK3β-SteE interacting helix (magenta), with key hydrophobic interface residues of SteE highlighted. (**D**) Affinity of interaction between the indicated $SteE^{138\text{-}148}$ phosphorylated peptides and $His_6STAT3^{127\text{-}715}$ was determined by biolayer interferometry.

© The Author(s)
